# Underestimation in temporal numerosity judgments computationally explained by population coding model

**DOI:** 10.1038/s41598-022-19941-8

**Published:** 2022-09-17

**Authors:** Takahiro Kawabe, Yusuke Ujitoko, Takumi Yokosaka, Scinob Kuroki

**Affiliations:** NTT Communication Science Laboratories, Nippon Telegraph and Telephone Corporation, Atsugi, Japan

**Keywords:** Psychology, Human behaviour

## Abstract

The ability to judge numerosity is essential to an animal’s survival. Nevertheless, the number of signals presented in a sequence is often underestimated. We attempted to elucidate the mechanism for the underestimation by means of computational modeling based on population coding. In the model, the population of neurons which were selective to the logarithmic number of signals responded to sequential signals and the population activity was integrated by a temporal window. The total number of signals was decoded by a weighted average of the integrated activity. The model predicted well the general trends in the human data while the prediction was not fully sufficient for the novel aging effect wherein underestimation was significantly greater for the elderly than for the young in specific stimulus conditions. Barring the aging effect, we can conclude that humans judge the number of signals in sequence by temporally integrating the neural representations of numerosity.

## Introduction

Animals can behave adaptively by recognizing their own actions and the number of sensory events that occur during those actions. For example, pigeons can discriminate the number of objects in spatial patterns^1^, and bees can judge the number of landmarks to be passed in order to obtain food^2^. Various species of organisms have brain regions that process numerosities^3^, suggesting that there has been evolutionary selection pressure to make them sensitive to numerosity. In other words, we can say that numerosity judgment is a basic ability of living things^4^. Indeed, many species such as dogs^5–7^, elephants^8^, frogs^9,10^, fish^11,12^, parrots^13,14^, and chicks^15,16^ in the animal kingdom can judge the numerosity of external stimuli.

Humans can judge the number of spatially and/or temporally discrete signals. The mechanism of numerosity judgment can be described as information processing in several stages. First, in the relatively early processing stages, the mechanism of numerosity judgment differs depending on the format of the stimuli. In other words, the number of signals presented consecutively in time is processed by different neural populations than the number of signals presented simultaneously in space^17^. In the later processing stages, numerosity is processed abstractly, regardless of the stimulus format or signal presentation modality^17–19^. Furthermore, in the higher stages, internal processing for some mathematical tasks is performed^20,21^. Thus, multiple levels of neural information processing are involved in the judgment of numerosity.

In this study, we discuss the judgment of the number of signals presented in temporal succession, which may be related to the relatively early processing stages described above. The judgment of the number of temporally continuous signals is called a temporal numerosity judgment (TNJ). The brain can judge the number of sequential signals presented in various sensory modalities, including tactile^22–24^, visual^25^, auditory^26,27^, and multisensory^28,29^ modalities, despite modality-specific differences in temporal characteristics^25,26,28^.

One of the hallmarks of TNJ is underestimation of the numerosity. A low level of numerosity can be reported relatively accurately, but as it increases, the reported number of signals becomes smaller than the actual number^24,28,30^. The temporal interval between successively presented signals also affects the underestimation. Specifically, as the time interval becomes shorter, the underestimation becomes stronger^28^.

To the best of our knowledge, there is no research that discusses how the underestimation in the number of sequential signals occurs. We believe that a clarification of the mechanism for underestimation in TNJ will promote the understanding of TNJ itself. In this study, we hypothesized that underestimation is caused by the temporal integration of the activities of neural populations when temporally continuous signals are input to the brain. In general, the underestimation of spatial and temporal extents has been explained in terms of the temporal integration of previous and recent neural signals^31–34^. We assumed that a similar kind of temporal integration would occur in the numerosity dimension, and this would cause underestimation in TNJ.

In this context, what sort of neural representation can be integrated across time in the numerosity dimension? We focused on the responses of neural populations involved in numerosity judgment^18,35^. It is noteworthy that in Nieder’s studies, the neural populations showed systematic responses when the sequence of signals was presented to macaque monkeys. Specifically, when a sequential stimulus consisting of three successive signals was presented, neurons that are selective for “1” predominantly responded to the first signal, neurons that are selective for “2” predominantly responded to the second signal, and neurons that are selective for “3” predominantly responded to the third signal^36^. Therefore, in order to correctly decode that the total number of stimuli is “3”, it is necessary for the brain to focus on the activities of the neural populations that responded to the final (that is, the third) stimulus signal, while discounting the activities of the neural populations that responded to signals prior to the final signal (that is, the first and the second signals). If this discounting process is successful, the number of signals will be accurately determined based on the responses to the final signal. When the discounting process fails, the processing mediating the determination of the total number of stimuli is likely influenced by the population responses to the signals presented prior to the final signal, in addition to the population responses to the final signal, and this may lead to underestimation. In other words, to judge the numerosity of signals in sequence, the brain needs to integrate the population responses across time. Based on this idea, we hypothesized that temporal integration of neural population activities representing numerosity might be the cause of underestimation. The hypothesis may be described at the neural level in the following way: Activities of each population neuron selective to numerosity may undergo synaptic modulations that correspond to the gaining of population activities with a temporal window in our computational model, and the post-synaptic activities are summed by higher-order units to determine the numerosity of vibrations.

**Figure 1:**
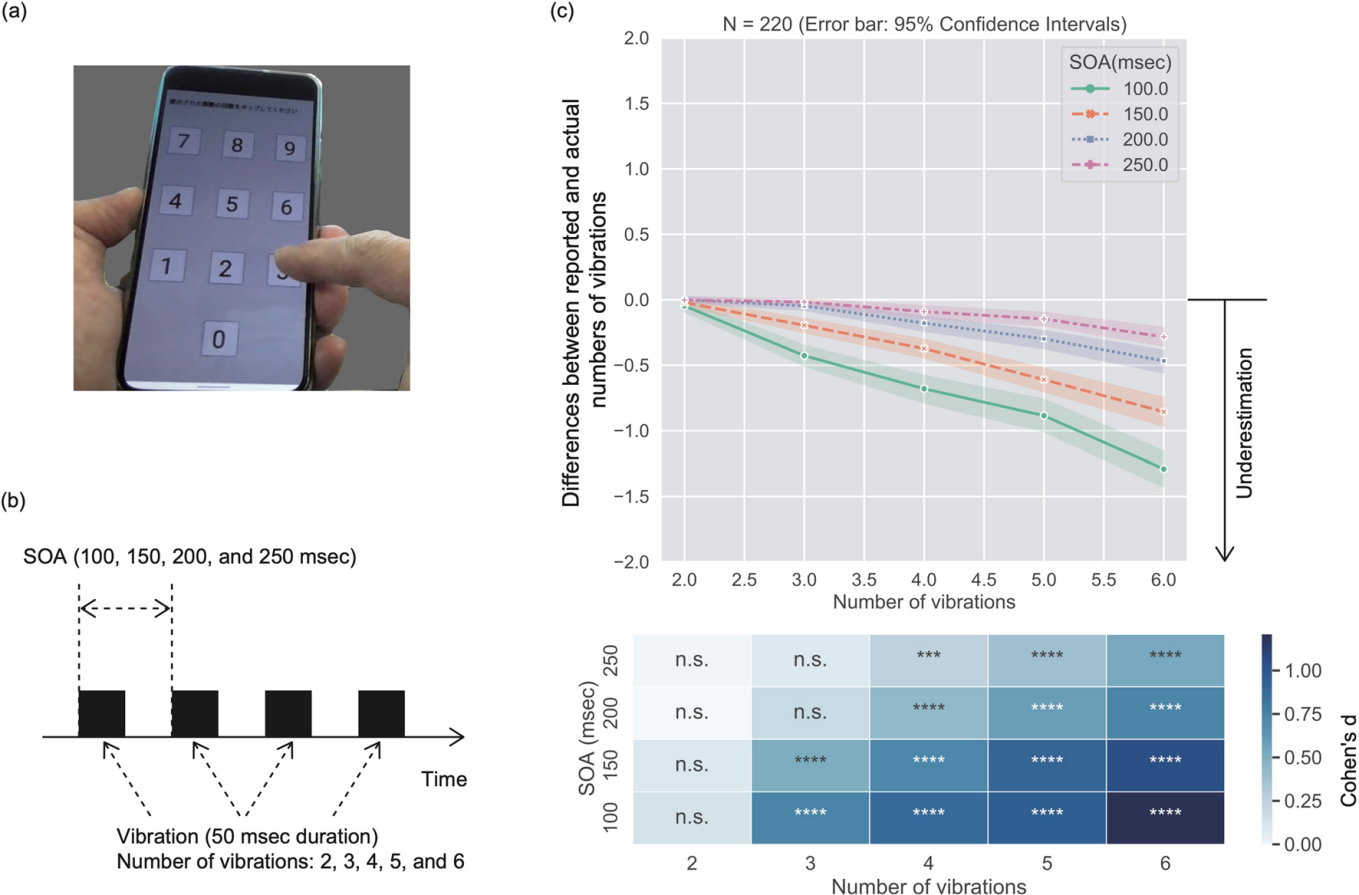
(**a**) A photo of our experimental scene. (**b**) A schematic description of stimuli. (**c**) Upper: Mean difference between actual and reported numbers of vibrations as a function of the number of actual vibrations. The difference was calculated by subtracting the actual from the reported numbers of vibrations. Thus, positive and negative values for the difference denote overestimation and underestimation, respectively. Lower: Heatmap of Cohen’s d for each age band. Asterisks (*p < 0.05, **p < 0.01, ***p < 0.001, ****p < 0.0001) denote the significance level (with correction by the Holm’s method) of one-sample t-tests for the mean difference with an array of zeros. *n.s.* non-significance.

The purpose of this study was to investigate computationally whether the underestimation in the TNJ of successively presented signals could be explained by the temporal integration of numerosity representations, using a neural population coding model. We conducted an online experiment using the vibration function of a smartphone (Fig. 1a). Although smartphone vibrations emit both tactile and auditory signals, we discuss our results focusing mainly on the effect of tactile signals on the TNJ. Since auditory stimuli are transmitted to the ear as air vibrations, they can be affected to a significant degree by differences in the listener’s immediate environment. In contrast, tactile stimuli in the form of smartphone vibrations are transmitted directly from the smartphone to the skin and thus are less affected by differences in the external environment. Thus vibration stimuli were selected as the stimuli for our online experiment, where the external environment cannot be well controlled. We obtained large-scale data from various age bands. Using a neural population coding model, we attempted to explain the overall tendency of TNJ and its underestimation. We also explored whether the model could describe the data of participants in the different age bands, though we do not have a priori expectations about the effect of aging on underestimation in TNJ. As shown in Fig. 1b, we controlled the stimuli with the 4 levels of stimulus onset asynchronies (SOA; 100, 150, 200, and 250 msec) and the 5 levels of the number of smartphone vibrations (2, 3, 4, 5, and 6).

## Results

### Psychophysical experiment

The upper panel of Fig. 1c shows the difference between the reported and the actual numbers of vibrations as a function of the actual number of vibrations. Because the difference was calculated by subtracting the actual from the reported numbers of vibrations, the positive and negative values of the difference indicate overestimation and underestimation in TNJ. In the lower panel, asterisks show the significance of one-sample t-tests to check whether the underestimation deviated significantly from zero. The heat map shows Cohen’s *d* calculated with the following formula,

1 $$\begin{aligned} d = \frac{m-\mu }{s}, \end{aligned}$$

wherein *m* denotes a sample mean, $\mu$ denotes a value against which the sample mean is compared (in this case, 0), and *s* denotes the standard deviation of the sample, which is calculated with n-1 degrees of freedom. The results showed that when the number of vibrations was 2, no significant underestimation occurred. On the other hand, when the number of vibrations was larger than four, a significant underestimation was observed with all SOAs. Moreover, the effect of underestimation was larger with the smaller SOAs. The results are consistent with the previous studies^24,28,30^ showing that the underestimation in TNJ increased with the number of vibrations, and in contrast, decreased with SOA.

**Figure 2:**
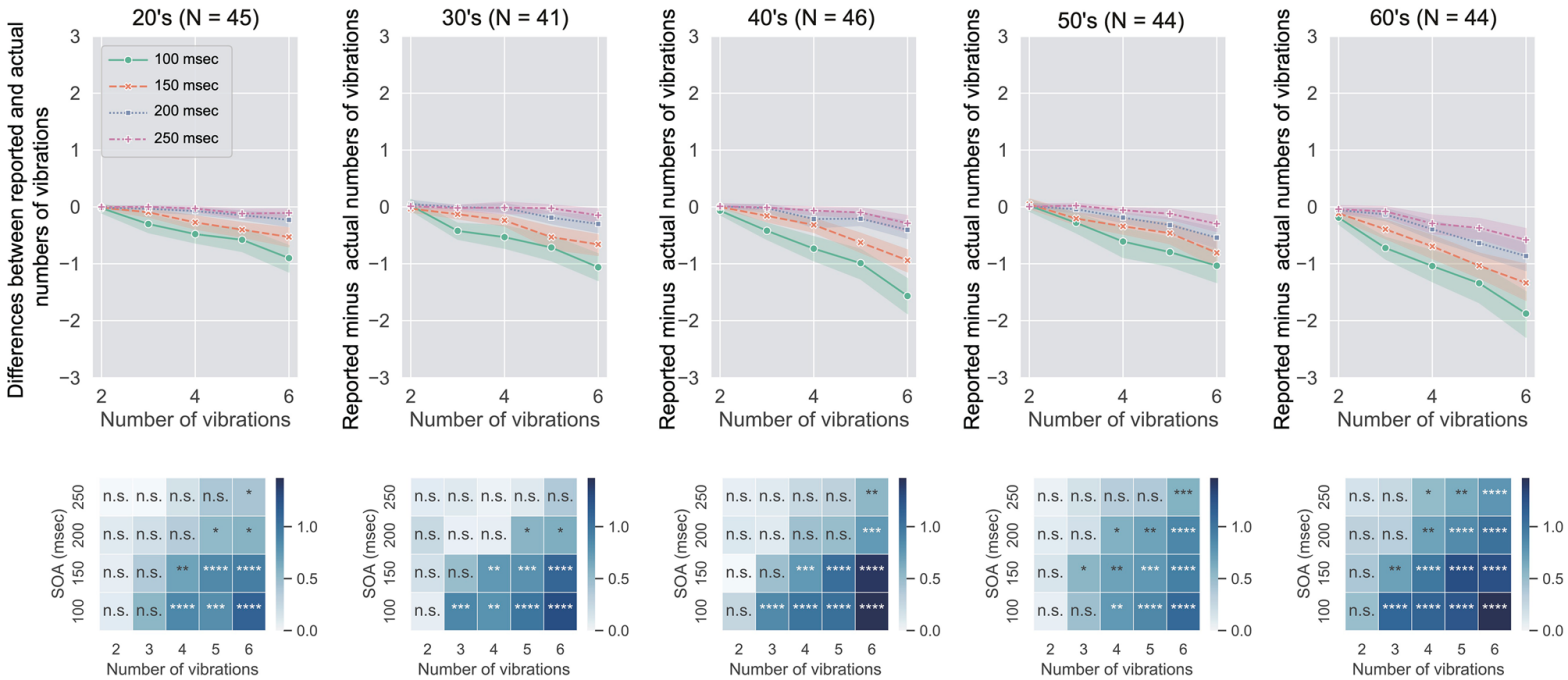
Upper: Mean difference between actual and reported numbers of vibrations as a function of the number of actual vibrations. The data of the 20’s, 30’s, 40’s, 50’s and 60’s age bands are plotted from left to right in Fig. 2. The error bars denote $95\%$ confidence intervals though error bars are not visible because the intervals are so small. Lower: Heatmap of Cohen’s d for each age band. Asterisks (*p < .05, **p < .01, ***p < .001, ***p < .0001) denote the significance level (with correction by the Holm’s method) of one-sample t-tests for the mean difference with an array of zeros. n.s. denotes non-significance. Each graph shows the difference as a function of the number of vibrations for each SOA condition.

Figure 2 shows data which are aggregated in terms of each age band. Using the mean reported number of vibrations, we conducted a three-way mixed ANOVA with age band as a between-participant factor and SOA and the number of vibrations as within-participant factors. The results of the ANOVA are shown in Table 1 and the results of multiple comparison tests and the simple main effect of the significant main effects are shown in Supplementary data 1.

**Table 1:** ANOVA table of mean reported numbers in the experiment.

Factor	SS	df	MS	F-ratio	p-value	$\eta _{G}^{2}$
A (age band)	85.900	4.00	21.475	7.979	<0.0001	0.066
s $\times$ A	578.633	215.00	2.691			
B (SOA)	202.511	1.41	143.805	193.805	<0.0001	0.142
A $\times$ B	13.123	5.63	2.330	3.128	0.007	0.010
s $\times$ A $\times$ B	225.489	302.77	0.745			
C (number of vibrations)	263.322	1.61	163.256	276.546	<0.0001	0.178
A $\times$ C	27.092	6.45	4.199	7.113	<0.0001	0.022
s $\times$ A $\times$ C	204.719	346.78	0.590			
B $\times$ C	67.645	7.24	9.338	70.114	<0.0001	0.0053
A $\times$ B $\times$ C	6.490	28.98	0.224	1.682	0.013	0.005
s $\times$ A $\times$ B $\times$ C	207.429	1557.41	0.133			

Both the main effects of SOA and the number of vibrations were significant. Moreover, the interaction between them was also significant. Further analyses showed that the effect of SOA was significant when the number of vibrations was three or more. The ANOVA results showed that the magnitude of underestimation did not vary with SOA when the number of vibrations was 2.

Our results newly demonstrated the effect of aging on the underestimation in TNJ. The multiple comparison test of the significant main effect of age band showed that the underestimation in the 60’s was significantly larger than that in other age bands. The results indicate that the performance of TNJ changes during the aging process. This idea is also supported by the analysis of Cohen’s d. The lower panels of Fig. 2 show the significance of one-sample t-test and Cohen’s d. Comparing the data among the age bands, Cohen’s d in the 60’s was larger than the one in other age bands, particularly in the condition with the shorter SOAs and the greater number of vibrations. The results indicate that in the 60’s, the underestimation became stronger than in the younger age bands.

### Computational modeling

**Figure 3:**
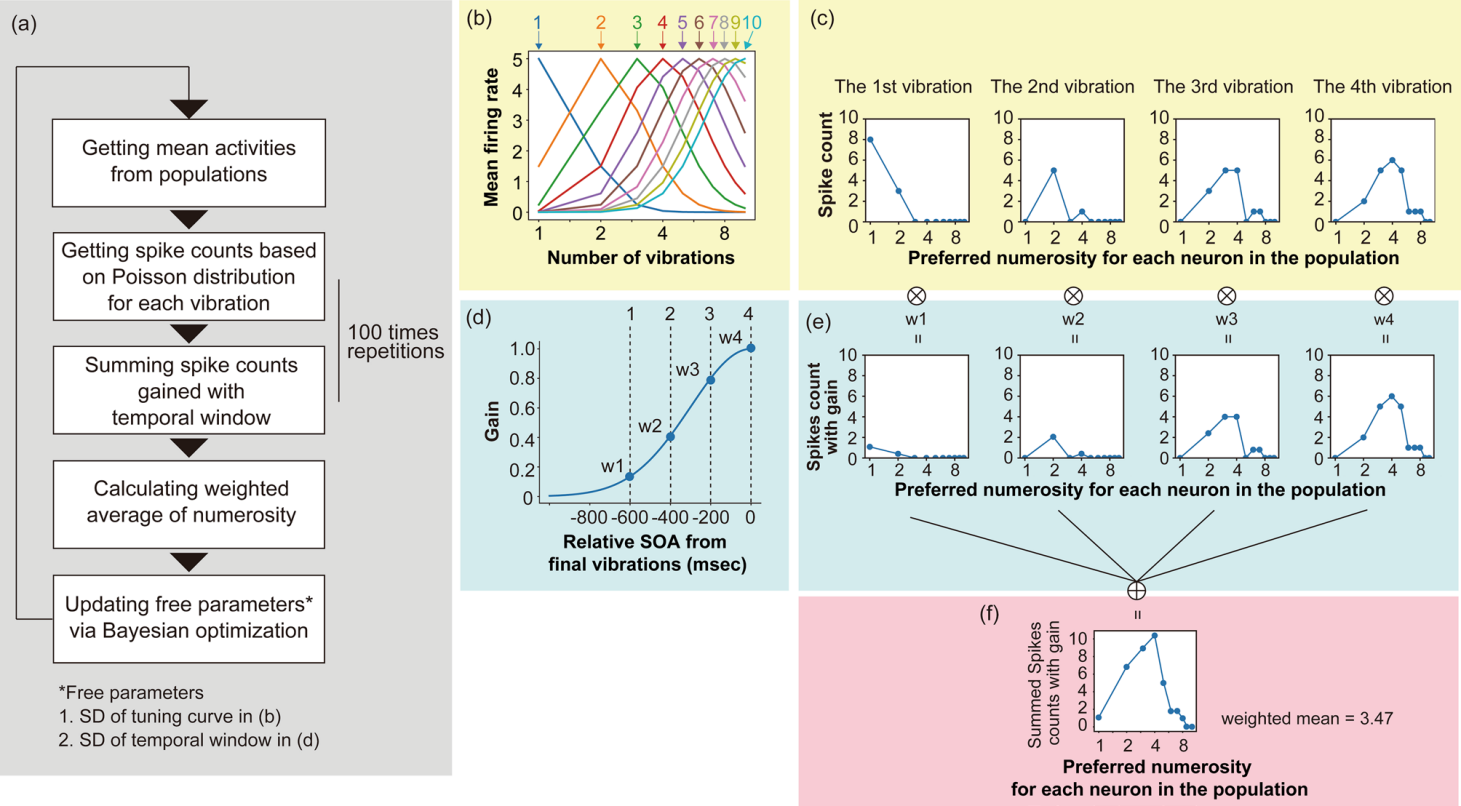
(**a**) A pipeline of processing steps in our model. (**b**) Tuning curves of the population of neurons that are selective to number. Here the standard deviation (SD) of the tuning curves is set at 0.6. (**c**) Spike count that was determined on the basis of Poisson randomness. The Poisson distribution that was used for the determination of the spike count of each neuron had a mean value that was drawn from each tuning curve. (**d**) A schematic explanation of temporal window and gain (w1, w2, w3, and w4). Here the SD of the temporal window is set at 300 ms. (**e**) Spike count with gain. (**f**) Summed version of spike count with gain as a function of preferred numerosity for each neuron.

The purpose of this study was to test computationally whether the temporal integration of neural numerosity representations could explain the underestimation in TNJ. As described in Fig. 3a, our model consists of the following five processing steps.

1. In general, a single neuron selectively responds to a stimulus feature value such as image orientation and velocity^37,38^. As the feature value moves away from the one optimal to the neuron, the neuron’s activities (i.e., mean firing rates) decrease. The transition of mean firing rates as a function of feature values shows the neuron’s tuning curve. A specific feature value in stimuli may thus be read out from the neuron’s activity, provided that the properties of the tuning curve such as mean and variability are known. Nevertheless, it is not practical to try to understand the nature of the tuning curves of all neurons in the brain. As each neuron is often part of a population that selectively responds to similar feature values in the stimulus, previous studies have suggested that it is possible to decode feature values in stimuli more robustly by considering the response of the population of neurons rather than the responses of a single neuron^39^. This superiority of population coding has also been reported for the numerosity judgment^40,41^. Based on these findings, our model also assumes that the brain decodes the number of stimuli by reckoning the population pattern of activity. The tuning curve of the neuron selective to numerosity is defined by a Gaussian function, and is well plotted on a logarithmic scale^42,43^. Thus, in the computation we also assumed populations of neurons each having selectivity to numerosity according to a logarithmic scale (Fig. 3b). Based on the previous study^36^, we assumed that neurons which are tuned to the numerosity $\textit{n}$ will respond to the *n*th vibrations in a sequence. In our model, for the *n*th vibration, the response of all neurons in the population to the numerosity $\textit{n}$ is obtained as the mean firing rate. As shown in Fig. 3b, the population consists of 10 neurons each of which has a tuning curve centered on one of the logarithmic values of 1, 2, 3, 4, 5, 6, 7, 8, 9, and 10. Although we arbitrarily tested populations with several different numbers of neurons (ranging from 7 to 13), there were no obvious (and apparently meaningful) differences among them. In the preliminary simulation, we observed that the model with ten neurons produced the highest performance (See Supplementary Fig. 2). Hence, in this article we report the case of the population with ten neurons.
2. Obtaining spike count based on Poisson distribution for each vibration. Then, based on basic population coding scheme^39,44^, the model obtains spike counts on the basis of a Poisson distribution whose mean corresponds to the mean firing rate (Fig. 3c). We call the pattern of spike counts across the ten neurons as a “population response”. When a sequence contains *n* signals, the population responses to the 1st to the *n*th vibration are calculated. In the simulation, the *n* ranged from 2 to 6.
3. Summing the spike counts gained with temporal window. In TNJ, the decision about the number of signals cannot be made until after the final signal is presented and the brain judges that no further signal will come. Hence, there is an uncertainty about the timing of sequence termination. Due to the uncertainty, the population activity for numerosity needs to be integrated across time. Temporal uncertainty can be modeled by using a Gaussian function along a temporal dimension^45^. We assumed that representation of the number of signals was integrated within a temporal window of integration^46–49^. Assuming a temporal Gaussian function which is centered at the timing of the final signal (Fig. 3d), the model weights the spike count by the Gaussian function (Fig. 3e), and sums the gained spike counts for the preferred numerosity of each neuron in the population (Fig. 3f). Each vibration in a sequence input to the model was mapped along the time dimension according to SOAs. Specifically, the last vibration in the sequence was made to start at 0 ms, and hence, earlier vibrations were mapped to earlier timings according to SOAs. The above temporal window had a peak at 0 ms, which was the onset timing of the last vibration. In our calculation, we repeated a set from the processing step 2 to step 3 100 times and its averaged values were sent to the next stage.
4. Calculating weighted average of numerosity. Based on the summation of the gained spike count, the model decodes the numerosity of signals *N* in a sequence on the basis of the following formula,
  2 $$\begin{aligned} N = 2^{\sum (\frac{S_{i}}{\sum S_{i}}log_{2}i)}, \end{aligned}$$
  wherein *i* denotes the preferred numerosity for each neuron in the population ( *i* = 1, ..., 10) and $S_{i}$ denote the summed spike counts with gain for neuron selective to numerosity *i*.
5. Updating free parameters via Bayesian optimization. The model has two free parameters. One parameter is the standard deviation of the tuning curve of neurons in the population (Fig. 3b). The second is the standard deviation of the temporal window (Fig. 3d). Based on the absolute difference between the weighted average of numerosity and actual number of vibrations, the free parameters are updated by Bayesian optimization^50^ which is implemented in scikit-optimize/scikit-optimize v0.5.2^51^. We adopted Bayesian optimization rather than other methods such as grid search or random search^52,53^ because Bayesian optimization can find optimal parameters more rapidly and efficiently than other methods. In the simulation, the outcome of the optimization after 50 repetitions was taken as giving the final values of the free parameters.

As a novel assumption, the population coding model in the present study assumes the temporal integration of the population responses across time. In general, population coding uses a single set of population responses which do not take temporal dimensions into account^39^. To model computationally the representation of working memory, a previous study^54^ assumed the temporal drifting of the activity of populations over time. We did not employ the drifting as a possible computation scheme of underestimation because the drifting that is modeled by using Brownian motion along a stimulus dimension does not seem to be appropriate to describe the underestimation, which is a unidirectionally biased phenomenon.

**Figure 4:**
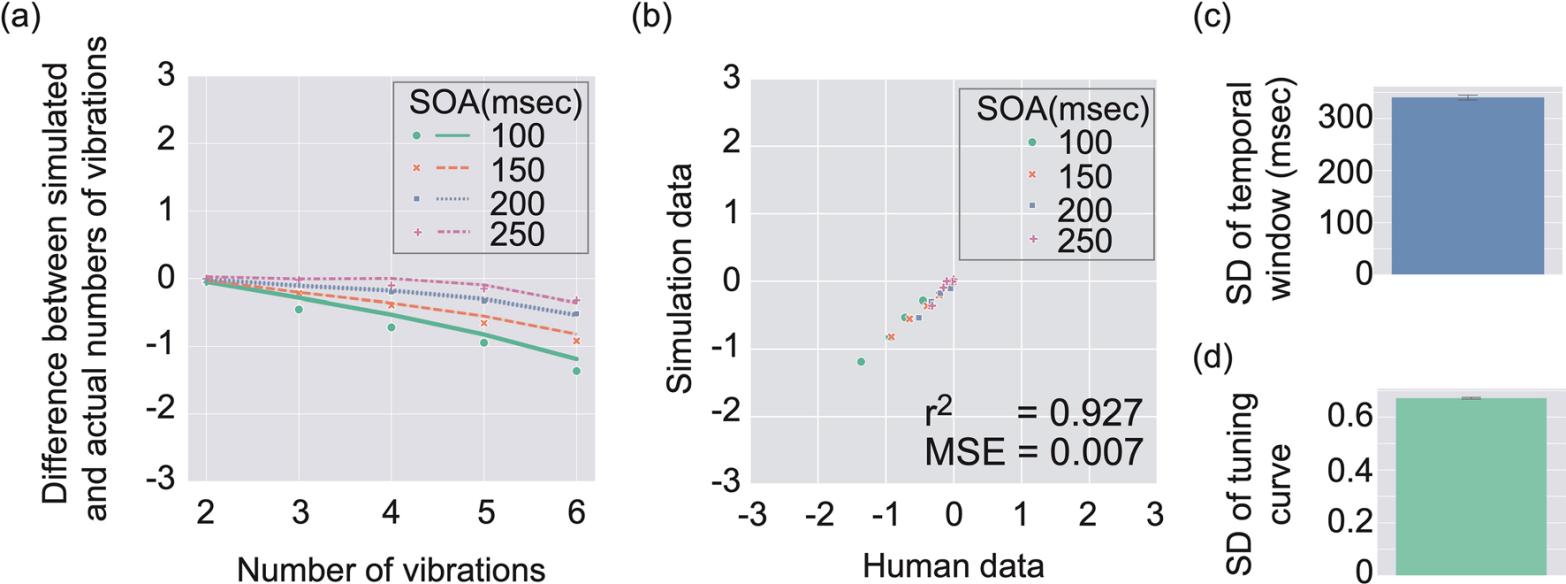
Simulation results. (**a**) Difference between simulated and actual numbers of vibrations is shown by using lines as a function of the number of vibrations in stimuli. Error bars indicate $95\%$ confidence intervals (N $=$ 20). Human data are replotted by using markers. (**b**) A correlation plot between human data (horizontal axis) and simulation data (vertical axis). (**c**) Mean SD of temporal window. Error bar denotes $95\%$ confidence intervals (*N* $=$ 20). (**d**) Mean SD of tuning curves. Error bar denotes $95\%$ confidence intervals (*N* $=$ 20).

By using the model, we conducted the optimization 20 times and obtained 20 sets of the free parameters which were optimized by the simulation. Based on each set of the free parameters, our model output the simulated number of vibrations when the actual number of vibrations (2, 3, 4, 5, and 6) and SOAs (100, 150, 200, and 250 msec) were varied. Figure  4a shows the mean difference between simulated and actual numbers of vibrations. The model data indicated by lines apparently fits to the human data indicated by markers. We calculated r-squared between the mean human data and the mean simulation data and found that the r-squared value was high ($r^2$=0.927) and prediction error (mean squared error: MSE) was low (*MSE* $=$ 0.007), indicating that our model successfully accounted for the human data. Figure 4c,d show the SDs of temporal window and tuning curves, respectively. Both of the values apparently fell in the range that is reasonable in terms of human temporal processing and number processing. To ascertain whether the model could explain data that were not employed for the model training, we conducted a fivefold cross-validation of the model, which was iterated 20 times with different data splits, and confirmed that the prediction of data unseen by the model was also reasonably good (Supplementary Fig. 1).

Although the model prediction was generally good, it seemed that the fitting of the model did not look so great when the SOA was short (in particular, 100 ms). As described above, the participants in the 60’s age band showed a larger underestimation than the participants in the other age groups. Thus, there was a possibility that our model could not capture the characteristic of underestimation in TNJ by the 60’s age group.

**Figure 5:**
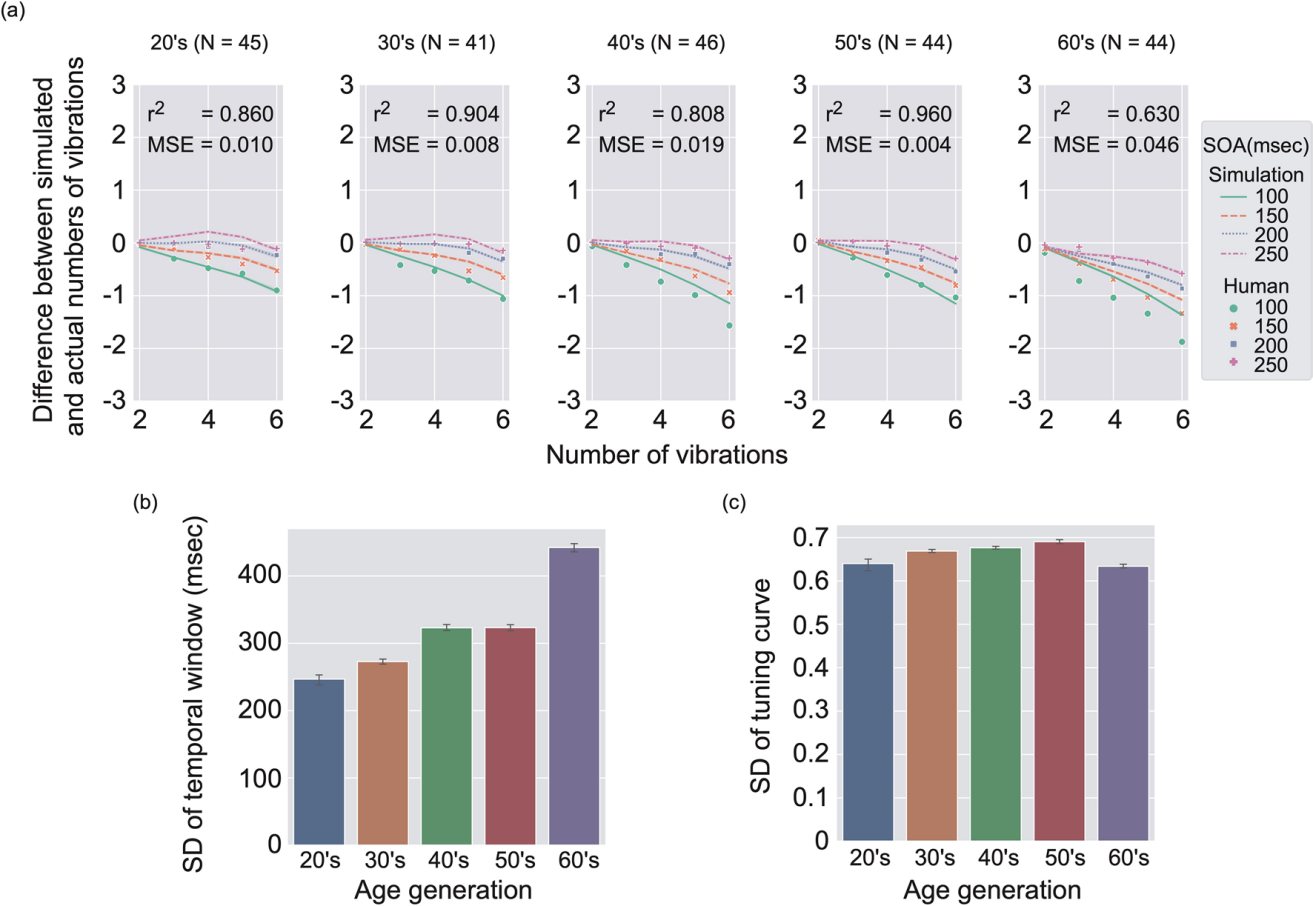
Simulation results for each age band. (**a**) Difference between simulated and actual numbers of vibrations is shown by using lines as a function of the number of vibrations in stimuli. Error bars indicate $95\%$ confidence intervals (N $=$ 20). Human data are replotted by using markers. From left to right, panels for 20’s, 30’s, 40’s, 50’s and 60’s age band data are shown. (**b**) Mean SD of temporal window as a function of age band. Error bars denote $95\%$ confidence intervals (*N* $=$ 20). (**d**) Mean SD of tuning curves as a function of age bands. Error bars denote $95\%$ confidence intervals (*N* $=$ 20).

To check this possibility, by using the identical model, we simulated underestimation in TNJ for each age band. Figure 5a shows the results of simulation for each age band. As expected, the success of the model predictions depended on the age band of the participants. As long as we checked r-squared and MSE, the model prediction was good in general, while the prediction was not so compelling for the data of the 60’s age band when the SOAs were 100 and 150 ms, while the data in the longer SOA conditions such as 200 and 250 ms could be well predicted by our model. The results indicate that our model could predict the data of various levels of age band except for the data in the short SOA conditions of some age bands, in particular the 60’s age band. Fig. 5b shows the fitted SD of temporal window for each age band. The results showed that the SD of the temporal window increased with the age band though the fitted parameter for 60’s is not reliable due to the unsuccessful prediction of human data. Figure 5c shows the fitted SD of the tuning curve for each age band. The results showed that the SD of the tuning curve increased with the age band. Although the SD of the tuning curve dropped for the 60’s age band, we again consider the fitted parameter for the 60’s is not reliable due to the unsuccessful prediction of human data.

## Discussion

The results of the present study are consistent with our idea that temporal integration of numerosity representation underlies underestimation in TNJ. Specifically, the weighted average of population responses that were obtained across time generally accounted for human data. The results indicate that the brain integrates the neural evidence about the number of vibrations across time and makes a decision on numerosity of signals in a sequence. Because our model assumes some novel aspects of information processing such as the temporal integration of population responses and calculation of their weighted average, further evidence in neuroscience is required to test whether the algorithm in our model is indeed implemented in biological neural processing.

The present study first reported the aging effect in TNJ. Specifically, the underestimation was greater in the 60’s than other younger age-groups. A closer look at the data for each age band showed that our model could not explain the underestimation for all age bands. In particular, large underestimations reported by the participants of the 60’s age band in the case of short SOA and large vibration number conditions were not covered by our model. We speculate that to explain the aging effect, the model needs to be improved by implementing one or more additional factors that can simultaneously take the effects of the SOA and the numbers of vibrations into account.

In our model, the averaged spike counts gained in a time window are sent to the next stage. The contribution of this study is to show that, from an algorithmic point of view, the averaged spike counts can be a valid input to the next stage. On the other hand, it should be noted that this calculation is not based on neuronal evidence that the higher-order units assumed to be located in the next stage receive the averaged spike counts. The calculation of averaged spike counts could be replaced by other types of statistics for spike counts. For example, in our model, the averaged spike count could computationally be replaced with summed spike counts since the calculation in the next stage is a weighted average of the numbers, which is not affected by whether the input is an averaged or summed spike count. What information is actually sent to the higher-order units would need to be carefully considered in light of the neurophysiological findings.

A potential factor for the aging effect is temporal sensitivity. It is known that the sensitivity to tactile^55–61^ and auditory temporal structure^57,62^ declines with aging. One of the previous studies focused on the temporal discrimination threshold for a sequence of two vibrations and found that the threshold increased with aging^60^. Moreover, another study^56^ has reported that temporal masking of a target vibration by another preceding vibration stimulus was stronger in the elderly than the young. A previous study^28^ also interpreted the underestimation in multisensory TNJ by in terms of sensory persistence. In our experiment, no significant underestimation was observed when the number of vibrations was 2 even for the 60’s age group, which is not always consistent with temporal masking which reportedly occurs more strongly in the elderly than in the young. On the other hand, signals in the middle of a long sequence likely undergo forward and/or backward masking. Further studies are warranted to substantiate this speculation of the aging effect in TNJ.

As for the question of why underestimation occurs, we have a tentative answer that underestimation does not always have a positive biological meaning. As shown in the previous study^28^, it is possible for participants to judge the number of signals in sequence accurately when SOA is long, but not when it is short. Given this, it seems that the brain may not be optimized for such a task like judging the numerosity of signals in rapid succession. Eventually, more general information processing parts of the brain, such as the temporal integration of signals as the model in the present study assumes, will affect the judgment of numerosity, resulting in underestimation.

## Methods of behavioral experiments

### Participants

Two hundred and fifty-six people (113 females) participated in this experiment. Their mean age was 45.14 (SD 13.60). Almost the same number of people in each age band participated in the experiment (51, 50, 52, 51 and 52 people for 20’s, 30’s, 40’s, 50’s and 60’s age bands). A Japanese crowdsourcing research company recruited the participants online and paid for their participation. The participants were unaware of the specific purpose of the experiment. Ethical approval for this study was obtained from the Ethics Committee at Nippon Telegraph and Telephone Corporation (Approval number: R02-009 by NTT Communication Science Laboratories Ethics Committee). The experiments were conducted according to principles that have their origin in the Declaration of Helsinki. Written informed consent was obtained from all observers in this study.

### Apparatus

We conducted the experiment online. For this reason, the experiment was carried out by using a smartphone owned by each participant.

### Stimuli

**Figure 6:**
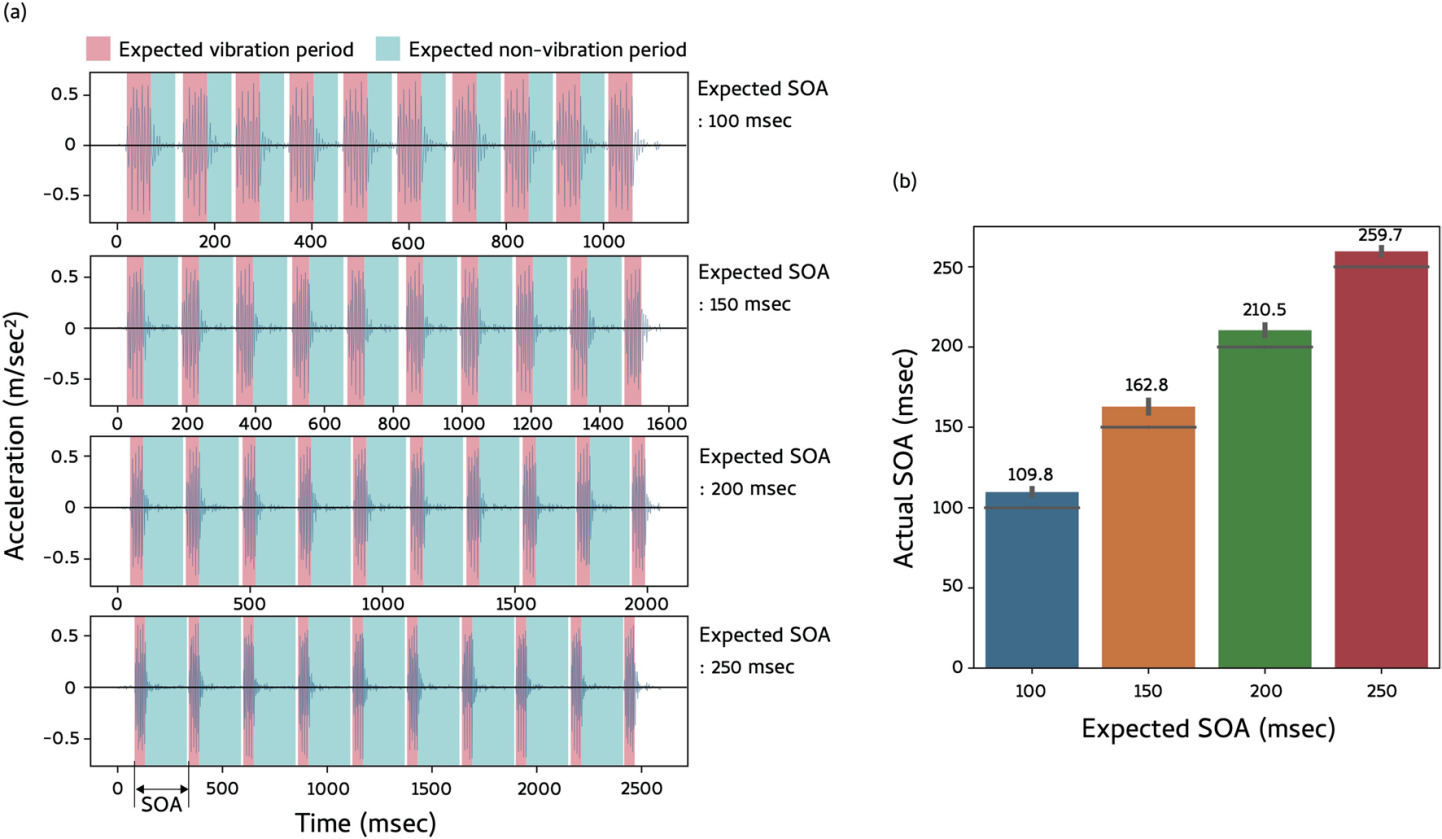
(**a**) Acceleration patterns of Google Pixel 5 when subject to a train of ten vibrations at four levels of SOA. (**b**) Mean SOAs as a function of expected SOA. Values above each bar indicate mean SOAs for each SOA condition. Error bars denote standard errors of mean.

Stimuli were defined by using a Javascript API (navigator.vibrate) which works on the Chrome browser in Android smartphones. For example, by describing “navigator.vibrate([50, 100, 50, 100, 50])” in the script, we presented a vibration train with the vibration duration of 50 ms, SOA of 150 ms, and the number of vibrations of 3. In our experiment, the duration of each vibration was fixed at 50 ms. Moreover, we used four levels of SOA (100, 150, 200, and 250 ms) and six levels of the number of vibrations (0, 2, 3, 4, 5, and 6). The number of vibrations in the stimuli was determined in accordance with the previous study^23^ showing a robust interaction between the number of vibration stimuli and inter-vibration temporal intervals on the tactile TNJ. We employed the condition with 0 vibrations because we wanted to use this condition as catch trials to exclude from the analysis any participants who did not seriously perform the task. To ascertain whether our manipulation of the duration and SOA of vibrations properly worked, we measured the physical vibration of smartphones during stimulation by using an accelerometer implemented in the smartphones. Figure 6a shows the acceleration pattern of Google Pixel 5 on the desk when subject to a train of ten vibrations at four levels of SOA. We calculated mean SOAs and plot them in Fig. 6b. Mean SOAs deviated by approximately 10 ms from the expected SOA. Because the deviation was constant across four levels of SOA and the magnitude of deviation was not large, we judged that it was possible to use smartphones to conduct the experiment. Besides Google Pixel 5, we observed similar acceleration patterns for SONY Xperia, Sharp Aquos sense 4, and Samsung Galaxy note 10. Thus, our manipulation of the vibration by using the API was reproduced in various types of smartphone.

### Procedure

During the experiment, participants were instructed to hold a smartphone in their hand. The participants could register smartphone vibrations as tactile and auditory sensations, and it is possible that they could have used both tactile and auditory signals to perform the task. Each trial was initiated by tapping a black rectangle presented on the screen. The black rectangle was presented among three white rectangles. The positions of these rectangles were shuffled from trial to trial to increase the attentional engagement of the participants toward the task. In a period of 500 ms, a train of vibrations was presented. Five-hundred ms after the start of the train of vibrations, buttons each containing one of ten digits were presented on the screen (see Fig. 1a). The task of the participants was to report the number of vibrations they felt by tapping the button having a digit that corresponded to their judgment. After 1000 ms, the next trial began. Each participant performed 96 trials consisting of 4 (SOAs: 100, 150, 200, and 250 ms) $\times$ 6 (numbers of vibrations: 0, 2, 3, 4, 5, and 6) $\times$ 4 repetitions. The order of the trials was pseudo-randomized for a participant and also varied across the participants. The trials were performed in a single session. Although we did not measure how long each participant took to complete the session, from the preliminary testing it was expected that each participant would take 5–10 min to complete the task.

### Analysis

Based on the performance in the catch trials, from our analysis, we excluded participants who did not participate in the trials seriously. Specifically, we wanted to exclude those participants who paid insufficient attention to the task in the catch trials, in which no vibration was presented, and the participants were expected to report 0 as the number of vibrations. We excluded the data obtained from participants whose mean percentage of correct reports was less than $93.75\%$ (15 correct reports out of 16 cases) in the catch trials. Moreover, we also wanted to exclude data obtained from participants who paid insufficient attention to the task in the trials with vibrations. Therefore, we excluded the data of participants who reported “0” in more than $5\%$ of trials with vibrations. We adopted the exclusion criteria with approximately $5\%$ error rates, keeping in mind both the appropriate removal of those who were not performing the task seriously and securing sufficient data so as not to reduce the power of the test. As a result, the number of participants excluded from further analysis in each age band was 6, 9, 6, 7, and 8 for those in their 20s, 30s, 40s, 50s and 60s, respectively, and hence, the number of participants that underwent analysis was 45, 41, 46, 44, and 44 in each of the respective age band. For each of the participants, the mean reported number of vibrations was calculated by averaging four reports in each condition. We also subtracted the actual number of vibrations from the reported number of vibrations. The positive and negative values of the subtraction indicated the overestimation and underestimation of the number of vibrations. The calculated values were subject to a three-way mixed ANOVA with age bands as a between-participant factor and SOA and the number of vibrations as within-participant factors. Degrees of freedom were adjusted by Greenhouse-Geisser’s Epsilon. The results are shown in Table  1.

## Supplementary Information

Supplementary Figures.

Supplementary Information.

Supplementary Information.

## Data Availability

Supplementary material contains the raw data of this study (Exp1_raw_data (for submission).csv). Further information is also available upon request to the first author (TK) of this study.
