# Supplementary material for: Underestimation in temporal numerosity judgments computationally explained by population coding model
Source: Sci Rep. 2022 Sep 17;12:15632. doi: 10.1038/s41598-022-19941-8 (PMC9482646; doi:10.1038/s41598-022-19941-8)
Supplement: Supplementary file 1 — Supplementary Figures. [file 41598_2022_19941_MOESM1_ESM.pdf]

Supplementary Figures

Supplementary Figure 1

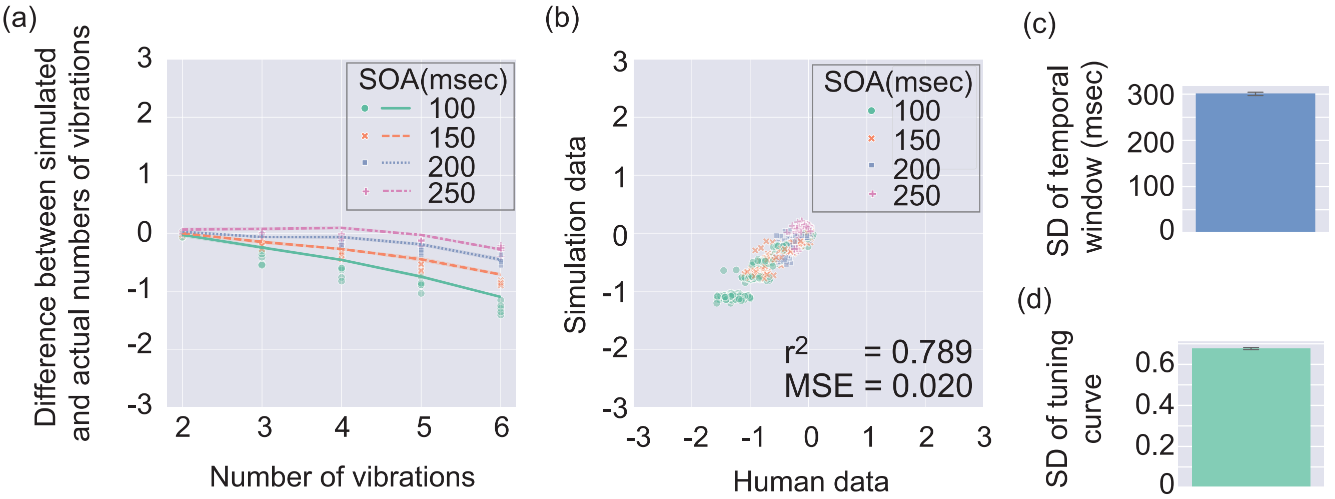

Supplementary Figure 1. The results of five-fold cross-validations that were repeated twenty times. (a) Difference between simulated and actual numbers of vibrations is shown by using lines as a function of the number of vibrations in stimuli. Error bars indicate 95% confidence intervals (N = 100). Human data are replotted by using markers. (b) A correlation plot between human data (horizontal axis) and simulation data (vertical axis). (c) Mean SD of temporal window. Error bar denotes 95% confidence intervals (N = 100). (d) Mean SD of tuning curves. Error bar denotes 95% confidence intervals (N = 100).

Supplementary Figure 2

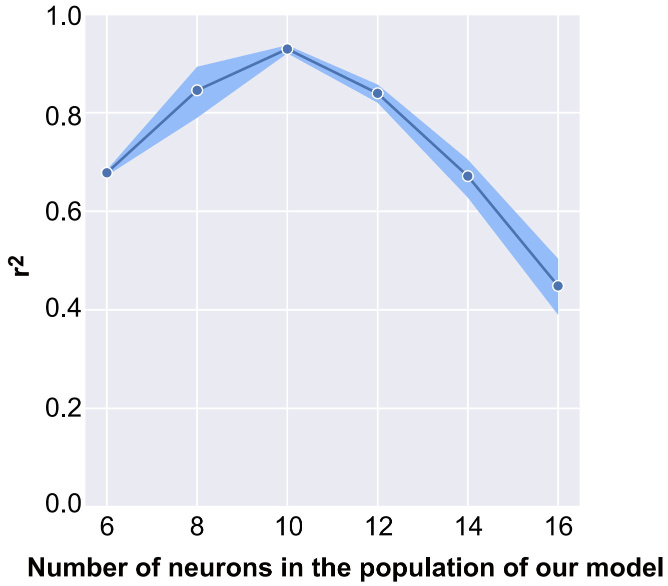

Supplementary Figure 2. R-squared between the mean human data and the mean simulation data as a function of the number of neurons in the population of our model. Error stripe denotes 95% confidence intervals for 20 times iterations of simulation.
