# Supplementary material for: Underestimation in temporal numerosity judgments computationally explained by population coding model
Source: Sci Rep. 2022 Sep 17;12:15632. doi: 10.1038/s41598-022-19941-8 (PMC9482646; doi:10.1038/s41598-022-19941-8)
Supplement: Supplementary file 2 — Supplementary Information. [file 41598_2022_19941_MOESM2_ESM.pdf]

[AsBC-Type Design]

This output was generated by anovakun 4.8.5 under R version 4.0.2.
It was executed on Mon Oct 11 18:22:21 2021.

<<Factors and their levels>>

A: Age populations
a1: 20's
a2: 30's
a3: 40's
a4: 50's
a5: 60's

B: ISI:
b1: 50 ms
b2: 100 ms
43: 150 ms
b1: 200 ms

C: Number of vibrations
c1: 2
c2: 3
c3: 4
c4: 5
c5: 6

<< DESCRIPTIVE STATISTICS >>

A	B	C	n	Mean	S.D.
a1	b1	c1	45	-0.0222	0.2601
a1	b1	c2	45	-0.3000	0.5503
a1	b1	c3	45	-0.4778	0.5509
a1	b1	c4	45	-0.5778	0.7108
a1	b1	c5	45	-0.9000	0.7984
a1	b2	c1	45	-0.0111	0.1406
a1	b2	c2	45	-0.0944	0.2516
a1	b2	c3	45	-0.2722	0.4018
a1	b2	c4	45	-0.4000	0.4689
a1	b2	c5	45	-0.5278	0.5671
a1	b3	c1	45	-0.0111	0.1187
a1	b3	c2	45	-0.0333	0.1735
a1	b3	c3	45	-0.0722	0.2038
a1	b3	c4	45	-0.1444	0.2743
a1	b3	c5	45	-0.2278	0.3946
a1	b4	c1	45	0.0000	0.0000
a1	b4	c2	45	0.0000	0.0754
a1	b4	c3	45	-0.0278	0.1619
a1	b4	c4	45	-0.1167	0.2900

a1	b4	c5	45	−0.1056	0.2528
a2	b1	c1	41	0.0244	0.3298
a2	b1	c2	41	−0.4207	0.4791
a2	b1	c3	41	−0.5305	0.6782
a2	b1	c4	41	−0.7134	0.7171
a2	b1	c5	41	−1.0610	0.8344
a2	b2	c1	41	−0.0305	0.1695
a2	b2	c2	41	−0.1280	0.2569
a2	b2	c3	41	−0.2378	0.3160
a2	b2	c4	41	−0.5305	0.6713
a2	b2	c5	41	−0.6585	0.6040
a2	b3	c1	41	0.0488	0.2033
a2	b3	c2	41	−0.0061	0.1044
a2	b3	c3	41	−0.0183	0.2704
a2	b3	c4	41	−0.1890	0.3344
a2	b3	c5	41	−0.2988	0.4944
a2	b4	c1	41	0.0122	0.1364
a2	b4	c2	41	−0.0183	0.1713
a2	b4	c3	41	−0.0122	0.3352
a2	b4	c4	41	−0.0244	0.2359
a2	b4	c5	41	−0.1463	0.3831
a3	b1	c1	46	−0.0652	0.2496
a3	b1	c2	46	−0.4185	0.4946
a3	b1	c3	46	−0.7337	0.7273
a3	b1	c4	46	−0.9891	0.9617
a3	b1	c5	46	−1.5652	1.0832
a3	b2	c1	46	0.0000	0.0527
a3	b2	c2	46	−0.1576	0.3389
a3	b2	c3	46	−0.3152	0.4231
a3	b2	c4	46	−0.6250	0.5980
a3	b2	c5	46	−0.9402	0.6671
a3	b3	c1	46	0.0109	0.0906
a3	b3	c2	46	−0.0217	0.2347
a3	b3	c3	46	−0.2120	0.4624
a3	b3	c4	46	−0.2065	0.4160
a3	b3	c5	46	−0.4022	0.5413
a3	b4	c1	46	0.0109	0.1577
a3	b4	c2	46	−0.0109	0.1173
a3	b4	c3	46	−0.0652	0.2710
a3	b4	c4	46	−0.0978	0.3139
a3	b4	c5	46	−0.2880	0.5296
a4	b1	c1	44	0.0227	0.3316
a4	b1	c2	44	−0.2784	0.6057
a4	b1	c3	44	−0.6080	0.8130
a4	b1	c4	44	−0.7955	0.9265
a4	b1	c5	44	−1.0341	0.9684
a4	b2	c1	44	0.0455	0.3015
a4	b2	c2	44	−0.2045	0.3707
a4	b2	c3	44	−0.3409	0.4944
a4	b2	c4	44	−0.4602	0.6122
a4	b2	c5	44	−0.8068	0.8758

a4	b3	c1	44	0.0114	0.1422
a4	b3	c2	44	-0.0398	0.2219
a4	b3	c3	44	-0.1875	0.3150
a4	b3	c4	44	-0.3182	0.4649
a4	b3	c5	44	-0.5398	0.6170
a4	b4	c1	44	0.0057	0.1373
a4	b4	c2	44	0.0227	0.1301
a4	b4	c3	44	-0.0568	0.1693
a4	b4	c4	44	-0.1193	0.3120
a4	b4	c5	44	-0.2955	0.5037
a5	b1	c1	44	-0.1875	0.3855
a5	b1	c2	44	-0.7216	0.6630
a5	b1	c3	44	-1.0398	0.9384
a5	b1	c4	44	-1.3409	1.1011
a5	b1	c5	44	-1.8750	1.2912
a5	b2	c1	44	-0.1136	0.2825
a5	b2	c2	44	-0.3920	0.5665
a5	b2	c3	44	-0.6932	0.7089
a5	b2	c4	44	-1.0341	0.8134
a5	b2	c5	44	-1.3352	1.0633
a5	b3	c1	44	-0.0568	0.1857
a5	b3	c2	44	-0.1307	0.3906
a5	b3	c3	44	-0.3920	0.5741
a5	b3	c4	44	-0.6364	0.7576
a5	b3	c5	44	-0.8636	0.8499
a5	b4	c1	44	-0.0398	0.2347
a5	b4	c2	44	-0.0795	0.3270
a5	b4	c3	44	-0.2898	0.5730
a5	b4	c4	44	-0.3693	0.5943
a5	b4	c5	44	-0.5795	0.7601

<< SPHERICITY INDICES >>

== Mendoza's Multisample Sphericity Test and Epsilons ==

Effect	Lambda	approx.Chi	df	p		LB	GG	HF	CM
Global	0.0000	4708.2404	949	0.0000	***	0.0526	0.1952	0.1990	0.1990
B	0.0000	505.5132	29	0.0000	***	0.3333	0.4694	0.4716	0.4715
C	0.0000	638.2447	49	0.0000	***	0.2500	0.4032	0.4058	0.4057
B x C	0.0000	1286.8026	389	0.0000	***	0.0833	0.6036	0.6268	0.6266

LB = lower.bound, GG = Greenhouse-Geisser
HF = Huynh-Feldt-Lecoutre, CM = Chi-Muller

<< ANOVA TABLE >>

== Adjusted by Greenhouse-Geisser's Epsilon ==
== This data is UNBALANCED!! ==
== Type III SS is applied. ==

Source	SS	df	MS	F-ratio	p-value		G.eta^2
A	85.9002	4	21.4751	7.9794	0.0000	***	0.0660
s x A	578.6335	215	2.6913				
B	202.5116	1.41	143.8052	193.0917	0.0000	***	0.1427
A x B	13.1230	5.63	2.3297	3.1281	0.0066	**	0.0107
s x A x B	225.4887	302.77	0.7448				
C	263.3223	1.61	163.2561	276.5455	0.0000	***	0.1780
A x C	27.0923	6.45	4.1992	7.1132	0.0000	***	0.0218
s x A x C	204.7196	346.78	0.5903				
B x C	67.6450	7.24	9.3384	70.1139	0.0000	***	0.0527
A x B x C	6.4899	28.98	0.2240	1.6817	0.0133	*	0.0053
s x A x B x C	207.4292	1557.41	0.1332				
Total	1885.3868	4399	0.4286				

+p < .10, *p < .05, **p < .01, ***p < .001

<< POST ANALYSES >>

< MULTIPLE COMPARISON for "A" >

== Holm's Sequentially Rejective Bonferroni Procedure ==

== The factor < A > is analysed as independent means. ==
== Alpha level is 0.05. ==

A	n	Mean	S.D.
a1	900	-0.2161	0.4577
a2	820	-0.2470	0.5286
a3	920	-0.3546	0.6534
a4	880	-0.2989	0.6136
a5	880	-0.6085	0.8659

Pair	Diff	t-value	df	p	adj.p	
a1-a5	0.3924	5.0456	215	0.0000	0.0000	a1 > a5 *
a2-a5	0.3616	4.5408	215	0.0000	0.0001	a2 > a5 *
a4-a5	0.3097	3.9594	215	0.0001	0.0008	a4 > a5 *
a3-a5	0.2539	3.2823	215	0.0012	0.0084	a3 > a5 *
a1-a3	0.1385	1.8008	215	0.0731	0.4388	a1 = a3
a2-a3	0.1077	1.3666	215	0.1732	0.8659	a2 = a3
a1-a4	0.0828	1.0640	215	0.2885	1.0000	a1 = a4
a3-a4	-0.0558	0.7208	215	0.4718	1.0000	a3 = a4
a2-a4	0.0519	0.6519	215	0.5151	1.0000	a2 = a4
a1-a2	0.0308	0.3894	215	0.6974	1.0000	a1 = a2

< MULTIPLE COMPARISON for "B" >

== Holm's Sequentially Rejective Bonferroni Procedure ==
== The factor < B > is analysed as dependent means. ==
== Alpha level is 0.05. ==

B	n	Mean	S.D.
b1	1100	-0.6643	0.8848
b2	1100	-0.4106	0.6349
b3	1100	-0.1975	0.4593
b4	1100	-0.1076	0.3653

Pair	Diff	t-value	df	p	adj.p	
b1-b4	-0.5567	15.2457	215	0.0000	0.0000	b1 < b4 *
b2-b4	-0.3029	14.5156	215	0.0000	0.0000	b2 < b4 *
b1-b3	-0.4669	14.1617	215	0.0000	0.0000	b1 < b3 *
b2-b3	-0.2131	13.5767	215	0.0000	0.0000	b2 < b3 *
b1-b2	-0.2538	10.4522	215	0.0000	0.0000	b1 < b2 *
b3-b4	-0.0898	7.9403	215	0.0000	0.0000	b3 < b4 *

< SIMPLE EFFECTS for "A x B" INTERACTION >

A	B	n	Mean	S.D.
a1	b1	225	-0.4556	0.6650
a1	b2	225	-0.2611	0.4367
a1	b3	225	-0.0978	0.2618
a1	b4	225	-0.0500	0.1948
a2	b1	205	-0.5402	0.7220
a2	b2	205	-0.3171	0.5056
a2	b3	205	-0.0927	0.3339
a2	b4	205	-0.0378	0.2725
a3	b1	230	-0.7543	0.9153
a3	b2	230	-0.4076	0.5746
a3	b3	230	-0.1663	0.4104
a3	b4	230	-0.0902	0.3284
a4	b1	220	-0.5386	0.8465
a4	b2	220	-0.3534	0.6301
a4	b3	220	-0.2148	0.4362
a4	b4	220	-0.0886	0.3080
a5	b1	220	-1.0330	1.0855
a5	b2	220	-0.7136	0.8489
a5	b3	220	-0.4159	0.6696
a5	b4	220	-0.2716	0.5642

Effect	Lambda	approx.Chi	df	p		LB	GG	HF	CM
B at a1	0.0000	99.6523	5	0.0000	***	0.3333	0.4594	0.4693	0.4665
B at a2	0.0000	93.3090	5	0.0000	***	0.3333	0.4330	0.4415	0.4382
B at a3	0.0000	87.3948	5	0.0000	***	0.3333	0.4859	0.4981	0.4952
B at a4	0.0000	80.7478	5	0.0000	***	0.3333	0.4958	0.5094	0.5062
B at a5	0.0000	129.9266	5	0.0000	***	0.3333	0.4073	0.4130	0.4104

LB = lower.bound, GG = Greenhouse-Geisser

HF = Huynh-Feldt-Lecoutre, CM = Chi-Muller

Source	SS	df	MS	F-ratio	p-value	G.eta^2

```
------------------------------------------------------------------
    A at b1  48.1947     4 12.0487   6.0163   0.0001 ***   0.0737
   Er at b1 430.5769   215  2.0027
------------------------------------------------------------------
    A at b2  27.7454     4  6.9363   7.5709   0.0000 ***   0.0822
   Er at b2 196.9807   215  0.9162
------------------------------------------------------------------
    A at b3  15.2733     4  3.8183   7.7139   0.0000 ***   0.0792
   Er at b3 106.4231   215  0.4950
------------------------------------------------------------------
    A at b4   7.8098     4  1.9525   5.9847   0.0001 ***   0.0595
   Er at b4  70.1415   215  0.3262
------------------------------------------------------------------
    B at a1  22.7147  1.38 16.4831  32.3928   0.0000 ***   0.1428
s x B at a1  30.8540 60.63  0.5089
------------------------------------------------------------------
    B at a2  32.4881   1.3 25.0114  33.4020   0.0000 ***   0.1737
s x B at a2  38.9056 51.96  0.7488
------------------------------------------------------------------
    B at a3  61.6312  1.46 42.2762  51.4098   0.0000 ***   0.2063
s x B at a3  53.9469  65.6  0.8223
------------------------------------------------------------------
    B at a4  24.5813  1.49 16.5278  20.5316   0.0000 ***   0.0907
s x B at a4  51.4813 63.95  0.8050
------------------------------------------------------------------
    B at a5  75.1991  1.22 61.5462  64.2845   0.0000 ***   0.1454
s x B at a5  50.3009 52.54  0.9574
------------------------------------------------------------------
                        +p < .10, *p < .05, **p < .01, ***p < .001
```

```
< MULTIPLE COMPARISON for "A at b1" >

== Holm's Sequentially Rejective Bonferroni Procedure ==
== The factor < A at b1 > is analysed as independent means. ==
== Alpha level is 0.05. ==

----------------------------------------------------------
  Pair     Diff  t-value  df       p   adj.p
----------------------------------------------------------
 a1-a5   0.5774   4.3032 215  0.0000  0.0003  a1 > a5 *
 a4-a5   0.4943   3.6635 215  0.0003  0.0028  a4 > a5 *
 a2-a5   0.4927   3.5866 215  0.0004  0.0033  a2 > a5 *
 a1-a3   0.2988   2.2517 215  0.0254  0.1775  a1 = a3
 a3-a5   0.2786   2.0876 215  0.0380  0.2281  a3 = a5
 a3-a4  -0.2157   1.6164 215  0.1075  0.5374  a3 = a4
 a2-a3   0.2141   1.5751 215  0.1167  0.5374  a2 = a3
 a1-a2   0.0847   0.6198 215  0.5360  1.0000  a1 = a2
 a1-a4   0.0831   0.6192 215  0.5365  1.0000  a1 = a4
 a2-a4  -0.0016   0.0117 215  0.9907  1.0000  a2 = a4
----------------------------------------------------------


< MULTIPLE COMPARISON for "A at b2" >
```

== Holm's Sequentially Rejective Bonferroni Procedure ==
== The factor < A at b2 > is analysed as independent means. ==
== Alpha level is 0.05. ==

Pair	Diff	t-value	df	p	adj.p	
a1-a5	0.4525	4.9862	215	0.0000	0.0000	a1 > a5 *
a2-a5	0.3966	4.2679	215	0.0000	0.0003	a2 > a5 *
a4-a5	0.3602	3.9471	215	0.0001	0.0009	a4 > a5 *
a3-a5	0.3060	3.3903	215	0.0008	0.0058	a3 > a5 *
a1-a3	0.1465	1.6323	215	0.1041	0.6245	a1 = a3
a1-a4	0.0923	1.0170	215	0.3103	1.0000	a1 = a4
a2-a3	0.0905	0.9847	215	0.3259	1.0000	a2 = a3
a1-a2	0.0560	0.6055	215	0.5455	1.0000	a1 = a2
a3-a4	-0.0542	0.6004	215	0.5488	1.0000	a3 = a4
a2-a4	0.0363	0.3911	215	0.6961	1.0000	a2 = a4

< MULTIPLE COMPARISON for "A at b3" >

== Holm's Sequentially Rejective Bonferroni Procedure ==
== The factor < A at b3 > is analysed as independent means. ==
== Alpha level is 0.05. ==

Pair	Diff	t-value	df	p	adj.p	
a1-a5	0.3181	4.7690	215	0.0000	0.0000	a1 > a5 *
a2-a5	0.3232	4.7326	215	0.0000	0.0000	a2 > a5 *
a3-a5	0.2496	3.7620	215	0.0002	0.0017	a3 > a5 *
a4-a5	0.2011	2.9984	215	0.0030	0.0212	a4 > a5 *
a2-a4	0.1221	1.7876	215	0.0752	0.4515	a2 = a4
a1-a4	0.1170	1.7538	215	0.0809	0.4515	a1 = a4
a2-a3	0.0736	1.0894	215	0.2772	1.0000	a2 = a3
a1-a3	0.0685	1.0387	215	0.3001	1.0000	a1 = a3
a3-a4	0.0485	0.7305	215	0.4659	1.0000	a3 = a4
a1-a2	-0.0051	0.0750	215	0.9403	1.0000	a1 = a2

< MULTIPLE COMPARISON for "A at b4" >

== Holm's Sequentially Rejective Bonferroni Procedure ==
== The factor < A at b4 > is analysed as independent means. ==
== Alpha level is 0.05. ==

Pair	Diff	t-value	df	p	adj.p	
a2-a5	0.2338	4.2164	215	0.0000	0.0004	a2 > a5 *
a1-a5	0.2216	4.0917	215	0.0001	0.0005	a1 > a5 *

a3-a5	0.1814	3.3672	215	0.0009	0.0072	a3 > a5 *
a4-a5	0.1830	3.3595	215	0.0009	0.0072	a4 > a5 *
a2-a3	0.0524	0.9554	215	0.3405	1.0000	a2 = a3
a2-a4	0.0508	0.9168	215	0.3603	1.0000	a2 = a4
a1-a3	0.0402	0.7509	215	0.4535	1.0000	a1 = a3
a1-a4	0.0386	0.7134	215	0.4764	1.0000	a1 = a4
a1-a2	-0.0122	0.2211	215	0.8252	1.0000	a1 = a2
a3-a4	-0.0016	0.0294	215	0.9766	1.0000	a3 = a4

< MULTIPLE COMPARISON for "B at a1" >

== Holm's Sequentially Rejective Bonferroni Procedure ==
== The factor < B at a1 > is analysed as dependent means. ==
== Alpha level is 0.05. ==

Pair	Diff	t-value	df	p	adj.p	
b2-b3	-0.1633	6.3694	44	0.0000	0.0000	b2 < b3 *
b1-b3	-0.3578	6.1830	44	0.0000	0.0000	b1 < b3 *
b1-b4	-0.4056	5.9917	44	0.0000	0.0000	b1 < b4 *
b2-b4	-0.2111	5.2152	44	0.0000	0.0000	b2 < b4 *
b1-b2	-0.1944	4.7949	44	0.0000	0.0000	b1 < b2 *
b3-b4	-0.0478	1.9573	44	0.0567	0.0567	b3 = b4

< MULTIPLE COMPARISON for "B at a2" >

== Holm's Sequentially Rejective Bonferroni Procedure ==
== The factor < B at a2 > is analysed as dependent means. ==
== Alpha level is 0.05. ==

Pair	Diff	t-value	df	p	adj.p	
b1-b4	-0.5024	6.2219	40	0.0000	0.0000	b1 < b4 *
b2-b4	-0.2793	6.0410	40	0.0000	0.0000	b2 < b4 *
b1-b3	-0.4476	5.8166	40	0.0000	0.0000	b1 < b3 *
b2-b3	-0.2244	5.5868	40	0.0000	0.0000	b2 < b3 *
b1-b2	-0.2232	4.7564	40	0.0000	0.0001	b1 < b2 *
b3-b4	-0.0549	2.2694	40	0.0287	0.0287	b3 < b4 *

< MULTIPLE COMPARISON for "B at a3" >

== Holm's Sequentially Rejective Bonferroni Procedure ==
== The factor < B at a3 > is analysed as dependent means. ==
== Alpha level is 0.05. ==

Pair	Diff	t-value	df	p	adj.p	
b2-b3	-0.2413	8.7577	45	0.0000	0.0000	b2 < b3 *
b1-b4	-0.6641	7.8521	45	0.0000	0.0000	b1 < b4 *
b1-b3	-0.5880	7.7521	45	0.0000	0.0000	b1 < b3 *
b2-b4	-0.3174	7.3395	45	0.0000	0.0000	b2 < b4 *
b1-b2	-0.3467	5.2119	45	0.0000	0.0000	b1 < b2 *
b3-b4	-0.0761	2.5564	45	0.0140	0.0140	b3 < b4 *

< MULTIPLE COMPARISON for "B at a4" >

== Holm's Sequentially Rejective Bonferroni Procedure ==
== The factor < B at a4 > is analysed as dependent means. ==
== Alpha level is 0.05. ==

Pair	Diff	t-value	df	p	adj.p	
b2-b4	-0.2648	5.7243	43	0.0000	0.0000	b2 < b4 *
b3-b4	-0.1261	5.4663	43	0.0000	0.0000	b3 < b4 *
b1-b4	-0.4500	5.4508	43	0.0000	0.0000	b1 < b4 *
b1-b3	-0.3239	3.9912	43	0.0003	0.0008	b1 < b3 *
b2-b3	-0.1386	3.3954	43	0.0015	0.0030	b2 < b3 *
b1-b2	-0.1852	2.9181	43	0.0056	0.0056	b1 < b2 *

< MULTIPLE COMPARISON for "B at a5" >

== Holm's Sequentially Rejective Bonferroni Procedure ==
== The factor < B at a5 > is analysed as dependent means. ==
== Alpha level is 0.05. ==

Pair	Diff	t-value	df	p	adj.p	
b1-b4	-0.7614	8.4106	43	0.0000	0.0000	b1 < b4 *
b1-b3	-0.6170	8.2361	43	0.0000	0.0000	b1 < b3 *
b2-b4	-0.4420	7.9161	43	0.0000	0.0000	b2 < b4 *
b2-b3	-0.2977	7.5275	43	0.0000	0.0000	b2 < b3 *
b1-b2	-0.3193	6.7846	43	0.0000	0.0000	b1 < b2 *
b3-b4	-0.1443	6.0798	43	0.0000	0.0000	b3 < b4 *

< MULTIPLE COMPARISON for "C" >

== Holm's Sequentially Rejective Bonferroni Procedure ==
== The factor < C > is analysed as dependent means. ==
== Alpha level is 0.05. ==

C	n	Mean	S.D.

c1	880	-0.0173	0.2221
c2	880	-0.1717	0.4139
c3	880	-0.3291	0.5801
c4	880	-0.4844	0.7149
c5	880	-0.7225	0.8806

Pair	Diff	t-value	df	p	adj.p	
c2-c5	0.5509	19.9155	215	0.0000	0.0000	c2 > c5 *
c1-c5	0.7053	18.6862	215	0.0000	0.0000	c1 > c5 *
c3-c5	0.3934	18.4609	215	0.0000	0.0000	c3 > c5 *
c1-c4	0.4672	15.5769	215	0.0000	0.0000	c1 > c4 *
c2-c4	0.3128	15.3209	215	0.0000	0.0000	c2 > c4 *
c1-c3	0.3119	13.7695	215	0.0000	0.0000	c1 > c3 *
c4-c5	0.2381	13.4987	215	0.0000	0.0000	c4 > c5 *
c2-c3	0.1575	11.5678	215	0.0000	0.0000	c2 > c3 *
c1-c2	0.1544	10.3563	215	0.0000	0.0000	c1 > c2 *
c3-c4	0.1553	10.3144	215	0.0000	0.0000	c3 > c4 *

< SIMPLE EFFECTS for "A x C" INTERACTION >

A	C	n	Mean	S.D.
a1	c1	180	-0.0111	0.1582
a1	c2	180	-0.1069	0.3353
a1	c3	180	-0.2125	0.4038
a1	c4	180	-0.3097	0.5037
a1	c5	180	-0.4403	0.6199
a2	c1	164	0.0137	0.2220
a2	c2	164	-0.1433	0.3324
a2	c3	164	-0.1997	0.4774
a2	c4	164	-0.3643	0.5935
a2	c5	164	-0.5412	0.6941
a3	c1	184	-0.0109	0.1586
a3	c2	184	-0.1522	0.3639
a3	c3	184	-0.3315	0.5539
a3	c4	184	-0.4796	0.7128
a3	c5	184	-0.7989	0.8926
a4	c1	176	0.0213	0.2433
a4	c2	176	-0.1250	0.3937
a4	c3	176	-0.2983	0.5443
a4	c4	176	-0.4233	0.6642
a4	c5	176	-0.6690	0.8077
a5	c1	176	-0.0994	0.2855
a5	c2	176	-0.3310	0.5621
a5	c3	176	-0.6037	0.7664
a5	c4	176	-0.8452	0.9094
a5	c5	176	-1.1634	1.1180

Effect	Lambda	approx.Chi	df	p		LB	GG	HF	CM
C at a1	0.0000	84.3064	9	0.0000	***	0.2500	0.4780	0.4992	0.4962
C at a2	0.0000	113.2983	9	0.0000	***	0.2500	0.3875	0.4002	0.3973
C at a3	0.0000	95.5785	9	0.0000	***	0.2500	0.4912	0.5134	0.5105
C at a4	0.0000	117.6883	9	0.0000	***	0.2500	0.3911	0.4033	0.4007
C at a5	0.0000	172.8961	9	0.0000	***	0.2500	0.3293	0.3356	0.3335

LB = lower.bound, GG = Greenhouse-Geisser

HF = Huynh-Feldt-Lecoutre, CM = Chi-Muller

Source	SS	df	MS	F-ratio	p-value		G.eta^2
A at c1	1.6218	4	0.4054	4.1364	0.0030	**	0.0383
Er at c1	21.0740	215	0.0980				
A at c2	5.8055	4	1.4514	4.5469	0.0015	**	0.0466
Er at c2	68.6281	215	0.3192				
A at c3	18.6307	4	4.6577	7.0625	0.0000	***	0.0749
Er at c3	141.7905	215	0.6595				
A at c4	31.4247	4	7.8562	7.4818	0.0000	***	0.0844
Er at c4	225.7583	215	1.0500				
A at c5	55.5099	4	13.8775	9.1495	0.0000	***	0.1026
Er at c5	326.1023	215	1.5168				
C at a1	20.3344	1.91	10.6343	35.0226	0.0000	***	0.1298
s x C at a1	25.5468	84.13	0.3036				
C at a2	29.7271	1.55	19.1776	45.1088	0.0000	***	0.1613
s x C at a2	26.3604	62	0.4251				
C at a3	68.5774	1.96	34.9015	70.5206	0.0000	***	0.2244
s x C at a3	43.7601	88.42	0.4949				
C at a4	50.2036	1.56	32.0878	52.7214	0.0000	***	0.1693
s x C at a4	40.9464	67.28	0.6086				

```
--------------------------------------------------------------------
    C at a5 123.2128  1.32 93.5430  77.7927   0.0000 ***   0.2180
s x C at a5  68.1060 56.64  1.2025
--------------------------------------------------------------------
                        +p < .10, *p < .05, **p < .01, ***p < .001
```

< MULTIPLE COMPARISON for "A at c1" >

== Holm's Sequentially Rejective Bonferroni Procedure ==
== The factor < A at c1 > is analysed as independent means. ==
== Alpha level is 0.05. ==

Pair	Diff	t-value	df	p	adj.p	
a4-a5	0.1207	3.6177	215	0.0004	0.0037	a4 > a5 *
a2-a5	0.1132	3.3300	215	0.0010	0.0092	a2 > a5 *
a3-a5	0.0886	2.6829	215	0.0079	0.0629	a3 = a5
a1-a5	0.0883	2.6612	215	0.0084	0.0629	a1 = a5
a1-a4	-0.0324	0.9768	215	0.3298	1.0000	a1 = a4
a3-a4	-0.0322	0.9748	215	0.3308	1.0000	a3 = a4
a1-a2	-0.0248	0.7347	215	0.4633	1.0000	a1 = a2
a2-a3	0.0246	0.7314	215	0.4654	1.0000	a2 = a3
a2-a4	-0.0076	0.2233	215	0.8235	1.0000	a2 = a4
a1-a3	-0.0002	0.0074	215	0.9941	1.0000	a1 = a3

< MULTIPLE COMPARISON for "A at c2" >

== Holm's Sequentially Rejective Bonferroni Procedure ==
== The factor < A at c2 > is analysed as independent means. ==
== Alpha level is 0.05. ==

Pair	Diff	t-value	df	p	adj.p	
a1-a5	0.2240	3.7405	215	0.0002	0.0024	a1 > a5 *
a4-a5	0.2060	3.4198	215	0.0007	0.0067	a4 > a5 *
a2-a5	0.1877	3.0606	215	0.0025	0.0199	a2 > a5 *
a3-a5	0.1788	3.0014	215	0.0030	0.0210	a3 > a5 *
a1-a3	0.0452	0.7636	215	0.4459	1.0000	a1 = a3
a1-a2	0.0363	0.5960	215	0.5518	1.0000	a1 = a2
a3-a4	-0.0272	0.4562	215	0.6487	1.0000	a3 = a4
a1-a4	0.0181	0.3015	215	0.7633	1.0000	a1 = a4
a2-a4	-0.0183	0.2983	215	0.7657	1.0000	a2 = a4
a2-a3	0.0089	0.1464	215	0.8838	1.0000	a2 = a3

< MULTIPLE COMPARISON for "A at c3" >

== Holm's Sequentially Rejective Bonferroni Procedure ==

== The factor < A at c3 > is analysed as independent means. ==
== Alpha level is 0.05. ==

Pair	Diff	t-value	df	p	adj.p	
a2-a5	0.4040	4.5837	215	0.0000	0.0001	a2 > a5 *
a1-a5	0.3912	4.5442	215	0.0000	0.0001	a1 > a5 *
a4-a5	0.3054	3.5278	215	0.0005	0.0041	a4 > a5 *
a3-a5	0.2722	3.1787	215	0.0017	0.0119	a3 > a5 *
a2-a3	0.1318	1.5116	215	0.1321	0.7926	a2 = a3
a1-a3	0.1190	1.3980	215	0.1635	0.8177	a1 = a3
a2-a4	0.0986	1.1187	215	0.2645	1.0000	a2 = a4
a1-a4	0.0858	0.9966	215	0.3201	1.0000	a1 = a4
a3-a4	-0.0332	0.3881	215	0.6984	1.0000	a3 = a4
a1-a2	-0.0128	0.1461	215	0.8840	1.0000	a1 = a2

< MULTIPLE COMPARISON for "A at c4" >

== Holm's Sequentially Rejective Bonferroni Procedure ==
== The factor < A at c4 > is analysed as independent means. ==
== Alpha level is 0.05. ==

Pair	Diff	t-value	df	p	adj.p	
a1-a5	0.5354	4.9293	215	0.0000	0.0000	a1 > a5 *
a2-a5	0.4808	4.3235	215	0.0000	0.0002	a2 > a5 *
a4-a5	0.4219	3.8621	215	0.0001	0.0012	a4 > a5 *
a3-a5	0.3656	3.3834	215	0.0009	0.0060	a3 > a5 *
a1-a3	0.1699	1.5815	215	0.1152	0.6914	a1 = a3
a2-a3	0.1153	1.0477	215	0.2960	1.0000	a2 = a3
a1-a4	0.1136	1.0455	215	0.2969	1.0000	a1 = a4
a2-a4	0.0590	0.5302	215	0.5965	1.0000	a2 = a4
a3-a4	-0.0563	0.5213	215	0.6027	1.0000	a3 = a4
a1-a2	0.0546	0.4937	215	0.6221	1.0000	a1 = a2

< MULTIPLE COMPARISON for "A at c5" >

== Holm's Sequentially Rejective Bonferroni Procedure ==
== The factor < A at c5 > is analysed as independent means. ==
== Alpha level is 0.05. ==

Pair	Diff	t-value	df	p	adj.p	
a1-a5	0.7231	5.5385	215	0.0000	0.0000	a1 > a5 *
a2-a5	0.6222	4.6549	215	0.0000	0.0001	a2 > a5 *
a4-a5	0.4943	3.7652	215	0.0002	0.0017	a4 > a5 *
a3-a5	0.3644	2.8066	215	0.0055	0.0383	a3 > a5 *

a1-a3	0.3586	2.7777	215	0.0060	0.0383	a1 > a3 *
a2-a3	0.2578	1.9489	215	0.0526	0.2630	a2 = a3
a1-a4	0.2288	1.7522	215	0.0812	0.3247	a1 = a4
a3-a4	-0.1299	1.0002	215	0.3183	0.9550	a3 = a4
a2-a4	0.1279	0.9567	215	0.3398	0.9550	a2 = a4
a1-a2	0.1009	0.7588	215	0.4488	0.9550	a1 = a2

< MULTIPLE COMPARISON for "C at a1" >

== Holm's Sequentially Rejective Bonferroni Procedure ==
== The factor < C at a1 > is analysed as dependent means. ==
== Alpha level is 0.05. ==

Pair	Diff	t-value	df	p	adj.p	
c2-c5	0.3333	7.0288	44	0.0000	0.0000	c2 > c5 *
c1-c5	0.4292	6.8439	44	0.0000	0.0000	c1 > c5 *
c1-c4	0.2986	6.2106	44	0.0000	0.0000	c1 > c4 *
c1-c3	0.2014	6.0601	44	0.0000	0.0000	c1 > c3 *
c3-c5	0.2278	5.7979	44	0.0000	0.0000	c3 > c5 *
c2-c4	0.2028	5.6875	44	0.0000	0.0000	c2 > c4 *
c2-c3	0.1056	4.3414	44	0.0001	0.0003	c2 > c3 *
c4-c5	0.1306	3.7832	44	0.0005	0.0014	c4 > c5 *
c1-c2	0.0958	3.5130	44	0.0010	0.0021	c1 > c2 *
c3-c4	0.0972	2.8185	44	0.0072	0.0072	c3 > c4 *

< MULTIPLE COMPARISON for "C at a2" >

== Holm's Sequentially Rejective Bonferroni Procedure ==
== The factor < C at a2 > is analysed as dependent means. ==
== Alpha level is 0.05. ==

Pair	Diff	t-value	df	p	adj.p	
c3-c5	0.3415	8.0565	40	0.0000	0.0000	c3 > c5 *
c1-c5	0.5549	7.6997	40	0.0000	0.0000	c1 > c5 *
c2-c5	0.3979	7.5904	40	0.0000	0.0000	c2 > c5 *
c1-c4	0.3780	6.2463	40	0.0000	0.0000	c1 > c4 *
c4-c5	0.1768	5.6189	40	0.0000	0.0000	c4 > c5 *
c1-c3	0.2134	5.4408	40	0.0000	0.0000	c1 > c3 *
c1-c2	0.1570	5.2449	40	0.0000	0.0000	c1 > c2 *
c2-c4	0.2210	5.2360	40	0.0000	0.0000	c2 > c4 *
c3-c4	0.1646	5.1234	40	0.0000	0.0000	c3 > c4 *
c2-c3	0.0564	2.6541	40	0.0114	0.0114	c2 > c3 *

< MULTIPLE COMPARISON for "C at a3" >

== Holm's Sequentially Rejective Bonferroni Procedure ==
== The factor < C at a3 > is analysed as dependent means. ==
== Alpha level is 0.05. ==

Pair	Diff	t-value	df	p	adj.p	
c2-c5	0.6467	11.2981	45	0.0000	0.0000	c2 > c5 *
c1-c5	0.7880	9.9307	45	0.0000	0.0000	c1 > c5 *
c3-c5	0.4674	9.3314	45	0.0000	0.0000	c3 > c5 *
c2-c4	0.3274	7.6520	45	0.0000	0.0000	c2 > c4 *
c1-c4	0.4688	7.1314	45	0.0000	0.0000	c1 > c4 *
c4-c5	0.3193	6.7253	45	0.0000	0.0000	c4 > c5 *
c1-c3	0.3207	6.1614	45	0.0000	0.0000	c1 > c3 *
c2-c3	0.1793	5.3070	45	0.0000	0.0000	c2 > c3 *
c3-c4	0.1481	4.8179	45	0.0000	0.0000	c3 > c4 *
c1-c2	0.1413	4.1824	45	0.0001	0.0001	c1 > c2 *

< MULTIPLE COMPARISON for "C at a4" >

== Holm's Sequentially Rejective Bonferroni Procedure ==
== The factor < C at a4 > is analysed as dependent means. ==
== Alpha level is 0.05. ==

Pair	Diff	t-value	df	p	adj.p	
c2-c5	0.5440	8.6973	43	0.0000	0.0000	c2 > c5 *
c1-c5	0.6903	8.0231	43	0.0000	0.0000	c1 > c5 *
c3-c5	0.3707	7.1157	43	0.0000	0.0000	c3 > c5 *
c2-c4	0.2983	6.8981	43	0.0000	0.0000	c2 > c4 *
c1-c3	0.3196	6.8176	43	0.0000	0.0000	c1 > c3 *
c1-c4	0.4446	6.7741	43	0.0000	0.0000	c1 > c4 *
c2-c3	0.1733	6.6218	43	0.0000	0.0000	c2 > c3 *
c4-c5	0.2457	6.1441	43	0.0000	0.0000	c4 > c5 *
c1-c2	0.1463	4.2728	43	0.0001	0.0002	c1 > c2 *
c3-c4	0.1250	3.6248	43	0.0008	0.0008	c3 > c4 *

< MULTIPLE COMPARISON for "C at a5" >

== Holm's Sequentially Rejective Bonferroni Procedure ==
== The factor < C at a5 > is analysed as dependent means. ==
== Alpha level is 0.05. ==

Pair	Diff	t-value	df	p	adj.p	
c3-c5	0.5597	10.7618	43	0.0000	0.0000	c3 > c5 *
c2-c5	0.8324	10.0456	43	0.0000	0.0000	c2 > c5 *

c1-c5	1.0639	9.4595	43	0.0000	0.0000	c1 > c5 *
c2-c4	0.5142	8.5067	43	0.0000	0.0000	c2 > c4 *
c1-c4	0.7457	8.4317	43	0.0000	0.0000	c1 > c4 *
c4-c5	0.3182	7.9614	43	0.0000	0.0000	c4 > c5 *
c1-c3	0.5043	7.0234	43	0.0000	0.0000	c1 > c3 *
c3-c4	0.2415	6.7106	43	0.0000	0.0000	c3 > c4 *
c2-c3	0.2727	6.6519	43	0.0000	0.0000	c2 > c3 *
c1-c2	0.2315	5.8406	43	0.0000	0.0000	c1 > c2 *

< SIMPLE EFFECTS for "B x C" INTERACTION >

B	C	n	Mean	S.D.
b1	c1	220	-0.0466	0.3211
b1	c2	220	-0.4273	0.5802
b1	c3	220	-0.6795	0.7724
b1	c4	220	-0.8852	0.9288
b1	c5	220	-1.2909	1.0709
b2	c1	220	-0.0216	0.2146
b2	c2	220	-0.1955	0.3865
b2	c3	220	-0.3727	0.5110
b2	c4	220	-0.6102	0.6741
b2	c5	220	-0.8557	0.8203
b3	c1	220	0.0000	0.1548
b3	c2	220	-0.0466	0.2468
b3	c3	220	-0.1784	0.4079
b3	c4	220	-0.2989	0.5081
b3	c5	220	-0.4670	0.6351
b4	c1	220	-0.0023	0.1530
b4	c2	220	-0.0170	0.1866
b4	c3	220	-0.0909	0.3482
b4	c4	220	-0.1466	0.3865
b4	c5	220	-0.2841	0.5369

Effect	Lambda	approx.Chi	df	p		LB	GG	HF	CM
B at c1	0.0000	293.2677	29	0.0000	***	0.3333	0.6697	0.6760	0.6759
B at c2	0.0000	314.2335	29	0.0000	***	0.3333	0.5796	0.5840	0.5838
B at c3	0.0000	241.8911	29	0.0000	***	0.3333	0.6243	0.6296	0.6294
B at c4	0.0000	226.5019	29	0.0000	***	0.3333	0.6381	0.6437	0.6436
B at c5	0.0000	262.8179	29	0.0000	***	0.3333	0.6465	0.6523	0.6521
C at b1	0.0000	325.9533	49	0.0000	***	0.2500	0.5988	0.6060	

```
0.6058
 C at b2  0.0000    357.6419  49 0.0000 *** 0.2500 0.5736 0.5801
0.5800
 C at b3  0.0000    433.2277  49 0.0000 *** 0.2500 0.5696 0.5761
0.5759
 C at b4  0.0000    449.7029  49 0.0000 *** 0.2500 0.6651 0.6742
0.6741
--------------------------------------------------------------------
------
                              LB = lower.bound, GG = Greenhouse-
Geisser
                             HF = Huynh-Feldt-Lecoutre, CM = Chi-
Muller

--------------------------------------------------------------------
-
     Source        SS     df       MS  F-ratio  p-value      G.eta^2
--------------------------------------------------------------------
-
    B at c1   0.3008   2.01  0.1497   3.2835   0.0382 *     0.0073
s x B at c1  19.6976 431.97  0.0456
--------------------------------------------------------------------
-
    B at c2  23.2320   1.74 13.3599  99.5929   0.0000 ***   0.1636
s x B at c2  50.1530 373.87  0.1341
--------------------------------------------------------------------
-
    B at c3  44.7761   1.87 23.9084 108.8523   0.0000 ***   0.1628
s x B at c3  88.4396 402.66  0.2196
--------------------------------------------------------------------
-
    B at c4  71.2120   1.91 37.1984 132.7361   0.0000 ***   0.1727
s x B at c4 115.3461 411.59  0.2802
--------------------------------------------------------------------
-
    B at c5 130.6356   1.94 67.3548 176.3333   0.0000 ***   0.2121
s x B at c5 159.2816    417  0.3820
--------------------------------------------------------------------
-
    C at b1 192.1547    2.4 80.2311 236.2301   0.0000 ***   0.2409
s x C at b1 174.8857 514.93  0.3396
--------------------------------------------------------------------
-
    C at b2  95.5524   2.29 41.6482 182.1085   0.0000 ***   0.2357
s x C at b2 112.8105 493.27  0.2287
--------------------------------------------------------------------
-
    C at b3  31.8865   2.28 13.9943  96.4349   0.0000 ***   0.1523
s x C at b3  71.0904 489.88  0.1451
--------------------------------------------------------------------
-
    C at b4  11.3737   2.66  4.2751  45.8255   0.0000 ***   0.0843
s x C at b4  53.3622    572  0.0933
--------------------------------------------------------------------
```

-

+p < .10, *p < .05, **p < .01, ***p < .001

< MULTIPLE COMPARISON for "B at c1" >

== Holm's Sequentially Rejective Bonferroni Procedure ==
== The factor < B at c1 > is analysed as dependent means. ==
== Alpha level is 0.05. ==

Pair	Diff	t-value	df	p	adj.p	
b1-b3	-0.0462	2.2229	215	0.0273	0.1636	b1 = b3
b1-b4	-0.0434	2.0789	215	0.0388	0.1941	b1 = b4
b2-b3	-0.0226	1.6722	215	0.0959	0.3838	b2 = b3
b2-b4	-0.0198	1.5020	215	0.1346	0.4037	b2 = b4
b1-b2	-0.0236	1.2664	215	0.2067	0.4135	b1 = b2
b3-b4	0.0028	0.2836	215	0.7770	0.7770	b3 = b4

< MULTIPLE COMPARISON for "B at c2" >

== Holm's Sequentially Rejective Bonferroni Procedure ==
== The factor < B at c2 > is analysed as dependent means. ==
== Alpha level is 0.05. ==

Pair	Diff	t-value	df	p	adj.p	
b1-b4	-0.4106	11.6207	215	0.0000	0.0000	b1 < b4 *
b1-b3	-0.3815	11.3140	215	0.0000	0.0000	b1 < b3 *
b2-b3	-0.1490	8.2968	215	0.0000	0.0000	b2 < b3 *
b2-b4	-0.1781	8.2108	215	0.0000	0.0000	b2 < b4 *
b1-b2	-0.2325	7.7794	215	0.0000	0.0000	b1 < b2 *
b3-b4	-0.0291	2.2004	215	0.0288	0.0288	b3 < b4 *

< MULTIPLE COMPARISON for "B at c3" >

== Holm's Sequentially Rejective Bonferroni Procedure ==
== The factor < B at c3 > is analysed as dependent means. ==
== Alpha level is 0.05. ==

Pair	Diff	t-value	df	p	adj.p	
b1-b4	-0.5876	12.5319	215	0.0000	0.0000	b1 < b4 *
b1-b3	-0.5015	11.3546	215	0.0000	0.0000	b1 < b3 *
b2-b4	-0.2815	9.4387	215	0.0000	0.0000	b2 < b4 *
b1-b2	-0.3061	8.5004	215	0.0000	0.0000	b1 < b2 *
b2-b3	-0.1955	7.6115	215	0.0000	0.0000	b2 < b3 *

```
 b3-b4  -0.0860   3.8662 215  0.0001  0.0001  b3 < b4 *
----------------------------------------------------------


< MULTIPLE COMPARISON for "B at c4" >

== Holm's Sequentially Rejective Bonferroni Procedure ==
== The factor < B at c4 > is analysed as dependent means. ==
== Alpha level is 0.05. ==

----------------------------------------------------------
  Pair     Diff  t-value  df       p   adj.p
----------------------------------------------------------
 b1-b4  -0.7378  13.7046 215  0.0000  0.0000  b1 < b4 *
 b2-b4  -0.4645  13.1920 215  0.0000  0.0000  b2 < b4 *
 b1-b3  -0.5844  12.0424 215  0.0000  0.0000  b1 < b3 *
 b2-b3  -0.3111  10.4774 215  0.0000  0.0000  b2 < b3 *
 b1-b2  -0.2734   6.4480 215  0.0000  0.0000  b1 < b2 *
 b3-b4  -0.1534   6.2799 215  0.0000  0.0000  b3 < b4 *
----------------------------------------------------------


< MULTIPLE COMPARISON for "B at c5" >

== Holm's Sequentially Rejective Bonferroni Procedure ==
== The factor < B at c5 > is analysed as dependent means. ==
== Alpha level is 0.05. ==

----------------------------------------------------------
  Pair     Diff  t-value  df       p   adj.p
----------------------------------------------------------
 b1-b4  -1.0041  16.0627 215  0.0000  0.0000  b1 < b4 *
 b1-b3  -0.8206  14.4569 215  0.0000  0.0000  b1 < b3 *
 b2-b4  -0.5707  12.7956 215  0.0000  0.0000  b2 < b4 *
 b2-b3  -0.3873  10.6975 215  0.0000  0.0000  b2 < b3 *
 b1-b2  -0.4333   9.1149 215  0.0000  0.0000  b1 < b2 *
 b3-b4  -0.1834   6.4854 215  0.0000  0.0000  b3 < b4 *
----------------------------------------------------------


< MULTIPLE COMPARISON for "C at b1" >

== Holm's Sequentially Rejective Bonferroni Procedure ==
== The factor < C at b1 > is analysed as dependent means. ==
== Alpha level is 0.05. ==

---------------------------------------------------------
  Pair    Diff  t-value  df       p   adj.p
---------------------------------------------------------
 c1-c5  1.2415  20.0876 215  0.0000  0.0000  c1 > c5 *
 c2-c5  0.8592  18.1716 215  0.0000  0.0000  c2 > c5 *
 c3-c5  0.6091  17.1506 215  0.0000  0.0000  c3 > c5 *
 c1-c4  0.8378  15.4408 215  0.0000  0.0000  c1 > c4 *
 c1-c3  0.6324  14.7801 215  0.0000  0.0000  c1 > c3 *
```

Pair	Diff	t-value	df	p	adj.p	
c1-c2	0.3823	11.7566	215	0.0000	0.0000	c1 > c2 *
c2-c4	0.4555	11.5398	215	0.0000	0.0000	c2 > c4 *
c4-c5	0.4037	9.8876	215	0.0000	0.0000	c4 > c5 *
c2-c3	0.2501	8.3961	215	0.0000	0.0000	c2 > c3 *
c3-c4	0.2054	5.8015	215	0.0000	0.0000	c3 > c4 *

< MULTIPLE COMPARISON for "C at b2" >

== Holm's Sequentially Rejective Bonferroni Procedure ==
== The factor < C at b2 > is analysed as dependent means. ==
== Alpha level is 0.05. ==

Pair	Diff	t-value	df	p	adj.p	
c2-c5	0.6584	16.4073	215	0.0000	0.0000	c2 > c5 *
c1-c5	0.8318	16.2390	215	0.0000	0.0000	c1 > c5 *
c1-c4	0.5880	14.7609	215	0.0000	0.0000	c1 > c4 *
c2-c4	0.4146	13.7962	215	0.0000	0.0000	c2 > c4 *
c3-c5	0.4818	13.5236	215	0.0000	0.0000	c3 > c5 *
c1-c3	0.3499	11.3278	215	0.0000	0.0000	c1 > c3 *
c3-c4	0.2381	8.1021	215	0.0000	0.0000	c3 > c4 *
c2-c3	0.1765	7.6715	215	0.0000	0.0000	c2 > c3 *
c4-c5	0.2438	7.4918	215	0.0000	0.0000	c4 > c5 *
c1-c2	0.1734	7.4353	215	0.0000	0.0000	c1 > c2 *

< MULTIPLE COMPARISON for "C at b3" >

== Holm's Sequentially Rejective Bonferroni Procedure ==
== The factor < C at b3 > is analysed as dependent means. ==
== Alpha level is 0.05. ==

Pair	Diff	t-value	df	p	adj.p	
c2-c5	0.4201	12.4831	215	0.0000	0.0000	c2 > c5 *
c1-c5	0.4670	11.9294	215	0.0000	0.0000	c1 > c5 *
c2-c4	0.2526	10.4034	215	0.0000	0.0000	c2 > c4 *
c1-c4	0.2995	9.8879	215	0.0000	0.0000	c1 > c4 *
c3-c5	0.2900	9.6654	215	0.0000	0.0000	c3 > c5 *
c4-c5	0.1675	7.1571	215	0.0000	0.0000	c4 > c5 *
c1-c3	0.1770	7.0394	215	0.0000	0.0000	c1 > c3 *
c2-c3	0.1301	6.2944	215	0.0000	0.0000	c2 > c3 *
c3-c4	0.1225	5.0479	215	0.0000	0.0000	c3 > c4 *
c1-c2	0.0469	2.9282	215	0.0038	0.0038	c1 > c2 *

< MULTIPLE COMPARISON for "C at b4" >

== Holm's Sequentially Rejective Bonferroni Procedure ==
== The factor < C at b4 > is analysed as dependent means. ==
== Alpha level is 0.05. ==

Pair	Diff	t-value	df	p	adj.p	
c1-c5	0.2808	8.9801	215	0.0000	0.0000	c1 > c5 *
c2-c5	0.2658	8.8318	215	0.0000	0.0000	c2 > c5 *
c3-c5	0.1926	7.2054	215	0.0000	0.0000	c3 > c5 *
c1-c4	0.1433	6.2427	215	0.0000	0.0000	c1 > c4 *
c2-c4	0.1283	6.1465	215	0.0000	0.0000	c2 > c4 *
c4-c5	0.1375	4.8567	215	0.0000	0.0000	c4 > c5 *
c1-c3	0.0882	4.4092	215	0.0000	0.0001	c1 > c3 *
c2-c3	0.0732	4.0493	215	0.0001	0.0002	c2 > c3 *
c3-c4	0.0551	2.7174	215	0.0071	0.0142	c3 > c4 *
c1-c2	0.0150	1.2274	215	0.2210	0.2210	c1 = c2

< HIGHER-ORDER "A x B x C" INTERACTION >
*** Split the dataset for further analysis. ***

[[Simple Effects for a1]]

[sAB-Type Design]

This output was generated by anovakun 4.8.5 under R version 4.0.2.
It was executed on Mon Oct 11 18:22:23 2021.

<< DESCRIPTIVE STATISTICS >>

B	C	n	Mean	S.D.
b1	c1	45	-0.0222	0.2601
b1	c2	45	-0.3000	0.5503
b1	c3	45	-0.4778	0.5509
b1	c4	45	-0.5778	0.7108
b1	c5	45	-0.9000	0.7984
b2	c1	45	-0.0111	0.1406
b2	c2	45	-0.0944	0.2516
b2	c3	45	-0.2722	0.4018
b2	c4	45	-0.4000	0.4689
b2	c5	45	-0.5278	0.5671
b3	c1	45	-0.0111	0.1187
b3	c2	45	-0.0333	0.1735
b3	c3	45	-0.0722	0.2038
b3	c4	45	-0.1444	0.2743
b3	c5	45	-0.2278	0.3946
b4	c1	45	0.0000	0.0000
b4	c2	45	0.0000	0.0754

b4	c3	45	-0.0278	0.1619
b4	c4	45	-0.1167	0.2900
b4	c5	45	-0.1056	0.2528

<< SPHERICITY INDICES >>

== Mendoza's Multisample Sphericity Test and Epsilons ==

Effect	Lambda	approx.Chi	df	p		LB	GG	HF	CM
Global	0.0000	896.5969	189	0.0000	***	0.0526	0.1850	0.2030	0.2018
B	0.0000	99.6523	5	0.0000	***	0.3333	0.4594	0.4693	0.4665
C	0.0000	84.3064	9	0.0000	***	0.2500	0.4780	0.4992	0.4962
B x C	0.0000	233.9413	77	0.0000	***	0.0833	0.5507	0.6583	0.6543

LB = lower.bound, GG = Greenhouse-Geisser
HF = Huynh-Feldt-Lecoutre, CM = Chi-Muller

<< ANOVA TABLE >>

== Adjusted by Greenhouse-Geisser's Epsilon ==

Source	SS	df	MS	F-ratio	p-value		G.eta^2
s	49.7726	44	1.1312				
B	22.7147	1.38	16.4831	32.3928	0.0000	***	0.1428
s x B	30.8540	60.63	0.5089				
C	20.3344	1.91	10.6343	35.0226	0.0000	***	0.1298
s x C	25.5468	84.13	0.3036				
B x C	8.9228	6.61	1.3503	13.0018	0.0000	***	0.0614
s x B x C	30.1960	290.75	0.1039				
Total	188.3414	899	0.2095				

+p < .10, *p < .05, **p < .01, ***p < .001

<< POST ANALYSES >>

< MULTIPLE COMPARISON for "B" >

== Holm's Sequentially Rejective Bonferroni Procedure ==
== The factor < B > is analysed as dependent means. ==
== Alpha level is 0.05. ==

B	n	Mean	S.D.
b1	225	-0.4556	0.6650
b2	225	-0.2611	0.4367
b3	225	-0.0978	0.2618
b4	225	-0.0500	0.1948

Pair	Diff	t-value	df	p	adj.p	
b2-b3	-0.1633	6.3694	44	0.0000	0.0000	b2 < b3 *
b1-b3	-0.3578	6.1830	44	0.0000	0.0000	b1 < b3 *
b1-b4	-0.4056	5.9917	44	0.0000	0.0000	b1 < b4 *
b2-b4	-0.2111	5.2152	44	0.0000	0.0000	b2 < b4 *
b1-b2	-0.1944	4.7949	44	0.0000	0.0000	b1 < b2 *
b3-b4	-0.0478	1.9573	44	0.0567	0.0567	b3 = b4

< MULTIPLE COMPARISON for "C" >

== Holm's Sequentially Rejective Bonferroni Procedure ==
== The factor < C > is analysed as dependent means. ==
== Alpha level is 0.05. ==

C	n	Mean	S.D.
c1	180	-0.0111	0.1582
c2	180	-0.1069	0.3353
c3	180	-0.2125	0.4038
c4	180	-0.3097	0.5037
c5	180	-0.4403	0.6199

Pair	Diff	t-value	df	p	adj.p	
c2-c5	0.3333	7.0288	44	0.0000	0.0000	c2 > c5 *
c1-c5	0.4292	6.8439	44	0.0000	0.0000	c1 > c5 *
c1-c4	0.2986	6.2106	44	0.0000	0.0000	c1 > c4 *
c1-c3	0.2014	6.0601	44	0.0000	0.0000	c1 > c3 *
c3-c5	0.2278	5.7979	44	0.0000	0.0000	c3 > c5 *
c2-c4	0.2028	5.6875	44	0.0000	0.0000	c2 > c4 *

c2-c3	0.1056	4.3414	44	0.0001	0.0003	c2 > c3 *
c4-c5	0.1306	3.7832	44	0.0005	0.0014	c4 > c5 *
c1-c2	0.0958	3.5130	44	0.0010	0.0021	c1 > c2 *
c3-c4	0.0972	2.8185	44	0.0072	0.0072	c3 > c4 *

< SIMPLE EFFECTS for "B x C" INTERACTION >

Effect	Lambda	approx.Chi	df	p		LB	GG	HF	CM
B at c1	0.0000	55.2157	5	0.0000	***	0.3333	0.5477	0.5661	0.5627
B at c2	0.0000	111.8155	5	0.0000	***	0.3333	0.4417	0.4502	0.4474
B at c3	0.0000	48.8540	5	0.0000	***	0.3333	0.6376	0.6659	0.6619
B at c4	0.0000	30.4731	5	0.0000	***	0.3333	0.6807	0.7142	0.7098
B at c5	0.0000	45.1779	5	0.0000	***	0.3333	0.5842	0.6065	0.6029
C at b1	0.0000	38.8624	9	0.0000	***	0.2500	0.6910	0.7419	0.7374
C at b2	0.0000	61.5209	9	0.0000	***	0.2500	0.5984	0.6352	0.6313
C at b3	0.0000	49.2256	9	0.0000	***	0.2500	0.5887	0.6241	0.6203
C at b4	0.0000	93.6185	9	0.0000	***	0.2500	0.6169	0.6564	0.6524

LB = lower.bound, GG = Greenhouse-Geisser
HF = Huynh-Feldt-Lecoutre, CM = Chi-Muller

Source	SS	df	MS	F-ratio	p-value		G.eta^2
B at c1	0.0111	1.64	0.0068	0.1870	0.7868	ns	0.0025
s x B at c1	2.6139	72.3	0.0362				
B at c2	2.4427	1.33	1.8433	11.1185	0.0005	***	0.1214
s x B at c2	9.6667	58.31	0.1658				
B at c3	5.7483	1.91	3.0051	20.9376	0.0000	***	0.1970
s x B at c3	12.0799	84.17	0.1435				
B at c4	6.5066	2.04	3.1862	15.1856	0.0000	***	0.1433
s x B at c4	18.8528	89.85	0.2098				

B at c5	16.9288	1.75	9.6585	41.7602	0.0000	***	0.2461
s x B at c5	17.8368	77.12	0.2313				
C at b1	19.1222	2.76	6.9186	31.0440	0.0000	***	0.1930
s x C at b1	27.1028	121.61	0.2229				
C at b2	8.1361	2.39	3.3991	23.1500	0.0000	***	0.1904
s x C at b2	15.4639	105.32	0.1468				
C at b3	1.4128	2.35	0.6000	8.6792	0.0001	***	0.0920
s x C at b3	7.1622	103.61	0.0691				
C at b4	0.5861	2.47	0.2375	4.2882	0.0106	*	0.0690
s x C at b4	6.0139	108.57	0.0554				

+p < .10, *p < .05, **p < .01, ***p < .001

< MULTIPLE COMPARISON for "B at c2" >

== Holm's Sequentially Rejective Bonferroni Procedure ==
== The factor < B at c2 > is analysed as dependent means. ==
== Alpha level is 0.05. ==

Pair	Diff	t-value	df	p	adj.p	
b1-b4	-0.3000	3.6399	44	0.0007	0.0043	b1 < b4 *
b1-b3	-0.2667	3.5696	44	0.0009	0.0044	b1 < b3 *
b1-b2	-0.2056	2.9619	44	0.0049	0.0197	b1 < b2 *
b2-b3	-0.0611	2.6932	44	0.0100	0.0299	b2 < b3 *
b2-b4	-0.0944	2.6389	44	0.0115	0.0299	b2 < b4 *
b3-b4	-0.0333	1.4306	44	0.1596	0.1596	b3 = b4

< MULTIPLE COMPARISON for "B at c3" >

== Holm's Sequentially Rejective Bonferroni Procedure ==
== The factor < B at c3 > is analysed as dependent means. ==
== Alpha level is 0.05. ==

Pair	Diff	t-value	df	p	adj.p	
b1-b4	-0.4500	5.4347	44	0.0000	0.0000	b1 < b4 *
b1-b3	-0.4056	5.2846	44	0.0000	0.0000	b1 < b3 *
b2-b3	-0.2000	4.3425	44	0.0001	0.0003	b2 < b3 *
b2-b4	-0.2444	3.7449	44	0.0005	0.0016	b2 < b4 *
b1-b2	-0.2056	3.3319	44	0.0018	0.0035	b1 < b2 *
b3-b4	-0.0444	1.1591	44	0.2527	0.2527	b3 = b4

< MULTIPLE COMPARISON for "B at c4" >

== Holm's Sequentially Rejective Bonferroni Procedure ==
== The factor < B at c4 > is analysed as dependent means. ==
== Alpha level is 0.05. ==

Pair	Diff	t-value	df	p	adj.p	
b1-b3	-0.4333	4.6192	44	0.0000	0.0002	b1 < b3 *
b1-b4	-0.4611	4.4001	44	0.0001	0.0003	b1 < b4 *
b2-b3	-0.2556	4.3572	44	0.0001	0.0003	b2 < b3 *
b2-b4	-0.2833	3.7057	44	0.0006	0.0018	b2 < b4 *
b1-b2	-0.1778	2.3249	44	0.0248	0.0495	b1 < b2 *
b3-b4	-0.0278	0.4933	44	0.6242	0.6242	b3 = b4

< MULTIPLE COMPARISON for "B at c5" >

== Holm's Sequentially Rejective Bonferroni Procedure ==
== The factor < B at c5 > is analysed as dependent means. ==
== Alpha level is 0.05. ==

Pair	Diff	t-value	df	p	adj.p	
b1-b4	-0.7944	7.4466	44	0.0000	0.0000	b1 < b4 *
b1-b3	-0.6722	7.1806	44	0.0000	0.0000	b1 < b3 *
b2-b4	-0.4222	5.7698	44	0.0000	0.0000	b2 < b4 *
b1-b2	-0.3722	5.4598	44	0.0000	0.0000	b1 < b2 *
b2-b3	-0.3000	5.0636	44	0.0000	0.0000	b2 < b3 *
b3-b4	-0.1222	2.5079	44	0.0159	0.0159	b3 < b4 *

< MULTIPLE COMPARISON for "C at b1" >

== Holm's Sequentially Rejective Bonferroni Procedure ==
== The factor < C at b1 > is analysed as dependent means. ==
== Alpha level is 0.05. ==

Pair	Diff	t-value	df	p	adj.p	
c1-c5	0.8778	8.0016	44	0.0000	0.0000	c1 > c5 *
c2-c5	0.6000	7.2529	44	0.0000	0.0000	c2 > c5 *
c1-c3	0.4556	6.5217	44	0.0000	0.0000	c1 > c3 *
c3-c5	0.4222	6.0237	44	0.0000	0.0000	c3 > c5 *
c1-c4	0.5556	5.1844	44	0.0000	0.0000	c1 > c4 *
c4-c5	0.3222	3.9839	44	0.0003	0.0013	c4 > c5 *
c1-c2	0.2778	3.7650	44	0.0005	0.0020	c1 > c2 *
c2-c3	0.1778	3.3877	44	0.0015	0.0045	c2 > c3 *

c2-c4	0.2778	3.2856	44	0.0020	0.0045	c2 > c4 *
c3-c4	0.1000	1.2499	44	0.2180	0.2180	c3 = c4

< MULTIPLE COMPARISON for "C at b2" >

== Holm's Sequentially Rejective Bonferroni Procedure ==
== The factor < C at b2 > is analysed as dependent means. ==
== Alpha level is 0.05. ==

Pair	Diff	t-value	df	p	adj.p	
c1-c5	0.5167	6.0014	44	0.0000	0.0000	c1 > c5 *
c2-c5	0.4333	5.9720	44	0.0000	0.0000	c2 > c5 *
c1-c4	0.3889	5.5939	44	0.0000	0.0000	c1 > c4 *
c2-c4	0.3056	5.3386	44	0.0000	0.0000	c2 > c4 *
c1-c3	0.2611	4.6968	44	0.0000	0.0002	c1 > c3 *
c3-c5	0.2556	3.7019	44	0.0006	0.0030	c3 > c5 *
c2-c3	0.1778	3.4272	44	0.0013	0.0053	c2 > c3 *
c1-c2	0.0833	2.6220	44	0.0120	0.0359	c1 > c2 *
c4-c5	0.1278	2.3809	44	0.0217	0.0433	c4 > c5 *
c3-c4	0.1278	2.0655	44	0.0448	0.0448	c3 > c4 *

< MULTIPLE COMPARISON for "C at b3" >

== Holm's Sequentially Rejective Bonferroni Procedure ==
== The factor < C at b3 > is analysed as dependent means. ==
== Alpha level is 0.05. ==

Pair	Diff	t-value	df	p	adj.p	
c3-c5	0.1556	3.6175	44	0.0008	0.0076	c3 > c5 *
c2-c5	0.1944	3.5364	44	0.0010	0.0087	c2 > c5 *
c1-c5	0.2167	3.4744	44	0.0012	0.0093	c1 > c5 *
c2-c4	0.1111	3.1623	44	0.0028	0.0198	c2 > c4 *
c1-c4	0.1333	3.0336	44	0.0040	0.0243	c1 > c4 *
c3-c4	0.0722	2.2295	44	0.0309	0.1547	c3 = c4
c4-c5	0.0833	1.9149	44	0.0620	0.2481	c4 = c5
c1-c3	0.0611	1.7132	44	0.0937	0.2812	c1 = c3
c2-c3	0.0389	1.2656	44	0.2123	0.4246	c2 = c3
c1-c2	0.0222	0.7031	44	0.4857	0.4857	c1 = c2

< MULTIPLE COMPARISON for "C at b4" >

== Holm's Sequentially Rejective Bonferroni Procedure ==
== The factor < C at b4 > is analysed as dependent means. ==
== Alpha level is 0.05. ==

Pair	Diff	t-value	df	p	adj.p	
c1-c5	0.1056	2.8014	44	0.0075	0.0753	c1 = c5
c2-c5	0.1056	2.7411	44	0.0088	0.0793	c2 = c5
c1-c4	0.1167	2.6988	44	0.0098	0.0793	c1 = c4
c2-c4	0.1167	2.6120	44	0.0123	0.0859	c2 = c4
c3-c5	0.0778	1.9264	44	0.0605	0.3632	c3 = c5
c3-c4	0.0889	1.8353	44	0.0732	0.3661	c3 = c4
c1-c3	0.0278	1.1512	44	0.2559	1.0000	c1 = c3
c2-c3	0.0278	1.0935	44	0.2801	1.0000	c2 = c3
c4-c5	-0.0111	0.1999	44	0.8425	1.0000	c4 = c5
c1-c2	0.0000	0.0000	44	1.0000	1.0000	c1 = c2

output is over --------------------///

[[Simple Effects for a2]]

[sAB-Type Design]

This output was generated by anovakun 4.8.5 under R version 4.0.2.
It was executed on Mon Oct 11 18:22:24 2021.

<< DESCRIPTIVE STATISTICS >>

B	C	n	Mean	S.D.
b1	c1	41	0.0244	0.3298
b1	c2	41	-0.4207	0.4791
b1	c3	41	-0.5305	0.6782
b1	c4	41	-0.7134	0.7171
b1	c5	41	-1.0610	0.8344
b2	c1	41	-0.0305	0.1695
b2	c2	41	-0.1280	0.2569
b2	c3	41	-0.2378	0.3160
b2	c4	41	-0.5305	0.6713
b2	c5	41	-0.6585	0.6040
b3	c1	41	0.0488	0.2033
b3	c2	41	-0.0061	0.1044
b3	c3	41	-0.0183	0.2704
b3	c4	41	-0.1890	0.3344
b3	c5	41	-0.2988	0.4944
b4	c1	41	0.0122	0.1364
b4	c2	41	-0.0183	0.1713
b4	c3	41	-0.0122	0.3352
b4	c4	41	-0.0244	0.2359
b4	c5	41	-0.1463	0.3831

```
<< SPHERICITY INDICES >>

== Mendoza's Multisample Sphericity Test and Epsilons ==

--------------------------------------------------------------------
-----
 Effect  Lambda  approx.Chi  df      p         LB     GG     HF
CM
--------------------------------------------------------------------
-----
 Global  0.0000    951.6359 189 0.0000 *** 0.0526 0.1562 0.1701
0.1689
      B  0.0000     93.3090   5 0.0000 *** 0.3333 0.4330 0.4415
0.4382
      C  0.0000    113.2983   9 0.0000 *** 0.2500 0.3875 0.4002
0.3973
  B x C  0.0000    229.1032  77 0.0000 *** 0.0833 0.4780 0.5671
0.5629
--------------------------------------------------------------------
-----
                              LB = lower.bound, GG = Greenhouse-
Geisser
                             HF = Huynh-Feldt-Lecoutre, CM = Chi-
Muller


<< ANOVA TABLE >>

== Adjusted by Greenhouse-Geisser's Epsilon ==

-------------------------------------------------------------------
   Source       SS     df      MS  F-ratio  p-value      G.eta^2
-------------------------------------------------------------------
        s  55.2486     40  1.3812
-------------------------------------------------------------------
        B  32.4881    1.3 25.0114  33.4020   0.0000 ***   0.1737
    s x B  38.9056  51.96  0.7488
-------------------------------------------------------------------
        C  29.7271   1.55 19.1776  45.1088   0.0000 ***   0.1613
    s x C  26.3604     62  0.4251
-------------------------------------------------------------------
    B x C  12.1308   5.74  2.1149  14.2687   0.0000 ***   0.0728
s x B x C  34.0067 229.43  0.1482
-------------------------------------------------------------------
    Total 228.8674    819  0.2794
                       +p < .10, *p < .05, **p < .01, ***p < .001


<< POST ANALYSES >>

< MULTIPLE COMPARISON for "B" >

== Holm's Sequentially Rejective Bonferroni Procedure ==
```

== The factor < B > is analysed as dependent means. ==
== Alpha level is 0.05. ==

B	n	Mean	S.D.
b1	205	-0.5402	0.7220
b2	205	-0.3171	0.5056
b3	205	-0.0927	0.3339
b4	205	-0.0378	0.2725

Pair	Diff	t-value	df	p	adj.p	
b1-b4	-0.5024	6.2219	40	0.0000	0.0000	b1 < b4 *
b2-b4	-0.2793	6.0410	40	0.0000	0.0000	b2 < b4 *
b1-b3	-0.4476	5.8166	40	0.0000	0.0000	b1 < b3 *
b2-b3	-0.2244	5.5868	40	0.0000	0.0000	b2 < b3 *
b1-b2	-0.2232	4.7564	40	0.0000	0.0001	b1 < b2 *
b3-b4	-0.0549	2.2694	40	0.0287	0.0287	b3 < b4 *

< MULTIPLE COMPARISON for "C" >

== Holm's Sequentially Rejective Bonferroni Procedure ==
== The factor < C > is analysed as dependent means. ==
== Alpha level is 0.05. ==

C	n	Mean	S.D.
c1	164	0.0137	0.2220
c2	164	-0.1433	0.3324
c3	164	-0.1997	0.4774
c4	164	-0.3643	0.5935
c5	164	-0.5412	0.6941

Pair	Diff	t-value	df	p	adj.p	
c3-c5	0.3415	8.0565	40	0.0000	0.0000	c3 > c5 *
c1-c5	0.5549	7.6997	40	0.0000	0.0000	c1 > c5 *
c2-c5	0.3979	7.5904	40	0.0000	0.0000	c2 > c5 *
c1-c4	0.3780	6.2463	40	0.0000	0.0000	c1 > c4 *
c4-c5	0.1768	5.6189	40	0.0000	0.0000	c4 > c5 *
c1-c3	0.2134	5.4408	40	0.0000	0.0000	c1 > c3 *
c1-c2	0.1570	5.2449	40	0.0000	0.0000	c1 > c2 *
c2-c4	0.2210	5.2360	40	0.0000	0.0000	c2 > c4 *
c3-c4	0.1646	5.1234	40	0.0000	0.0000	c3 > c4 *
c2-c3	0.0564	2.6541	40	0.0114	0.0114	c2 > c3 *

< SIMPLE EFFECTS for "B x C" INTERACTION >

Effect	Lambda	approx.Chi	df	p		LB	GG	HF	CM
B at c1	0.0000	55.0422	5	0.0000	***	0.3333	0.5598	0.5816	0.5773
B at c2	0.0000	61.2685	5	0.0000	***	0.3333	0.5161	0.5329	0.5290
B at c3	0.0000	38.6279	5	0.0000	***	0.3333	0.5918	0.6173	0.6128
B at c4	0.0000	46.1119	5	0.0000	***	0.3333	0.6321	0.6627	0.6578
B at c5	0.0000	50.1290	5	0.0000	***	0.3333	0.5426	0.5624	0.5583
C at b1	0.0000	68.4244	9	0.0000	***	0.2500	0.5054	0.5324	0.5285
C at b2	0.0000	60.8486	9	0.0000	***	0.2500	0.5910	0.6306	0.6260
C at b3	0.0000	76.1514	9	0.0000	***	0.2500	0.4715	0.4940	0.4904
C at b4	0.0000	48.4921	9	0.0000	***	0.2500	0.6645	0.7162	0.7110

LB = lower.bound, GG = Greenhouse-Geisser

HF = Huynh-Feldt-Lecoutre, CM = Chi-Muller

Source	SS	df	MS	F-ratio	p-value		G.eta^2
B at c1	0.1353	1.68	0.0806	1.2443	0.2900	ns	0.0168
s x B at c1	4.3491	67.18	0.0647				
B at c2	4.5777	1.55	2.9568	23.9641	0.0000	***	0.2542
s x B at c2	7.6410	61.93	0.1234				
B at c3	7.3365	1.78	4.1324	15.8699	0.0000	***	0.1975
s x B at c3	18.4916	71.01	0.2604				
B at c4	12.1261	1.9	6.3946	27.4006	0.0000	***	0.2112
s x B at c4	17.7020	75.85	0.2334				
B at c5	20.4432	1.63	12.5585	33.0681	0.0000	***	0.2603
s x B at c5	24.7287	65.11	0.3798				
C at b1	26.0079	2.02	12.8654	38.3639	0.0000	***	0.2445

```
s x C at b1 27.1171  80.86  0.3354
--------------------------------------------------------------------
    C at b2 11.7378   2.36  4.9650  27.2383   0.0000 ***   0.2251
s x C at b2 17.2372  94.56  0.1823
--------------------------------------------------------------------
    C at b3  3.4768   1.89  1.8436  13.2790   0.0000 ***   0.1529
s x C at b3 10.4732  75.43  0.1388
--------------------------------------------------------------------
    C at b4  0.6354   2.66  0.2390   4.5878   0.0064 **    0.0420
s x C at b4  5.5396 106.32  0.0521
--------------------------------------------------------------------
                        +p < .10, *p < .05, **p < .01, ***p < .001


< MULTIPLE COMPARISON for "B at c2" >

== Holm's Sequentially Rejective Bonferroni Procedure ==
== The factor < B at c2 > is analysed as dependent means. ==
== Alpha level is 0.05. ==

----------------------------------------------------------
  Pair     Diff  t-value  df       p   adj.p
----------------------------------------------------------
 b1-b3  -0.4146   5.6124  40  0.0000  0.0000  b1 < b3 *
 b1-b4  -0.4024   5.2573  40  0.0000  0.0000  b1 < b4 *
 b1-b2  -0.2927   4.8952  40  0.0000  0.0001  b1 < b2 *
 b2-b3  -0.1220   3.3758  40  0.0016  0.0049  b2 < b3 *
 b2-b4  -0.1098   2.6186  40  0.0124  0.0248  b2 < b4 *
 b3-b4   0.0122   0.4669  40  0.6431  0.6431  b3 = b4
----------------------------------------------------------


< MULTIPLE COMPARISON for "B at c3" >

== Holm's Sequentially Rejective Bonferroni Procedure ==
== The factor < B at c3 > is analysed as dependent means. ==
== Alpha level is 0.05. ==

----------------------------------------------------------
  Pair     Diff  t-value  df       p   adj.p
----------------------------------------------------------
 b1-b4  -0.5183   4.5614  40  0.0000  0.0003  b1 < b4 *
 b1-b3  -0.5122   4.4611  40  0.0001  0.0003  b1 < b3 *
 b1-b2  -0.2927   3.5962  40  0.0009  0.0035  b1 < b2 *
 b2-b4  -0.2256   3.3416  40  0.0018  0.0054  b2 < b4 *
 b2-b3  -0.2195   3.2680  40  0.0022  0.0054  b2 < b3 *
 b3-b4  -0.0061   0.1059  40  0.9162  0.9162  b3 = b4
----------------------------------------------------------


< MULTIPLE COMPARISON for "B at c4" >

== Holm's Sequentially Rejective Bonferroni Procedure ==
== The factor < B at c4 > is analysed as dependent means. ==
```

== Alpha level is 0.05. ==

Pair	Diff	t-value	df	p	adj.p	
b1-b4	-0.6890	6.4704	40	0.0000	0.0000	b1 < b4 *
b2-b4	-0.5061	5.6711	40	0.0000	0.0000	b2 < b4 *
b1-b3	-0.5244	5.2121	40	0.0000	0.0000	b1 < b3 *
b2-b3	-0.3415	4.5236	40	0.0001	0.0002	b2 < b3 *
b3-b4	-0.1646	4.0566	40	0.0002	0.0004	b3 < b4 *
b1-b2	-0.1829	2.2858	40	0.0276	0.0276	b1 < b2 *

< MULTIPLE COMPARISON for "B at c5" >

== Holm's Sequentially Rejective Bonferroni Procedure ==
== The factor < B at c5 > is analysed as dependent means. ==
== Alpha level is 0.05. ==

Pair	Diff	t-value	df	p	adj.p	
b1-b4	-0.9146	6.6444	40	0.0000	0.0000	b1 < b4 *
b1-b3	-0.7622	6.0396	40	0.0000	0.0000	b1 < b3 *
b2-b4	-0.5122	5.4720	40	0.0000	0.0000	b2 < b4 *
b1-b2	-0.4024	4.7831	40	0.0000	0.0001	b1 < b2 *
b2-b3	-0.3598	4.5218	40	0.0001	0.0001	b2 < b3 *
b3-b4	-0.1524	2.6665	40	0.0110	0.0110	b3 < b4 *

< MULTIPLE COMPARISON for "C at b1" >

== Holm's Sequentially Rejective Bonferroni Procedure ==
== The factor < C at b1 > is analysed as dependent means. ==
== Alpha level is 0.05. ==

Pair	Diff	t-value	df	p	adj.p	
c1-c5	1.0854	7.7912	40	0.0000	0.0000	c1 > c5 *
c2-c5	0.6402	6.8670	40	0.0000	0.0000	c2 > c5 *
c3-c5	0.5305	6.6618	40	0.0000	0.0000	c3 > c5 *
c1-c4	0.7378	6.2824	40	0.0000	0.0000	c1 > c4 *
c1-c2	0.4451	5.4502	40	0.0000	0.0000	c1 > c2 *
c4-c5	0.3476	5.0205	40	0.0000	0.0001	c4 > c5 *
c1-c3	0.5549	4.9704	40	0.0000	0.0001	c1 > c3 *
c2-c4	0.2927	4.5663	40	0.0000	0.0001	c2 > c4 *
c3-c4	0.1829	3.3979	40	0.0015	0.0031	c3 > c4 *
c2-c3	0.1098	1.8526	40	0.0713	0.0713	c2 = c3

< MULTIPLE COMPARISON for "C at b2" >

== Holm's Sequentially Rejective Bonferroni Procedure ==
== The factor < C at b2 > is analysed as dependent means. ==
== Alpha level is 0.05. ==

Pair	Diff	t-value	df	p	adj.p	
c1-c5	0.6280	7.0164	40	0.0000	0.0000	c1 > c5 *
c2-c5	0.5305	6.5449	40	0.0000	0.0000	c2 > c5 *
c3-c5	0.4207	5.8253	40	0.0000	0.0000	c3 > c5 *
c1-c4	0.5000	5.5627	40	0.0000	0.0000	c1 > c4 *
c2-c4	0.4024	4.7831	40	0.0000	0.0001	c2 > c4 *
c1-c3	0.2073	4.2717	40	0.0001	0.0006	c1 > c3 *
c3-c4	0.2927	3.5962	40	0.0009	0.0035	c3 > c4 *
c1-c2	0.0976	2.8044	40	0.0077	0.0232	c1 > c2 *
c2-c3	0.1098	2.5119	40	0.0161	0.0323	c2 > c3 *
c4-c5	0.1280	1.7271	40	0.0919	0.0919	c4 = c5

< MULTIPLE COMPARISON for "C at b3" >

== Holm's Sequentially Rejective Bonferroni Procedure ==
== The factor < C at b3 > is analysed as dependent means. ==
== Alpha level is 0.05. ==

Pair	Diff	t-value	df	p	adj.p	
c1-c4	0.2378	4.6074	40	0.0000	0.0004	c1 > c4 *
c1-c5	0.3476	4.5404	40	0.0001	0.0005	c1 > c5 *
c2-c5	0.2927	3.7983	40	0.0005	0.0039	c2 > c5 *
c2-c4	0.1829	3.5921	40	0.0009	0.0062	c2 > c4 *
c3-c5	0.2805	3.5437	40	0.0010	0.0062	c3 > c5 *
c3-c4	0.1707	3.4878	40	0.0012	0.0062	c3 > c4 *
c4-c5	0.1098	2.4632	40	0.0182	0.0727	c4 = c5
c1-c2	0.0549	1.5960	40	0.1184	0.3551	c1 = c2
c1-c3	0.0671	1.5674	40	0.1249	0.3551	c1 = c3
c2-c3	0.0122	0.3497	40	0.7284	0.7284	c2 = c3

< MULTIPLE COMPARISON for "C at b4" >

== Holm's Sequentially Rejective Bonferroni Procedure ==
== The factor < C at b4 > is analysed as dependent means. ==
== Alpha level is 0.05. ==

Pair	Diff	t-value	df	p	adj.p	
c1-c5	0.1585	2.9553	40	0.0052	0.0522	c1 = c5

c3-c5	0.1341	2.8008	40	0.0078	0.0703	c3 = c5
c2-c5	0.1280	2.6724	40	0.0108	0.0868	c2 = c5
c4-c5	0.1220	2.5451	40	0.0149	0.1042	c4 = c5
c1-c4	0.0366	1.2325	40	0.2250	1.0000	c1 = c4
c1-c2	0.0305	1.1516	40	0.2563	1.0000	c1 = c2
c1-c3	0.0244	0.6278	40	0.5337	1.0000	c1 = c3
c3-c4	0.0122	0.4221	40	0.6752	1.0000	c3 = c4
c2-c4	0.0061	0.1835	40	0.8553	1.0000	c2 = c4
c2-c3	-0.0061	0.1332	40	0.8947	1.0000	c2 = c3

output is over --------------------///

[[Simple Effects for a3]]

[sAB-Type Design]

This output was generated by anovakun 4.8.5 under R version 4.0.2.
It was executed on Mon Oct 11 18:22:25 2021.

<< DESCRIPTIVE STATISTICS >>

B	C	n	Mean	S.D.
b1	c1	46	-0.0652	0.2496
b1	c2	46	-0.4185	0.4946
b1	c3	46	-0.7337	0.7273
b1	c4	46	-0.9891	0.9617
b1	c5	46	-1.5652	1.0832
b2	c1	46	0.0000	0.0527
b2	c2	46	-0.1576	0.3389
b2	c3	46	-0.3152	0.4231
b2	c4	46	-0.6250	0.5980
b2	c5	46	-0.9402	0.6671
b3	c1	46	0.0109	0.0906
b3	c2	46	-0.0217	0.2347
b3	c3	46	-0.2120	0.4624
b3	c4	46	-0.2065	0.4160
b3	c5	46	-0.4022	0.5413
b4	c1	46	0.0109	0.1577
b4	c2	46	-0.0109	0.1173
b4	c3	46	-0.0652	0.2710
b4	c4	46	-0.0978	0.3139
b4	c5	46	-0.2880	0.5296

<< SPHERICITY INDICES >>

== Mendoza's Multisample Sphericity Test and Epsilons ==

Effect	Lambda	approx.Chi	df	p		LB	GG	HF	CM
Global	0.0000	936.5255	189	0.0000	***	0.0526	0.1887	0.2070	0.2058
B	0.0000	87.3948	5	0.0000	***	0.3333	0.4859	0.4981	0.4952
C	0.0000	95.5785	9	0.0000	***	0.2500	0.4912	0.5134	0.5105
B x C	0.0000	262.6488	77	0.0000	***	0.0833	0.4860	0.5666	0.5633

LB = lower.bound, GG = Greenhouse-Geisser
HF = Huynh-Feldt-Lecoutre, CM = Chi-Muller

<< ANOVA TABLE >>

== Adjusted by Greenhouse-Geisser's Epsilon ==

Source	SS	df	MS	F-ratio	p-value		G.eta^2
s	87.2647	45	1.9392				
B	61.6312	1.46	42.2762	51.4098	0.0000	***	0.2063
s x B	53.9469	65.6	0.8223				
C	68.5774	1.96	34.9015	70.5206	0.0000	***	0.2244
s x C	43.7601	88.42	0.4949				
B x C	25.1171	5.83	4.3065	21.7066	0.0000	***	0.0958
s x B x C	52.0704	262.46	0.1984				
Total	392.3679	919	0.4270				

+p < .10, *p < .05, **p < .01, ***p < .001

<< POST ANALYSES >>

< MULTIPLE COMPARISON for "B" >

== Holm's Sequentially Rejective Bonferroni Procedure ==
== The factor < B > is analysed as dependent means. ==
== Alpha level is 0.05. ==

B	n	Mean	S.D.

b1	230	-0.7543	0.9153
b2	230	-0.4076	0.5746
b3	230	-0.1663	0.4104
b4	230	-0.0902	0.3284

Pair	Diff	t-value	df	p	adj.p	
b2-b3	-0.2413	8.7577	45	0.0000	0.0000	b2 < b3 *
b1-b4	-0.6641	7.8521	45	0.0000	0.0000	b1 < b4 *
b1-b3	-0.5880	7.7521	45	0.0000	0.0000	b1 < b3 *
b2-b4	-0.3174	7.3395	45	0.0000	0.0000	b2 < b4 *
b1-b2	-0.3467	5.2119	45	0.0000	0.0000	b1 < b2 *
b3-b4	-0.0761	2.5564	45	0.0140	0.0140	b3 < b4 *

< MULTIPLE COMPARISON for "C" >

== Holm's Sequentially Rejective Bonferroni Procedure ==
== The factor < C > is analysed as dependent means. ==
== Alpha level is 0.05. ==

C	n	Mean	S.D.
c1	184	-0.0109	0.1586
c2	184	-0.1522	0.3639
c3	184	-0.3315	0.5539
c4	184	-0.4796	0.7128
c5	184	-0.7989	0.8926

Pair	Diff	t-value	df	p	adj.p	
c2-c5	0.6467	11.2981	45	0.0000	0.0000	c2 > c5 *
c1-c5	0.7880	9.9307	45	0.0000	0.0000	c1 > c5 *
c3-c5	0.4674	9.3314	45	0.0000	0.0000	c3 > c5 *
c2-c4	0.3274	7.6520	45	0.0000	0.0000	c2 > c4 *
c1-c4	0.4688	7.1314	45	0.0000	0.0000	c1 > c4 *
c4-c5	0.3193	6.7253	45	0.0000	0.0000	c4 > c5 *
c1-c3	0.3207	6.1614	45	0.0000	0.0000	c1 > c3 *
c2-c3	0.1793	5.3070	45	0.0000	0.0000	c2 > c3 *
c3-c4	0.1481	4.8179	45	0.0000	0.0000	c3 > c4 *
c1-c2	0.1413	4.1824	45	0.0001	0.0001	c1 > c2 *

< SIMPLE EFFECTS for "B x C" INTERACTION >

```
------
  Effect  Lambda  approx.Chi  df      p         LB     GG     HF
CM
--------------------------------------------------------------------
------
 B at c1  0.0000     70.4796   5 0.0000 *** 0.3333 0.5365 0.5534
0.5502
 B at c2  0.0000     36.2544   5 0.0000 *** 0.3333 0.6605 0.6908
0.6868
 B at c3  0.0001     17.5019   5 0.0036 **  0.3333 0.7718 0.8161
0.8114
 B at c4  0.0000     45.5669   5 0.0000 *** 0.3333 0.6062 0.6303
0.6266
 B at c5  0.0000     35.6414   5 0.0000 *** 0.3333 0.6672 0.6983
0.6943
 C at b1  0.0000     72.0489   9 0.0000 *** 0.2500 0.5400 0.5682
0.5649
 C at b2  0.0000     49.3586   9 0.0000 *** 0.2500 0.6155 0.6538
0.6500
 C at b3  0.0000     46.2855   9 0.0000 *** 0.2500 0.7470 0.8061
0.8014
 C at b4  0.0000     94.4459   9 0.0000 *** 0.2500 0.5347 0.5622
0.5589
--------------------------------------------------------------------
------
                               LB = lower.bound, GG = Greenhouse-
Geisser
                              HF = Huynh-Feldt-Lecoutre, CM = Chi-
Muller

--------------------------------------------------------------------
     Source      SS     df       MS  F-ratio  p-value      G.eta^2
--------------------------------------------------------------------
    B at c1  0.1848   1.61  0.1148   2.6065   0.0918 +     0.0401
s x B at c1  3.1902  72.42  0.0440
--------------------------------------------------------------------
    B at c2  4.9647   1.98  2.5054  24.3889   0.0000 ***   0.2048
s x B at c2  9.1603  89.17  0.1027
--------------------------------------------------------------------
    B at c3 11.3723   2.32  4.9118  26.4948   0.0000 ***   0.2025
s x B at c3 19.3152 104.19  0.1854
--------------------------------------------------------------------
    B at c4 23.0499   1.82 12.6753  34.4349   0.0000 ***   0.2479
s x B at c4 30.1219  81.83  0.3681
--------------------------------------------------------------------
    B at c5 47.1766      2 23.5682  47.9983   0.0000 ***   0.3235
s x B at c5 44.2296  90.08  0.4910
--------------------------------------------------------------------
    C at b1 59.8353   2.16 27.6992  61.2444   0.0000 ***   0.3119
s x C at b1 43.9647  97.21  0.4523
--------------------------------------------------------------------
    C at b2 26.1332   2.46 10.6151  57.0403   0.0000 ***   0.3457
s x C at b2 20.6168 110.78  0.1861
--------------------------------------------------------------------
```

C at b3	5.1348	2.99	1.7184	13.8651	0.0000	***	0.1331
s x C at b3	16.6652	134.47	0.1239				
C at b4	2.5913	2.14	1.2116	7.9958	0.0005	***	0.1050
s x C at b4	14.5837	96.25	0.1515				

+p < .10, *p < .05, **p < .01, ***p < .001

< MULTIPLE COMPARISON for "B at c1" >

== Holm's Sequentially Rejective Bonferroni Procedure ==
== The factor < B at c1 > is analysed as dependent means. ==
== Alpha level is 0.05. ==

Pair	Diff	t-value	df	p	adj.p	
b1-b3	-0.0761	1.9251	45	0.0606	0.3633	b1 = b3
b1-b4	-0.0761	1.7078	45	0.0946	0.4728	b1 = b4
b1-b2	-0.0652	1.6978	45	0.0964	0.4728	b1 = b2
b2-b3	-0.0109	0.8135	45	0.4202	1.0000	b2 = b3
b2-b4	-0.0109	0.5303	45	0.5985	1.0000	b2 = b4
b3-b4	0.0000	0.0000	45	1.0000	1.0000	b3 = b4

< MULTIPLE COMPARISON for "B at c2" >

== Holm's Sequentially Rejective Bonferroni Procedure ==
== The factor < B at c2 > is analysed as dependent means. ==
== Alpha level is 0.05. ==

Pair	Diff	t-value	df	p	adj.p	
b1-b4	-0.4076	5.9637	45	0.0000	0.0000	b1 < b4 *
b1-b3	-0.3967	5.9120	45	0.0000	0.0000	b1 < b3 *
b1-b2	-0.2609	4.1977	45	0.0001	0.0005	b1 < b2 *
b2-b3	-0.1359	3.4485	45	0.0012	0.0037	b2 < b3 *
b2-b4	-0.1467	3.2114	45	0.0024	0.0049	b2 < b4 *
b3-b4	-0.0109	0.3397	45	0.7357	0.7357	b3 = b4

< MULTIPLE COMPARISON for "B at c3" >

== Holm's Sequentially Rejective Bonferroni Procedure ==
== The factor < B at c3 > is analysed as dependent means. ==
== Alpha level is 0.05. ==

Pair	Diff	t-value	df	p	adj.p

```
 b1-b4  -0.6685   6.6756  45  0.0000  0.0000  b1 < b4 *
 b1-b3  -0.5217   6.0094  45  0.0000  0.0000  b1 < b3 *
 b1-b2  -0.4185   4.7776  45  0.0000  0.0001  b1 < b2 *
 b2-b4  -0.2500   3.9014  45  0.0003  0.0009  b2 < b4 *
 b3-b4  -0.1467   2.2803  45  0.0274  0.0548  b3 = b4
 b2-b3  -0.1033   1.6666  45  0.1026  0.1026  b2 = b3
----------------------------------------------------------


< MULTIPLE COMPARISON for "B at c4" >

== Holm's Sequentially Rejective Bonferroni Procedure ==
== The factor < B at c4 > is analysed as dependent means. ==
== Alpha level is 0.05. ==

----------------------------------------------------------
  Pair     Diff  t-value  df       p   adj.p
----------------------------------------------------------
 b2-b3  -0.4185   6.9491  45  0.0000  0.0000  b2 < b3 *
 b1-b4  -0.8913   6.7745  45  0.0000  0.0000  b1 < b4 *
 b2-b4  -0.5272   6.5821  45  0.0000  0.0000  b2 < b4 *
 b1-b3  -0.7826   6.4393  45  0.0000  0.0000  b1 < b3 *
 b1-b2  -0.3641   3.2671  45  0.0021  0.0042  b1 < b2 *
 b3-b4  -0.1087   1.7961  45  0.0792  0.0792  b3 = b4
----------------------------------------------------------


< MULTIPLE COMPARISON for "B at c5" >

== Holm's Sequentially Rejective Bonferroni Procedure ==
== The factor < B at c5 > is analysed as dependent means. ==
== Alpha level is 0.05. ==

----------------------------------------------------------
  Pair     Diff  t-value  df       p   adj.p
----------------------------------------------------------
 b1-b3  -1.1630   8.4567  45  0.0000  0.0000  b1 < b3 *
 b1-b4  -1.2772   8.0559  45  0.0000  0.0000  b1 < b4 *
 b2-b3  -0.5380   7.1049  45  0.0000  0.0000  b2 < b3 *
 b2-b4  -0.6522   6.1458  45  0.0000  0.0000  b2 < b4 *
 b1-b2  -0.6250   4.6927  45  0.0000  0.0001  b1 < b2 *
 b3-b4  -0.1141   1.3959  45  0.1696  0.1696  b3 = b4
----------------------------------------------------------


< MULTIPLE COMPARISON for "C at b1" >

== Holm's Sequentially Rejective Bonferroni Procedure ==
== The factor < C at b1 > is analysed as dependent means. ==
== Alpha level is 0.05. ==

---------------------------------------------------------
  Pair    Diff  t-value  df       p   adj.p
---------------------------------------------------------
```

c1-c5	1.5000	9.7619	45	0.0000	0.0000	c1 > c5 *
c2-c5	1.1467	9.6556	45	0.0000	0.0000	c2 > c5 *
c3-c5	0.8315	9.0418	45	0.0000	0.0000	c3 > c5 *
c1-c4	0.9239	7.1713	45	0.0000	0.0000	c1 > c4 *
c1-c3	0.6685	6.9534	45	0.0000	0.0000	c1 > c3 *
c2-c4	0.5707	6.1482	45	0.0000	0.0000	c2 > c4 *
c4-c5	0.5761	5.7218	45	0.0000	0.0000	c4 > c5 *
c1-c2	0.3533	5.1284	45	0.0000	0.0000	c1 > c2 *
c2-c3	0.3152	4.4409	45	0.0001	0.0001	c2 > c3 *
c3-c4	0.2554	3.3996	45	0.0014	0.0014	c3 > c4 *

< MULTIPLE COMPARISON for "C at b2" >

== Holm's Sequentially Rejective Bonferroni Procedure ==
== The factor < C at b2 > is analysed as dependent means. ==
== Alpha level is 0.05. ==

Pair	Diff	t-value	df	p	adj.p	
c2-c5	0.7826	10.1419	45	0.0000	0.0000	c2 > c5 *
c1-c5	0.9402	9.5007	45	0.0000	0.0000	c1 > c5 *
c3-c5	0.6250	9.4951	45	0.0000	0.0000	c3 > c5 *
c2-c4	0.4674	7.1077	45	0.0000	0.0000	c2 > c4 *
c1-c4	0.6250	7.0882	45	0.0000	0.0000	c1 > c4 *
c1-c3	0.3152	5.0534	45	0.0000	0.0000	c1 > c3 *
c4-c5	0.3152	4.9035	45	0.0000	0.0001	c4 > c5 *
c3-c4	0.3098	4.7585	45	0.0000	0.0001	c3 > c4 *
c1-c2	0.1576	3.1537	45	0.0029	0.0057	c1 > c2 *
c2-c3	0.1576	2.9472	45	0.0051	0.0057	c2 > c3 *

< MULTIPLE COMPARISON for "C at b3" >

== Holm's Sequentially Rejective Bonferroni Procedure ==
== The factor < C at b3 > is analysed as dependent means. ==
== Alpha level is 0.05. ==

Pair	Diff	t-value	df	p	adj.p	
c2-c5	0.3804	5.6629	45	0.0000	0.0000	c2 > c5 *
c1-c5	0.4130	5.2318	45	0.0000	0.0000	c1 > c5 *
c2-c4	0.1848	3.9946	45	0.0002	0.0019	c2 > c4 *
c1-c4	0.2174	3.5641	45	0.0009	0.0061	c1 > c4 *
c1-c3	0.2228	3.2215	45	0.0024	0.0142	c1 > c3 *
c4-c5	0.1957	3.1012	45	0.0033	0.0166	c4 > c5 *
c2-c3	0.1902	2.9013	45	0.0057	0.0229	c2 > c3 *
c3-c5	0.1902	2.5358	45	0.0148	0.0443	c3 > c5 *
c1-c2	0.0326	0.9476	45	0.3484	0.6968	c1 = c2
c3-c4	-0.0054	0.0885	45	0.9299	0.9299	c3 = c4

< MULTIPLE COMPARISON for "C at b4" >

== Holm's Sequentially Rejective Bonferroni Procedure ==
== The factor < C at b4 > is analysed as dependent means. ==
== Alpha level is 0.05. ==

Pair	Diff	t-value	df	p	adj.p	
c1-c5	0.2989	3.8932	45	0.0003	0.0032	c1 > c5 *
c2-c5	0.2772	3.4445	45	0.0012	0.0112	c2 > c5 *
c3-c5	0.2228	3.4217	45	0.0013	0.0112	c3 > c5 *
c4-c5	0.1902	2.2328	45	0.0306	0.2141	c4 = c5
c2-c4	0.0870	2.1449	45	0.0374	0.2244	c2 = c4
c1-c4	0.1087	2.0479	45	0.0464	0.2322	c1 = c4
c1-c3	0.0761	1.6125	45	0.1138	0.4554	c1 = c3
c2-c3	0.0543	1.5673	45	0.1241	0.4554	c2 = c3
c1-c2	0.0217	0.7265	45	0.4713	0.9425	c1 = c2
c3-c4	0.0326	0.6429	45	0.5236	0.9425	c3 = c4

output is over --------------------///

[[Simple Effects for a4]]

[sAB-Type Design]

This output was generated by anovakun 4.8.5 under R version 4.0.2.
It was executed on Mon Oct 11 18:22:26 2021.

<< DESCRIPTIVE STATISTICS >>

B	C	n	Mean	S.D.
b1	c1	44	0.0227	0.3316
b1	c2	44	-0.2784	0.6057
b1	c3	44	-0.6080	0.8130
b1	c4	44	-0.7955	0.9265
b1	c5	44	-1.0341	0.9684
b2	c1	44	0.0455	0.3015
b2	c2	44	-0.2045	0.3707
b2	c3	44	-0.3409	0.4944
b2	c4	44	-0.4602	0.6122
b2	c5	44	-0.8068	0.8758
b3	c1	44	0.0114	0.1422
b3	c2	44	-0.0398	0.2219
b3	c3	44	-0.1875	0.3150
b3	c4	44	-0.3182	0.4649

b3	c5	44	-0.5398	0.6170
b4	c1	44	0.0057	0.1373
b4	c2	44	0.0227	0.1301
b4	c3	44	-0.0568	0.1693
b4	c4	44	-0.1193	0.3120
b4	c5	44	-0.2955	0.5037

<< SPHERICITY INDICES >>

== Mendoza's Multisample Sphericity Test and Epsilons ==

Effect	Lambda	approx.Chi	df	p		LB	GG	HF	CM
Global	0.0000	911.5294	189	0.0000	***	0.0526	0.2248	0.2527	0.2511
B	0.0000	80.7478	5	0.0000	***	0.3333	0.4958	0.5094	0.5062
C	0.0000	117.6883	9	0.0000	***	0.2500	0.3911	0.4033	0.4007
B x C	0.0000	303.2337	77	0.0000	***	0.0833	0.4306	0.4964	0.4932

LB = lower.bound, GG = Greenhouse-Geisser
HF = Huynh-Feldt-Lecoutre, CM = Chi-Muller

<< ANOVA TABLE >>

== Adjusted by Greenhouse-Geisser's Epsilon ==

Source	SS	df	MS	F-ratio	p-value		G.eta^2
s	102.6864	43	2.3881				
B	24.5813	1.49	16.5278	20.5316	0.0000	***	0.0907
s x B	51.4813	63.95	0.8050				
C	50.2036	1.56	32.0878	52.7214	0.0000	***	0.1693
s x C	40.9464	67.28	0.6086				
B x C	9.7391	5.17	1.8848	8.1696	0.0000	***	0.0380
s x B x C	51.2609	222.19	0.2307				
Total	330.8989	879	0.3764				

```
                    +p < .10, *p < .05, **p < .01, ***p < .001


<< POST ANALYSES >>

< MULTIPLE COMPARISON for "B" >

== Holm's Sequentially Rejective Bonferroni Procedure ==
== The factor < B > is analysed as dependent means. ==
== Alpha level is 0.05. ==

---------------------------
  B   n     Mean    S.D.
---------------------------
 b1 220  -0.5386  0.8465
 b2 220  -0.3534  0.6301
 b3 220  -0.2148  0.4362
 b4 220  -0.0886  0.3080
---------------------------

----------------------------------------------------------
  Pair     Diff  t-value  df       p   adj.p
----------------------------------------------------------
 b2-b4  -0.2648   5.7243  43  0.0000  0.0000  b2 < b4 *
 b3-b4  -0.1261   5.4663  43  0.0000  0.0000  b3 < b4 *
 b1-b4  -0.4500   5.4508  43  0.0000  0.0000  b1 < b4 *
 b1-b3  -0.3239   3.9912  43  0.0003  0.0008  b1 < b3 *
 b2-b3  -0.1386   3.3954  43  0.0015  0.0030  b2 < b3 *
 b1-b2  -0.1852   2.9181  43  0.0056  0.0056  b1 < b2 *
----------------------------------------------------------


< MULTIPLE COMPARISON for "C" >

== Holm's Sequentially Rejective Bonferroni Procedure ==
== The factor < C > is analysed as dependent means. ==
== Alpha level is 0.05. ==

---------------------------
  C   n     Mean    S.D.
---------------------------
 c1 176   0.0213  0.2433
 c2 176  -0.1250  0.3937
 c3 176  -0.2983  0.5443
 c4 176  -0.4233  0.6642
 c5 176  -0.6690  0.8077
---------------------------

---------------------------------------------------------
  Pair    Diff  t-value  df       p   adj.p
---------------------------------------------------------
 c2-c5  0.5440   8.6973  43  0.0000  0.0000  c2 > c5 *
 c1-c5  0.6903   8.0231  43  0.0000  0.0000  c1 > c5 *
 c3-c5  0.3707   7.1157  43  0.0000  0.0000  c3 > c5 *
```

```
 c2-c4  0.2983   6.8981  43  0.0000  0.0000  c2 > c4 *
 c1-c3  0.3196   6.8176  43  0.0000  0.0000  c1 > c3 *
 c1-c4  0.4446   6.7741  43  0.0000  0.0000  c1 > c4 *
 c2-c3  0.1733   6.6218  43  0.0000  0.0000  c2 > c3 *
 c4-c5  0.2457   6.1441  43  0.0000  0.0000  c4 > c5 *
 c1-c2  0.1463   4.2728  43  0.0001  0.0002  c1 > c2 *
 c3-c4  0.1250   3.6248  43  0.0008  0.0008  c3 > c4 *
---------------------------------------------------------


< SIMPLE EFFECTS for "B x C" INTERACTION >

--------------------------------------------------------------------
------
  Effect  Lambda  approx.Chi  df      p         LB     GG     HF
CM
--------------------------------------------------------------------
------
 B at c1  0.0000     52.3540   5 0.0000 *** 0.3333 0.7092 0.7472
0.7424
 B at c2  0.0000     53.6609   5 0.0000 *** 0.3333 0.5793 0.6015
0.5977
 B at c3  0.0000     69.7086   5 0.0000 *** 0.3333 0.5110 0.5261
0.5228
 B at c4  0.0000     44.0283   5 0.0000 *** 0.3333 0.5955 0.6196
0.6156
 B at c5  0.0000     39.1771   5 0.0000 *** 0.3333 0.6820 0.7164
0.7119
 C at b1  0.0000     50.5430   9 0.0000 *** 0.2500 0.6845 0.7357
0.7310
 C at b2  0.0000     72.3410   9 0.0000 *** 0.2500 0.5182 0.5449
0.5415
 C at b3  0.0000    103.1570   9 0.0000 *** 0.2500 0.4609 0.4806
0.4776
 C at b4  0.0000     70.6986   9 0.0000 *** 0.2500 0.5529 0.5841
0.5804
--------------------------------------------------------------------
------
                               LB = lower.bound, GG = Greenhouse-
Geisser
                              HF = Huynh-Feldt-Lecoutre, CM = Chi-
Muller

--------------------------------------------------------------------
     Source      SS     df       MS  F-ratio  p-value      G.eta^2
--------------------------------------------------------------------
    B at c1  0.0408   2.13  0.0192   0.3245   0.7370 ns    0.0039
s x B at c1  5.4123  91.49  0.0592
--------------------------------------------------------------------
    B at c2  2.5938   1.74  1.4926   9.6721   0.0004 ***   0.0956
s x B at c2 11.5312  74.73  0.1543
--------------------------------------------------------------------
    B at c3  7.4048   1.53  4.8304  14.3904   0.0000 ***   0.1428
s x B at c3 22.1264  65.92  0.3357
```

B at c4	10.7060	1.79	5.9929	18.2453	0.0000	***	0.1387
s x B at c4	25.2315	76.82	0.3285				
B at c5	13.5749	2.05	6.6351	15.1850	0.0000	***	0.1189
s x B at c5	38.4407	87.98	0.4369				
C at b1	30.7597	2.74	11.2340	37.1375	0.0000	***	0.1960
s x C at b1	35.6153	117.74	0.3025				
C at b2	17.5295	2.07	8.4564	25.1295	0.0000	***	0.2016
s x C at b2	29.9955	89.14	0.3365				
C at b3	8.7483	1.84	4.7448	24.3848	0.0000	***	0.2100
s x C at b3	15.4267	79.28	0.1946				
C at b4	2.9051	2.21	1.3137	11.1836	0.0000	***	0.1399
s x C at b4	11.1699	95.09	0.1175				

+p < .10, *p < .05, **p < .01, ***p < .001

< MULTIPLE COMPARISON for "B at c2" >

== Holm's Sequentially Rejective Bonferroni Procedure ==
== The factor < B at c2 > is analysed as dependent means. ==
== Alpha level is 0.05. ==

Pair	Diff	t-value	df	p	adj.p	
b2-b4	-0.2273	4.4878	43	0.0001	0.0003	b2 < b4 *
b2-b3	-0.1648	3.7046	43	0.0006	0.0030	b2 < b3 *
b1-b4	-0.3011	3.6580	43	0.0007	0.0030	b1 < b4 *
b1-b3	-0.2386	2.8795	43	0.0062	0.0186	b1 < b3 *
b3-b4	-0.0625	2.0459	43	0.0469	0.0938	b3 = b4
b1-b2	-0.0739	1.0187	43	0.3141	0.3141	b1 = b2

< MULTIPLE COMPARISON for "B at c3" >

== Holm's Sequentially Rejective Bonferroni Procedure ==
== The factor < B at c3 > is analysed as dependent means. ==
== Alpha level is 0.05. ==

Pair	Diff	t-value	df	p	adj.p	
b1-b4	-0.5511	4.4751	43	0.0001	0.0003	b1 < b4 *
b2-b4	-0.2841	4.0200	43	0.0002	0.0012	b2 < b4 *
b1-b3	-0.4205	3.6507	43	0.0007	0.0028	b1 < b3 *
b3-b4	-0.1307	3.3174	43	0.0019	0.0056	b3 < b4 *
b1-b2	-0.2670	2.9457	43	0.0052	0.0104	b1 < b2 *

Pair	Diff	t-value	df	p	adj.p	
b2-b3	-0.1534	2.5633	43	0.0140	0.0140	b2 < b3 *

< MULTIPLE COMPARISON for "B at c4" >

== Holm's Sequentially Rejective Bonferroni Procedure ==
== The factor < B at c4 > is analysed as dependent means. ==
== Alpha level is 0.05. ==

Pair	Diff	t-value	df	p	adj.p	
b2-b4	-0.3409	5.6001	43	0.0000	0.0000	b2 < b4 *
b1-b4	-0.6761	5.4082	43	0.0000	0.0000	b1 < b4 *
b1-b3	-0.4773	4.2069	43	0.0001	0.0005	b1 < b3 *
b3-b4	-0.1989	3.5469	43	0.0010	0.0029	b3 < b4 *
b1-b2	-0.3352	2.9240	43	0.0055	0.0110	b1 < b2 *
b2-b3	-0.1420	2.0398	43	0.0475	0.0475	b2 < b3 *

< MULTIPLE COMPARISON for "B at c5" >

== Holm's Sequentially Rejective Bonferroni Procedure ==
== The factor < B at c5 > is analysed as dependent means. ==
== Alpha level is 0.05. ==

Pair	Diff	t-value	df	p	adj.p	
b1-b4	-0.7386	5.5411	43	0.0000	0.0000	b1 < b4 *
b2-b4	-0.5114	5.0540	43	0.0000	0.0000	b2 < b4 *
b3-b4	-0.2443	4.1099	43	0.0002	0.0007	b3 < b4 *
b1-b3	-0.4943	3.3709	43	0.0016	0.0048	b1 < b3 *
b2-b3	-0.2670	2.7247	43	0.0093	0.0185	b2 < b3 *
b1-b2	-0.2273	1.6656	43	0.1031	0.1031	b1 = b2

< MULTIPLE COMPARISON for "C at b1" >

== Holm's Sequentially Rejective Bonferroni Procedure ==
== The factor < C at b1 > is analysed as dependent means. ==
== Alpha level is 0.05. ==

Pair	Diff	t-value	df	p	adj.p	
c1-c5	1.0568	8.6322	43	0.0000	0.0000	c1 > c5 *
c2-c5	0.7557	7.8438	43	0.0000	0.0000	c2 > c5 *
c1-c4	0.8182	6.7655	43	0.0000	0.0000	c1 > c4 *
c1-c3	0.6307	6.5463	43	0.0000	0.0000	c1 > c3 *
c3-c5	0.4261	6.3308	43	0.0000	0.0000	c3 > c5 *

c2-c4	0.5170	5.7033	43	0.0000	0.0000	c2 > c4 *
c1-c2	0.3011	4.6463	43	0.0000	0.0001	c1 > c2 *
c2-c3	0.3295	4.4297	43	0.0001	0.0002	c2 > c3 *
c4-c5	0.2386	2.0659	43	0.0449	0.0898	c4 = c5
c3-c4	0.1875	1.8642	43	0.0691	0.0898	c3 = c4

< MULTIPLE COMPARISON for "C at b2" >

== Holm's Sequentially Rejective Bonferroni Procedure ==
== The factor < C at b2 > is analysed as dependent means. ==
== Alpha level is 0.05. ==

Pair	Diff	t-value	df	p	adj.p	
c1-c5	0.8523	6.0405	43	0.0000	0.0000	c1 > c5 *
c2-c5	0.6023	5.7283	43	0.0000	0.0000	c2 > c5 *
c1-c4	0.5057	5.5099	43	0.0000	0.0000	c1 > c4 *
c1-c3	0.3864	4.7664	43	0.0000	0.0002	c1 > c3 *
c2-c4	0.2557	4.5647	43	0.0000	0.0002	c2 > c4 *
c3-c5	0.4659	4.4815	43	0.0001	0.0003	c3 > c5 *
c1-c2	0.2500	3.6762	43	0.0007	0.0026	c1 > c2 *
c4-c5	0.3466	3.6139	43	0.0008	0.0026	c4 > c5 *
c2-c3	0.1364	2.9913	43	0.0046	0.0092	c2 > c3 *
c3-c4	0.1193	2.0262	43	0.0490	0.0490	c3 > c4 *

< MULTIPLE COMPARISON for "C at b3" >

== Holm's Sequentially Rejective Bonferroni Procedure ==
== The factor < C at b3 > is analysed as dependent means. ==
== Alpha level is 0.05. ==

Pair	Diff	t-value	df	p	adj.p	
c2-c5	0.5000	5.8920	43	0.0000	0.0000	c2 > c5 *
c1-c5	0.5511	5.6785	43	0.0000	0.0000	c1 > c5 *
c3-c5	0.3523	5.2434	43	0.0000	0.0000	c3 > c5 *
c1-c4	0.3295	4.8889	43	0.0000	0.0001	c1 > c4 *
c2-c4	0.2784	4.6518	43	0.0000	0.0002	c2 > c4 *
c2-c3	0.1477	4.2464	43	0.0001	0.0006	c2 > c3 *
c1-c3	0.1989	3.8889	43	0.0003	0.0014	c1 > c3 *
c4-c5	0.2216	3.8094	43	0.0004	0.0014	c4 > c5 *
c3-c4	0.1307	2.2854	43	0.0273	0.0546	c3 = c4
c1-c2	0.0511	1.5939	43	0.1183	0.1183	c1 = c2

< MULTIPLE COMPARISON for "C at b4" >

== Holm's Sequentially Rejective Bonferroni Procedure ==
== The factor < C at b4 > is analysed as dependent means. ==
== Alpha level is 0.05. ==

Pair	Diff	t-value	df	p	adj.p	
c2-c5	0.3182	4.3941	43	0.0001	0.0007	c2 > c5 *
c1-c5	0.3011	4.1794	43	0.0001	0.0013	c1 > c5 *
c3-c5	0.2386	3.3252	43	0.0018	0.0145	c3 > c5 *
c2-c4	0.1420	3.1188	43	0.0032	0.0227	c2 > c4 *
c4-c5	0.1761	2.6360	43	0.0116	0.0697	c4 = c5
c1-c4	0.1250	2.5811	43	0.0133	0.0697	c1 = c4
c2-c3	0.0795	2.5462	43	0.0146	0.0697	c2 = c3
c1-c3	0.0625	1.9149	43	0.0622	0.1865	c1 = c3
c3-c4	0.0625	1.2793	43	0.2076	0.4153	c3 = c4
c1-c2	-0.0170	0.6841	43	0.4976	0.4976	c1 = c2

output is over --------------------///

[[Simple Effects for a5]]

[sAB-Type Design]

This output was generated by anovakun 4.8.5 under R version 4.0.2.
It was executed on Mon Oct 11 18:22:27 2021.

<< DESCRIPTIVE STATISTICS >>

B	C	n	Mean	S.D.
b1	c1	44	-0.1875	0.3855
b1	c2	44	-0.7216	0.6630
b1	c3	44	-1.0398	0.9384
b1	c4	44	-1.3409	1.1011
b1	c5	44	-1.8750	1.2912
b2	c1	44	-0.1136	0.2825
b2	c2	44	-0.3920	0.5665
b2	c3	44	-0.6932	0.7089
b2	c4	44	-1.0341	0.8134
b2	c5	44	-1.3352	1.0633
b3	c1	44	-0.0568	0.1857
b3	c2	44	-0.1307	0.3906
b3	c3	44	-0.3920	0.5741
b3	c4	44	-0.6364	0.7576
b3	c5	44	-0.8636	0.8499
b4	c1	44	-0.0398	0.2347
b4	c2	44	-0.0795	0.3270
b4	c3	44	-0.2898	0.5730
b4	c4	44	-0.3693	0.5943

```
 b4  c5  44  -0.5795  0.7601
-------------------------------
```

<< SPHERICITY INDICES >>

== Mendoza's Multisample Sphericity Test and Epsilons ==

Effect	Lambda	approx.Chi	df	p		LB	GG	HF	CM
Global	0.0000	918.4691	189	0.0000	***	0.0526	0.1446	0.1554	0.1544
B	0.0000	129.9266	5	0.0000	***	0.3333	0.4073	0.4130	0.4104
C	0.0000	172.8961	9	0.0000	***	0.2500	0.3293	0.3356	0.3335
B x C	0.0000	208.5531	77	0.0000	***	0.0833	0.4998	0.5899	0.5861

LB = lower.bound, GG = Greenhouse-Geisser
HF = Huynh-Feldt-Lecoutre, CM = Chi-Muller

<< ANOVA TABLE >>

== Adjusted by Greenhouse-Geisser's Epsilon ==

Source	SS	df	MS	F-ratio	p-value		G.eta^2
s	283.6611	43	6.5968				
B	75.1991	1.22	61.5462	64.2845	0.0000	***	0.1454
s x B	50.3009	52.54	0.9574				
C	123.2128	1.32	93.5430	77.7927	0.0000	***	0.2180
s x C	68.1060	56.64	1.2025				
B x C	18.6361	6	3.1070	20.0864	0.0000	***	0.0405
s x B x C	39.8952	257.92	0.1547				
Total	659.0111	879	0.7497				

+p < .10, *p < .05, **p < .01, ***p < .001

<< POST ANALYSES >>

< MULTIPLE COMPARISON for "B" >

== Holm's Sequentially Rejective Bonferroni Procedure ==
== The factor < B > is analysed as dependent means. ==
== Alpha level is 0.05. ==

B	n	Mean	S.D.
b1	220	-1.0330	1.0855
b2	220	-0.7136	0.8489
b3	220	-0.4159	0.6696
b4	220	-0.2716	0.5642

Pair	Diff	t-value	df	p	adj.p	
b1-b4	-0.7614	8.4106	43	0.0000	0.0000	b1 < b4 *
b1-b3	-0.6170	8.2361	43	0.0000	0.0000	b1 < b3 *
b2-b4	-0.4420	7.9161	43	0.0000	0.0000	b2 < b4 *
b2-b3	-0.2977	7.5275	43	0.0000	0.0000	b2 < b3 *
b1-b2	-0.3193	6.7846	43	0.0000	0.0000	b1 < b2 *
b3-b4	-0.1443	6.0798	43	0.0000	0.0000	b3 < b4 *

< MULTIPLE COMPARISON for "C" >

== Holm's Sequentially Rejective Bonferroni Procedure ==
== The factor < C > is analysed as dependent means. ==
== Alpha level is 0.05. ==

C	n	Mean	S.D.
c1	176	-0.0994	0.2855
c2	176	-0.3310	0.5621
c3	176	-0.6037	0.7664
c4	176	-0.8452	0.9094
c5	176	-1.1634	1.1180

Pair	Diff	t-value	df	p	adj.p	
c3-c5	0.5597	10.7618	43	0.0000	0.0000	c3 > c5 *
c2-c5	0.8324	10.0456	43	0.0000	0.0000	c2 > c5 *
c1-c5	1.0639	9.4595	43	0.0000	0.0000	c1 > c5 *
c2-c4	0.5142	8.5067	43	0.0000	0.0000	c2 > c4 *
c1-c4	0.7457	8.4317	43	0.0000	0.0000	c1 > c4 *
c4-c5	0.3182	7.9614	43	0.0000	0.0000	c4 > c5 *
c1-c3	0.5043	7.0234	43	0.0000	0.0000	c1 > c3 *
c3-c4	0.2415	6.7106	43	0.0000	0.0000	c3 > c4 *

```
 c2-c3  0.2727   6.6519  43  0.0000  0.0000  c2 > c3 *
 c1-c2  0.2315   5.8406  43  0.0000  0.0000  c1 > c2 *
---------------------------------------------------------


< SIMPLE EFFECTS for "B x C" INTERACTION >

--------------------------------------------------------------------
------
  Effect  Lambda  approx.Chi  df      p         LB     GG     HF
CM
--------------------------------------------------------------------
------
 B at c1  0.0000     35.5918   5 0.0000 *** 0.3333 0.6323 0.6606
0.6564
 B at c2  0.0000     42.7628   5 0.0000 *** 0.3333 0.6551 0.6862
0.6819
 B at c3  0.0000     52.3698   5 0.0000 *** 0.3333 0.5758 0.5977
0.5940
 B at c4  0.0000     50.4845   5 0.0000 *** 0.3333 0.5563 0.5760
0.5724
 B at c5  0.0000     62.2948   5 0.0000 *** 0.3333 0.5267 0.5434
0.5400
 C at b1  0.0000     81.3738   9 0.0000 *** 0.2500 0.4677 0.4882
0.4851
 C at b2  0.0000     82.2970   9 0.0000 *** 0.2500 0.4524 0.4711
0.4681
 C at b3  0.0000    101.6198   9 0.0000 *** 0.2500 0.4273 0.4432
0.4404
 C at b4  0.0000     71.8027   9 0.0000 *** 0.2500 0.5170 0.5436
0.5401
--------------------------------------------------------------------
------
                               LB = lower.bound, GG = Greenhouse-
Geisser
                              HF = Huynh-Feldt-Lecoutre, CM = Chi-
Muller

-------------------------------------------------------------------
     Source      SS    df      MS  F-ratio  p-value      G.eta^2
-------------------------------------------------------------------
    B at c1  0.5866   1.9  0.3093   6.1048   0.0039 **    0.0411
s x B at c1  4.1321 81.56  0.0507
-------------------------------------------------------------------
    B at c2 11.4244  1.97  5.8128  40.4194   0.0000 ***   0.2066
s x B at c2 12.1538 84.51  0.1438
-------------------------------------------------------------------
    B at c3 15.0266  1.73  8.6984  39.3356   0.0000 ***   0.1462
s x B at c3 16.4265 74.28  0.2211
-------------------------------------------------------------------
    B at c4 24.2653  1.67 14.5408  44.5180   0.0000 ***   0.1677
s x B at c4 23.4379 71.76  0.3266
-------------------------------------------------------------------
    B at c5 42.5323  1.58 26.9168  53.7185   0.0000 ***   0.1944
```

```
s x B at c5 34.0458 67.95  0.5011
--------------------------------------------------------------------
    C at b1 71.0892  1.87 37.9998  74.4013   0.0000 ***   0.2755
s x C at b1 41.0858 80.44  0.5107
--------------------------------------------------------------------
    C at b2 41.9278  1.81 23.1695  61.1210   0.0000 ***   0.2656
s x C at b2 29.4972 77.81  0.3791
--------------------------------------------------------------------
    C at b3 20.2369  1.71 11.8396  40.7333   0.0000 ***   0.2061
s x C at b3 21.3631  73.5  0.2907
--------------------------------------------------------------------
    C at b4  8.5949  2.07  4.1560  23.0195   0.0000 ***   0.1233
s x C at b4 16.0551 88.93  0.1805
--------------------------------------------------------------------
                       +p < .10, *p < .05, **p < .01, ***p < .001


< MULTIPLE COMPARISON for "B at c1" >

== Holm's Sequentially Rejective Bonferroni Procedure ==
== The factor < B at c1 > is analysed as dependent means. ==
== Alpha level is 0.05. ==

-----------------------------------------------------------
  Pair     Diff  t-value  df       p   adj.p
-----------------------------------------------------------
 b1-b4  -0.1477   2.8985  43  0.0059  0.0353  b1 < b4 *
 b1-b3  -0.1307   2.8207  43  0.0072  0.0361  b1 < b3 *
 b2-b4  -0.0739   2.6705  43  0.0106  0.0426  b2 < b4 *
 b2-b3  -0.0568   1.9490  43  0.0578  0.1735  b2 = b3
 b1-b2  -0.0739   1.7626  43  0.0851  0.1735  b1 = b2
 b3-b4  -0.0170   0.6841  43  0.4976  0.4976  b3 = b4
-----------------------------------------------------------


< MULTIPLE COMPARISON for "B at c2" >

== Holm's Sequentially Rejective Bonferroni Procedure ==
== The factor < B at c2 > is analysed as dependent means. ==
== Alpha level is 0.05. ==

-----------------------------------------------------------
  Pair     Diff  t-value  df       p   adj.p
-----------------------------------------------------------
 b1-b4  -0.6420   7.6221  43  0.0000  0.0000  b1 < b4 *
 b1-b3  -0.5909   7.6171  43  0.0000  0.0000  b1 < b3 *
 b2-b3  -0.2614   5.0246  43  0.0000  0.0000  b2 < b3 *
 b2-b4  -0.3125   4.9175  43  0.0000  0.0000  b2 < b4 *
 b1-b2  -0.3295   4.8193  43  0.0000  0.0000  b1 < b2 *
 b3-b4  -0.0511   1.5005  43  0.1408  0.1408  b3 = b4
-----------------------------------------------------------


< MULTIPLE COMPARISON for "B at c3" >
```

== Holm's Sequentially Rejective Bonferroni Procedure ==
== The factor < B at c3 > is analysed as dependent means. ==
== Alpha level is 0.05. ==

Pair	Diff	t-value	df	p	adj.p	
b1-b4	-0.7500	7.3641	43	0.0000	0.0000	b1 < b4 *
b1-b3	-0.6477	6.6106	43	0.0000	0.0000	b1 < b3 *
b2-b4	-0.4034	6.1457	43	0.0000	0.0000	b2 < b4 *
b2-b3	-0.3011	5.9647	43	0.0000	0.0000	b2 < b3 *
b1-b2	-0.3466	4.4647	43	0.0001	0.0001	b1 < b2 *
b3-b4	-0.1023	2.3645	43	0.0226	0.0226	b3 < b4 *

< MULTIPLE COMPARISON for "B at c4" >

== Holm's Sequentially Rejective Bonferroni Procedure ==
== The factor < B at c4 > is analysed as dependent means. ==
== Alpha level is 0.05. ==

Pair	Diff	t-value	df	p	adj.p	
b2-b4	-0.6648	7.8357	43	0.0000	0.0000	b2 < b4 *
b1-b4	-0.9716	7.5718	43	0.0000	0.0000	b1 < b4 *
b1-b3	-0.7045	6.4919	43	0.0000	0.0000	b1 < b3 *
b2-b3	-0.3977	5.8352	43	0.0000	0.0000	b2 < b3 *
b3-b4	-0.2670	4.8761	43	0.0000	0.0000	b3 < b4 *
b1-b2	-0.3068	3.8157	43	0.0004	0.0004	b1 < b2 *

< MULTIPLE COMPARISON for "B at c5" >

== Holm's Sequentially Rejective Bonferroni Procedure ==
== The factor < B at c5 > is analysed as dependent means. ==
== Alpha level is 0.05. ==

Pair	Diff	t-value	df	p	adj.p	
b1-b4	-1.2955	8.3736	43	0.0000	0.0000	b1 < b4 *
b1-b3	-1.0114	8.1875	43	0.0000	0.0000	b1 < b3 *
b2-b4	-0.7557	6.4081	43	0.0000	0.0000	b2 < b4 *
b1-b2	-0.5398	6.2765	43	0.0000	0.0000	b1 < b2 *
b2-b3	-0.4716	5.3819	43	0.0000	0.0000	b2 < b3 *
b3-b4	-0.2841	4.6056	43	0.0000	0.0000	b3 < b4 *

< MULTIPLE COMPARISON for "C at b1" >

== Holm's Sequentially Rejective Bonferroni Procedure ==
== The factor < C at b1 > is analysed as dependent means. ==
== Alpha level is 0.05. ==

Pair	Diff	t-value	df	p	adj.p	
c1-c5	1.6875	10.6484	43	0.0000	0.0000	c1 > c5 *
c3-c5	0.8352	9.9365	43	0.0000	0.0000	c3 > c5 *
c2-c5	1.1534	9.0069	43	0.0000	0.0000	c2 > c5 *
c1-c4	1.1534	8.9175	43	0.0000	0.0000	c1 > c4 *
c1-c3	0.8523	8.3598	43	0.0000	0.0000	c1 > c3 *
c1-c2	0.5341	7.1869	43	0.0000	0.0000	c1 > c2 *
c4-c5	0.5341	6.7921	43	0.0000	0.0000	c4 > c5 *
c2-c4	0.6193	6.1302	43	0.0000	0.0000	c2 > c4 *
c2-c3	0.3182	4.4220	43	0.0001	0.0001	c2 > c3 *
c3-c4	0.3011	3.9588	43	0.0003	0.0003	c3 > c4 *

< MULTIPLE COMPARISON for "C at b2" >

== Holm's Sequentially Rejective Bonferroni Procedure ==
== The factor < C at b2 > is analysed as dependent means. ==
== Alpha level is 0.05. ==

Pair	Diff	t-value	df	p	adj.p	
c2-c4	0.6420	8.9786	43	0.0000	0.0000	c2 > c4 *
c1-c4	0.9205	8.9310	43	0.0000	0.0000	c1 > c4 *
c2-c5	0.9432	8.8257	43	0.0000	0.0000	c2 > c5 *
c1-c5	1.2216	8.6026	43	0.0000	0.0000	c1 > c5 *
c3-c5	0.6420	7.8833	43	0.0000	0.0000	c3 > c5 *
c1-c3	0.5795	6.5982	43	0.0000	0.0000	c1 > c3 *
c3-c4	0.3409	5.6001	43	0.0000	0.0000	c3 > c4 *
c2-c3	0.3011	5.0614	43	0.0000	0.0000	c2 > c3 *
c1-c2	0.2784	4.3425	43	0.0001	0.0002	c1 > c2 *
c4-c5	0.3011	4.2900	43	0.0001	0.0002	c4 > c5 *

< MULTIPLE COMPARISON for "C at b3" >

== Holm's Sequentially Rejective Bonferroni Procedure ==
== The factor < C at b3 > is analysed as dependent means. ==
== Alpha level is 0.05. ==

Pair	Diff	t-value	df	p	adj.p	
c2-c5	0.7330	8.2873	43	0.0000	0.0000	c2 > c5 *
c1-c5	0.8068	7.1108	43	0.0000	0.0000	c1 > c5 *

c3-c5	0.4716	7.0768	43	0.0000	0.0000	c3 > c5 *
c2-c4	0.5057	6.9780	43	0.0000	0.0000	c2 > c4 *
c1-c4	0.5795	5.8067	43	0.0000	0.0000	c1 > c4 *
c2-c3	0.2614	5.0246	43	0.0000	0.0000	c2 > c3 *
c4-c5	0.2273	4.8081	43	0.0000	0.0001	c4 > c5 *
c1-c3	0.3352	4.7326	43	0.0000	0.0001	c1 > c3 *
c3-c4	0.2443	3.8024	43	0.0004	0.0009	c3 > c4 *
c1-c2	0.0739	1.6433	43	0.1076	0.1076	c1 = c2

< MULTIPLE COMPARISON for "C at b4" >

== Holm's Sequentially Rejective Bonferroni Procedure ==
== The factor < C at b4 > is analysed as dependent means. ==
== Alpha level is 0.05. ==

Pair	Diff	t-value	df	p	adj.p	
c2-c5	0.5000	6.0908	43	0.0000	0.0000	c2 > c5 *
c1-c5	0.5398	5.7595	43	0.0000	0.0000	c1 > c5 *
c1-c4	0.3295	4.6576	43	0.0000	0.0002	c1 > c4 *
c2-c4	0.2898	4.6046	43	0.0000	0.0003	c2 > c4 *
c3-c5	0.2898	4.3901	43	0.0001	0.0004	c3 > c5 *
c4-c5	0.2102	4.3141	43	0.0001	0.0005	c4 > c5 *
c1-c3	0.2500	3.7027	43	0.0006	0.0024	c1 > c3 *
c2-c3	0.2102	3.6237	43	0.0008	0.0024	c2 > c3 *
c3-c4	0.0795	1.8244	43	0.0750	0.1501	c3 = c4
c1-c2	0.0398	1.0692	43	0.2909	0.2909	c1 = c2

[[Simple Effects for b1]]

[AsB-Type Design]

This output was generated by anovakun 4.8.5 under R version 4.0.2.
It was executed on Mon Oct 11 18:22:27 2021.

<< DESCRIPTIVE STATISTICS >>

A	C	n	Mean	S.D.
a1	c1	45	-0.0222	0.2601
a1	c2	45	-0.3000	0.5503
a1	c3	45	-0.4778	0.5509
a1	c4	45	-0.5778	0.7108
a1	c5	45	-0.9000	0.7984
a2	c1	41	0.0244	0.3298
a2	c2	41	-0.4207	0.4791

```
 a2  c3  41  -0.5305  0.6782
 a2  c4  41  -0.7134  0.7171
 a2  c5  41  -1.0610  0.8344
 a3  c1  46  -0.0652  0.2496
 a3  c2  46  -0.4185  0.4946
 a3  c3  46  -0.7337  0.7273
 a3  c4  46  -0.9891  0.9617
 a3  c5  46  -1.5652  1.0832
 a4  c1  44   0.0227  0.3316
 a4  c2  44  -0.2784  0.6057
 a4  c3  44  -0.6080  0.8130
 a4  c4  44  -0.7955  0.9265
 a4  c5  44  -1.0341  0.9684
 a5  c1  44  -0.1875  0.3855
 a5  c2  44  -0.7216  0.6630
 a5  c3  44  -1.0398  0.9384
 a5  c4  44  -1.3409  1.1011
 a5  c5  44  -1.8750  1.2912
-------------------------------


<< SPHERICITY INDICES >>

== Mendoza's Multisample Sphericity Test and Epsilons ==

--------------------------------------------------------------------
-----
 Effect  Lambda  approx.Chi  df      p         LB     GG     HF
CM
--------------------------------------------------------------------
-----
      C  0.0000    325.9533  49 0.0000 *** 0.2500 0.5988 0.6060
0.6058
--------------------------------------------------------------------
-----
                              LB = lower.bound, GG = Greenhouse-
Geisser
                             HF = Huynh-Feldt-Lecoutre, CM = Chi-
Muller


<< ANOVA TABLE >>

== Adjusted by Greenhouse-Geisser's Epsilon ==
== This data is UNBALANCED!! ==
== Type III SS is applied. ==

-------------------------------------------------------------------
   Source       SS     df      MS  F-ratio  p-value      G.eta^2
-------------------------------------------------------------------
        A  48.1947      4 12.0487   6.0163   0.0001 ***   0.0737
    s x A 430.5769    215  2.0027
-------------------------------------------------------------------
        C 192.1547    2.4 80.2311 236.2301   0.0000 ***   0.2409
```

```
    A x C  13.3433   9.58  1.3928   4.1010   0.0000 ***   0.0216
s x A x C 174.8857 514.93  0.3396
-------------------------------------------------------------------
    Total 860.4716   1099  0.7830
                       +p < .10, *p < .05, **p < .01, ***p < .001
```

<< POST ANALYSES >>

< MULTIPLE COMPARISON for "A" >

== Holm's Sequentially Rejective Bonferroni Procedure ==
== The factor < A > is analysed as independent means. ==
== Alpha level is 0.05. ==

A	n	Mean	S.D.
a1	225	-0.4556	0.6650
a2	205	-0.5402	0.7220
a3	230	-0.7543	0.9153
a4	220	-0.5386	0.8465
a5	220	-1.0330	1.0855

Pair	Diff	t-value	df	p	adj.p	
a1-a5	0.5774	4.3032	215	0.0000	0.0003	a1 > a5 *
a4-a5	0.4943	3.6635	215	0.0003	0.0028	a4 > a5 *
a2-a5	0.4927	3.5866	215	0.0004	0.0033	a2 > a5 *
a1-a3	0.2988	2.2517	215	0.0254	0.1775	a1 = a3
a3-a5	0.2786	2.0876	215	0.0380	0.2281	a3 = a5
a3-a4	-0.2157	1.6164	215	0.1075	0.5374	a3 = a4
a2-a3	0.2141	1.5751	215	0.1167	0.5374	a2 = a3
a1-a2	0.0847	0.6198	215	0.5360	1.0000	a1 = a2
a1-a4	0.0831	0.6192	215	0.5365	1.0000	a1 = a4
a2-a4	-0.0016	0.0117	215	0.9907	1.0000	a2 = a4

< MULTIPLE COMPARISON for "C" >

== Holm's Sequentially Rejective Bonferroni Procedure ==
== The factor < C > is analysed as dependent means. ==
== Alpha level is 0.05. ==

C	n	Mean	S.D.
c1	220	-0.0456	0.3211
c2	220	-0.4278	0.5802
c3	220	-0.6779	0.7724
c4	220	-0.8833	0.9288

```
 c5 220  -1.2871  1.0709
---------------------------

---------------------------------------------------------
  Pair    Diff  t-value  df       p   adj.p
---------------------------------------------------------
 c1-c5  1.2415  20.0876 215  0.0000  0.0000  c1 > c5 *
 c2-c5  0.8592  18.1716 215  0.0000  0.0000  c2 > c5 *
 c3-c5  0.6091  17.1506 215  0.0000  0.0000  c3 > c5 *
 c1-c4  0.8378  15.4408 215  0.0000  0.0000  c1 > c4 *
 c1-c3  0.6324  14.7801 215  0.0000  0.0000  c1 > c3 *
 c1-c2  0.3823  11.7566 215  0.0000  0.0000  c1 > c2 *
 c2-c4  0.4555  11.5398 215  0.0000  0.0000  c2 > c4 *
 c4-c5  0.4037   9.8876 215  0.0000  0.0000  c4 > c5 *
 c2-c3  0.2501   8.3961 215  0.0000  0.0000  c2 > c3 *
 c3-c4  0.2054   5.8015 215  0.0000  0.0000  c3 > c4 *
---------------------------------------------------------


< SIMPLE EFFECTS for "A x C" INTERACTION >

--------------------------------------------------------------------
------
  Effect  Lambda  approx.Chi  df      p         LB     GG     HF
CM
--------------------------------------------------------------------
------
 C at a1  0.0000     38.8624   9 0.0000 *** 0.2500 0.6910 0.7419
0.7374
 C at a2  0.0000     68.4244   9 0.0000 *** 0.2500 0.5054 0.5324
0.5285
 C at a3  0.0000     72.0489   9 0.0000 *** 0.2500 0.5400 0.5682
0.5649
 C at a4  0.0000     50.5430   9 0.0000 *** 0.2500 0.6845 0.7357
0.7310
 C at a5  0.0000     81.3738   9 0.0000 *** 0.2500 0.4677 0.4882
0.4851
--------------------------------------------------------------------
------
                               LB = lower.bound, GG = Greenhouse-
Geisser
                              HF = Huynh-Feldt-Lecoutre, CM = Chi-
Muller

--------------------------------------------------------------------
-
     Source        SS     df       MS  F-ratio  p-value      G.eta^2
--------------------------------------------------------------------
-
    A at c1   1.3343      4  0.3336   3.3749   0.0106 *     0.0591
   Er at c1  21.2506    215  0.0988
--------------------------------------------------------------------
-
    A at c2   5.5207      4  1.3802   4.3516   0.0021 **    0.0749
```

```
   Er at c2  68.1906    215  0.3172
--------------------------------------------------------------------
-
    A at c3   8.8129      4  2.2032   3.8877   0.0045 **    0.0675
   Er at c3 121.8451    215  0.5667
--------------------------------------------------------------------
-
    A at c4  15.4516      4  3.8629   4.7879   0.0010 **    0.0818
   Er at c4 173.4629    215  0.8068
--------------------------------------------------------------------
-
    A at c5  30.4185      4  7.6046   7.4078   0.0000 ***   0.1211
   Er at c5 220.7133    215  1.0266
--------------------------------------------------------------------
-
    C at a1  19.1222   2.76  6.9186  31.0440   0.0000 ***   0.1930
s x C at a1  27.1028 121.61  0.2229
--------------------------------------------------------------------
-
    C at a2  26.0079   2.02 12.8654  38.3639   0.0000 ***   0.2445
s x C at a2  27.1171  80.86  0.3354
--------------------------------------------------------------------
-
    C at a3  59.8353   2.16 27.6992  61.2444   0.0000 ***   0.3119
s x C at a3  43.9647  97.21  0.4523
--------------------------------------------------------------------
-
    C at a4  30.7597   2.74 11.2340  37.1375   0.0000 ***   0.1960
s x C at a4  35.6153 117.74  0.3025
--------------------------------------------------------------------
-
    C at a5  71.0892   1.87 37.9998  74.4013   0.0000 ***   0.2755
s x C at a5  41.0858  80.44  0.5107
--------------------------------------------------------------------
-
                         +p < .10, *p < .05, **p < .01, ***p < .001


< MULTIPLE COMPARISON for "A at c1" >

== Holm's Sequentially Rejective Bonferroni Procedure ==
== The factor < A at c1 > is analysed as independent means. ==
== Alpha level is 0.05. ==

----------------------------------------------------------
  Pair     Diff  t-value  df       p   adj.p
----------------------------------------------------------
 a4-a5   0.2102   3.1364 215  0.0019  0.0195  a4 > a5 *
 a2-a5   0.2119   3.1049 215  0.0022  0.0195  a2 > a5 *
 a1-a5   0.1653   2.4796 215  0.0139  0.1114  a1 = a5
 a3-a5   0.1223   1.8445 215  0.0665  0.4654  a3 = a5
 a2-a3   0.0896   1.3271 215  0.1859  1.0000  a2 = a3
 a3-a4  -0.0879   1.3266 215  0.1861  1.0000  a3 = a4
 a1-a2  -0.0466   0.6867 215  0.4930  1.0000  a1 = a2
```

```
 a1-a4  -0.0449   0.6744 215  0.5008  1.0000  a1 = a4
 a1-a3   0.0430   0.6523 215  0.5149  1.0000  a1 = a3
 a2-a4   0.0017   0.0244 215  0.9806  1.0000  a2 = a4
----------------------------------------------------------


< MULTIPLE COMPARISON for "A at c2" >

== Holm's Sequentially Rejective Bonferroni Procedure ==
== The factor < A at c2 > is analysed as independent means. ==
== Alpha level is 0.05. ==

----------------------------------------------------------
  Pair     Diff  t-value  df       p   adj.p
----------------------------------------------------------
 a4-a5   0.4432   3.6911 215  0.0003  0.0028  a4 > a5 *
 a1-a5   0.4216   3.5309 215  0.0005  0.0046  a1 > a5 *
 a3-a5   0.3031   2.5524 215  0.0114  0.0911  a3 = a5
 a2-a5   0.3009   2.4611 215  0.0146  0.1025  a2 = a5
 a3-a4  -0.1401   1.1795 215  0.2395  1.0000  a3 = a4
 a2-a4  -0.1423   1.1642 215  0.2456  1.0000  a2 = a4
 a1-a3   0.1185   1.0034 215  0.3168  1.0000  a1 = a3
 a1-a2   0.1207   0.9929 215  0.3219  1.0000  a1 = a2
 a1-a4  -0.0216   0.1808 215  0.8567  1.0000  a1 = a4
 a2-a3  -0.0023   0.0186 215  0.9852  1.0000  a2 = a3
----------------------------------------------------------


< MULTIPLE COMPARISON for "A at c3" >

== Holm's Sequentially Rejective Bonferroni Procedure ==
== The factor < A at c3 > is analysed as independent means. ==
== Alpha level is 0.05. ==

----------------------------------------------------------
  Pair     Diff  t-value  df       p   adj.p
----------------------------------------------------------
 a1-a5   0.5620   3.5212 215  0.0005  0.0052  a1 > a5 *
 a2-a5   0.5093   3.1166 215  0.0021  0.0187  a2 > a5 *
 a4-a5   0.4318   2.6905 215  0.0077  0.0616  a4 = a5
 a3-a5   0.3061   1.9281 215  0.0552  0.3861  a3 = a5
 a1-a3   0.2559   1.6214 215  0.1064  0.6384  a1 = a3
 a2-a3   0.2032   1.2568 215  0.2102  1.0000  a2 = a3
 a1-a4   0.1302   0.8156 215  0.4156  1.0000  a1 = a4
 a3-a4  -0.1257   0.7921 215  0.4292  1.0000  a3 = a4
 a2-a4   0.0775   0.4741 215  0.6359  1.0000  a2 = a4
 a1-a2   0.0527   0.3243 215  0.7460  1.0000  a1 = a2
----------------------------------------------------------


< MULTIPLE COMPARISON for "A at c4" >

== Holm's Sequentially Rejective Bonferroni Procedure ==
== The factor < A at c4 > is analysed as independent means. ==
```

== Alpha level is 0.05. ==

Pair	Diff	t-value	df	p	adj.p	
a1-a5	0.7631	4.0073	215	0.0001	0.0008	a1 > a5 *
a2-a5	0.6275	3.2184	215	0.0015	0.0134	a2 > a5 *
a4-a5	0.5455	2.8483	215	0.0048	0.0386	a4 > a5 *
a1-a3	0.4114	2.1842	215	0.0300	0.2102	a1 = a3
a3-a5	0.3518	1.8572	215	0.0646	0.3879	a3 = a5
a2-a3	0.2757	1.4292	215	0.1544	0.7720	a2 = a3
a1-a4	0.2177	1.1431	215	0.2543	1.0000	a1 = a4
a3-a4	-0.1937	1.0225	215	0.3077	1.0000	a3 = a4
a1-a2	0.1356	0.6994	215	0.4850	1.0000	a1 = a2
a2-a4	0.0820	0.4208	215	0.6743	1.0000	a2 = a4

< MULTIPLE COMPARISON for "A at c5" >

== Holm's Sequentially Rejective Bonferroni Procedure ==
== The factor < A at c5 > is analysed as independent means. ==
== Alpha level is 0.05. ==

Pair	Diff	t-value	df	p	adj.p	
a1-a5	0.9750	4.5389	215	0.0000	0.0001	a1 > a5 *
a4-a5	0.8409	3.8928	215	0.0001	0.0012	a4 > a5 *
a2-a5	0.8140	3.7013	215	0.0003	0.0022	a2 > a5 *
a1-a3	0.6652	3.1314	215	0.0020	0.0139	a1 > a3 *
a3-a4	-0.5311	2.4859	215	0.0137	0.0821	a3 = a4
a2-a3	0.5042	2.3172	215	0.0214	0.1072	a2 = a3
a3-a5	0.3098	1.4499	215	0.1485	0.5941	a3 = a5
a1-a2	0.1610	0.7359	215	0.4626	1.0000	a1 = a2
a1-a4	0.1341	0.6242	215	0.5331	1.0000	a1 = a4
a2-a4	-0.0269	0.1222	215	0.9028	1.0000	a2 = a4

< MULTIPLE COMPARISON for "C at a1" >

== Holm's Sequentially Rejective Bonferroni Procedure ==
== The factor < C at a1 > is analysed as dependent means. ==
== Alpha level is 0.05. ==

Pair	Diff	t-value	df	p	adj.p	
c1-c5	0.8778	8.0016	44	0.0000	0.0000	c1 > c5 *
c2-c5	0.6000	7.2529	44	0.0000	0.0000	c2 > c5 *
c1-c3	0.4556	6.5217	44	0.0000	0.0000	c1 > c3 *
c3-c5	0.4222	6.0237	44	0.0000	0.0000	c3 > c5 *
c1-c4	0.5556	5.1844	44	0.0000	0.0000	c1 > c4 *

c4-c5	0.3222	3.9839	44	0.0003	0.0013	c4 > c5 *
c1-c2	0.2778	3.7650	44	0.0005	0.0020	c1 > c2 *
c2-c3	0.1778	3.3877	44	0.0015	0.0045	c2 > c3 *
c2-c4	0.2778	3.2856	44	0.0020	0.0045	c2 > c4 *
c3-c4	0.1000	1.2499	44	0.2180	0.2180	c3 = c4

< MULTIPLE COMPARISON for "C at a2" >

== Holm's Sequentially Rejective Bonferroni Procedure ==
== The factor < C at a2 > is analysed as dependent means. ==
== Alpha level is 0.05. ==

Pair	Diff	t-value	df	p	adj.p	
c1-c5	1.0854	7.7912	40	0.0000	0.0000	c1 > c5 *
c2-c5	0.6402	6.8670	40	0.0000	0.0000	c2 > c5 *
c3-c5	0.5305	6.6618	40	0.0000	0.0000	c3 > c5 *
c1-c4	0.7378	6.2824	40	0.0000	0.0000	c1 > c4 *
c1-c2	0.4451	5.4502	40	0.0000	0.0000	c1 > c2 *
c4-c5	0.3476	5.0205	40	0.0000	0.0001	c4 > c5 *
c1-c3	0.5549	4.9704	40	0.0000	0.0001	c1 > c3 *
c2-c4	0.2927	4.5663	40	0.0000	0.0001	c2 > c4 *
c3-c4	0.1829	3.3979	40	0.0015	0.0031	c3 > c4 *
c2-c3	0.1098	1.8526	40	0.0713	0.0713	c2 = c3

< MULTIPLE COMPARISON for "C at a3" >

== Holm's Sequentially Rejective Bonferroni Procedure ==
== The factor < C at a3 > is analysed as dependent means. ==
== Alpha level is 0.05. ==

Pair	Diff	t-value	df	p	adj.p	
c1-c5	1.5000	9.7619	45	0.0000	0.0000	c1 > c5 *
c2-c5	1.1467	9.6556	45	0.0000	0.0000	c2 > c5 *
c3-c5	0.8315	9.0418	45	0.0000	0.0000	c3 > c5 *
c1-c4	0.9239	7.1713	45	0.0000	0.0000	c1 > c4 *
c1-c3	0.6685	6.9534	45	0.0000	0.0000	c1 > c3 *
c2-c4	0.5707	6.1482	45	0.0000	0.0000	c2 > c4 *
c4-c5	0.5761	5.7218	45	0.0000	0.0000	c4 > c5 *
c1-c2	0.3533	5.1284	45	0.0000	0.0000	c1 > c2 *
c2-c3	0.3152	4.4409	45	0.0001	0.0001	c2 > c3 *
c3-c4	0.2554	3.3996	45	0.0014	0.0014	c3 > c4 *

< MULTIPLE COMPARISON for "C at a4" >

== Holm's Sequentially Rejective Bonferroni Procedure ==
== The factor < C at a4 > is analysed as dependent means. ==
== Alpha level is 0.05. ==

Pair	Diff	t-value	df	p	adj.p	
c1-c5	1.0568	8.6322	43	0.0000	0.0000	c1 > c5 *
c2-c5	0.7557	7.8438	43	0.0000	0.0000	c2 > c5 *
c1-c4	0.8182	6.7655	43	0.0000	0.0000	c1 > c4 *
c1-c3	0.6307	6.5463	43	0.0000	0.0000	c1 > c3 *
c3-c5	0.4261	6.3308	43	0.0000	0.0000	c3 > c5 *
c2-c4	0.5170	5.7033	43	0.0000	0.0000	c2 > c4 *
c1-c2	0.3011	4.6463	43	0.0000	0.0001	c1 > c2 *
c2-c3	0.3295	4.4297	43	0.0001	0.0002	c2 > c3 *
c4-c5	0.2386	2.0659	43	0.0449	0.0898	c4 = c5
c3-c4	0.1875	1.8642	43	0.0691	0.0898	c3 = c4

< MULTIPLE COMPARISON for "C at a5" >

== Holm's Sequentially Rejective Bonferroni Procedure ==
== The factor < C at a5 > is analysed as dependent means. ==
== Alpha level is 0.05. ==

Pair	Diff	t-value	df	p	adj.p	
c1-c5	1.6875	10.6484	43	0.0000	0.0000	c1 > c5 *
c3-c5	0.8352	9.9365	43	0.0000	0.0000	c3 > c5 *
c2-c5	1.1534	9.0069	43	0.0000	0.0000	c2 > c5 *
c1-c4	1.1534	8.9175	43	0.0000	0.0000	c1 > c4 *
c1-c3	0.8523	8.3598	43	0.0000	0.0000	c1 > c3 *
c1-c2	0.5341	7.1869	43	0.0000	0.0000	c1 > c2 *
c4-c5	0.5341	6.7921	43	0.0000	0.0000	c4 > c5 *
c2-c4	0.6193	6.1302	43	0.0000	0.0000	c2 > c4 *
c2-c3	0.3182	4.4220	43	0.0001	0.0001	c2 > c3 *
c3-c4	0.3011	3.9588	43	0.0003	0.0003	c3 > c4 *

output is over ---------------------///

[[Simple Effects for b2]]

[AsB-Type Design]

This output was generated by anovakun 4.8.5 under R version 4.0.2.
It was executed on Mon Oct 11 18:22:28 2021.

<< DESCRIPTIVE STATISTICS >>

A	C	n	Mean	S.D.
a1	c1	45	-0.0111	0.1406
a1	c2	45	-0.0944	0.2516
a1	c3	45	-0.2722	0.4018
a1	c4	45	-0.4000	0.4689
a1	c5	45	-0.5278	0.5671
a2	c1	41	-0.0305	0.1695
a2	c2	41	-0.1280	0.2569
a2	c3	41	-0.2378	0.3160
a2	c4	41	-0.5305	0.6713
a2	c5	41	-0.6585	0.6040
a3	c1	46	0.0000	0.0527
a3	c2	46	-0.1576	0.3389
a3	c3	46	-0.3152	0.4231
a3	c4	46	-0.6250	0.5980
a3	c5	46	-0.9402	0.6671
a4	c1	44	0.0455	0.3015
a4	c2	44	-0.2045	0.3707
a4	c3	44	-0.3409	0.4944
a4	c4	44	-0.4602	0.6122
a4	c5	44	-0.8068	0.8758
a5	c1	44	-0.1136	0.2825
a5	c2	44	-0.3920	0.5665
a5	c3	44	-0.6932	0.7089
a5	c4	44	-1.0341	0.8134
a5	c5	44	-1.3352	1.0633

<< SPHERICITY INDICES >>

== Mendoza's Multisample Sphericity Test and Epsilons ==

Effect	Lambda	approx.Chi	df	p		LB	GG	HF	CM
C	0.0000	357.6419	49	0.0000	***	0.2500	0.5736	0.5801	0.5800

LB = lower.bound, GG = Greenhouse-Geisser
HF = Huynh-Feldt-Lecoutre, CM = Chi-Muller

<< ANOVA TABLE >>

== Adjusted by Greenhouse-Geisser's Epsilon ==

== This data is UNBALANCED!! ==
== Type III SS is applied. ==

Source	SS	df	MS	F-ratio	p-value		G.eta^2
A	27.7454	4	6.9363	7.5709	0.0000	***	0.0822
s x A	196.9807	215	0.9162				
C	95.5524	2.29	41.6482	182.1085	0.0000	***	0.2357
A x C	9.3250	9.18	1.0161	4.4430	0.0000	***	0.0292
s x A x C	112.8105	493.27	0.2287				
Total	443.0011	1099	0.4031				

+p < .10, *p < .05, **p < .01, ***p < .001

<< POST ANALYSES >>

< MULTIPLE COMPARISON for "A" >

== Holm's Sequentially Rejective Bonferroni Procedure ==
== The factor < A > is analysed as independent means. ==
== Alpha level is 0.05. ==

A	n	Mean	S.D.
a1	225	-0.2611	0.4367
a2	205	-0.3171	0.5056
a3	230	-0.4076	0.5746
a4	220	-0.3534	0.6301
a5	220	-0.7136	0.8489

Pair	Diff	t-value	df	p	adj.p		
a1-a5	0.4525	4.9862	215	0.0000	0.0000	a1 > a5	*
a2-a5	0.3966	4.2679	215	0.0000	0.0003	a2 > a5	*
a4-a5	0.3602	3.9471	215	0.0001	0.0009	a4 > a5	*
a3-a5	0.3060	3.3903	215	0.0008	0.0058	a3 > a5	*
a1-a3	0.1465	1.6323	215	0.1041	0.6245	a1 = a3	
a1-a4	0.0923	1.0170	215	0.3103	1.0000	a1 = a4	
a2-a3	0.0905	0.9847	215	0.3259	1.0000	a2 = a3	
a1-a2	0.0560	0.6055	215	0.5455	1.0000	a1 = a2	
a3-a4	-0.0542	0.6004	215	0.5488	1.0000	a3 = a4	
a2-a4	0.0363	0.3911	215	0.6961	1.0000	a2 = a4	

< MULTIPLE COMPARISON for "C" >

== Holm's Sequentially Rejective Bonferroni Procedure ==

== The factor < C > is analysed as dependent means. ==
== Alpha level is 0.05. ==

C	n	Mean	S.D.
c1	220	-0.0220	0.2146
c2	220	-0.1953	0.3865
c3	220	-0.3719	0.5110
c4	220	-0.6100	0.6741
c5	220	-0.8537	0.8203

Pair	Diff	t-value	df	p	adj.p	
c2-c5	0.6584	16.4073	215	0.0000	0.0000	c2 > c5 *
c1-c5	0.8318	16.2390	215	0.0000	0.0000	c1 > c5 *
c1-c4	0.5880	14.7609	215	0.0000	0.0000	c1 > c4 *
c2-c4	0.4146	13.7962	215	0.0000	0.0000	c2 > c4 *
c3-c5	0.4818	13.5236	215	0.0000	0.0000	c3 > c5 *
c1-c3	0.3499	11.3278	215	0.0000	0.0000	c1 > c3 *
c3-c4	0.2381	8.1021	215	0.0000	0.0000	c3 > c4 *
c2-c3	0.1765	7.6715	215	0.0000	0.0000	c2 > c3 *
c4-c5	0.2438	7.4918	215	0.0000	0.0000	c4 > c5 *
c1-c2	0.1734	7.4353	215	0.0000	0.0000	c1 > c2 *

< SIMPLE EFFECTS for "A x C" INTERACTION >

Effect	Lambda	approx.Chi	df	p		LB	GG	HF	CM
C at a1	0.0000	61.5209	9	0.0000	***	0.2500	0.5984	0.6352	0.6313
C at a2	0.0000	60.8486	9	0.0000	***	0.2500	0.5910	0.6306	0.6260
C at a3	0.0000	49.3586	9	0.0000	***	0.2500	0.6155	0.6538	0.6500
C at a4	0.0000	72.3410	9	0.0000	***	0.2500	0.5182	0.5449	0.5415
C at a5	0.0000	82.2970	9	0.0000	***	0.2500	0.4524	0.4711	0.4681

LB = lower.bound, GG = Greenhouse-Geisser
HF = Huynh-Feldt-Lecoutre, CM = Chi-Muller

Source	SS	df	MS	F-ratio	p-value		G.eta^2
A at c1	0.6002	4	0.1500	3.4013	0.0101	*	0.0595
Er at c1	9.4847	215	0.0441				
A at c2	2.4155	4	0.6039	4.2841	0.0023	**	0.0738
Er at c2	30.3050	215	0.1410				
A at c3	5.9160	4	1.4790	6.2021	0.0001	***	0.1035
Er at c3	51.2703	215	0.2385				
A at c4	11.1546	4	2.7886	6.7854	0.0000	***	0.1121
Er at c4	88.3599	215	0.4110				
A at c5	16.9842	4	4.2460	7.0023	0.0000	***	0.1153
Er at c5	130.3712	215	0.6064				
C at a1	8.1361	2.39	3.3991	23.1500	0.0000	***	0.1904
s x C at a1	15.4639	105.32	0.1468				
C at a2	11.7378	2.36	4.9650	27.2383	0.0000	***	0.2251
s x C at a2	17.2372	94.56	0.1823				
C at a3	26.1332	2.46	10.6151	57.0403	0.0000	***	0.3457
s x C at a3	20.6168	110.78	0.1861				
C at a4	17.5295	2.07	8.4564	25.1295	0.0000	***	0.2016
s x C at a4	29.9955	89.14	0.3365				
C at a5	41.9278	1.81	23.1695	61.1210	0.0000	***	0.2656
s x C at a5	29.4972	77.81	0.3791				

+p < .10, *p < .05, **p < .01, ***p < .001

< MULTIPLE COMPARISON for "A at c1" >

== Holm's Sequentially Rejective Bonferroni Procedure ==
== The factor < A at c1 > is analysed as independent means. ==
== Alpha level is 0.05. ==

Pair	Diff	t-value	df	p	adj.p	
a4-a5	0.1591	3.5527	215	0.0005	0.0047	a4 > a5 *
a3-a5	0.1136	2.5657	215	0.0110	0.0988	a3 = a5
a1-a5	0.1025	2.3024	215	0.0223	0.1782	a1 = a5
a2-a5	0.0831	1.8238	215	0.0696	0.4870	a2 = a5
a2-a4	-0.0759	1.6657	215	0.0972	0.5834	a2 = a4
a1-a4	-0.0566	1.2703	215	0.2054	1.0000	a1 = a4
a3-a4	-0.0455	1.0263	215	0.3059	1.0000	a3 = a4
a2-a3	-0.0305	0.6758	215	0.4999	1.0000	a2 = a3
a1-a2	0.0194	0.4273	215	0.6696	1.0000	a1 = a2
a1-a3	-0.0111	0.2523	215	0.8010	1.0000	a1 = a3

< MULTIPLE COMPARISON for "A at c2" >

== Holm's Sequentially Rejective Bonferroni Procedure ==
== The factor < A at c2 > is analysed as independent means. ==
== Alpha level is 0.05. ==

Pair	Diff	t-value	df	p	adj.p	
a1-a5	0.2976	3.7388	215	0.0002	0.0024	a1 > a5 *
a2-a5	0.2640	3.2394	215	0.0014	0.0125	a2 > a5 *
a3-a5	0.2344	2.9612	215	0.0034	0.0273	a3 > a5 *
a4-a5	0.1875	2.3425	215	0.0201	0.1405	a4 = a5
a1-a4	0.1101	1.3832	215	0.1680	1.0000	a1 = a4
a2-a4	0.0765	0.9387	215	0.3490	1.0000	a2 = a4
a1-a3	0.0632	0.8024	215	0.4232	1.0000	a1 = a3
a3-a4	0.0469	0.5929	215	0.5539	1.0000	a3 = a4
a1-a2	0.0336	0.4146	215	0.6789	1.0000	a1 = a2
a2-a3	0.0296	0.3666	215	0.7143	1.0000	a2 = a3

< MULTIPLE COMPARISON for "A at c3" >

== Holm's Sequentially Rejective Bonferroni Procedure ==
== The factor < A at c3 > is analysed as independent means. ==
== Alpha level is 0.05. ==

Pair	Diff	t-value	df	p	adj.p	
a2-a5	0.4554	4.2960	215	0.0000	0.0003	a2 > a5 *
a1-a5	0.4210	4.0660	215	0.0001	0.0006	a1 > a5 *
a3-a5	0.3780	3.6705	215	0.0003	0.0024	a3 > a5 *
a4-a5	0.3523	3.3836	215	0.0009	0.0060	a4 > a5 *
a2-a4	0.1031	0.9727	215	0.3318	1.0000	a2 = a4
a2-a3	0.0774	0.7381	215	0.4613	1.0000	a2 = a3
a1-a4	0.0687	0.6634	215	0.5078	1.0000	a1 = a4

a1-a3	0.0430	0.4199	215	0.6750	1.0000	a1 = a3
a1-a2	-0.0344	0.3264	215	0.7444	1.0000	a1 = a2
a3-a4	0.0257	0.2495	215	0.8032	1.0000	a3 = a4

< MULTIPLE COMPARISON for "A at c4" >

== Holm's Sequentially Rejective Bonferroni Procedure ==
== The factor < A at c4 > is analysed as independent means. ==
== Alpha level is 0.05. ==

Pair	Diff	t-value	df	p	adj.p	
a1-a5	0.6341	4.6653	215	0.0000	0.0001	a1 > a5 *
a4-a5	0.5739	4.1987	215	0.0000	0.0004	a4 > a5 *
a2-a5	0.5036	3.6190	215	0.0004	0.0029	a2 > a5 *
a3-a5	0.4091	3.0262	215	0.0028	0.0195	a3 > a5 *
a1-a3	0.2250	1.6739	215	0.0956	0.5736	a1 = a3
a3-a4	-0.1648	1.2189	215	0.2242	1.0000	a3 = a4
a1-a2	0.1305	0.9428	215	0.3469	1.0000	a1 = a2
a2-a3	0.0945	0.6864	215	0.4932	1.0000	a2 = a3
a2-a4	-0.0703	0.5049	215	0.6141	1.0000	a2 = a4
a1-a4	0.0602	0.4431	215	0.6581	1.0000	a1 = a4

< MULTIPLE COMPARISON for "A at c5" >

== Holm's Sequentially Rejective Bonferroni Procedure ==
== The factor < A at c5 > is analysed as independent means. ==
== Alpha level is 0.05. ==

Pair	Diff	t-value	df	p	adj.p	
a1-a5	0.8074	4.8908	215	0.0000	0.0000	a1 > a5 *
a2-a5	0.6767	4.0034	215	0.0001	0.0008	a2 > a5 *
a4-a5	0.5284	3.1828	215	0.0017	0.0134	a4 > a5 *
a1-a3	0.4124	2.5261	215	0.0123	0.0858	a1 = a3
a3-a5	0.3950	2.4056	215	0.0170	0.1020	a3 = a5
a1-a4	0.2790	1.6902	215	0.0924	0.4622	a1 = a4
a2-a3	0.2817	1.6842	215	0.0936	0.4622	a2 = a3
a2-a4	0.1483	0.8773	215	0.3813	1.0000	a2 = a4
a3-a4	-0.1334	0.8124	215	0.4175	1.0000	a3 = a4
a1-a2	0.1308	0.7778	215	0.4376	1.0000	a1 = a2

< MULTIPLE COMPARISON for "C at a1" >

== Holm's Sequentially Rejective Bonferroni Procedure ==
== The factor < C at a1 > is analysed as dependent means. ==

== Alpha level is 0.05. ==

Pair	Diff	t-value	df	p	adj.p	
c1-c5	0.5167	6.0014	44	0.0000	0.0000	c1 > c5 *
c2-c5	0.4333	5.9720	44	0.0000	0.0000	c2 > c5 *
c1-c4	0.3889	5.5939	44	0.0000	0.0000	c1 > c4 *
c2-c4	0.3056	5.3386	44	0.0000	0.0000	c2 > c4 *
c1-c3	0.2611	4.6968	44	0.0000	0.0002	c1 > c3 *
c3-c5	0.2556	3.7019	44	0.0006	0.0030	c3 > c5 *
c2-c3	0.1778	3.4272	44	0.0013	0.0053	c2 > c3 *
c1-c2	0.0833	2.6220	44	0.0120	0.0359	c1 > c2 *
c4-c5	0.1278	2.3809	44	0.0217	0.0433	c4 > c5 *
c3-c4	0.1278	2.0655	44	0.0448	0.0448	c3 > c4 *

< MULTIPLE COMPARISON for "C at a2" >

== Holm's Sequentially Rejective Bonferroni Procedure ==
== The factor < C at a2 > is analysed as dependent means. ==
== Alpha level is 0.05. ==

Pair	Diff	t-value	df	p	adj.p	
c1-c5	0.6280	7.0164	40	0.0000	0.0000	c1 > c5 *
c2-c5	0.5305	6.5449	40	0.0000	0.0000	c2 > c5 *
c3-c5	0.4207	5.8253	40	0.0000	0.0000	c3 > c5 *
c1-c4	0.5000	5.5627	40	0.0000	0.0000	c1 > c4 *
c2-c4	0.4024	4.7831	40	0.0000	0.0001	c2 > c4 *
c1-c3	0.2073	4.2717	40	0.0001	0.0006	c1 > c3 *
c3-c4	0.2927	3.5962	40	0.0009	0.0035	c3 > c4 *
c1-c2	0.0976	2.8044	40	0.0077	0.0232	c1 > c2 *
c2-c3	0.1098	2.5119	40	0.0161	0.0323	c2 > c3 *
c4-c5	0.1280	1.7271	40	0.0919	0.0919	c4 = c5

< MULTIPLE COMPARISON for "C at a3" >

== Holm's Sequentially Rejective Bonferroni Procedure ==
== The factor < C at a3 > is analysed as dependent means. ==
== Alpha level is 0.05. ==

Pair	Diff	t-value	df	p	adj.p	
c2-c5	0.7826	10.1419	45	0.0000	0.0000	c2 > c5 *
c1-c5	0.9402	9.5007	45	0.0000	0.0000	c1 > c5 *
c3-c5	0.6250	9.4951	45	0.0000	0.0000	c3 > c5 *
c2-c4	0.4674	7.1077	45	0.0000	0.0000	c2 > c4 *
c1-c4	0.6250	7.0882	45	0.0000	0.0000	c1 > c4 *

c1-c3	0.3152	5.0534	45	0.0000	0.0000	c1 > c3 *
c4-c5	0.3152	4.9035	45	0.0000	0.0001	c4 > c5 *
c3-c4	0.3098	4.7585	45	0.0000	0.0001	c3 > c4 *
c1-c2	0.1576	3.1537	45	0.0029	0.0057	c1 > c2 *
c2-c3	0.1576	2.9472	45	0.0051	0.0057	c2 > c3 *

< MULTIPLE COMPARISON for "C at a4" >

== Holm's Sequentially Rejective Bonferroni Procedure ==
== The factor < C at a4 > is analysed as dependent means. ==
== Alpha level is 0.05. ==

Pair	Diff	t-value	df	p	adj.p	
c1-c5	0.8523	6.0405	43	0.0000	0.0000	c1 > c5 *
c2-c5	0.6023	5.7283	43	0.0000	0.0000	c2 > c5 *
c1-c4	0.5057	5.5099	43	0.0000	0.0000	c1 > c4 *
c1-c3	0.3864	4.7664	43	0.0000	0.0002	c1 > c3 *
c2-c4	0.2557	4.5647	43	0.0000	0.0002	c2 > c4 *
c3-c5	0.4659	4.4815	43	0.0001	0.0003	c3 > c5 *
c1-c2	0.2500	3.6762	43	0.0007	0.0026	c1 > c2 *
c4-c5	0.3466	3.6139	43	0.0008	0.0026	c4 > c5 *
c2-c3	0.1364	2.9913	43	0.0046	0.0092	c2 > c3 *
c3-c4	0.1193	2.0262	43	0.0490	0.0490	c3 > c4 *

< MULTIPLE COMPARISON for "C at a5" >

== Holm's Sequentially Rejective Bonferroni Procedure ==
== The factor < C at a5 > is analysed as dependent means. ==
== Alpha level is 0.05. ==

Pair	Diff	t-value	df	p	adj.p	
c2-c4	0.6420	8.9786	43	0.0000	0.0000	c2 > c4 *
c1-c4	0.9205	8.9310	43	0.0000	0.0000	c1 > c4 *
c2-c5	0.9432	8.8257	43	0.0000	0.0000	c2 > c5 *
c1-c5	1.2216	8.6026	43	0.0000	0.0000	c1 > c5 *
c3-c5	0.6420	7.8833	43	0.0000	0.0000	c3 > c5 *
c1-c3	0.5795	6.5982	43	0.0000	0.0000	c1 > c3 *
c3-c4	0.3409	5.6001	43	0.0000	0.0000	c3 > c4 *
c2-c3	0.3011	5.0614	43	0.0000	0.0000	c2 > c3 *
c1-c2	0.2784	4.3425	43	0.0001	0.0002	c1 > c2 *
c4-c5	0.3011	4.2900	43	0.0001	0.0002	c4 > c5 *

output is over --------------------///

[[Simple Effects for b3]]

[AsB-Type Design]

This output was generated by anovakun 4.8.5 under R version 4.0.2.
It was executed on Mon Oct 11 18:22:29 2021.

<< DESCRIPTIVE STATISTICS >>

A	C	n	Mean	S.D.
a1	c1	45	-0.0111	0.1187
a1	c2	45	-0.0333	0.1735
a1	c3	45	-0.0722	0.2038
a1	c4	45	-0.1444	0.2743
a1	c5	45	-0.2278	0.3946
a2	c1	41	0.0488	0.2033
a2	c2	41	-0.0061	0.1044
a2	c3	41	-0.0183	0.2704
a2	c4	41	-0.1890	0.3344
a2	c5	41	-0.2988	0.4944
a3	c1	46	0.0109	0.0906
a3	c2	46	-0.0217	0.2347
a3	c3	46	-0.2120	0.4624
a3	c4	46	-0.2065	0.4160
a3	c5	46	-0.4022	0.5413
a4	c1	44	0.0114	0.1422
a4	c2	44	-0.0398	0.2219
a4	c3	44	-0.1875	0.3150
a4	c4	44	-0.3182	0.4649
a4	c5	44	-0.5398	0.6170
a5	c1	44	-0.0568	0.1857
a5	c2	44	-0.1307	0.3906
a5	c3	44	-0.3920	0.5741
a5	c4	44	-0.6364	0.7576
a5	c5	44	-0.8636	0.8499

<< SPHERICITY INDICES >>

== Mendoza's Multisample Sphericity Test and Epsilons ==

Effect	Lambda	approx.Chi	df	p		LB	GG	HF	CM
C	0.0000	433.2277	49	0.0000	***	0.2500	0.5696	0.5761	0.5759

LB = lower.bound, GG = Greenhouse-Geisser
HF = Huynh-Feldt-Lecoutre, CM = Chi-Muller

<< ANOVA TABLE >>

== Adjusted by Greenhouse-Geisser's Epsilon ==
== This data is UNBALANCED!! ==
== Type III SS is applied. ==

Source	SS	df	MS	F-ratio	p-value		G.eta^2
A	15.2733	4	3.8183	7.7139	0.0000	***	0.0792
s x A	106.4231	215	0.4950				
C	31.8865	2.28	13.9943	96.4349	0.0000	***	0.1523
A x C	7.0939	9.11	0.7783	5.3636	0.0000	***	0.0384
s x A x C	71.0904	489.88	0.1451				
Total	231.7964	1099	0.2109				

+p < .10, *p < .05, **p < .01, ***p < .001

<< POST ANALYSES >>

< MULTIPLE COMPARISON for "A" >

== Holm's Sequentially Rejective Bonferroni Procedure ==
== The factor < A > is analysed as independent means. ==
== Alpha level is 0.05. ==

A	n	Mean	S.D.
a1	225	-0.0978	0.2618
a2	205	-0.0927	0.3339
a3	230	-0.1663	0.4104
a4	220	-0.2148	0.4362
a5	220	-0.4159	0.6696

Pair	Diff	t-value	df	p	adj.p		
a1-a5	0.3181	4.7690	215	0.0000	0.0000	a1 > a5	*
a2-a5	0.3232	4.7326	215	0.0000	0.0000	a2 > a5	*
a3-a5	0.2496	3.7620	215	0.0002	0.0017	a3 > a5	*
a4-a5	0.2011	2.9984	215	0.0030	0.0212	a4 > a5	*
a2-a4	0.1221	1.7876	215	0.0752	0.4515	a2 = a4	
a1-a4	0.1170	1.7538	215	0.0809	0.4515	a1 = a4	

a2-a3	0.0736	1.0894	215	0.2772	1.0000	a2 = a3
a1-a3	0.0685	1.0387	215	0.3001	1.0000	a1 = a3
a3-a4	0.0485	0.7305	215	0.4659	1.0000	a3 = a4
a1-a2	-0.0051	0.0750	215	0.9403	1.0000	a1 = a2

< MULTIPLE COMPARISON for "C" >

== Holm's Sequentially Rejective Bonferroni Procedure ==
== The factor < C > is analysed as dependent means. ==
== Alpha level is 0.05. ==

C	n	Mean	S.D.
c1	220	0.0006	0.1548
c2	220	-0.0463	0.2468
c3	220	-0.1764	0.4079
c4	220	-0.2989	0.5081
c5	220	-0.4664	0.6351

Pair	Diff	t-value	df	p	adj.p	
c2-c5	0.4201	12.4831	215	0.0000	0.0000	c2 > c5 *
c1-c5	0.4670	11.9294	215	0.0000	0.0000	c1 > c5 *
c2-c4	0.2526	10.4034	215	0.0000	0.0000	c2 > c4 *
c1-c4	0.2995	9.8879	215	0.0000	0.0000	c1 > c4 *
c3-c5	0.2900	9.6654	215	0.0000	0.0000	c3 > c5 *
c4-c5	0.1675	7.1571	215	0.0000	0.0000	c4 > c5 *
c1-c3	0.1770	7.0394	215	0.0000	0.0000	c1 > c3 *
c2-c3	0.1301	6.2944	215	0.0000	0.0000	c2 > c3 *
c3-c4	0.1225	5.0479	215	0.0000	0.0000	c3 > c4 *
c1-c2	0.0469	2.9282	215	0.0038	0.0038	c1 > c2 *

< SIMPLE EFFECTS for "A x C" INTERACTION >

Effect	Lambda	approx.Chi	df	p		LB	GG	HF	CM
C at a1	0.0000	49.2256	9	0.0000	***	0.2500	0.5887	0.6241	0.6203
C at a2	0.0000	76.1514	9	0.0000	***	0.2500	0.4715	0.4940	0.4904
C at a3	0.0000	46.2855	9	0.0000	***	0.2500	0.7470	0.8061	0.8014
C at a4	0.0000	103.1570	9	0.0000	***	0.2500	0.4609	0.4806	

```
0.4776
 C at a5  0.0000    101.6198   9 0.0000 *** 0.2500 0.4273 0.4432
0.4404
--------------------------------------------------------------------
------
                               LB = lower.bound, GG = Greenhouse-
Geisser
                              HF = Huynh-Feldt-Lecoutre, CM = Chi-
Muller

--------------------------------------------------------------------
     Source      SS     df      MS  F-ratio  p-value      G.eta^2
--------------------------------------------------------------------
    A at c1  0.2563      4  0.0641   2.7585   0.0288 *     0.0488
   Er at c1  4.9937    215  0.0232
--------------------------------------------------------------------
    A at c2  0.4167      4  0.1042   1.7339   0.1436 ns    0.0313
   Er at c2 12.9182    215  0.0601
--------------------------------------------------------------------
    A at c3  3.6221      4  0.9055   5.9333   0.0002 ***   0.0994
   Er at c3 32.8128    215  0.1526
--------------------------------------------------------------------
    A at c4  6.9882      4  1.7471   7.5807   0.0000 ***   0.1236
   Er at c4 49.5490    215  0.2305
--------------------------------------------------------------------
    A at c5 11.0839      4  2.7710   7.7131   0.0000 ***   0.1255
   Er at c5 77.2397    215  0.3593
--------------------------------------------------------------------
    C at a1  1.4128   2.35  0.6000   8.6792   0.0001 ***   0.0920
s x C at a1  7.1622 103.61  0.0691
--------------------------------------------------------------------
    C at a2  3.4768   1.89  1.8436  13.2790   0.0000 ***   0.1529
s x C at a2 10.4732  75.43  0.1388
--------------------------------------------------------------------
    C at a3  5.1348   2.99  1.7184  13.8651   0.0000 ***   0.1331
s x C at a3 16.6652 134.47  0.1239
--------------------------------------------------------------------
    C at a4  8.7483   1.84  4.7448  24.3848   0.0000 ***   0.2100
s x C at a4 15.4267  79.28  0.1946
--------------------------------------------------------------------
    C at a5 20.2369   1.71 11.8396  40.7333   0.0000 ***   0.2061
s x C at a5 21.3631   73.5  0.2907
--------------------------------------------------------------------
                        +p < .10, *p < .05, **p < .01, ***p < .001


< MULTIPLE COMPARISON for "A at c1" >

== Holm's Sequentially Rejective Bonferroni Procedure ==
== The factor < A at c1 > is analysed as independent means. ==
== Alpha level is 0.05. ==

----------------------------------------------------------
  Pair     Diff  t-value  df        p   adj.p
```

a2-a5	0.1056	3.1921	215	0.0016	0.0162	a2 > a5 *
a3-a5	0.0677	2.1062	215	0.0363	0.3271	a3 = a5
a4-a5	0.0682	2.0984	215	0.0370	0.3271	a4 = a5
a1-a2	-0.0599	1.8202	215	0.0701	0.4908	a1 = a2
a1-a5	0.0457	1.4146	215	0.1586	0.9518	a1 = a5
a2-a3	0.0379	1.1582	215	0.2481	1.0000	a2 = a3
a2-a4	0.0374	1.1311	215	0.2593	1.0000	a2 = a4
a1-a4	-0.0225	0.6956	215	0.4875	1.0000	a1 = a4
a1-a3	-0.0220	0.6879	215	0.4923	1.0000	a1 = a3
a3-a4	-0.0005	0.0154	215	0.9877	1.0000	a3 = a4

< MULTIPLE COMPARISON for "A at c3" >

== Holm's Sequentially Rejective Bonferroni Procedure ==
== The factor < A at c3 > is analysed as independent means. ==
== Alpha level is 0.05. ==

Pair	Diff	t-value	df	p	adj.p	
a2-a5	0.3738	4.4075	215	0.0000	0.0002	a2 > a5 *
a1-a5	0.3198	3.8614	215	0.0001	0.0013	a1 > a5 *
a4-a5	0.2045	2.4558	215	0.0148	0.1188	a4 = a5
a2-a3	0.1937	2.3081	215	0.0219	0.1536	a2 = a3
a3-a5	0.1801	2.1861	215	0.0299	0.1793	a3 = a5
a2-a4	0.1692	1.9954	215	0.0473	0.2363	a2 = a4
a1-a3	0.1397	1.7059	215	0.0895	0.3578	a1 = a3
a1-a4	0.1153	1.3918	215	0.1654	0.4963	a1 = a4
a1-a2	-0.0539	0.6394	215	0.5232	1.0000	a1 = a2
a3-a4	-0.0245	0.2969	215	0.7668	1.0000	a3 = a4

< MULTIPLE COMPARISON for "A at c4" >

== Holm's Sequentially Rejective Bonferroni Procedure ==
== The factor < A at c4 > is analysed as independent means. ==
== Alpha level is 0.05. ==

Pair	Diff	t-value	df	p	adj.p	
a1-a5	0.4919	4.8332	215	0.0000	0.0000	a1 > a5 *
a2-a5	0.4473	4.2929	215	0.0000	0.0002	a2 > a5 *
a3-a5	0.4298	4.2461	215	0.0000	0.0003	a3 > a5 *
a4-a5	0.3182	3.1088	215	0.0021	0.0149	a4 > a5 *
a1-a4	0.1737	1.7070	215	0.0893	0.5356	a1 = a4
a2-a4	0.1292	1.2395	215	0.2165	1.0000	a2 = a4
a3-a4	0.1117	1.1030	215	0.2713	1.0000	a3 = a4
a1-a3	0.0621	0.6167	215	0.5381	1.0000	a1 = a3
a1-a2	0.0446	0.4301	215	0.6675	1.0000	a1 = a2

a2-a3	0.0175	0.1697	215	0.8654	1.0000	a2 = a3

< MULTIPLE COMPARISON for "A at c5" >

== Holm's Sequentially Rejective Bonferroni Procedure ==
== The factor < A at c5 > is analysed as independent means. ==
== Alpha level is 0.05. ==

Pair	Diff	t-value	df	p	adj.p	
a1-a5	0.6359	5.0038	215	0.0000	0.0000	a1 > a5 *
a2-a5	0.5649	4.3416	215	0.0000	0.0002	a2 > a5 *
a3-a5	0.4615	3.6511	215	0.0003	0.0026	a3 > a5 *
a4-a5	0.3239	2.5344	215	0.0120	0.0838	a4 = a5
a1-a4	0.3120	2.4552	215	0.0149	0.0893	a1 = a4
a2-a4	0.2410	1.8523	215	0.0654	0.3268	a2 = a4
a1-a3	0.1744	1.3877	215	0.1667	0.6666	a1 = a3
a3-a4	0.1376	1.0887	215	0.2775	0.8326	a3 = a4
a2-a3	0.1034	0.8032	215	0.4228	0.8455	a2 = a3
a1-a2	0.0710	0.5487	215	0.5838	0.8455	a1 = a2

< MULTIPLE COMPARISON for "C at a1" >

== Holm's Sequentially Rejective Bonferroni Procedure ==
== The factor < C at a1 > is analysed as dependent means. ==
== Alpha level is 0.05. ==

Pair	Diff	t-value	df	p	adj.p	
c3-c5	0.1556	3.6175	44	0.0008	0.0076	c3 > c5 *
c2-c5	0.1944	3.5364	44	0.0010	0.0087	c2 > c5 *
c1-c5	0.2167	3.4744	44	0.0012	0.0093	c1 > c5 *
c2-c4	0.1111	3.1623	44	0.0028	0.0198	c2 > c4 *
c1-c4	0.1333	3.0336	44	0.0040	0.0243	c1 > c4 *
c3-c4	0.0722	2.2295	44	0.0309	0.1547	c3 = c4
c4-c5	0.0833	1.9149	44	0.0620	0.2481	c4 = c5
c1-c3	0.0611	1.7132	44	0.0937	0.2812	c1 = c3
c2-c3	0.0389	1.2656	44	0.2123	0.4246	c2 = c3
c1-c2	0.0222	0.7031	44	0.4857	0.4857	c1 = c2

< MULTIPLE COMPARISON for "C at a2" >

== Holm's Sequentially Rejective Bonferroni Procedure ==
== The factor < C at a2 > is analysed as dependent means. ==
== Alpha level is 0.05. ==

Pair	Diff	t-value	df	p	adj.p	
c1-c4	0.2378	4.6074	40	0.0000	0.0004	c1 > c4 *
c1-c5	0.3476	4.5404	40	0.0001	0.0005	c1 > c5 *
c2-c5	0.2927	3.7983	40	0.0005	0.0039	c2 > c5 *
c2-c4	0.1829	3.5921	40	0.0009	0.0062	c2 > c4 *
c3-c5	0.2805	3.5437	40	0.0010	0.0062	c3 > c5 *
c3-c4	0.1707	3.4878	40	0.0012	0.0062	c3 > c4 *
c4-c5	0.1098	2.4632	40	0.0182	0.0727	c4 = c5
c1-c2	0.0549	1.5960	40	0.1184	0.3551	c1 = c2
c1-c3	0.0671	1.5674	40	0.1249	0.3551	c1 = c3
c2-c3	0.0122	0.3497	40	0.7284	0.7284	c2 = c3

< MULTIPLE COMPARISON for "C at a3" >

== Holm's Sequentially Rejective Bonferroni Procedure ==
== The factor < C at a3 > is analysed as dependent means. ==
== Alpha level is 0.05. ==

Pair	Diff	t-value	df	p	adj.p	
c2-c5	0.3804	5.6629	45	0.0000	0.0000	c2 > c5 *
c1-c5	0.4130	5.2318	45	0.0000	0.0000	c1 > c5 *
c2-c4	0.1848	3.9946	45	0.0002	0.0019	c2 > c4 *
c1-c4	0.2174	3.5641	45	0.0009	0.0061	c1 > c4 *
c1-c3	0.2228	3.2215	45	0.0024	0.0142	c1 > c3 *
c4-c5	0.1957	3.1012	45	0.0033	0.0166	c4 > c5 *
c2-c3	0.1902	2.9013	45	0.0057	0.0229	c2 > c3 *
c3-c5	0.1902	2.5358	45	0.0148	0.0443	c3 > c5 *
c1-c2	0.0326	0.9476	45	0.3484	0.6968	c1 = c2
c3-c4	-0.0054	0.0885	45	0.9299	0.9299	c3 = c4

< MULTIPLE COMPARISON for "C at a4" >

== Holm's Sequentially Rejective Bonferroni Procedure ==
== The factor < C at a4 > is analysed as dependent means. ==
== Alpha level is 0.05. ==

Pair	Diff	t-value	df	p	adj.p	
c2-c5	0.5000	5.8920	43	0.0000	0.0000	c2 > c5 *
c1-c5	0.5511	5.6785	43	0.0000	0.0000	c1 > c5 *
c3-c5	0.3523	5.2434	43	0.0000	0.0000	c3 > c5 *
c1-c4	0.3295	4.8889	43	0.0000	0.0001	c1 > c4 *
c2-c4	0.2784	4.6518	43	0.0000	0.0002	c2 > c4 *
c2-c3	0.1477	4.2464	43	0.0001	0.0006	c2 > c3 *
c1-c3	0.1989	3.8889	43	0.0003	0.0014	c1 > c3 *

c4-c5	0.2216	3.8094	43	0.0004	0.0014	c4 > c5 *
c3-c4	0.1307	2.2854	43	0.0273	0.0546	c3 = c4
c1-c2	0.0511	1.5939	43	0.1183	0.1183	c1 = c2

< MULTIPLE COMPARISON for "C at a5" >

== Holm's Sequentially Rejective Bonferroni Procedure ==
== The factor < C at a5 > is analysed as dependent means. ==
== Alpha level is 0.05. ==

Pair	Diff	t-value	df	p	adj.p	
c2-c5	0.7330	8.2873	43	0.0000	0.0000	c2 > c5 *
c1-c5	0.8068	7.1108	43	0.0000	0.0000	c1 > c5 *
c3-c5	0.4716	7.0768	43	0.0000	0.0000	c3 > c5 *
c2-c4	0.5057	6.9780	43	0.0000	0.0000	c2 > c4 *
c1-c4	0.5795	5.8067	43	0.0000	0.0000	c1 > c4 *
c2-c3	0.2614	5.0246	43	0.0000	0.0000	c2 > c3 *
c4-c5	0.2273	4.8081	43	0.0000	0.0001	c4 > c5 *
c1-c3	0.3352	4.7326	43	0.0000	0.0001	c1 > c3 *
c3-c4	0.2443	3.8024	43	0.0004	0.0009	c3 > c4 *
c1-c2	0.0739	1.6433	43	0.1076	0.1076	c1 = c2

output is over --------------------///

[[Simple Effects for b4]]

[AsB-Type Design]

This output was generated by anovakun 4.8.5 under R version 4.0.2.
It was executed on Mon Oct 11 18:22:30 2021.

<< DESCRIPTIVE STATISTICS >>

A	C	n	Mean	S.D.
a1	c1	45	0.0000	0.0000
a1	c2	45	0.0000	0.0754
a1	c3	45	-0.0278	0.1619
a1	c4	45	-0.1167	0.2900
a1	c5	45	-0.1056	0.2528
a2	c1	41	0.0122	0.1364
a2	c2	41	-0.0183	0.1713
a2	c3	41	-0.0122	0.3352
a2	c4	41	-0.0244	0.2359
a2	c5	41	-0.1463	0.3831
a3	c1	46	0.0109	0.1577

```
 a3  c2  46  -0.0109  0.1173
 a3  c3  46  -0.0652  0.2710
 a3  c4  46  -0.0978  0.3139
 a3  c5  46  -0.2880  0.5296
 a4  c1  44   0.0057  0.1373
 a4  c2  44   0.0227  0.1301
 a4  c3  44  -0.0568  0.1693
 a4  c4  44  -0.1193  0.3120
 a4  c5  44  -0.2955  0.5037
 a5  c1  44  -0.0398  0.2347
 a5  c2  44  -0.0795  0.3270
 a5  c3  44  -0.2898  0.5730
 a5  c4  44  -0.3693  0.5943
 a5  c5  44  -0.5795  0.7601
-------------------------------


<< SPHERICITY INDICES >>

== Mendoza's Multisample Sphericity Test and Epsilons ==

--------------------------------------------------------------------
-----
 Effect  Lambda  approx.Chi  df      p         LB     GG     HF
CM
--------------------------------------------------------------------
-----
      C  0.0000    449.7029  49 0.0000 *** 0.2500 0.6651 0.6742
0.6741
--------------------------------------------------------------------
-----
                              LB = lower.bound, GG = Greenhouse-
Geisser
                             HF = Huynh-Feldt-Lecoutre, CM = Chi-
Muller


<< ANOVA TABLE >>

== Adjusted by Greenhouse-Geisser's Epsilon ==
== This data is UNBALANCED!! ==
== Type III SS is applied. ==

-----------------------------------------------------------------
   Source       SS    df     MS  F-ratio  p-value      G.eta^2
-----------------------------------------------------------------
        A   7.8098     4 1.9525   5.9847   0.0001 ***   0.0595
    s x A  70.1415   215 0.3262
-----------------------------------------------------------------
        C  11.3737  2.66 4.2751  45.8255   0.0000 ***   0.0843
    A x C   3.8199 10.64 0.3590   3.8477   0.0000 ***   0.0300
s x A x C  53.3622   572 0.0933
-----------------------------------------------------------------
    Total 146.6264  1099 0.1334
```

+p < .10, *p < .05, **p < .01, ***p < .001

<< POST ANALYSES >>

< MULTIPLE COMPARISON for "A" >

== Holm's Sequentially Rejective Bonferroni Procedure ==
== The factor < A > is analysed as independent means. ==
== Alpha level is 0.05. ==

A	n	Mean	S.D.
a1	225	-0.0500	0.1948
a2	205	-0.0378	0.2725
a3	230	-0.0902	0.3284
a4	220	-0.0886	0.3080
a5	220	-0.2716	0.5642

Pair	Diff	t-value	df	p	adj.p	
a2-a5	0.2338	4.2164	215	0.0000	0.0004	a2 > a5 *
a1-a5	0.2216	4.0917	215	0.0001	0.0005	a1 > a5 *
a3-a5	0.1814	3.3672	215	0.0009	0.0072	a3 > a5 *
a4-a5	0.1830	3.3595	215	0.0009	0.0072	a4 > a5 *
a2-a3	0.0524	0.9554	215	0.3405	1.0000	a2 = a3
a2-a4	0.0508	0.9168	215	0.3603	1.0000	a2 = a4
a1-a3	0.0402	0.7509	215	0.4535	1.0000	a1 = a3
a1-a4	0.0386	0.7134	215	0.4764	1.0000	a1 = a4
a1-a2	-0.0122	0.2211	215	0.8252	1.0000	a1 = a2
a3-a4	-0.0016	0.0294	215	0.9766	1.0000	a3 = a4

< MULTIPLE COMPARISON for "C" >

== Holm's Sequentially Rejective Bonferroni Procedure ==
== The factor < C > is analysed as dependent means. ==
== Alpha level is 0.05. ==

C	n	Mean	S.D.
c1	220	-0.0022	0.1530
c2	220	-0.0172	0.1866
c3	220	-0.0904	0.3482
c4	220	-0.1455	0.3865
c5	220	-0.2830	0.5369

Pair	Diff	t-value	df	p	adj.p	
c1-c5	0.2808	8.9801	215	0.0000	0.0000	c1 > c5 *
c2-c5	0.2658	8.8318	215	0.0000	0.0000	c2 > c5 *
c3-c5	0.1926	7.2054	215	0.0000	0.0000	c3 > c5 *
c1-c4	0.1433	6.2427	215	0.0000	0.0000	c1 > c4 *
c2-c4	0.1283	6.1465	215	0.0000	0.0000	c2 > c4 *
c4-c5	0.1375	4.8567	215	0.0000	0.0000	c4 > c5 *
c1-c3	0.0882	4.4092	215	0.0000	0.0001	c1 > c3 *
c2-c3	0.0732	4.0493	215	0.0001	0.0002	c2 > c3 *
c3-c4	0.0551	2.7174	215	0.0071	0.0142	c3 > c4 *
c1-c2	0.0150	1.2274	215	0.2210	0.2210	c1 = c2

< SIMPLE EFFECTS for "A x C" INTERACTION >

Effect	Lambda	approx.Chi	df	p		LB	GG	HF	CM
C at a1	0.0000	93.6185	9	0.0000	***	0.2500	0.6169	0.6564	0.6524
C at a2	0.0000	48.4921	9	0.0000	***	0.2500	0.6645	0.7162	0.7110
C at a3	0.0000	94.4459	9	0.0000	***	0.2500	0.5347	0.5622	0.5589
C at a4	0.0000	70.6986	9	0.0000	***	0.2500	0.5529	0.5841	0.5804
C at a5	0.0000	71.8027	9	0.0000	***	0.2500	0.5170	0.5436	0.5401

LB = lower.bound, GG = Greenhouse-Geisser
HF = Huynh-Feldt-Lecoutre, CM = Chi-Muller

Source	SS	df	MS	F-ratio	p-value		G.eta^2
A at c1	0.0814	4	0.0204	0.8679	0.4840	ns	0.0159
Er at c1	5.0424	215	0.0235				
A at c2	0.2564	4	0.0641	1.8704	0.1167	ns	0.0336
Er at c2	7.3672	215	0.0343				
A at c3	2.2549	4	0.5637	4.9874	0.0007	***	0.0849
Er at c3	24.3019	215	0.1130				
A at c4	2.9774	4	0.7443	5.3825	0.0004	***	0.0910
Er at c4	29.7326	215	0.1383				

```
-------------------------------------------------------------------
    A at c5  6.0597       4 1.5149   5.7082   0.0002 ***   0.0960
   Er at c5 57.0597     215 0.2654
-------------------------------------------------------------------
    C at a1  0.5861    2.47 0.2375   4.2882   0.0106 *     0.0690
s x C at a1  6.0139  108.57 0.0554
-------------------------------------------------------------------
    C at a2  0.6354    2.66 0.2390   4.5878   0.0064 **    0.0420
s x C at a2  5.5396  106.32 0.0521
-------------------------------------------------------------------
    C at a3  2.5913    2.14 1.2116   7.9958   0.0005 ***   0.1050
s x C at a3 14.5837   96.25 0.1515
-------------------------------------------------------------------
    C at a4  2.9051    2.21 1.3137  11.1836   0.0000 ***   0.1399
s x C at a4 11.1699   95.09 0.1175
-------------------------------------------------------------------
    C at a5  8.5949    2.07 4.1560  23.0195   0.0000 ***   0.1233
s x C at a5 16.0551   88.93 0.1805
-------------------------------------------------------------------
                       +p < .10, *p < .05, **p < .01, ***p < .001


< MULTIPLE COMPARISON for "A at c3" >

== Holm's Sequentially Rejective Bonferroni Procedure ==
== The factor < A at c3 > is analysed as independent means. ==
== Alpha level is 0.05. ==

----------------------------------------------------------
  Pair     Diff  t-value  df       p   adj.p
----------------------------------------------------------
 a2-a5   0.2776   3.8036 215  0.0002  0.0019  a2 > a5 *
 a1-a5   0.2620   3.6756 215  0.0003  0.0027  a1 > a5 *
 a4-a5   0.2330   3.2500 215  0.0013  0.0107  a4 > a5 *
 a3-a5   0.2246   3.1674 215  0.0018  0.0123  a3 > a5 *
 a2-a3   0.0530   0.7343 215  0.4636  1.0000  a2 = a3
 a2-a4   0.0446   0.6115 215  0.5415  1.0000  a2 = a4
 a1-a3   0.0374   0.5311 215  0.5959  1.0000  a1 = a3
 a1-a4   0.0290   0.4074 215  0.6841  1.0000  a1 = a4
 a1-a2  -0.0156   0.2147 215  0.8302  1.0000  a1 = a2
 a3-a4  -0.0084   0.1185 215  0.9058  1.0000  a3 = a4
----------------------------------------------------------


< MULTIPLE COMPARISON for "A at c4" >

== Holm's Sequentially Rejective Bonferroni Procedure ==
== The factor < A at c4 > is analysed as independent means. ==
== Alpha level is 0.05. ==

----------------------------------------------------------
  Pair     Diff  t-value  df       p   adj.p
----------------------------------------------------------
 a2-a5   0.3449   4.2731 215  0.0000  0.0003  a2 > a5 *
```

a3-a5	0.2715	3.4621	215	0.0006	0.0058	a3 > a5 *
a1-a5	0.2527	3.2045	215	0.0016	0.0125	a1 > a5 *
a4-a5	0.2500	3.1532	215	0.0018	0.0129	a4 > a5 *
a2-a4	0.0949	1.1760	215	0.2409	1.0000	a2 = a4
a1-a2	-0.0923	1.1493	215	0.2517	1.0000	a1 = a2
a2-a3	0.0734	0.9194	215	0.3589	1.0000	a2 = a3
a3-a4	0.0215	0.2741	215	0.7843	1.0000	a3 = a4
a1-a3	-0.0188	0.2416	215	0.8093	1.0000	a1 = a3
a1-a4	0.0027	0.0336	215	0.9732	1.0000	a1 = a4

< MULTIPLE COMPARISON for "A at c5" >

== Holm's Sequentially Rejective Bonferroni Procedure ==
== The factor < A at c5 > is analysed as independent means. ==
== Alpha level is 0.05. ==

Pair	Diff	t-value	df	p	adj.p	
a1-a5	0.4740	4.3397	215	0.0000	0.0002	a1 > a5 *
a2-a5	0.4332	3.8740	215	0.0001	0.0013	a2 > a5 *
a3-a5	0.2915	2.6834	215	0.0079	0.0628	a3 = a5
a4-a5	0.2841	2.5866	215	0.0104	0.0725	a4 = a5
a1-a4	0.1899	1.7387	215	0.0835	0.5012	a1 = a4
a1-a3	0.1825	1.6895	215	0.0926	0.5012	a1 = a3
a2-a4	0.1491	1.3335	215	0.1838	0.7352	a2 = a4
a2-a3	0.1417	1.2807	215	0.2017	0.7352	a2 = a3
a1-a2	0.0408	0.3667	215	0.7142	1.0000	a1 = a2
a3-a4	0.0074	0.0682	215	0.9457	1.0000	a3 = a4

< MULTIPLE COMPARISON for "C at a1" >

== Holm's Sequentially Rejective Bonferroni Procedure ==
== The factor < C at a1 > is analysed as dependent means. ==
== Alpha level is 0.05. ==

Pair	Diff	t-value	df	p	adj.p	
c1-c5	0.1056	2.8014	44	0.0075	0.0753	c1 = c5
c2-c5	0.1056	2.7411	44	0.0088	0.0793	c2 = c5
c1-c4	0.1167	2.6988	44	0.0098	0.0793	c1 = c4
c2-c4	0.1167	2.6120	44	0.0123	0.0859	c2 = c4
c3-c5	0.0778	1.9264	44	0.0605	0.3632	c3 = c5
c3-c4	0.0889	1.8353	44	0.0732	0.3661	c3 = c4
c1-c3	0.0278	1.1512	44	0.2559	1.0000	c1 = c3
c2-c3	0.0278	1.0935	44	0.2801	1.0000	c2 = c3
c4-c5	-0.0111	0.1999	44	0.8425	1.0000	c4 = c5
c1-c2	0.0000	0.0000	44	1.0000	1.0000	c1 = c2

< MULTIPLE COMPARISON for "C at a2" >

== Holm's Sequentially Rejective Bonferroni Procedure ==
== The factor < C at a2 > is analysed as dependent means. ==
== Alpha level is 0.05. ==

Pair	Diff	t-value	df	p	adj.p	
c1-c5	0.1585	2.9553	40	0.0052	0.0522	c1 = c5
c3-c5	0.1341	2.8008	40	0.0078	0.0703	c3 = c5
c2-c5	0.1280	2.6724	40	0.0108	0.0868	c2 = c5
c4-c5	0.1220	2.5451	40	0.0149	0.1042	c4 = c5
c1-c4	0.0366	1.2325	40	0.2250	1.0000	c1 = c4
c1-c2	0.0305	1.1516	40	0.2563	1.0000	c1 = c2
c1-c3	0.0244	0.6278	40	0.5337	1.0000	c1 = c3
c3-c4	0.0122	0.4221	40	0.6752	1.0000	c3 = c4
c2-c4	0.0061	0.1835	40	0.8553	1.0000	c2 = c4
c2-c3	-0.0061	0.1332	40	0.8947	1.0000	c2 = c3

< MULTIPLE COMPARISON for "C at a3" >

== Holm's Sequentially Rejective Bonferroni Procedure ==
== The factor < C at a3 > is analysed as dependent means. ==
== Alpha level is 0.05. ==

Pair	Diff	t-value	df	p	adj.p	
c1-c5	0.2989	3.8932	45	0.0003	0.0032	c1 > c5 *
c2-c5	0.2772	3.4445	45	0.0012	0.0112	c2 > c5 *
c3-c5	0.2228	3.4217	45	0.0013	0.0112	c3 > c5 *
c4-c5	0.1902	2.2328	45	0.0306	0.2141	c4 = c5
c2-c4	0.0870	2.1449	45	0.0374	0.2244	c2 = c4
c1-c4	0.1087	2.0479	45	0.0464	0.2322	c1 = c4
c1-c3	0.0761	1.6125	45	0.1138	0.4554	c1 = c3
c2-c3	0.0543	1.5673	45	0.1241	0.4554	c2 = c3
c1-c2	0.0217	0.7265	45	0.4713	0.9425	c1 = c2
c3-c4	0.0326	0.6429	45	0.5236	0.9425	c3 = c4

< MULTIPLE COMPARISON for "C at a4" >

== Holm's Sequentially Rejective Bonferroni Procedure ==
== The factor < C at a4 > is analysed as dependent means. ==
== Alpha level is 0.05. ==

Pair	Diff	t-value	df	p	adj.p

c2-c5	0.3182	4.3941	43	0.0001	0.0007	c2 > c5 *
c1-c5	0.3011	4.1794	43	0.0001	0.0013	c1 > c5 *
c3-c5	0.2386	3.3252	43	0.0018	0.0145	c3 > c5 *
c2-c4	0.1420	3.1188	43	0.0032	0.0227	c2 > c4 *
c4-c5	0.1761	2.6360	43	0.0116	0.0697	c4 = c5
c1-c4	0.1250	2.5811	43	0.0133	0.0697	c1 = c4
c2-c3	0.0795	2.5462	43	0.0146	0.0697	c2 = c3
c1-c3	0.0625	1.9149	43	0.0622	0.1865	c1 = c3
c3-c4	0.0625	1.2793	43	0.2076	0.4153	c3 = c4
c1-c2	-0.0170	0.6841	43	0.4976	0.4976	c1 = c2

< MULTIPLE COMPARISON for "C at a5" >

== Holm's Sequentially Rejective Bonferroni Procedure ==
== The factor < C at a5 > is analysed as dependent means. ==
== Alpha level is 0.05. ==

Pair	Diff	t-value	df	p	adj.p	
c2-c5	0.5000	6.0908	43	0.0000	0.0000	c2 > c5 *
c1-c5	0.5398	5.7595	43	0.0000	0.0000	c1 > c5 *
c1-c4	0.3295	4.6576	43	0.0000	0.0002	c1 > c4 *
c2-c4	0.2898	4.6046	43	0.0000	0.0003	c2 > c4 *
c3-c5	0.2898	4.3901	43	0.0001	0.0004	c3 > c5 *
c4-c5	0.2102	4.3141	43	0.0001	0.0005	c4 > c5 *
c1-c3	0.2500	3.7027	43	0.0006	0.0024	c1 > c3 *
c2-c3	0.2102	3.6237	43	0.0008	0.0024	c2 > c3 *
c3-c4	0.0795	1.8244	43	0.0750	0.1501	c3 = c4
c1-c2	0.0398	1.0692	43	0.2909	0.2909	c1 = c2

[[Simple Effects for c1]]

[AsB-Type Design]

This output was generated by anovakun 4.8.5 under R version 4.0.2.
It was executed on Mon Oct 11 18:22:31 2021.

<< DESCRIPTIVE STATISTICS >>

A	B	n	Mean	S.D.
a1	b1	45	-0.0222	0.2601
a1	b2	45	-0.0111	0.1406
a1	b3	45	-0.0111	0.1187
a1	b4	45	0.0000	0.0000
a2	b1	41	0.0244	0.3298

```
 a2  b2  41  -0.0305  0.1695
 a2  b3  41   0.0488  0.2033
 a2  b4  41   0.0122  0.1364
 a3  b1  46  -0.0652  0.2496
 a3  b2  46   0.0000  0.0527
 a3  b3  46   0.0109  0.0906
 a3  b4  46   0.0109  0.1577
 a4  b1  44   0.0227  0.3316
 a4  b2  44   0.0455  0.3015
 a4  b3  44   0.0114  0.1422
 a4  b4  44   0.0057  0.1373
 a5  b1  44  -0.1875  0.3855
 a5  b2  44  -0.1136  0.2825
 a5  b3  44  -0.0568  0.1857
 a5  b4  44  -0.0398  0.2347
-------------------------------


<< SPHERICITY INDICES >>

== Mendoza's Multisample Sphericity Test and Epsilons ==

--------------------------------------------------------------------
-----
 Effect  Lambda  approx.Chi  df      p         LB     GG     HF
CM
--------------------------------------------------------------------
-----
      B  0.0000    293.2677  29 0.0000 *** 0.3333 0.6697 0.6760
0.6759
--------------------------------------------------------------------
-----
                              LB = lower.bound, GG = Greenhouse-
Geisser
                             HF = Huynh-Feldt-Lecoutre, CM = Chi-
Muller


<< ANOVA TABLE >>

== Adjusted by Greenhouse-Geisser's Epsilon ==
== This data is UNBALANCED!! ==
== Type III SS is applied. ==

-----------------------------------------------------------------
   Source      SS     df     MS  F-ratio  p-value      G.eta^2
-----------------------------------------------------------------
        A  1.6218      4 0.4054   4.1364   0.0030 **    0.0383
    s x A 21.0740    215 0.0980
-----------------------------------------------------------------
        B  0.3008   2.01 0.1497   3.2835   0.0382 *     0.0073
    A x B  0.6504   8.04 0.0809   1.7749   0.0797 +     0.0157
s x A x B 19.6976 431.97 0.0456
-----------------------------------------------------------------
```

```
    Total 43.3520    879 0.0493
                     +p < .10, *p < .05, **p < .01, ***p < .001
```

<< POST ANALYSES >>

< MULTIPLE COMPARISON for "A" >

== Holm's Sequentially Rejective Bonferroni Procedure ==
== The factor < A > is analysed as independent means. ==
== Alpha level is 0.05. ==

A	n	Mean	S.D.
a1	180	-0.0111	0.1582
a2	164	0.0137	0.2220
a3	184	-0.0109	0.1586
a4	176	0.0213	0.2433
a5	176	-0.0994	0.2855

Pair	Diff	t-value	df	p	adj.p	
a4-a5	0.1207	3.6177	215	0.0004	0.0037	a4 > a5 *
a2-a5	0.1132	3.3300	215	0.0010	0.0092	a2 > a5 *
a3-a5	0.0886	2.6829	215	0.0079	0.0629	a3 = a5
a1-a5	0.0883	2.6612	215	0.0084	0.0629	a1 = a5
a1-a4	-0.0324	0.9768	215	0.3298	1.0000	a1 = a4
a3-a4	-0.0322	0.9748	215	0.3308	1.0000	a3 = a4
a1-a2	-0.0248	0.7347	215	0.4633	1.0000	a1 = a2
a2-a3	0.0246	0.7314	215	0.4654	1.0000	a2 = a3
a2-a4	-0.0076	0.2233	215	0.8235	1.0000	a2 = a4
a1-a3	-0.0002	0.0074	215	0.9941	1.0000	a1 = a3

< MULTIPLE COMPARISON for "B" >

== Holm's Sequentially Rejective Bonferroni Procedure ==
== The factor < B > is analysed as dependent means. ==
== Alpha level is 0.05. ==

B	n	Mean	S.D.
b1	220	-0.0456	0.3211
b2	220	-0.0220	0.2146
b3	220	0.0006	0.1548
b4	220	-0.0022	0.1530

Pair	Diff	t-value	df	p	adj.p	
b1-b3	-0.0462	2.2229	215	0.0273	0.1636	b1 = b3
b1-b4	-0.0434	2.0789	215	0.0388	0.1941	b1 = b4
b2-b3	-0.0226	1.6722	215	0.0959	0.3838	b2 = b3
b2-b4	-0.0198	1.5020	215	0.1346	0.4037	b2 = b4
b1-b2	-0.0236	1.2664	215	0.2067	0.4135	b1 = b2
b3-b4	0.0028	0.2836	215	0.7770	0.7770	b3 = b4

< SIMPLE EFFECTS for "A x B" INTERACTION >

Effect	Lambda	approx.Chi	df	p		LB	GG	HF	CM
B at a1	0.0000	55.2157	5	0.0000	***	0.3333	0.5477	0.5661	0.5627
B at a2	0.0000	55.0422	5	0.0000	***	0.3333	0.5598	0.5816	0.5773
B at a3	0.0000	70.4796	5	0.0000	***	0.3333	0.5365	0.5534	0.5502
B at a4	0.0000	52.3540	5	0.0000	***	0.3333	0.7092	0.7472	0.7424
B at a5	0.0000	35.5918	5	0.0000	***	0.3333	0.6323	0.6606	0.6564

LB = lower.bound, GG = Greenhouse-Geisser
HF = Huynh-Feldt-Lecoutre, CM = Chi-Muller

Source	SS	df	MS	F-ratio	p-value		G.eta^2
A at b1	1.3343	4	0.3336	3.3749	0.0106	*	0.0591
Er at b1	21.2506	215	0.0988				
A at b2	0.6002	4	0.1500	3.4013	0.0101	*	0.0595
Er at b2	9.4847	215	0.0441				
A at b3	0.2563	4	0.0641	2.7585	0.0288	*	0.0488
Er at b3	4.9937	215	0.0232				
A at b4	0.0814	4	0.0204	0.8679	0.4840	ns	0.0159
Er at b4	5.0424	215	0.0235				
B at a1	0.0111	1.64	0.0068	0.1870	0.7868	ns	0.0025
s x B at a1	2.6139	72.3	0.0362				

B at a2	0.1353	1.68	0.0806	1.2443	0.2900	ns	0.0168
s x B at a2	4.3491	67.18	0.0647				
B at a3	0.1848	1.61	0.1148	2.6065	0.0918	+	0.0401
s x B at a3	3.1902	72.42	0.0440				
B at a4	0.0408	2.13	0.0192	0.3245	0.7370	ns	0.0039
s x B at a4	5.4123	91.49	0.0592				
B at a5	0.5866	1.9	0.3093	6.1048	0.0039	**	0.0411
s x B at a5	4.1321	81.56	0.0507				

+p < .10, *p < .05, **p < .01, ***p < .001

< MULTIPLE COMPARISON for "A at b1" >

== Holm's Sequentially Rejective Bonferroni Procedure ==
== The factor < A at b1 > is analysed as independent means. ==
== Alpha level is 0.05. ==

Pair	Diff	t-value	df	p	adj.p	
a4-a5	0.2102	3.1364	215	0.0019	0.0195	a4 > a5 *
a2-a5	0.2119	3.1049	215	0.0022	0.0195	a2 > a5 *
a1-a5	0.1653	2.4796	215	0.0139	0.1114	a1 = a5
a3-a5	0.1223	1.8445	215	0.0665	0.4654	a3 = a5
a2-a3	0.0896	1.3271	215	0.1859	1.0000	a2 = a3
a3-a4	-0.0879	1.3266	215	0.1861	1.0000	a3 = a4
a1-a2	-0.0466	0.6867	215	0.4930	1.0000	a1 = a2
a1-a4	-0.0449	0.6744	215	0.5008	1.0000	a1 = a4
a1-a3	0.0430	0.6523	215	0.5149	1.0000	a1 = a3
a2-a4	0.0017	0.0244	215	0.9806	1.0000	a2 = a4

< MULTIPLE COMPARISON for "A at b2" >

== Holm's Sequentially Rejective Bonferroni Procedure ==
== The factor < A at b2 > is analysed as independent means. ==
== Alpha level is 0.05. ==

Pair	Diff	t-value	df	p	adj.p	
a4-a5	0.1591	3.5527	215	0.0005	0.0047	a4 > a5 *
a3-a5	0.1136	2.5657	215	0.0110	0.0988	a3 = a5
a1-a5	0.1025	2.3024	215	0.0223	0.1782	a1 = a5
a2-a5	0.0831	1.8238	215	0.0696	0.4870	a2 = a5
a2-a4	-0.0759	1.6657	215	0.0972	0.5834	a2 = a4
a1-a4	-0.0566	1.2703	215	0.2054	1.0000	a1 = a4
a3-a4	-0.0455	1.0263	215	0.3059	1.0000	a3 = a4
a2-a3	-0.0305	0.6758	215	0.4999	1.0000	a2 = a3

```
 a1-a2   0.0194   0.4273 215  0.6696  1.0000  a1 = a2
 a1-a3  -0.0111   0.2523 215  0.8010  1.0000  a1 = a3
----------------------------------------------------------


< MULTIPLE COMPARISON for "A at b3" >

== Holm's Sequentially Rejective Bonferroni Procedure ==
== The factor < A at b3 > is analysed as independent means. ==
== Alpha level is 0.05. ==

----------------------------------------------------------
  Pair     Diff  t-value  df       p   adj.p
----------------------------------------------------------
 a2-a5   0.1056   3.1921 215  0.0016  0.0162  a2 > a5 *
 a3-a5   0.0677   2.1062 215  0.0363  0.3271  a3 = a5
 a4-a5   0.0682   2.0984 215  0.0370  0.3271  a4 = a5
 a1-a2  -0.0599   1.8202 215  0.0701  0.4908  a1 = a2
 a1-a5   0.0457   1.4146 215  0.1586  0.9518  a1 = a5
 a2-a3   0.0379   1.1582 215  0.2481  1.0000  a2 = a3
 a2-a4   0.0374   1.1311 215  0.2593  1.0000  a2 = a4
 a1-a4  -0.0225   0.6956 215  0.4875  1.0000  a1 = a4
 a1-a3  -0.0220   0.6879 215  0.4923  1.0000  a1 = a3
 a3-a4  -0.0005   0.0154 215  0.9877  1.0000  a3 = a4
----------------------------------------------------------


< MULTIPLE COMPARISON for "B at a3" >

== Holm's Sequentially Rejective Bonferroni Procedure ==
== The factor < B at a3 > is analysed as dependent means. ==
== Alpha level is 0.05. ==

----------------------------------------------------------
  Pair     Diff  t-value  df       p   adj.p
----------------------------------------------------------
 b1-b3  -0.0761   1.9251  45  0.0606  0.3633  b1 = b3
 b1-b4  -0.0761   1.7078  45  0.0946  0.4728  b1 = b4
 b1-b2  -0.0652   1.6978  45  0.0964  0.4728  b1 = b2
 b2-b3  -0.0109   0.8135  45  0.4202  1.0000  b2 = b3
 b2-b4  -0.0109   0.5303  45  0.5985  1.0000  b2 = b4
 b3-b4   0.0000   0.0000  45  1.0000  1.0000  b3 = b4
----------------------------------------------------------


< MULTIPLE COMPARISON for "B at a5" >

== Holm's Sequentially Rejective Bonferroni Procedure ==
== The factor < B at a5 > is analysed as dependent means. ==
== Alpha level is 0.05. ==

----------------------------------------------------------
  Pair     Diff  t-value  df       p   adj.p
----------------------------------------------------------
```

b1-b4	-0.1477	2.8985	43	0.0059	0.0353	b1 < b4 *
b1-b3	-0.1307	2.8207	43	0.0072	0.0361	b1 < b3 *
b2-b4	-0.0739	2.6705	43	0.0106	0.0426	b2 < b4 *
b2-b3	-0.0568	1.9490	43	0.0578	0.1735	b2 = b3
b1-b2	-0.0739	1.7626	43	0.0851	0.1735	b1 = b2
b3-b4	-0.0170	0.6841	43	0.4976	0.4976	b3 = b4

output is over --------------------///

[[Simple Effects for c2]]

[AsB-Type Design]

This output was generated by anovakun 4.8.5 under R version 4.0.2.
It was executed on Mon Oct 11 18:22:31 2021.

<< DESCRIPTIVE STATISTICS >>

A	B	n	Mean	S.D.
a1	b1	45	-0.3000	0.5503
a1	b2	45	-0.0944	0.2516
a1	b3	45	-0.0333	0.1735
a1	b4	45	0.0000	0.0754
a2	b1	41	-0.4207	0.4791
a2	b2	41	-0.1280	0.2569
a2	b3	41	-0.0061	0.1044
a2	b4	41	-0.0183	0.1713
a3	b1	46	-0.4185	0.4946
a3	b2	46	-0.1576	0.3389
a3	b3	46	-0.0217	0.2347
a3	b4	46	-0.0109	0.1173
a4	b1	44	-0.2784	0.6057
a4	b2	44	-0.2045	0.3707
a4	b3	44	-0.0398	0.2219
a4	b4	44	0.0227	0.1301
a5	b1	44	-0.7216	0.6630
a5	b2	44	-0.3920	0.5665
a5	b3	44	-0.1307	0.3906
a5	b4	44	-0.0795	0.3270

<< SPHERICITY INDICES >>

== Mendoza's Multisample Sphericity Test and Epsilons ==

Effect	Lambda	approx.Chi	df	p	LB	GG	HF

```
CM
----------------------------------------------------------------------
-----
      B  0.0000    314.2335  29 0.0000 *** 0.3333 0.5796 0.5840
0.5838
----------------------------------------------------------------------
-----
                              LB = lower.bound, GG = Greenhouse-
Geisser
                             HF = Huynh-Feldt-Lecoutre, CM = Chi-
Muller


<< ANOVA TABLE >>

== Adjusted by Greenhouse-Geisser's Epsilon ==
== This data is UNBALANCED!! ==
== Type III SS is applied. ==

--------------------------------------------------------------------
   Source       SS     df      MS  F-ratio  p-value      G.eta^2
--------------------------------------------------------------------
        A   5.8055      4  1.4514   4.5469   0.0015 **    0.0466
    s x A  68.6281    215  0.3192
--------------------------------------------------------------------
        B  23.2320   1.74 13.3599  99.5929   0.0000 ***   0.1636
    A x B   2.8038   6.96  0.4031   3.0049   0.0045 **    0.0231
s x A x B  50.1530 373.87  0.1341
--------------------------------------------------------------------
    Total 150.5898    879  0.1713
                       +p < .10, *p < .05, **p < .01, ***p < .001


<< POST ANALYSES >>

< MULTIPLE COMPARISON for "A" >

== Holm's Sequentially Rejective Bonferroni Procedure ==
== The factor < A > is analysed as independent means. ==
== Alpha level is 0.05. ==

---------------------------
  A   n     Mean    S.D.
---------------------------
 a1 180  -0.1069  0.3353
 a2 164  -0.1433  0.3324
 a3 184  -0.1522  0.3639
 a4 176  -0.1250  0.3937
 a5 176  -0.3310  0.5621
---------------------------

-----------------------------------------------------------
  Pair     Diff  t-value  df       p   adj.p
-----------------------------------------------------------
```

```
 a1-a5   0.2240   3.7405 215  0.0002  0.0024  a1 > a5 *
 a4-a5   0.2060   3.4198 215  0.0007  0.0067  a4 > a5 *
 a2-a5   0.1877   3.0606 215  0.0025  0.0199  a2 > a5 *
 a3-a5   0.1788   3.0014 215  0.0030  0.0210  a3 > a5 *
 a1-a3   0.0452   0.7636 215  0.4459  1.0000  a1 = a3
 a1-a2   0.0363   0.5960 215  0.5518  1.0000  a1 = a2
 a3-a4  -0.0272   0.4562 215  0.6487  1.0000  a3 = a4
 a1-a4   0.0181   0.3015 215  0.7633  1.0000  a1 = a4
 a2-a4  -0.0183   0.2983 215  0.7657  1.0000  a2 = a4
 a2-a3   0.0089   0.1464 215  0.8838  1.0000  a2 = a3
----------------------------------------------------------


< MULTIPLE COMPARISON for "B" >

== Holm's Sequentially Rejective Bonferroni Procedure ==
== The factor < B > is analysed as dependent means. ==
== Alpha level is 0.05. ==

---------------------------
  B   n     Mean    S.D.
---------------------------
 b1 220  -0.4278  0.5802
 b2 220  -0.1953  0.3865
 b3 220  -0.0463  0.2468
 b4 220  -0.0172  0.1866
---------------------------

----------------------------------------------------------
  Pair     Diff  t-value  df       p   adj.p
----------------------------------------------------------
 b1-b4  -0.4106  11.6207 215  0.0000  0.0000  b1 < b4 *
 b1-b3  -0.3815  11.3140 215  0.0000  0.0000  b1 < b3 *
 b2-b3  -0.1490   8.2968 215  0.0000  0.0000  b2 < b3 *
 b2-b4  -0.1781   8.2108 215  0.0000  0.0000  b2 < b4 *
 b1-b2  -0.2325   7.7794 215  0.0000  0.0000  b1 < b2 *
 b3-b4  -0.0291   2.2004 215  0.0288  0.0288  b3 < b4 *
----------------------------------------------------------


< SIMPLE EFFECTS for "A x B" INTERACTION >

--------------------------------------------------------------------
------
  Effect  Lambda  approx.Chi  df      p         LB     GG     HF
CM
--------------------------------------------------------------------
------
 B at a1  0.0000    111.8155   5 0.0000 *** 0.3333 0.4417 0.4502
0.4474
 B at a2  0.0000     61.2685   5 0.0000 *** 0.3333 0.5161 0.5329
0.5290
 B at a3  0.0000     36.2544   5 0.0000 *** 0.3333 0.6605 0.6908
0.6868
```

```
 B at a4  0.0000     53.6609   5 0.0000 *** 0.3333 0.5793 0.6015
0.5977
 B at a5  0.0000     42.7628   5 0.0000 *** 0.3333 0.6551 0.6862
0.6819
--------------------------------------------------------------------
------
                               LB = lower.bound, GG = Greenhouse-
Geisser
                              HF = Huynh-Feldt-Lecoutre, CM = Chi-
Muller

------------------------------------------------------------------
     Source      SS    df      MS  F-ratio  p-value      G.eta^2
------------------------------------------------------------------
    A at b1  5.5207     4 1.3802   4.3516   0.0021 **     0.0749
   Er at b1 68.1906   215 0.3172
------------------------------------------------------------------
    A at b2  2.4155     4 0.6039   4.2841   0.0023 **     0.0738
   Er at b2 30.3050   215 0.1410
------------------------------------------------------------------
    A at b3  0.4167     4 0.1042   1.7339   0.1436 ns     0.0313
   Er at b3 12.9182   215 0.0601
------------------------------------------------------------------
    A at b4  0.2564     4 0.0641   1.8704   0.1167 ns     0.0336
   Er at b4  7.3672   215 0.0343
------------------------------------------------------------------
    B at a1  2.4427  1.33 1.8433  11.1185   0.0005 ***    0.1214
s x B at a1  9.6667 58.31 0.1658
------------------------------------------------------------------
    B at a2  4.5777  1.55 2.9568  23.9641   0.0000 ***    0.2542
s x B at a2  7.6410 61.93 0.1234
------------------------------------------------------------------
    B at a3  4.9647  1.98 2.5054  24.3889   0.0000 ***    0.2048
s x B at a3  9.1603 89.17 0.1027
------------------------------------------------------------------
    B at a4  2.5938  1.74 1.4926   9.6721   0.0004 ***    0.0956
s x B at a4 11.5312 74.73 0.1543
------------------------------------------------------------------
    B at a5 11.4244  1.97 5.8128  40.4194   0.0000 ***    0.2066
s x B at a5 12.1538 84.51 0.1438
------------------------------------------------------------------
                      +p < .10, *p < .05, **p < .01, ***p < .001


< MULTIPLE COMPARISON for "A at b1" >

== Holm's Sequentially Rejective Bonferroni Procedure ==
== The factor < A at b1 > is analysed as independent means. ==
== Alpha level is 0.05. ==

----------------------------------------------------------
  Pair     Diff  t-value  df       p   adj.p
----------------------------------------------------------
 a4-a5   0.4432   3.6911 215  0.0003  0.0028  a4 > a5 *
```

a1-a5	0.4216	3.5309	215	0.0005	0.0046	a1 > a5 *
a3-a5	0.3031	2.5524	215	0.0114	0.0911	a3 = a5
a2-a5	0.3009	2.4611	215	0.0146	0.1025	a2 = a5
a3-a4	-0.1401	1.1795	215	0.2395	1.0000	a3 = a4
a2-a4	-0.1423	1.1642	215	0.2456	1.0000	a2 = a4
a1-a3	0.1185	1.0034	215	0.3168	1.0000	a1 = a3
a1-a2	0.1207	0.9929	215	0.3219	1.0000	a1 = a2
a1-a4	-0.0216	0.1808	215	0.8567	1.0000	a1 = a4
a2-a3	-0.0023	0.0186	215	0.9852	1.0000	a2 = a3

< MULTIPLE COMPARISON for "A at b2" >

== Holm's Sequentially Rejective Bonferroni Procedure ==
== The factor < A at b2 > is analysed as independent means. ==
== Alpha level is 0.05. ==

Pair	Diff	t-value	df	p	adj.p	
a1-a5	0.2976	3.7388	215	0.0002	0.0024	a1 > a5 *
a2-a5	0.2640	3.2394	215	0.0014	0.0125	a2 > a5 *
a3-a5	0.2344	2.9612	215	0.0034	0.0273	a3 > a5 *
a4-a5	0.1875	2.3425	215	0.0201	0.1405	a4 = a5
a1-a4	0.1101	1.3832	215	0.1680	1.0000	a1 = a4
a2-a4	0.0765	0.9387	215	0.3490	1.0000	a2 = a4
a1-a3	0.0632	0.8024	215	0.4232	1.0000	a1 = a3
a3-a4	0.0469	0.5929	215	0.5539	1.0000	a3 = a4
a1-a2	0.0336	0.4146	215	0.6789	1.0000	a1 = a2
a2-a3	0.0296	0.3666	215	0.7143	1.0000	a2 = a3

< MULTIPLE COMPARISON for "B at a1" >

== Holm's Sequentially Rejective Bonferroni Procedure ==
== The factor < B at a1 > is analysed as dependent means. ==
== Alpha level is 0.05. ==

Pair	Diff	t-value	df	p	adj.p	
b1-b4	-0.3000	3.6399	44	0.0007	0.0043	b1 < b4 *
b1-b3	-0.2667	3.5696	44	0.0009	0.0044	b1 < b3 *
b1-b2	-0.2056	2.9619	44	0.0049	0.0197	b1 < b2 *
b2-b3	-0.0611	2.6932	44	0.0100	0.0299	b2 < b3 *
b2-b4	-0.0944	2.6389	44	0.0115	0.0299	b2 < b4 *
b3-b4	-0.0333	1.4306	44	0.1596	0.1596	b3 = b4

< MULTIPLE COMPARISON for "B at a2" >

== Holm's Sequentially Rejective Bonferroni Procedure ==
== The factor < B at a2 > is analysed as dependent means. ==
== Alpha level is 0.05. ==

Pair	Diff	t-value	df	p	adj.p	
b1-b3	-0.4146	5.6124	40	0.0000	0.0000	b1 < b3 *
b1-b4	-0.4024	5.2573	40	0.0000	0.0000	b1 < b4 *
b1-b2	-0.2927	4.8952	40	0.0000	0.0001	b1 < b2 *
b2-b3	-0.1220	3.3758	40	0.0016	0.0049	b2 < b3 *
b2-b4	-0.1098	2.6186	40	0.0124	0.0248	b2 < b4 *
b3-b4	0.0122	0.4669	40	0.6431	0.6431	b3 = b4

< MULTIPLE COMPARISON for "B at a3" >

== Holm's Sequentially Rejective Bonferroni Procedure ==
== The factor < B at a3 > is analysed as dependent means. ==
== Alpha level is 0.05. ==

Pair	Diff	t-value	df	p	adj.p	
b1-b4	-0.4076	5.9637	45	0.0000	0.0000	b1 < b4 *
b1-b3	-0.3967	5.9120	45	0.0000	0.0000	b1 < b3 *
b1-b2	-0.2609	4.1977	45	0.0001	0.0005	b1 < b2 *
b2-b3	-0.1359	3.4485	45	0.0012	0.0037	b2 < b3 *
b2-b4	-0.1467	3.2114	45	0.0024	0.0049	b2 < b4 *
b3-b4	-0.0109	0.3397	45	0.7357	0.7357	b3 = b4

< MULTIPLE COMPARISON for "B at a4" >

== Holm's Sequentially Rejective Bonferroni Procedure ==
== The factor < B at a4 > is analysed as dependent means. ==
== Alpha level is 0.05. ==

Pair	Diff	t-value	df	p	adj.p	
b2-b4	-0.2273	4.4878	43	0.0001	0.0003	b2 < b4 *
b2-b3	-0.1648	3.7046	43	0.0006	0.0030	b2 < b3 *
b1-b4	-0.3011	3.6580	43	0.0007	0.0030	b1 < b4 *
b1-b3	-0.2386	2.8795	43	0.0062	0.0186	b1 < b3 *
b3-b4	-0.0625	2.0459	43	0.0469	0.0938	b3 = b4
b1-b2	-0.0739	1.0187	43	0.3141	0.3141	b1 = b2

< MULTIPLE COMPARISON for "B at a5" >

== Holm's Sequentially Rejective Bonferroni Procedure ==
== The factor < B at a5 > is analysed as dependent means. ==
== Alpha level is 0.05. ==

Pair	Diff	t-value	df	p	adj.p	
b1-b4	-0.6420	7.6221	43	0.0000	0.0000	b1 < b4 *
b1-b3	-0.5909	7.6171	43	0.0000	0.0000	b1 < b3 *
b2-b3	-0.2614	5.0246	43	0.0000	0.0000	b2 < b3 *
b2-b4	-0.3125	4.9175	43	0.0000	0.0000	b2 < b4 *
b1-b2	-0.3295	4.8193	43	0.0000	0.0000	b1 < b2 *
b3-b4	-0.0511	1.5005	43	0.1408	0.1408	b3 = b4

output is over --------------------///

[[Simple Effects for c3]]

[AsB-Type Design]

This output was generated by anovakun 4.8.5 under R version 4.0.2.
It was executed on Mon Oct 11 18:22:32 2021.

<< DESCRIPTIVE STATISTICS >>

A	B	n	Mean	S.D.
a1	b1	45	-0.4778	0.5509
a1	b2	45	-0.2722	0.4018
a1	b3	45	-0.0722	0.2038
a1	b4	45	-0.0278	0.1619
a2	b1	41	-0.5305	0.6782
a2	b2	41	-0.2378	0.3160
a2	b3	41	-0.0183	0.2704
a2	b4	41	-0.0122	0.3352
a3	b1	46	-0.7337	0.7273
a3	b2	46	-0.3152	0.4231
a3	b3	46	-0.2120	0.4624
a3	b4	46	-0.0652	0.2710
a4	b1	44	-0.6080	0.8130
a4	b2	44	-0.3409	0.4944
a4	b3	44	-0.1875	0.3150
a4	b4	44	-0.0568	0.1693
a5	b1	44	-1.0398	0.9384
a5	b2	44	-0.6932	0.7089
a5	b3	44	-0.3920	0.5741
a5	b4	44	-0.2898	0.5730

<< SPHERICITY INDICES >>

== Mendoza's Multisample Sphericity Test and Epsilons ==

Effect	Lambda	approx.Chi	df	p		LB	GG	HF	CM
B	0.0000	241.8911	29	0.0000	***	0.3333	0.6243	0.6296	0.6294

LB = lower.bound, GG = Greenhouse-Geisser
HF = Huynh-Feldt-Lecoutre, CM = Chi-Muller

<< ANOVA TABLE >>

== Adjusted by Greenhouse-Geisser's Epsilon ==
== This data is UNBALANCED!! ==
== Type III SS is applied. ==

Source	SS	df	MS	F-ratio	p-value		G.eta^2
A	18.6307	4	4.6577	7.0625	0.0000	***	0.0749
s x A	141.7905	215	0.6595				
B	44.7761	1.87	23.9084	108.8523	0.0000	***	0.1628
A x B	1.9752	7.49	0.2637	1.2005	0.2993	ns	0.0085
s x A x B	88.4396	402.66	0.2196				
Total	295.7494	879	0.3365				

+p < .10, *p < .05, **p < .01, ***p < .001

<< POST ANALYSES >>

< MULTIPLE COMPARISON for "A" >

== Holm's Sequentially Rejective Bonferroni Procedure ==
== The factor < A > is analysed as independent means. ==
== Alpha level is 0.05. ==

A	n	Mean	S.D.
a1	180	-0.2125	0.4038
a2	164	-0.1997	0.4774
a3	184	-0.3315	0.5539

a4	176	-0.2983	0.5443
a5	176	-0.6037	0.7664

Pair	Diff	t-value	df	p	adj.p		
a2-a5	0.4040	4.5837	215	0.0000	0.0001	a2 > a5	*
a1-a5	0.3912	4.5442	215	0.0000	0.0001	a1 > a5	*
a4-a5	0.3054	3.5278	215	0.0005	0.0041	a4 > a5	*
a3-a5	0.2722	3.1787	215	0.0017	0.0119	a3 > a5	*
a2-a3	0.1318	1.5116	215	0.1321	0.7926	a2 = a3	
a1-a3	0.1190	1.3980	215	0.1635	0.8177	a1 = a3	
a2-a4	0.0986	1.1187	215	0.2645	1.0000	a2 = a4	
a1-a4	0.0858	0.9966	215	0.3201	1.0000	a1 = a4	
a3-a4	-0.0332	0.3881	215	0.6984	1.0000	a3 = a4	
a1-a2	-0.0128	0.1461	215	0.8840	1.0000	a1 = a2	

< MULTIPLE COMPARISON for "B" >

== Holm's Sequentially Rejective Bonferroni Procedure ==
== The factor < B > is analysed as dependent means. ==
== Alpha level is 0.05. ==

B	n	Mean	S.D.
b1	220	-0.6779	0.7724
b2	220	-0.3719	0.5110
b3	220	-0.1764	0.4079
b4	220	-0.0904	0.3482

Pair	Diff	t-value	df	p	adj.p		
b1-b4	-0.5876	12.5319	215	0.0000	0.0000	b1 < b4	*
b1-b3	-0.5015	11.3546	215	0.0000	0.0000	b1 < b3	*
b2-b4	-0.2815	9.4387	215	0.0000	0.0000	b2 < b4	*
b1-b2	-0.3061	8.5004	215	0.0000	0.0000	b1 < b2	*
b2-b3	-0.1955	7.6115	215	0.0000	0.0000	b2 < b3	*
b3-b4	-0.0860	3.8662	215	0.0001	0.0001	b3 < b4	*

output is over --------------------///

[[Simple Effects for c4]]

[AsB-Type Design]

This output was generated by anovakun 4.8.5 under R version 4.0.2.
It was executed on Mon Oct 11 18:22:32 2021.

<< DESCRIPTIVE STATISTICS >>

A	B	n	Mean	S.D.
a1	b1	45	-0.5778	0.7108
a1	b2	45	-0.4000	0.4689
a1	b3	45	-0.1444	0.2743
a1	b4	45	-0.1167	0.2900
a2	b1	41	-0.7134	0.7171
a2	b2	41	-0.5305	0.6713
a2	b3	41	-0.1890	0.3344
a2	b4	41	-0.0244	0.2359
a3	b1	46	-0.9891	0.9617
a3	b2	46	-0.6250	0.5980
a3	b3	46	-0.2065	0.4160
a3	b4	46	-0.0978	0.3139
a4	b1	44	-0.7955	0.9265
a4	b2	44	-0.4602	0.6122
a4	b3	44	-0.3182	0.4649
a4	b4	44	-0.1193	0.3120
a5	b1	44	-1.3409	1.1011
a5	b2	44	-1.0341	0.8134
a5	b3	44	-0.6364	0.7576
a5	b4	44	-0.3693	0.5943

<< SPHERICITY INDICES >>

== Mendoza's Multisample Sphericity Test and Epsilons ==

Effect	Lambda	approx.Chi	df	p		LB	GG	HF	CM
B	0.0000	226.5019	29	0.0000	***	0.3333	0.6381	0.6437	0.6436

LB = lower.bound, GG = Greenhouse-Geisser
HF = Huynh-Feldt-Lecoutre, CM = Chi-Muller

<< ANOVA TABLE >>

== Adjusted by Greenhouse-Geisser's Epsilon ==
== This data is UNBALANCED!! ==
== Type III SS is applied. ==

Source	SS	df	MS	F-ratio	p-value		G.eta^2
A	31.4247	4	7.8562	7.4818	0.0000	***	0.0844
s x A	225.7583	215	1.0500				
B	71.2120	1.91	37.1984	132.7361	0.0000	***	0.1727
A x B	5.1471	7.66	0.6722	2.3985	0.0170	*	0.0149
s x A x B	115.3461	411.59	0.2802				
Total	449.1830	879	0.5110				

+p < .10, *p < .05, **p < .01, ***p < .001

<< POST ANALYSES >>

< MULTIPLE COMPARISON for "A" >

== Holm's Sequentially Rejective Bonferroni Procedure ==
== The factor < A > is analysed as independent means. ==
== Alpha level is 0.05. ==

A	n	Mean	S.D.
a1	180	-0.3097	0.5037
a2	164	-0.3643	0.5935
a3	184	-0.4796	0.7128
a4	176	-0.4233	0.6642
a5	176	-0.8452	0.9094

Pair	Diff	t-value	df	p	adj.p		
a1-a5	0.5354	4.9293	215	0.0000	0.0000	a1 > a5	*
a2-a5	0.4808	4.3235	215	0.0000	0.0002	a2 > a5	*
a4-a5	0.4219	3.8621	215	0.0001	0.0012	a4 > a5	*
a3-a5	0.3656	3.3834	215	0.0009	0.0060	a3 > a5	*
a1-a3	0.1699	1.5815	215	0.1152	0.6914	a1 = a3	
a2-a3	0.1153	1.0477	215	0.2960	1.0000	a2 = a3	
a1-a4	0.1136	1.0455	215	0.2969	1.0000	a1 = a4	
a2-a4	0.0590	0.5302	215	0.5965	1.0000	a2 = a4	
a3-a4	-0.0563	0.5213	215	0.6027	1.0000	a3 = a4	
a1-a2	0.0546	0.4937	215	0.6221	1.0000	a1 = a2	

< MULTIPLE COMPARISON for "B" >

== Holm's Sequentially Rejective Bonferroni Procedure ==
== The factor < B > is analysed as dependent means. ==
== Alpha level is 0.05. ==

B	n	Mean	S.D.
b1	220	-0.8833	0.9288
b2	220	-0.6100	0.6741
b3	220	-0.2989	0.5081
b4	220	-0.1455	0.3865

Pair	Diff	t-value	df	p	adj.p	
b1-b4	-0.7378	13.7046	215	0.0000	0.0000	b1 < b4 *
b2-b4	-0.4645	13.1920	215	0.0000	0.0000	b2 < b4 *
b1-b3	-0.5844	12.0424	215	0.0000	0.0000	b1 < b3 *
b2-b3	-0.3111	10.4774	215	0.0000	0.0000	b2 < b3 *
b1-b2	-0.2734	6.4480	215	0.0000	0.0000	b1 < b2 *
b3-b4	-0.1534	6.2799	215	0.0000	0.0000	b3 < b4 *

< SIMPLE EFFECTS for "A x B" INTERACTION >

Effect	Lambda	approx.Chi	df	p		LB	GG	HF	CM
B at a1	0.0000	30.4731	5	0.0000	***	0.3333	0.6807	0.7142	0.7098
B at a2	0.0000	46.1119	5	0.0000	***	0.3333	0.6321	0.6627	0.6578
B at a3	0.0000	45.5669	5	0.0000	***	0.3333	0.6062	0.6303	0.6266
B at a4	0.0000	44.0283	5	0.0000	***	0.3333	0.5955	0.6196	0.6156
B at a5	0.0000	50.4845	5	0.0000	***	0.3333	0.5563	0.5760	0.5724

LB = lower.bound, GG = Greenhouse-Geisser
HF = Huynh-Feldt-Lecoutre, CM = Chi-Muller

Source	SS	df	MS	F-ratio	p-value		G.eta^2
A at b1	15.4516	4	3.8629	4.7879	0.0010	**	0.0818

Er at b1	173.4629	215	0.8068				
A at b2	11.1546	4	2.7886	6.7854	0.0000	***	0.1121
Er at b2	88.3599	215	0.4110				
A at b3	6.9882	4	1.7471	7.5807	0.0000	***	0.1236
Er at b3	49.5490	215	0.2305				
A at b4	2.9774	4	0.7443	5.3825	0.0004	***	0.0910
Er at b4	29.7326	215	0.1383				
B at a1	6.5066	2.04	3.1862	15.1856	0.0000	***	0.1433
s x B at a1	18.8528	89.85	0.2098				
B at a2	12.1261	1.9	6.3946	27.4006	0.0000	***	0.2112
s x B at a2	17.7020	75.85	0.2334				
B at a3	23.0499	1.82	12.6753	34.4349	0.0000	***	0.2479
s x B at a3	30.1219	81.83	0.3681				
B at a4	10.7060	1.79	5.9929	18.2453	0.0000	***	0.1387
s x B at a4	25.2315	76.82	0.3285				
B at a5	24.2653	1.67	14.5408	44.5180	0.0000	***	0.1677
s x B at a5	23.4379	71.76	0.3266				

+p < .10, *p < .05, **p < .01, ***p < .001

< MULTIPLE COMPARISON for "A at b1" >

== Holm's Sequentially Rejective Bonferroni Procedure ==
== The factor < A at b1 > is analysed as independent means. ==
== Alpha level is 0.05. ==

Pair	Diff	t-value	df	p	adj.p	
a1-a5	0.7631	4.0073	215	0.0001	0.0008	a1 > a5 *
a2-a5	0.6275	3.2184	215	0.0015	0.0134	a2 > a5 *
a4-a5	0.5455	2.8483	215	0.0048	0.0386	a4 > a5 *
a1-a3	0.4114	2.1842	215	0.0300	0.2102	a1 = a3
a3-a5	0.3518	1.8572	215	0.0646	0.3879	a3 = a5
a2-a3	0.2757	1.4292	215	0.1544	0.7720	a2 = a3
a1-a4	0.2177	1.1431	215	0.2543	1.0000	a1 = a4
a3-a4	-0.1937	1.0225	215	0.3077	1.0000	a3 = a4
a1-a2	0.1356	0.6994	215	0.4850	1.0000	a1 = a2
a2-a4	0.0820	0.4208	215	0.6743	1.0000	a2 = a4

< MULTIPLE COMPARISON for "A at b2" >

== Holm's Sequentially Rejective Bonferroni Procedure ==

== The factor < A at b2 > is analysed as independent means. ==
== Alpha level is 0.05. ==

Pair	Diff	t-value	df	p	adj.p	
a1-a5	0.6341	4.6653	215	0.0000	0.0001	a1 > a5 *
a4-a5	0.5739	4.1987	215	0.0000	0.0004	a4 > a5 *
a2-a5	0.5036	3.6190	215	0.0004	0.0029	a2 > a5 *
a3-a5	0.4091	3.0262	215	0.0028	0.0195	a3 > a5 *
a1-a3	0.2250	1.6739	215	0.0956	0.5736	a1 = a3
a3-a4	-0.1648	1.2189	215	0.2242	1.0000	a3 = a4
a1-a2	0.1305	0.9428	215	0.3469	1.0000	a1 = a2
a2-a3	0.0945	0.6864	215	0.4932	1.0000	a2 = a3
a2-a4	-0.0703	0.5049	215	0.6141	1.0000	a2 = a4
a1-a4	0.0602	0.4431	215	0.6581	1.0000	a1 = a4

< MULTIPLE COMPARISON for "A at b3" >

== Holm's Sequentially Rejective Bonferroni Procedure ==
== The factor < A at b3 > is analysed as independent means. ==
== Alpha level is 0.05. ==

Pair	Diff	t-value	df	p	adj.p	
a1-a5	0.4919	4.8332	215	0.0000	0.0000	a1 > a5 *
a2-a5	0.4473	4.2929	215	0.0000	0.0002	a2 > a5 *
a3-a5	0.4298	4.2461	215	0.0000	0.0003	a3 > a5 *
a4-a5	0.3182	3.1088	215	0.0021	0.0149	a4 > a5 *
a1-a4	0.1737	1.7070	215	0.0893	0.5356	a1 = a4
a2-a4	0.1292	1.2395	215	0.2165	1.0000	a2 = a4
a3-a4	0.1117	1.1030	215	0.2713	1.0000	a3 = a4
a1-a3	0.0621	0.6167	215	0.5381	1.0000	a1 = a3
a1-a2	0.0446	0.4301	215	0.6675	1.0000	a1 = a2
a2-a3	0.0175	0.1697	215	0.8654	1.0000	a2 = a3

< MULTIPLE COMPARISON for "A at b4" >

== Holm's Sequentially Rejective Bonferroni Procedure ==
== The factor < A at b4 > is analysed as independent means. ==
== Alpha level is 0.05. ==

Pair	Diff	t-value	df	p	adj.p	
a2-a5	0.3449	4.2731	215	0.0000	0.0003	a2 > a5 *
a3-a5	0.2715	3.4621	215	0.0006	0.0058	a3 > a5 *
a1-a5	0.2527	3.2045	215	0.0016	0.0125	a1 > a5 *
a4-a5	0.2500	3.1532	215	0.0018	0.0129	a4 > a5 *

```
 a2-a4   0.0949   1.1760 215  0.2409  1.0000  a2 = a4
 a1-a2  -0.0923   1.1493 215  0.2517  1.0000  a1 = a2
 a2-a3   0.0734   0.9194 215  0.3589  1.0000  a2 = a3
 a3-a4   0.0215   0.2741 215  0.7843  1.0000  a3 = a4
 a1-a3  -0.0188   0.2416 215  0.8093  1.0000  a1 = a3
 a1-a4   0.0027   0.0336 215  0.9732  1.0000  a1 = a4
----------------------------------------------------------


< MULTIPLE COMPARISON for "B at a1" >

== Holm's Sequentially Rejective Bonferroni Procedure ==
== The factor < B at a1 > is analysed as dependent means. ==
== Alpha level is 0.05. ==

----------------------------------------------------------
  Pair     Diff  t-value  df       p   adj.p
----------------------------------------------------------
 b1-b3  -0.4333   4.6192  44  0.0000  0.0002  b1 < b3 *
 b1-b4  -0.4611   4.4001  44  0.0001  0.0003  b1 < b4 *
 b2-b3  -0.2556   4.3572  44  0.0001  0.0003  b2 < b3 *
 b2-b4  -0.2833   3.7057  44  0.0006  0.0018  b2 < b4 *
 b1-b2  -0.1778   2.3249  44  0.0248  0.0495  b1 < b2 *
 b3-b4  -0.0278   0.4933  44  0.6242  0.6242  b3 = b4
----------------------------------------------------------


< MULTIPLE COMPARISON for "B at a2" >

== Holm's Sequentially Rejective Bonferroni Procedure ==
== The factor < B at a2 > is analysed as dependent means. ==
== Alpha level is 0.05. ==

----------------------------------------------------------
  Pair     Diff  t-value  df       p   adj.p
----------------------------------------------------------
 b1-b4  -0.6890   6.4704  40  0.0000  0.0000  b1 < b4 *
 b2-b4  -0.5061   5.6711  40  0.0000  0.0000  b2 < b4 *
 b1-b3  -0.5244   5.2121  40  0.0000  0.0000  b1 < b3 *
 b2-b3  -0.3415   4.5236  40  0.0001  0.0002  b2 < b3 *
 b3-b4  -0.1646   4.0566  40  0.0002  0.0004  b3 < b4 *
 b1-b2  -0.1829   2.2858  40  0.0276  0.0276  b1 < b2 *
----------------------------------------------------------


< MULTIPLE COMPARISON for "B at a3" >

== Holm's Sequentially Rejective Bonferroni Procedure ==
== The factor < B at a3 > is analysed as dependent means. ==
== Alpha level is 0.05. ==

----------------------------------------------------------
  Pair     Diff  t-value  df       p   adj.p
----------------------------------------------------------
```

Pair	Diff	t-value	df	p	adj.p	
b2-b3	-0.4185	6.9491	45	0.0000	0.0000	b2 < b3 *
b1-b4	-0.8913	6.7745	45	0.0000	0.0000	b1 < b4 *
b2-b4	-0.5272	6.5821	45	0.0000	0.0000	b2 < b4 *
b1-b3	-0.7826	6.4393	45	0.0000	0.0000	b1 < b3 *
b1-b2	-0.3641	3.2671	45	0.0021	0.0042	b1 < b2 *
b3-b4	-0.1087	1.7961	45	0.0792	0.0792	b3 = b4

< MULTIPLE COMPARISON for "B at a4" >

== Holm's Sequentially Rejective Bonferroni Procedure ==
== The factor < B at a4 > is analysed as dependent means. ==
== Alpha level is 0.05. ==

Pair	Diff	t-value	df	p	adj.p	
b2-b4	-0.3409	5.6001	43	0.0000	0.0000	b2 < b4 *
b1-b4	-0.6761	5.4082	43	0.0000	0.0000	b1 < b4 *
b1-b3	-0.4773	4.2069	43	0.0001	0.0005	b1 < b3 *
b3-b4	-0.1989	3.5469	43	0.0010	0.0029	b3 < b4 *
b1-b2	-0.3352	2.9240	43	0.0055	0.0110	b1 < b2 *
b2-b3	-0.1420	2.0398	43	0.0475	0.0475	b2 < b3 *

< MULTIPLE COMPARISON for "B at a5" >

== Holm's Sequentially Rejective Bonferroni Procedure ==
== The factor < B at a5 > is analysed as dependent means. ==
== Alpha level is 0.05. ==

Pair	Diff	t-value	df	p	adj.p	
b2-b4	-0.6648	7.8357	43	0.0000	0.0000	b2 < b4 *
b1-b4	-0.9716	7.5718	43	0.0000	0.0000	b1 < b4 *
b1-b3	-0.7045	6.4919	43	0.0000	0.0000	b1 < b3 *
b2-b3	-0.3977	5.8352	43	0.0000	0.0000	b2 < b3 *
b3-b4	-0.2670	4.8761	43	0.0000	0.0000	b3 < b4 *
b1-b2	-0.3068	3.8157	43	0.0004	0.0004	b1 < b2 *

output is over --------------------///

[[Simple Effects for c5]]

[AsB-Type Design]

This output was generated by anovakun 4.8.5 under R version 4.0.2.
It was executed on Mon Oct 11 18:22:33 2021.

<< DESCRIPTIVE STATISTICS >>

A	B	n	Mean	S.D.
a1	b1	45	-0.9000	0.7984
a1	b2	45	-0.5278	0.5671
a1	b3	45	-0.2278	0.3946
a1	b4	45	-0.1056	0.2528
a2	b1	41	-1.0610	0.8344
a2	b2	41	-0.6585	0.6040
a2	b3	41	-0.2988	0.4944
a2	b4	41	-0.1463	0.3831
a3	b1	46	-1.5652	1.0832
a3	b2	46	-0.9402	0.6671
a3	b3	46	-0.4022	0.5413
a3	b4	46	-0.2880	0.5296
a4	b1	44	-1.0341	0.9684
a4	b2	44	-0.8068	0.8758
a4	b3	44	-0.5398	0.6170
a4	b4	44	-0.2955	0.5037
a5	b1	44	-1.8750	1.2912
a5	b2	44	-1.3352	1.0633
a5	b3	44	-0.8636	0.8499
a5	b4	44	-0.5795	0.7601

<< SPHERICITY INDICES >>

== Mendoza's Multisample Sphericity Test and Epsilons ==

Effect	Lambda	approx.Chi	df	p		LB	GG	HF	CM
B	0.0000	262.8179	29	0.0000	***	0.3333	0.6465	0.6523	0.6521

LB = lower.bound, GG = Greenhouse-Geisser
HF = Huynh-Feldt-Lecoutre, CM = Chi-Muller

<< ANOVA TABLE >>

== Adjusted by Greenhouse-Geisser's Epsilon ==
== This data is UNBALANCED!! ==
== Type III SS is applied. ==

Source	SS	df	MS	F-ratio	p-value		G.eta^2
A	55.5099	4	13.8775	9.1495	0.0000	***	0.1026
s x A	326.1023	215	1.5168				
B	130.6356	1.94	67.3548	176.3333	0.0000	***	0.2121
A x B	9.0363	7.76	1.1648	3.0493	0.0027	**	0.0183
s x A x B	159.2816	417	0.3820				
Total	681.5497	879	0.7754				

+p < .10, *p < .05, **p < .01, ***p < .001

<< POST ANALYSES >>

< MULTIPLE COMPARISON for "A" >

== Holm's Sequentially Rejective Bonferroni Procedure ==
== The factor < A > is analysed as independent means. ==
== Alpha level is 0.05. ==

A	n	Mean	S.D.
a1	180	-0.4403	0.6199
a2	164	-0.5412	0.6941
a3	184	-0.7989	0.8926
a4	176	-0.6690	0.8077
a5	176	-1.1634	1.1180

Pair	Diff	t-value	df	p	adj.p	
a1-a5	0.7231	5.5385	215	0.0000	0.0000	a1 > a5 *
a2-a5	0.6222	4.6549	215	0.0000	0.0001	a2 > a5 *
a4-a5	0.4943	3.7652	215	0.0002	0.0017	a4 > a5 *
a3-a5	0.3644	2.8066	215	0.0055	0.0383	a3 > a5 *
a1-a3	0.3586	2.7777	215	0.0060	0.0383	a1 > a3 *
a2-a3	0.2578	1.9489	215	0.0526	0.2630	a2 = a3
a1-a4	0.2288	1.7522	215	0.0812	0.3247	a1 = a4
a3-a4	-0.1299	1.0002	215	0.3183	0.9550	a3 = a4
a2-a4	0.1279	0.9567	215	0.3398	0.9550	a2 = a4
a1-a2	0.1009	0.7588	215	0.4488	0.9550	a1 = a2

< MULTIPLE COMPARISON for "B" >

== Holm's Sequentially Rejective Bonferroni Procedure ==
== The factor < B > is analysed as dependent means. ==
== Alpha level is 0.05. ==

B	n	Mean	S.D.
b1	220	-1.2871	1.0709
b2	220	-0.8537	0.8203
b3	220	-0.4664	0.6351
b4	220	-0.2830	0.5369

Pair	Diff	t-value	df	p	adj.p	
b1-b4	-1.0041	16.0627	215	0.0000	0.0000	b1 < b4 *
b1-b3	-0.8206	14.4569	215	0.0000	0.0000	b1 < b3 *
b2-b4	-0.5707	12.7956	215	0.0000	0.0000	b2 < b4 *
b2-b3	-0.3873	10.6975	215	0.0000	0.0000	b2 < b3 *
b1-b2	-0.4333	9.1149	215	0.0000	0.0000	b1 < b2 *
b3-b4	-0.1834	6.4854	215	0.0000	0.0000	b3 < b4 *

< SIMPLE EFFECTS for "A x B" INTERACTION >

Effect	Lambda	approx.Chi	df	p		LB	GG	HF	CM
B at a1	0.0000	45.1779	5	0.0000	***	0.3333	0.5842	0.6065	0.6029
B at a2	0.0000	50.1290	5	0.0000	***	0.3333	0.5426	0.5624	0.5583
B at a3	0.0000	35.6414	5	0.0000	***	0.3333	0.6672	0.6983	0.6943
B at a4	0.0000	39.1771	5	0.0000	***	0.3333	0.6820	0.7164	0.7119
B at a5	0.0000	62.2948	5	0.0000	***	0.3333	0.5267	0.5434	0.5400

LB = lower.bound, GG = Greenhouse-Geisser
HF = Huynh-Feldt-Lecoutre, CM = Chi-Muller

Source	SS	df	MS	F-ratio	p-value		G.eta^2
A at b1	30.4185	4	7.6046	7.4078	0.0000	***	0.1211
Er at b1	220.7133	215	1.0266				
A at b2	16.9842	4	4.2460	7.0023	0.0000	***	0.1153

Er at b2	130.3712	215	0.6064				
A at b3	11.0839	4	2.7710	7.7131	0.0000	***	0.1255
Er at b3	77.2397	215	0.3593				
A at b4	6.0597	4	1.5149	5.7082	0.0002	***	0.0960
Er at b4	57.0597	215	0.2654				
B at a1	16.9288	1.75	9.6585	41.7602	0.0000	***	0.2461
s x B at a1	17.8368	77.12	0.2313				
B at a2	20.4432	1.63	12.5585	33.0681	0.0000	***	0.2603
s x B at a2	24.7287	65.11	0.3798				
B at a3	47.1766	2	23.5682	47.9983	0.0000	***	0.3235
s x B at a3	44.2296	90.08	0.4910				
B at a4	13.5749	2.05	6.6351	15.1850	0.0000	***	0.1189
s x B at a4	38.4407	87.98	0.4369				
B at a5	42.5323	1.58	26.9168	53.7185	0.0000	***	0.1944
s x B at a5	34.0458	67.95	0.5011				

+p < .10, *p < .05, **p < .01, ***p < .001

< MULTIPLE COMPARISON for "A at b1" >

== Holm's Sequentially Rejective Bonferroni Procedure ==
== The factor < A at b1 > is analysed as independent means. ==
== Alpha level is 0.05. ==

Pair	Diff	t-value	df	p	adj.p	
a1-a5	0.9750	4.5389	215	0.0000	0.0001	a1 > a5 *
a4-a5	0.8409	3.8928	215	0.0001	0.0012	a4 > a5 *
a2-a5	0.8140	3.7013	215	0.0003	0.0022	a2 > a5 *
a1-a3	0.6652	3.1314	215	0.0020	0.0139	a1 > a3 *
a3-a4	-0.5311	2.4859	215	0.0137	0.0821	a3 = a4
a2-a3	0.5042	2.3172	215	0.0214	0.1072	a2 = a3
a3-a5	0.3098	1.4499	215	0.1485	0.5941	a3 = a5
a1-a2	0.1610	0.7359	215	0.4626	1.0000	a1 = a2
a1-a4	0.1341	0.6242	215	0.5331	1.0000	a1 = a4
a2-a4	-0.0269	0.1222	215	0.9028	1.0000	a2 = a4

< MULTIPLE COMPARISON for "A at b2" >

== Holm's Sequentially Rejective Bonferroni Procedure ==
== The factor < A at b2 > is analysed as independent means. ==
== Alpha level is 0.05. ==

Pair	Diff	t-value	df	p	adj.p	
a1-a5	0.8074	4.8908	215	0.0000	0.0000	a1 > a5 *
a2-a5	0.6767	4.0034	215	0.0001	0.0008	a2 > a5 *
a4-a5	0.5284	3.1828	215	0.0017	0.0134	a4 > a5 *
a1-a3	0.4124	2.5261	215	0.0123	0.0858	a1 = a3
a3-a5	0.3950	2.4056	215	0.0170	0.1020	a3 = a5
a1-a4	0.2790	1.6902	215	0.0924	0.4622	a1 = a4
a2-a3	0.2817	1.6842	215	0.0936	0.4622	a2 = a3
a2-a4	0.1483	0.8773	215	0.3813	1.0000	a2 = a4
a3-a4	-0.1334	0.8124	215	0.4175	1.0000	a3 = a4
a1-a2	0.1308	0.7778	215	0.4376	1.0000	a1 = a2

< MULTIPLE COMPARISON for "A at b3" >

== Holm's Sequentially Rejective Bonferroni Procedure ==
== The factor < A at b3 > is analysed as independent means. ==
== Alpha level is 0.05. ==

Pair	Diff	t-value	df	p	adj.p	
a1-a5	0.6359	5.0038	215	0.0000	0.0000	a1 > a5 *
a2-a5	0.5649	4.3416	215	0.0000	0.0002	a2 > a5 *
a3-a5	0.4615	3.6511	215	0.0003	0.0026	a3 > a5 *
a4-a5	0.3239	2.5344	215	0.0120	0.0838	a4 = a5
a1-a4	0.3120	2.4552	215	0.0149	0.0893	a1 = a4
a2-a4	0.2410	1.8523	215	0.0654	0.3268	a2 = a4
a1-a3	0.1744	1.3877	215	0.1667	0.6666	a1 = a3
a3-a4	0.1376	1.0887	215	0.2775	0.8326	a3 = a4
a2-a3	0.1034	0.8032	215	0.4228	0.8455	a2 = a3
a1-a2	0.0710	0.5487	215	0.5838	0.8455	a1 = a2

< MULTIPLE COMPARISON for "A at b4" >

== Holm's Sequentially Rejective Bonferroni Procedure ==
== The factor < A at b4 > is analysed as independent means. ==
== Alpha level is 0.05. ==

Pair	Diff	t-value	df	p	adj.p	
a1-a5	0.4740	4.3397	215	0.0000	0.0002	a1 > a5 *
a2-a5	0.4332	3.8740	215	0.0001	0.0013	a2 > a5 *
a3-a5	0.2915	2.6834	215	0.0079	0.0628	a3 = a5
a4-a5	0.2841	2.5866	215	0.0104	0.0725	a4 = a5
a1-a4	0.1899	1.7387	215	0.0835	0.5012	a1 = a4
a1-a3	0.1825	1.6895	215	0.0926	0.5012	a1 = a3
a2-a4	0.1491	1.3335	215	0.1838	0.7352	a2 = a4

```
 a2-a3  0.1417   1.2807 215  0.2017  0.7352  a2 = a3
 a1-a2  0.0408   0.3667 215  0.7142  1.0000  a1 = a2
 a3-a4  0.0074   0.0682 215  0.9457  1.0000  a3 = a4
--------------------------------------------------------


< MULTIPLE COMPARISON for "B at a1" >

== Holm's Sequentially Rejective Bonferroni Procedure ==
== The factor < B at a1 > is analysed as dependent means. ==
== Alpha level is 0.05. ==

---------------------------------------------------------
  Pair     Diff  t-value  df       p   adj.p
---------------------------------------------------------
 b1-b4  -0.7944   7.4466  44  0.0000  0.0000  b1 < b4 *
 b1-b3  -0.6722   7.1806  44  0.0000  0.0000  b1 < b3 *
 b2-b4  -0.4222   5.7698  44  0.0000  0.0000  b2 < b4 *
 b1-b2  -0.3722   5.4598  44  0.0000  0.0000  b1 < b2 *
 b2-b3  -0.3000   5.0636  44  0.0000  0.0000  b2 < b3 *
 b3-b4  -0.1222   2.5079  44  0.0159  0.0159  b3 < b4 *
---------------------------------------------------------


< MULTIPLE COMPARISON for "B at a2" >

== Holm's Sequentially Rejective Bonferroni Procedure ==
== The factor < B at a2 > is analysed as dependent means. ==
== Alpha level is 0.05. ==

---------------------------------------------------------
  Pair     Diff  t-value  df       p   adj.p
---------------------------------------------------------
 b1-b4  -0.9146   6.6444  40  0.0000  0.0000  b1 < b4 *
 b1-b3  -0.7622   6.0396  40  0.0000  0.0000  b1 < b3 *
 b2-b4  -0.5122   5.4720  40  0.0000  0.0000  b2 < b4 *
 b1-b2  -0.4024   4.7831  40  0.0000  0.0001  b1 < b2 *
 b2-b3  -0.3598   4.5218  40  0.0001  0.0001  b2 < b3 *
 b3-b4  -0.1524   2.6665  40  0.0110  0.0110  b3 < b4 *
---------------------------------------------------------


< MULTIPLE COMPARISON for "B at a3" >

== Holm's Sequentially Rejective Bonferroni Procedure ==
== The factor < B at a3 > is analysed as dependent means. ==
== Alpha level is 0.05. ==

---------------------------------------------------------
  Pair     Diff  t-value  df       p   adj.p
---------------------------------------------------------
 b1-b3  -1.1630   8.4567  45  0.0000  0.0000  b1 < b3 *
 b1-b4  -1.2772   8.0559  45  0.0000  0.0000  b1 < b4 *
 b2-b3  -0.5380   7.1049  45  0.0000  0.0000  b2 < b3 *
```

b2-b4	-0.6522	6.1458	45	0.0000	0.0000	b2 < b4 *
b1-b2	-0.6250	4.6927	45	0.0000	0.0001	b1 < b2 *
b3-b4	-0.1141	1.3959	45	0.1696	0.1696	b3 = b4

< MULTIPLE COMPARISON for "B at a4" >

== Holm's Sequentially Rejective Bonferroni Procedure ==
== The factor < B at a4 > is analysed as dependent means. ==
== Alpha level is 0.05. ==

Pair	Diff	t-value	df	p	adj.p	
b1-b4	-0.7386	5.5411	43	0.0000	0.0000	b1 < b4 *
b2-b4	-0.5114	5.0540	43	0.0000	0.0000	b2 < b4 *
b3-b4	-0.2443	4.1099	43	0.0002	0.0007	b3 < b4 *
b1-b3	-0.4943	3.3709	43	0.0016	0.0048	b1 < b3 *
b2-b3	-0.2670	2.7247	43	0.0093	0.0185	b2 < b3 *
b1-b2	-0.2273	1.6656	43	0.1031	0.1031	b1 = b2

< MULTIPLE COMPARISON for "B at a5" >

== Holm's Sequentially Rejective Bonferroni Procedure ==
== The factor < B at a5 > is analysed as dependent means. ==
== Alpha level is 0.05. ==

Pair	Diff	t-value	df	p	adj.p	
b1-b4	-1.2955	8.3736	43	0.0000	0.0000	b1 < b4 *
b1-b3	-1.0114	8.1875	43	0.0000	0.0000	b1 < b3 *
b2-b4	-0.7557	6.4081	43	0.0000	0.0000	b2 < b4 *
b1-b2	-0.5398	6.2765	43	0.0000	0.0000	b1 < b2 *
b2-b3	-0.4716	5.3819	43	0.0000	0.0000	b2 < b3 *
b3-b4	-0.2841	4.6056	43	0.0000	0.0000	b3 < b4 *

output is over --------------------///

<Simple effect>

[[Simple Effects for a1]]

[sAB-Type Design]

This output was generated by anovakun 4.8.5 under R version 4.0.2.
It was executed on Mon Oct 11 18:22:23 2021.

<< DESCRIPTIVE STATISTICS >>

B	C	n	Mean	S.D.
b1	c1	45	-0.0222	0.2601
b1	c2	45	-0.3000	0.5503
b1	c3	45	-0.4778	0.5509
b1	c4	45	-0.5778	0.7108
b1	c5	45	-0.9000	0.7984
b2	c1	45	-0.0111	0.1406
b2	c2	45	-0.0944	0.2516
b2	c3	45	-0.2722	0.4018
b2	c4	45	-0.4000	0.4689
b2	c5	45	-0.5278	0.5671
b3	c1	45	-0.0111	0.1187
b3	c2	45	-0.0333	0.1735
b3	c3	45	-0.0722	0.2038
b3	c4	45	-0.1444	0.2743
b3	c5	45	-0.2278	0.3946
b4	c1	45	0.0000	0.0000
b4	c2	45	0.0000	0.0754
b4	c3	45	-0.0278	0.1619
b4	c4	45	-0.1167	0.2900
b4	c5	45	-0.1056	0.2528

<< SPHERICITY INDICES >>

== Mendoza's Multisample Sphericity Test and Epsilons ==

Effect	Lambda	approx.Chi	df	p		LB	GG	HF	CM
Global	0.0000	896.5969	189	0.0000	***	0.0526	0.1850	0.2030	0.2018
B	0.0000	99.6523	5	0.0000	***	0.3333	0.4594	0.4693	0.4665
C	0.0000	84.3064	9	0.0000	***	0.2500	0.4780	0.4992	0.4962
B x C	0.0000	233.9413	77	0.0000	***	0.0833	0.5507	0.6583	0.6543

LB = lower.bound, GG = Greenhouse-

Geisser
HF = Huynh-Feldt-Lecoutre, CM = Chi-
Muller

<< ANOVA TABLE >>

== Adjusted by Greenhouse-Geisser's Epsilon ==

Source	SS	df	MS	F-ratio	p-value		G.eta^2
s	49.7726	44	1.1312				
B	22.7147	1.38	16.4831	32.3928	0.0000	***	0.1428
s x B	30.8540	60.63	0.5089				
C	20.3344	1.91	10.6343	35.0226	0.0000	***	0.1298
s x C	25.5468	84.13	0.3036				
B x C	8.9228	6.61	1.3503	13.0018	0.0000	***	0.0614
s x B x C	30.1960	290.75	0.1039				
Total	188.3414	899	0.2095				

+p < .10, *p < .05, **p < .01, ***p < .001

<< POST ANALYSES >>

< MULTIPLE COMPARISON for "B" >

== Holm's Sequentially Rejective Bonferroni Procedure ==
== The factor < B > is analysed as dependent means. ==
== Alpha level is 0.05. ==

B	n	Mean	S.D.
b1	225	-0.4556	0.6650
b2	225	-0.2611	0.4367
b3	225	-0.0978	0.2618
b4	225	-0.0500	0.1948

Pair	Diff	t-value	df	p	adj.p	
b2-b3	-0.1633	6.3694	44	0.0000	0.0000	b2 < b3 *
b1-b3	-0.3578	6.1830	44	0.0000	0.0000	b1 < b3 *
b1-b4	-0.4056	5.9917	44	0.0000	0.0000	b1 < b4 *
b2-b4	-0.2111	5.2152	44	0.0000	0.0000	b2 < b4 *
b1-b2	-0.1944	4.7949	44	0.0000	0.0000	b1 < b2 *
b3-b4	-0.0478	1.9573	44	0.0567	0.0567	b3 = b4

< MULTIPLE COMPARISON for "C" >

== Holm's Sequentially Rejective Bonferroni Procedure ==
== The factor < C > is analysed as dependent means. ==
== Alpha level is 0.05. ==

C	n	Mean	S.D.
c1	180	-0.0111	0.1582
c2	180	-0.1069	0.3353
c3	180	-0.2125	0.4038
c4	180	-0.3097	0.5037
c5	180	-0.4403	0.6199

Pair	Diff	t-value	df	p	adj.p	
c2-c5	0.3333	7.0288	44	0.0000	0.0000	c2 > c5 *
c1-c5	0.4292	6.8439	44	0.0000	0.0000	c1 > c5 *
c1-c4	0.2986	6.2106	44	0.0000	0.0000	c1 > c4 *
c1-c3	0.2014	6.0601	44	0.0000	0.0000	c1 > c3 *
c3-c5	0.2278	5.7979	44	0.0000	0.0000	c3 > c5 *
c2-c4	0.2028	5.6875	44	0.0000	0.0000	c2 > c4 *
c2-c3	0.1056	4.3414	44	0.0001	0.0003	c2 > c3 *
c4-c5	0.1306	3.7832	44	0.0005	0.0014	c4 > c5 *
c1-c2	0.0958	3.5130	44	0.0010	0.0021	c1 > c2 *
c3-c4	0.0972	2.8185	44	0.0072	0.0072	c3 > c4 *

< SIMPLE EFFECTS for "B x C" INTERACTION >

Effect	Lambda	approx.Chi	df	p		LB	GG	HF	CM
B at c1	0.0000	55.2157	5	0.0000	***	0.3333	0.5477	0.5661	0.5627
B at c2	0.0000	111.8155	5	0.0000	***	0.3333	0.4417	0.4502	0.4474
B at c3	0.0000	48.8540	5	0.0000	***	0.3333	0.6376	0.6659	0.6619
B at c4	0.0000	30.4731	5	0.0000	***	0.3333	0.6807	0.7142	0.7098
B at c5	0.0000	45.1779	5	0.0000	***	0.3333	0.5842	0.6065	0.6029
C at b1	0.0000	38.8624	9	0.0000	***	0.2500	0.6910	0.7419	0.7374

```
 C at b2  0.0000     61.5209   9 0.0000 *** 0.2500 0.5984 0.6352
0.6313
 C at b3  0.0000     49.2256   9 0.0000 *** 0.2500 0.5887 0.6241
0.6203
 C at b4  0.0000     93.6185   9 0.0000 *** 0.2500 0.6169 0.6564
0.6524
--------------------------------------------------------------------
------
                               LB = lower.bound, GG = Greenhouse-
Geisser
                              HF = Huynh-Feldt-Lecoutre, CM = Chi-
Muller

-------------------------------------------------------------------
     Source       SS      df      MS  F-ratio  p-value      G.eta^2
-------------------------------------------------------------------
    B at c1  0.0111    1.64 0.0068   0.1870   0.7868 ns    0.0025
s x B at c1  2.6139    72.3 0.0362
-------------------------------------------------------------------
    B at c2  2.4427    1.33 1.8433  11.1185   0.0005 ***   0.1214
s x B at c2  9.6667   58.31 0.1658
-------------------------------------------------------------------
    B at c3  5.7483    1.91 3.0051  20.9376   0.0000 ***   0.1970
s x B at c3 12.0799   84.17 0.1435
-------------------------------------------------------------------
    B at c4  6.5066    2.04 3.1862  15.1856   0.0000 ***   0.1433
s x B at c4 18.8528   89.85 0.2098
-------------------------------------------------------------------
    B at c5 16.9288    1.75 9.6585  41.7602   0.0000 ***   0.2461
s x B at c5 17.8368   77.12 0.2313
-------------------------------------------------------------------
    C at b1 19.1222    2.76 6.9186  31.0440   0.0000 ***   0.1930
s x C at b1 27.1028  121.61 0.2229
-------------------------------------------------------------------
    C at b2  8.1361    2.39 3.3991  23.1500   0.0000 ***   0.1904
s x C at b2 15.4639  105.32 0.1468
-------------------------------------------------------------------
    C at b3  1.4128    2.35 0.6000   8.6792   0.0001 ***   0.0920
s x C at b3  7.1622  103.61 0.0691
-------------------------------------------------------------------
    C at b4  0.5861    2.47 0.2375   4.2882   0.0106 *     0.0690
s x C at b4  6.0139  108.57 0.0554
-------------------------------------------------------------------
                       +p < .10, *p < .05, **p < .01, ***p < .001


< MULTIPLE COMPARISON for "B at c2" >

== Holm's Sequentially Rejective Bonferroni Procedure ==
== The factor < B at c2 > is analysed as dependent means. ==
== Alpha level is 0.05. ==

----------------------------------------------------------
  Pair     Diff  t-value  df       p   adj.p
```

b1-b4	-0.3000	3.6399	44	0.0007	0.0043	b1 < b4 *
b1-b3	-0.2667	3.5696	44	0.0009	0.0044	b1 < b3 *
b1-b2	-0.2056	2.9619	44	0.0049	0.0197	b1 < b2 *
b2-b3	-0.0611	2.6932	44	0.0100	0.0299	b2 < b3 *
b2-b4	-0.0944	2.6389	44	0.0115	0.0299	b2 < b4 *
b3-b4	-0.0333	1.4306	44	0.1596	0.1596	b3 = b4

< MULTIPLE COMPARISON for "B at c3" >

== Holm's Sequentially Rejective Bonferroni Procedure ==
== The factor < B at c3 > is analysed as dependent means. ==
== Alpha level is 0.05. ==

Pair	Diff	t-value	df	p	adj.p	
b1-b4	-0.4500	5.4347	44	0.0000	0.0000	b1 < b4 *
b1-b3	-0.4056	5.2846	44	0.0000	0.0000	b1 < b3 *
b2-b3	-0.2000	4.3425	44	0.0001	0.0003	b2 < b3 *
b2-b4	-0.2444	3.7449	44	0.0005	0.0016	b2 < b4 *
b1-b2	-0.2056	3.3319	44	0.0018	0.0035	b1 < b2 *
b3-b4	-0.0444	1.1591	44	0.2527	0.2527	b3 = b4

< MULTIPLE COMPARISON for "B at c4" >

== Holm's Sequentially Rejective Bonferroni Procedure ==
== The factor < B at c4 > is analysed as dependent means. ==
== Alpha level is 0.05. ==

Pair	Diff	t-value	df	p	adj.p	
b1-b3	-0.4333	4.6192	44	0.0000	0.0002	b1 < b3 *
b1-b4	-0.4611	4.4001	44	0.0001	0.0003	b1 < b4 *
b2-b3	-0.2556	4.3572	44	0.0001	0.0003	b2 < b3 *
b2-b4	-0.2833	3.7057	44	0.0006	0.0018	b2 < b4 *
b1-b2	-0.1778	2.3249	44	0.0248	0.0495	b1 < b2 *
b3-b4	-0.0278	0.4933	44	0.6242	0.6242	b3 = b4

< MULTIPLE COMPARISON for "B at c5" >

== Holm's Sequentially Rejective Bonferroni Procedure ==
== The factor < B at c5 > is analysed as dependent means. ==
== Alpha level is 0.05. ==

Pair	Diff	t-value	df	p	adj.p

b1-b4	-0.7944	7.4466	44	0.0000	0.0000	b1 < b4 *
b1-b3	-0.6722	7.1806	44	0.0000	0.0000	b1 < b3 *
b2-b4	-0.4222	5.7698	44	0.0000	0.0000	b2 < b4 *
b1-b2	-0.3722	5.4598	44	0.0000	0.0000	b1 < b2 *
b2-b3	-0.3000	5.0636	44	0.0000	0.0000	b2 < b3 *
b3-b4	-0.1222	2.5079	44	0.0159	0.0159	b3 < b4 *

< MULTIPLE COMPARISON for "C at b1" >

== Holm's Sequentially Rejective Bonferroni Procedure ==
== The factor < C at b1 > is analysed as dependent means. ==
== Alpha level is 0.05. ==

Pair	Diff	t-value	df	p	adj.p	
c1-c5	0.8778	8.0016	44	0.0000	0.0000	c1 > c5 *
c2-c5	0.6000	7.2529	44	0.0000	0.0000	c2 > c5 *
c1-c3	0.4556	6.5217	44	0.0000	0.0000	c1 > c3 *
c3-c5	0.4222	6.0237	44	0.0000	0.0000	c3 > c5 *
c1-c4	0.5556	5.1844	44	0.0000	0.0000	c1 > c4 *
c4-c5	0.3222	3.9839	44	0.0003	0.0013	c4 > c5 *
c1-c2	0.2778	3.7650	44	0.0005	0.0020	c1 > c2 *
c2-c3	0.1778	3.3877	44	0.0015	0.0045	c2 > c3 *
c2-c4	0.2778	3.2856	44	0.0020	0.0045	c2 > c4 *
c3-c4	0.1000	1.2499	44	0.2180	0.2180	c3 = c4

< MULTIPLE COMPARISON for "C at b2" >

== Holm's Sequentially Rejective Bonferroni Procedure ==
== The factor < C at b2 > is analysed as dependent means. ==
== Alpha level is 0.05. ==

Pair	Diff	t-value	df	p	adj.p	
c1-c5	0.5167	6.0014	44	0.0000	0.0000	c1 > c5 *
c2-c5	0.4333	5.9720	44	0.0000	0.0000	c2 > c5 *
c1-c4	0.3889	5.5939	44	0.0000	0.0000	c1 > c4 *
c2-c4	0.3056	5.3386	44	0.0000	0.0000	c2 > c4 *
c1-c3	0.2611	4.6968	44	0.0000	0.0002	c1 > c3 *
c3-c5	0.2556	3.7019	44	0.0006	0.0030	c3 > c5 *
c2-c3	0.1778	3.4272	44	0.0013	0.0053	c2 > c3 *
c1-c2	0.0833	2.6220	44	0.0120	0.0359	c1 > c2 *
c4-c5	0.1278	2.3809	44	0.0217	0.0433	c4 > c5 *
c3-c4	0.1278	2.0655	44	0.0448	0.0448	c3 > c4 *

< MULTIPLE COMPARISON for "C at b3" >

== Holm's Sequentially Rejective Bonferroni Procedure ==
== The factor < C at b3 > is analysed as dependent means. ==
== Alpha level is 0.05. ==

Pair	Diff	t-value	df	p	adj.p	
c3-c5	0.1556	3.6175	44	0.0008	0.0076	c3 > c5 *
c2-c5	0.1944	3.5364	44	0.0010	0.0087	c2 > c5 *
c1-c5	0.2167	3.4744	44	0.0012	0.0093	c1 > c5 *
c2-c4	0.1111	3.1623	44	0.0028	0.0198	c2 > c4 *
c1-c4	0.1333	3.0336	44	0.0040	0.0243	c1 > c4 *
c3-c4	0.0722	2.2295	44	0.0309	0.1547	c3 = c4
c4-c5	0.0833	1.9149	44	0.0620	0.2481	c4 = c5
c1-c3	0.0611	1.7132	44	0.0937	0.2812	c1 = c3
c2-c3	0.0389	1.2656	44	0.2123	0.4246	c2 = c3
c1-c2	0.0222	0.7031	44	0.4857	0.4857	c1 = c2

< MULTIPLE COMPARISON for "C at b4" >

== Holm's Sequentially Rejective Bonferroni Procedure ==
== The factor < C at b4 > is analysed as dependent means. ==
== Alpha level is 0.05. ==

Pair	Diff	t-value	df	p	adj.p	
c1-c5	0.1056	2.8014	44	0.0075	0.0753	c1 = c5
c2-c5	0.1056	2.7411	44	0.0088	0.0793	c2 = c5
c1-c4	0.1167	2.6988	44	0.0098	0.0793	c1 = c4
c2-c4	0.1167	2.6120	44	0.0123	0.0859	c2 = c4
c3-c5	0.0778	1.9264	44	0.0605	0.3632	c3 = c5
c3-c4	0.0889	1.8353	44	0.0732	0.3661	c3 = c4
c1-c3	0.0278	1.1512	44	0.2559	1.0000	c1 = c3
c2-c3	0.0278	1.0935	44	0.2801	1.0000	c2 = c3
c4-c5	-0.0111	0.1999	44	0.8425	1.0000	c4 = c5
c1-c2	0.0000	0.0000	44	1.0000	1.0000	c1 = c2

output is over --------------------///

[[Simple Effects for a2]]

[sAB-Type Design]

This output was generated by anovakun 4.8.5 under R version 4.0.2.
It was executed on Mon Oct 11 18:22:24 2021.

<< DESCRIPTIVE STATISTICS >>

B	C	n	Mean	S.D.
b1	c1	41	0.0244	0.3298
b1	c2	41	-0.4207	0.4791
b1	c3	41	-0.5305	0.6782
b1	c4	41	-0.7134	0.7171
b1	c5	41	-1.0610	0.8344
b2	c1	41	-0.0305	0.1695
b2	c2	41	-0.1280	0.2569
b2	c3	41	-0.2378	0.3160
b2	c4	41	-0.5305	0.6713
b2	c5	41	-0.6585	0.6040
b3	c1	41	0.0488	0.2033
b3	c2	41	-0.0061	0.1044
b3	c3	41	-0.0183	0.2704
b3	c4	41	-0.1890	0.3344
b3	c5	41	-0.2988	0.4944
b4	c1	41	0.0122	0.1364
b4	c2	41	-0.0183	0.1713
b4	c3	41	-0.0122	0.3352
b4	c4	41	-0.0244	0.2359
b4	c5	41	-0.1463	0.3831

<< SPHERICITY INDICES >>

== Mendoza's Multisample Sphericity Test and Epsilons ==

Effect	Lambda	approx.Chi	df	p		LB	GG	HF	CM
Global	0.0000	951.6359	189	0.0000	***	0.0526	0.1562	0.1701	0.1689
B	0.0000	93.3090	5	0.0000	***	0.3333	0.4330	0.4415	0.4382
C	0.0000	113.2983	9	0.0000	***	0.2500	0.3875	0.4002	0.3973
B x C	0.0000	229.1032	77	0.0000	***	0.0833	0.4780	0.5671	0.5629

LB = lower.bound, GG = Greenhouse-Geisser
HF = Huynh-Feldt-Lecoutre, CM = Chi-Muller

<< ANOVA TABLE >>

== Adjusted by Greenhouse-Geisser's Epsilon ==

Source	SS	df	MS	F-ratio	p-value		G.eta^2
s	55.2486	40	1.3812				
B	32.4881	1.3	25.0114	33.4020	0.0000	***	0.1737
s x B	38.9056	51.96	0.7488				
C	29.7271	1.55	19.1776	45.1088	0.0000	***	0.1613
s x C	26.3604	62	0.4251				
B x C	12.1308	5.74	2.1149	14.2687	0.0000	***	0.0728
s x B x C	34.0067	229.43	0.1482				
Total	228.8674	819	0.2794				

+p < .10, *p < .05, **p < .01, ***p < .001

<< POST ANALYSES >>

< MULTIPLE COMPARISON for "B" >

== Holm's Sequentially Rejective Bonferroni Procedure ==
== The factor < B > is analysed as dependent means. ==
== Alpha level is 0.05. ==

B	n	Mean	S.D.
b1	205	-0.5402	0.7220
b2	205	-0.3171	0.5056
b3	205	-0.0927	0.3339
b4	205	-0.0378	0.2725

Pair	Diff	t-value	df	p	adj.p	
b1-b4	-0.5024	6.2219	40	0.0000	0.0000	b1 < b4 *
b2-b4	-0.2793	6.0410	40	0.0000	0.0000	b2 < b4 *
b1-b3	-0.4476	5.8166	40	0.0000	0.0000	b1 < b3 *
b2-b3	-0.2244	5.5868	40	0.0000	0.0000	b2 < b3 *
b1-b2	-0.2232	4.7564	40	0.0000	0.0001	b1 < b2 *
b3-b4	-0.0549	2.2694	40	0.0287	0.0287	b3 < b4 *

< MULTIPLE COMPARISON for "C" >

== Holm's Sequentially Rejective Bonferroni Procedure ==

== The factor < C > is analysed as dependent means. ==
== Alpha level is 0.05. ==

C	n	Mean	S.D.
c1	164	0.0137	0.2220
c2	164	-0.1433	0.3324
c3	164	-0.1997	0.4774
c4	164	-0.3643	0.5935
c5	164	-0.5412	0.6941

Pair	Diff	t-value	df	p	adj.p	
c3-c5	0.3415	8.0565	40	0.0000	0.0000	c3 > c5 *
c1-c5	0.5549	7.6997	40	0.0000	0.0000	c1 > c5 *
c2-c5	0.3979	7.5904	40	0.0000	0.0000	c2 > c5 *
c1-c4	0.3780	6.2463	40	0.0000	0.0000	c1 > c4 *
c4-c5	0.1768	5.6189	40	0.0000	0.0000	c4 > c5 *
c1-c3	0.2134	5.4408	40	0.0000	0.0000	c1 > c3 *
c1-c2	0.1570	5.2449	40	0.0000	0.0000	c1 > c2 *
c2-c4	0.2210	5.2360	40	0.0000	0.0000	c2 > c4 *
c3-c4	0.1646	5.1234	40	0.0000	0.0000	c3 > c4 *
c2-c3	0.0564	2.6541	40	0.0114	0.0114	c2 > c3 *

< SIMPLE EFFECTS for "B x C" INTERACTION >

Effect	Lambda	approx.Chi	df	p		LB	GG	HF	CM
B at c1	0.0000	55.0422	5	0.0000	***	0.3333	0.5598	0.5816	0.5773
B at c2	0.0000	61.2685	5	0.0000	***	0.3333	0.5161	0.5329	0.5290
B at c3	0.0000	38.6279	5	0.0000	***	0.3333	0.5918	0.6173	0.6128
B at c4	0.0000	46.1119	5	0.0000	***	0.3333	0.6321	0.6627	0.6578
B at c5	0.0000	50.1290	5	0.0000	***	0.3333	0.5426	0.5624	0.5583
C at b1	0.0000	68.4244	9	0.0000	***	0.2500	0.5054	0.5324	0.5285
C at b2	0.0000	60.8486	9	0.0000	***	0.2500	0.5910	0.6306	0.6260
C at b3	0.0000	76.1514	9	0.0000	***	0.2500	0.4715	0.4940	0.4904
C at b4	0.0000	48.4921	9	0.0000	***	0.2500	0.6645	0.7162	

0.7110

LB = lower.bound, GG = Greenhouse-Geisser
HF = Huynh-Feldt-Lecoutre, CM = Chi-Muller

Source	SS	df	MS	F-ratio	p-value		G.eta^2
B at c1	0.1353	1.68	0.0806	1.2443	0.2900	ns	0.0168
s x B at c1	4.3491	67.18	0.0647				
B at c2	4.5777	1.55	2.9568	23.9641	0.0000	***	0.2542
s x B at c2	7.6410	61.93	0.1234				
B at c3	7.3365	1.78	4.1324	15.8699	0.0000	***	0.1975
s x B at c3	18.4916	71.01	0.2604				
B at c4	12.1261	1.9	6.3946	27.4006	0.0000	***	0.2112
s x B at c4	17.7020	75.85	0.2334				
B at c5	20.4432	1.63	12.5585	33.0681	0.0000	***	0.2603
s x B at c5	24.7287	65.11	0.3798				
C at b1	26.0079	2.02	12.8654	38.3639	0.0000	***	0.2445
s x C at b1	27.1171	80.86	0.3354				
C at b2	11.7378	2.36	4.9650	27.2383	0.0000	***	0.2251
s x C at b2	17.2372	94.56	0.1823				
C at b3	3.4768	1.89	1.8436	13.2790	0.0000	***	0.1529
s x C at b3	10.4732	75.43	0.1388				
C at b4	0.6354	2.66	0.2390	4.5878	0.0064	**	0.0420
s x C at b4	5.5396	106.32	0.0521				

+p < .10, *p < .05, **p < .01, ***p < .001

< MULTIPLE COMPARISON for "B at c2" >

== Holm's Sequentially Rejective Bonferroni Procedure ==
== The factor < B at c2 > is analysed as dependent means. ==
== Alpha level is 0.05. ==

Pair	Diff	t-value	df	p	adj.p	
b1-b3	-0.4146	5.6124	40	0.0000	0.0000	b1 < b3 *
b1-b4	-0.4024	5.2573	40	0.0000	0.0000	b1 < b4 *
b1-b2	-0.2927	4.8952	40	0.0000	0.0001	b1 < b2 *
b2-b3	-0.1220	3.3758	40	0.0016	0.0049	b2 < b3 *

```
 b2-b4  -0.1098   2.6186  40  0.0124  0.0248  b2 < b4 *
 b3-b4   0.0122   0.4669  40  0.6431  0.6431  b3 = b4
----------------------------------------------------------


< MULTIPLE COMPARISON for "B at c3" >

== Holm's Sequentially Rejective Bonferroni Procedure ==
== The factor < B at c3 > is analysed as dependent means. ==
== Alpha level is 0.05. ==

----------------------------------------------------------
  Pair     Diff  t-value  df       p   adj.p
----------------------------------------------------------
 b1-b4  -0.5183   4.5614  40  0.0000  0.0003  b1 < b4 *
 b1-b3  -0.5122   4.4611  40  0.0001  0.0003  b1 < b3 *
 b1-b2  -0.2927   3.5962  40  0.0009  0.0035  b1 < b2 *
 b2-b4  -0.2256   3.3416  40  0.0018  0.0054  b2 < b4 *
 b2-b3  -0.2195   3.2680  40  0.0022  0.0054  b2 < b3 *
 b3-b4  -0.0061   0.1059  40  0.9162  0.9162  b3 = b4
----------------------------------------------------------


< MULTIPLE COMPARISON for "B at c4" >

== Holm's Sequentially Rejective Bonferroni Procedure ==
== The factor < B at c4 > is analysed as dependent means. ==
== Alpha level is 0.05. ==

----------------------------------------------------------
  Pair     Diff  t-value  df       p   adj.p
----------------------------------------------------------
 b1-b4  -0.6890   6.4704  40  0.0000  0.0000  b1 < b4 *
 b2-b4  -0.5061   5.6711  40  0.0000  0.0000  b2 < b4 *
 b1-b3  -0.5244   5.2121  40  0.0000  0.0000  b1 < b3 *
 b2-b3  -0.3415   4.5236  40  0.0001  0.0002  b2 < b3 *
 b3-b4  -0.1646   4.0566  40  0.0002  0.0004  b3 < b4 *
 b1-b2  -0.1829   2.2858  40  0.0276  0.0276  b1 < b2 *
----------------------------------------------------------


< MULTIPLE COMPARISON for "B at c5" >

== Holm's Sequentially Rejective Bonferroni Procedure ==
== The factor < B at c5 > is analysed as dependent means. ==
== Alpha level is 0.05. ==

----------------------------------------------------------
  Pair     Diff  t-value  df       p   adj.p
----------------------------------------------------------
 b1-b4  -0.9146   6.6444  40  0.0000  0.0000  b1 < b4 *
 b1-b3  -0.7622   6.0396  40  0.0000  0.0000  b1 < b3 *
 b2-b4  -0.5122   5.4720  40  0.0000  0.0000  b2 < b4 *
 b1-b2  -0.4024   4.7831  40  0.0000  0.0001  b1 < b2 *
```

b2-b3	-0.3598	4.5218	40	0.0001	0.0001	b2 < b3 *
b3-b4	-0.1524	2.6665	40	0.0110	0.0110	b3 < b4 *

< MULTIPLE COMPARISON for "C at b1" >

== Holm's Sequentially Rejective Bonferroni Procedure ==
== The factor < C at b1 > is analysed as dependent means. ==
== Alpha level is 0.05. ==

Pair	Diff	t-value	df	p	adj.p	
c1-c5	1.0854	7.7912	40	0.0000	0.0000	c1 > c5 *
c2-c5	0.6402	6.8670	40	0.0000	0.0000	c2 > c5 *
c3-c5	0.5305	6.6618	40	0.0000	0.0000	c3 > c5 *
c1-c4	0.7378	6.2824	40	0.0000	0.0000	c1 > c4 *
c1-c2	0.4451	5.4502	40	0.0000	0.0000	c1 > c2 *
c4-c5	0.3476	5.0205	40	0.0000	0.0001	c4 > c5 *
c1-c3	0.5549	4.9704	40	0.0000	0.0001	c1 > c3 *
c2-c4	0.2927	4.5663	40	0.0000	0.0001	c2 > c4 *
c3-c4	0.1829	3.3979	40	0.0015	0.0031	c3 > c4 *
c2-c3	0.1098	1.8526	40	0.0713	0.0713	c2 = c3

< MULTIPLE COMPARISON for "C at b2" >

== Holm's Sequentially Rejective Bonferroni Procedure ==
== The factor < C at b2 > is analysed as dependent means. ==
== Alpha level is 0.05. ==

Pair	Diff	t-value	df	p	adj.p	
c1-c5	0.6280	7.0164	40	0.0000	0.0000	c1 > c5 *
c2-c5	0.5305	6.5449	40	0.0000	0.0000	c2 > c5 *
c3-c5	0.4207	5.8253	40	0.0000	0.0000	c3 > c5 *
c1-c4	0.5000	5.5627	40	0.0000	0.0000	c1 > c4 *
c2-c4	0.4024	4.7831	40	0.0000	0.0001	c2 > c4 *
c1-c3	0.2073	4.2717	40	0.0001	0.0006	c1 > c3 *
c3-c4	0.2927	3.5962	40	0.0009	0.0035	c3 > c4 *
c1-c2	0.0976	2.8044	40	0.0077	0.0232	c1 > c2 *
c2-c3	0.1098	2.5119	40	0.0161	0.0323	c2 > c3 *
c4-c5	0.1280	1.7271	40	0.0919	0.0919	c4 = c5

< MULTIPLE COMPARISON for "C at b3" >

== Holm's Sequentially Rejective Bonferroni Procedure ==
== The factor < C at b3 > is analysed as dependent means. ==
== Alpha level is 0.05. ==

Pair	Diff	t-value	df	p	adj.p	
c1-c4	0.2378	4.6074	40	0.0000	0.0004	c1 > c4 *
c1-c5	0.3476	4.5404	40	0.0001	0.0005	c1 > c5 *
c2-c5	0.2927	3.7983	40	0.0005	0.0039	c2 > c5 *
c2-c4	0.1829	3.5921	40	0.0009	0.0062	c2 > c4 *
c3-c5	0.2805	3.5437	40	0.0010	0.0062	c3 > c5 *
c3-c4	0.1707	3.4878	40	0.0012	0.0062	c3 > c4 *
c4-c5	0.1098	2.4632	40	0.0182	0.0727	c4 = c5
c1-c2	0.0549	1.5960	40	0.1184	0.3551	c1 = c2
c1-c3	0.0671	1.5674	40	0.1249	0.3551	c1 = c3
c2-c3	0.0122	0.3497	40	0.7284	0.7284	c2 = c3

< MULTIPLE COMPARISON for "C at b4" >

== Holm's Sequentially Rejective Bonferroni Procedure ==
== The factor < C at b4 > is analysed as dependent means. ==
== Alpha level is 0.05. ==

Pair	Diff	t-value	df	p	adj.p	
c1-c5	0.1585	2.9553	40	0.0052	0.0522	c1 = c5
c3-c5	0.1341	2.8008	40	0.0078	0.0703	c3 = c5
c2-c5	0.1280	2.6724	40	0.0108	0.0868	c2 = c5
c4-c5	0.1220	2.5451	40	0.0149	0.1042	c4 = c5
c1-c4	0.0366	1.2325	40	0.2250	1.0000	c1 = c4
c1-c2	0.0305	1.1516	40	0.2563	1.0000	c1 = c2
c1-c3	0.0244	0.6278	40	0.5337	1.0000	c1 = c3
c3-c4	0.0122	0.4221	40	0.6752	1.0000	c3 = c4
c2-c4	0.0061	0.1835	40	0.8553	1.0000	c2 = c4
c2-c3	-0.0061	0.1332	40	0.8947	1.0000	c2 = c3

output is over --------------------///

[[Simple Effects for a3]]

[sAB-Type Design]

This output was generated by anovakun 4.8.5 under R version 4.0.2.
It was executed on Mon Oct 11 18:22:25 2021.

<< DESCRIPTIVE STATISTICS >>

B	C	n	Mean	S.D.

```
 b1  c1  46  -0.0652  0.2496
 b1  c2  46  -0.4185  0.4946
 b1  c3  46  -0.7337  0.7273
 b1  c4  46  -0.9891  0.9617
 b1  c5  46  -1.5652  1.0832
 b2  c1  46   0.0000  0.0527
 b2  c2  46  -0.1576  0.3389
 b2  c3  46  -0.3152  0.4231
 b2  c4  46  -0.6250  0.5980
 b2  c5  46  -0.9402  0.6671
 b3  c1  46   0.0109  0.0906
 b3  c2  46  -0.0217  0.2347
 b3  c3  46  -0.2120  0.4624
 b3  c4  46  -0.2065  0.4160
 b3  c5  46  -0.4022  0.5413
 b4  c1  46   0.0109  0.1577
 b4  c2  46  -0.0109  0.1173
 b4  c3  46  -0.0652  0.2710
 b4  c4  46  -0.0978  0.3139
 b4  c5  46  -0.2880  0.5296
-------------------------------


<< SPHERICITY INDICES >>

== Mendoza's Multisample Sphericity Test and Epsilons ==

--------------------------------------------------------------------
-----
 Effect  Lambda  approx.Chi  df      p         LB     GG     HF
CM
--------------------------------------------------------------------
-----
 Global  0.0000    936.5255 189 0.0000 *** 0.0526 0.1887 0.2070
0.2058
      B  0.0000     87.3948   5 0.0000 *** 0.3333 0.4859 0.4981
0.4952
      C  0.0000     95.5785   9 0.0000 *** 0.2500 0.4912 0.5134
0.5105
  B x C  0.0000    262.6488  77 0.0000 *** 0.0833 0.4860 0.5666
0.5633
--------------------------------------------------------------------
-----
                              LB = lower.bound, GG = Greenhouse-
Geisser
                             HF = Huynh-Feldt-Lecoutre, CM = Chi-
Muller


<< ANOVA TABLE >>

== Adjusted by Greenhouse-Geisser's Epsilon ==

-------------------------------------------------------------------
```

Source	SS	df	MS	F-ratio	p-value		G.eta^2
s	87.2647	45	1.9392				
B	61.6312	1.46	42.2762	51.4098	0.0000	***	0.2063
s x B	53.9469	65.6	0.8223				
C	68.5774	1.96	34.9015	70.5206	0.0000	***	0.2244
s x C	43.7601	88.42	0.4949				
B x C	25.1171	5.83	4.3065	21.7066	0.0000	***	0.0958
s x B x C	52.0704	262.46	0.1984				
Total	392.3679	919	0.4270				

+p < .10, *p < .05, **p < .01, ***p < .001

<< POST ANALYSES >>

< MULTIPLE COMPARISON for "B" >

== Holm's Sequentially Rejective Bonferroni Procedure ==
== The factor < B > is analysed as dependent means. ==
== Alpha level is 0.05. ==

B	n	Mean	S.D.
b1	230	-0.7543	0.9153
b2	230	-0.4076	0.5746
b3	230	-0.1663	0.4104
b4	230	-0.0902	0.3284

Pair	Diff	t-value	df	p	adj.p	
b2-b3	-0.2413	8.7577	45	0.0000	0.0000	b2 < b3 *
b1-b4	-0.6641	7.8521	45	0.0000	0.0000	b1 < b4 *
b1-b3	-0.5880	7.7521	45	0.0000	0.0000	b1 < b3 *
b2-b4	-0.3174	7.3395	45	0.0000	0.0000	b2 < b4 *
b1-b2	-0.3467	5.2119	45	0.0000	0.0000	b1 < b2 *
b3-b4	-0.0761	2.5564	45	0.0140	0.0140	b3 < b4 *

< MULTIPLE COMPARISON for "C" >

== Holm's Sequentially Rejective Bonferroni Procedure ==
== The factor < C > is analysed as dependent means. ==
== Alpha level is 0.05. ==

C	n	Mean	S.D.

```
---------------------------
 c1 184  -0.0109  0.1586
 c2 184  -0.1522  0.3639
 c3 184  -0.3315  0.5539
 c4 184  -0.4796  0.7128
 c5 184  -0.7989  0.8926
---------------------------

---------------------------------------------------------
  Pair    Diff  t-value  df       p   adj.p
---------------------------------------------------------
 c2-c5  0.6467  11.2981  45  0.0000  0.0000  c2 > c5 *
 c1-c5  0.7880   9.9307  45  0.0000  0.0000  c1 > c5 *
 c3-c5  0.4674   9.3314  45  0.0000  0.0000  c3 > c5 *
 c2-c4  0.3274   7.6520  45  0.0000  0.0000  c2 > c4 *
 c1-c4  0.4688   7.1314  45  0.0000  0.0000  c1 > c4 *
 c4-c5  0.3193   6.7253  45  0.0000  0.0000  c4 > c5 *
 c1-c3  0.3207   6.1614  45  0.0000  0.0000  c1 > c3 *
 c2-c3  0.1793   5.3070  45  0.0000  0.0000  c2 > c3 *
 c3-c4  0.1481   4.8179  45  0.0000  0.0000  c3 > c4 *
 c1-c2  0.1413   4.1824  45  0.0001  0.0001  c1 > c2 *
---------------------------------------------------------


< SIMPLE EFFECTS for "B x C" INTERACTION >

--------------------------------------------------------------------
------
  Effect  Lambda  approx.Chi  df      p         LB     GG     HF
CM
--------------------------------------------------------------------
------
 B at c1  0.0000     70.4796   5 0.0000 *** 0.3333 0.5365 0.5534
0.5502
 B at c2  0.0000     36.2544   5 0.0000 *** 0.3333 0.6605 0.6908
0.6868
 B at c3  0.0001     17.5019   5 0.0036 **  0.3333 0.7718 0.8161
0.8114
 B at c4  0.0000     45.5669   5 0.0000 *** 0.3333 0.6062 0.6303
0.6266
 B at c5  0.0000     35.6414   5 0.0000 *** 0.3333 0.6672 0.6983
0.6943
 C at b1  0.0000     72.0489   9 0.0000 *** 0.2500 0.5400 0.5682
0.5649
 C at b2  0.0000     49.3586   9 0.0000 *** 0.2500 0.6155 0.6538
0.6500
 C at b3  0.0000     46.2855   9 0.0000 *** 0.2500 0.7470 0.8061
0.8014
 C at b4  0.0000     94.4459   9 0.0000 *** 0.2500 0.5347 0.5622
0.5589
--------------------------------------------------------------------
------
                               LB = lower.bound, GG = Greenhouse-
Geisser
```

HF = Huynh-Feldt-Lecoutre, CM = Chi-Muller

Source	SS	df	MS	F-ratio	p-value		G.eta^2
B at c1	0.1848	1.61	0.1148	2.6065	0.0918	+	0.0401
s x B at c1	3.1902	72.42	0.0440				
B at c2	4.9647	1.98	2.5054	24.3889	0.0000	***	0.2048
s x B at c2	9.1603	89.17	0.1027				
B at c3	11.3723	2.32	4.9118	26.4948	0.0000	***	0.2025
s x B at c3	19.3152	104.19	0.1854				
B at c4	23.0499	1.82	12.6753	34.4349	0.0000	***	0.2479
s x B at c4	30.1219	81.83	0.3681				
B at c5	47.1766	2	23.5682	47.9983	0.0000	***	0.3235
s x B at c5	44.2296	90.08	0.4910				
C at b1	59.8353	2.16	27.6992	61.2444	0.0000	***	0.3119
s x C at b1	43.9647	97.21	0.4523				
C at b2	26.1332	2.46	10.6151	57.0403	0.0000	***	0.3457
s x C at b2	20.6168	110.78	0.1861				
C at b3	5.1348	2.99	1.7184	13.8651	0.0000	***	0.1331
s x C at b3	16.6652	134.47	0.1239				
C at b4	2.5913	2.14	1.2116	7.9958	0.0005	***	0.1050
s x C at b4	14.5837	96.25	0.1515				

+p < .10, *p < .05, **p < .01, ***p < .001

< MULTIPLE COMPARISON for "B at c1" >

== Holm's Sequentially Rejective Bonferroni Procedure ==
== The factor < B at c1 > is analysed as dependent means. ==
== Alpha level is 0.05. ==

Pair	Diff	t-value	df	p	adj.p	
b1-b3	-0.0761	1.9251	45	0.0606	0.3633	b1 = b3
b1-b4	-0.0761	1.7078	45	0.0946	0.4728	b1 = b4
b1-b2	-0.0652	1.6978	45	0.0964	0.4728	b1 = b2
b2-b3	-0.0109	0.8135	45	0.4202	1.0000	b2 = b3
b2-b4	-0.0109	0.5303	45	0.5985	1.0000	b2 = b4
b3-b4	0.0000	0.0000	45	1.0000	1.0000	b3 = b4

< MULTIPLE COMPARISON for "B at c2" >

== Holm's Sequentially Rejective Bonferroni Procedure ==
== The factor < B at c2 > is analysed as dependent means. ==
== Alpha level is 0.05. ==

Pair	Diff	t-value	df	p	adj.p	
b1-b4	-0.4076	5.9637	45	0.0000	0.0000	b1 < b4 *
b1-b3	-0.3967	5.9120	45	0.0000	0.0000	b1 < b3 *
b1-b2	-0.2609	4.1977	45	0.0001	0.0005	b1 < b2 *
b2-b3	-0.1359	3.4485	45	0.0012	0.0037	b2 < b3 *
b2-b4	-0.1467	3.2114	45	0.0024	0.0049	b2 < b4 *
b3-b4	-0.0109	0.3397	45	0.7357	0.7357	b3 = b4

< MULTIPLE COMPARISON for "B at c3" >

== Holm's Sequentially Rejective Bonferroni Procedure ==
== The factor < B at c3 > is analysed as dependent means. ==
== Alpha level is 0.05. ==

Pair	Diff	t-value	df	p	adj.p	
b1-b4	-0.6685	6.6756	45	0.0000	0.0000	b1 < b4 *
b1-b3	-0.5217	6.0094	45	0.0000	0.0000	b1 < b3 *
b1-b2	-0.4185	4.7776	45	0.0000	0.0001	b1 < b2 *
b2-b4	-0.2500	3.9014	45	0.0003	0.0009	b2 < b4 *
b3-b4	-0.1467	2.2803	45	0.0274	0.0548	b3 = b4
b2-b3	-0.1033	1.6666	45	0.1026	0.1026	b2 = b3

< MULTIPLE COMPARISON for "B at c4" >

== Holm's Sequentially Rejective Bonferroni Procedure ==
== The factor < B at c4 > is analysed as dependent means. ==
== Alpha level is 0.05. ==

Pair	Diff	t-value	df	p	adj.p	
b2-b3	-0.4185	6.9491	45	0.0000	0.0000	b2 < b3 *
b1-b4	-0.8913	6.7745	45	0.0000	0.0000	b1 < b4 *
b2-b4	-0.5272	6.5821	45	0.0000	0.0000	b2 < b4 *
b1-b3	-0.7826	6.4393	45	0.0000	0.0000	b1 < b3 *
b1-b2	-0.3641	3.2671	45	0.0021	0.0042	b1 < b2 *
b3-b4	-0.1087	1.7961	45	0.0792	0.0792	b3 = b4

< MULTIPLE COMPARISON for "B at c5" >

== Holm's Sequentially Rejective Bonferroni Procedure ==
== The factor < B at c5 > is analysed as dependent means. ==
== Alpha level is 0.05. ==

Pair	Diff	t-value	df	p	adj.p	
b1-b3	-1.1630	8.4567	45	0.0000	0.0000	b1 < b3 *
b1-b4	-1.2772	8.0559	45	0.0000	0.0000	b1 < b4 *
b2-b3	-0.5380	7.1049	45	0.0000	0.0000	b2 < b3 *
b2-b4	-0.6522	6.1458	45	0.0000	0.0000	b2 < b4 *
b1-b2	-0.6250	4.6927	45	0.0000	0.0001	b1 < b2 *
b3-b4	-0.1141	1.3959	45	0.1696	0.1696	b3 = b4

< MULTIPLE COMPARISON for "C at b1" >

== Holm's Sequentially Rejective Bonferroni Procedure ==
== The factor < C at b1 > is analysed as dependent means. ==
== Alpha level is 0.05. ==

Pair	Diff	t-value	df	p	adj.p	
c1-c5	1.5000	9.7619	45	0.0000	0.0000	c1 > c5 *
c2-c5	1.1467	9.6556	45	0.0000	0.0000	c2 > c5 *
c3-c5	0.8315	9.0418	45	0.0000	0.0000	c3 > c5 *
c1-c4	0.9239	7.1713	45	0.0000	0.0000	c1 > c4 *
c1-c3	0.6685	6.9534	45	0.0000	0.0000	c1 > c3 *
c2-c4	0.5707	6.1482	45	0.0000	0.0000	c2 > c4 *
c4-c5	0.5761	5.7218	45	0.0000	0.0000	c4 > c5 *
c1-c2	0.3533	5.1284	45	0.0000	0.0000	c1 > c2 *
c2-c3	0.3152	4.4409	45	0.0001	0.0001	c2 > c3 *
c3-c4	0.2554	3.3996	45	0.0014	0.0014	c3 > c4 *

< MULTIPLE COMPARISON for "C at b2" >

== Holm's Sequentially Rejective Bonferroni Procedure ==
== The factor < C at b2 > is analysed as dependent means. ==
== Alpha level is 0.05. ==

Pair	Diff	t-value	df	p	adj.p	
c2-c5	0.7826	10.1419	45	0.0000	0.0000	c2 > c5 *
c1-c5	0.9402	9.5007	45	0.0000	0.0000	c1 > c5 *
c3-c5	0.6250	9.4951	45	0.0000	0.0000	c3 > c5 *
c2-c4	0.4674	7.1077	45	0.0000	0.0000	c2 > c4 *
c1-c4	0.6250	7.0882	45	0.0000	0.0000	c1 > c4 *

c1-c3	0.3152	5.0534	45	0.0000	0.0000	c1 > c3 *
c4-c5	0.3152	4.9035	45	0.0000	0.0001	c4 > c5 *
c3-c4	0.3098	4.7585	45	0.0000	0.0001	c3 > c4 *
c1-c2	0.1576	3.1537	45	0.0029	0.0057	c1 > c2 *
c2-c3	0.1576	2.9472	45	0.0051	0.0057	c2 > c3 *

< MULTIPLE COMPARISON for "C at b3" >

== Holm's Sequentially Rejective Bonferroni Procedure ==
== The factor < C at b3 > is analysed as dependent means. ==
== Alpha level is 0.05. ==

Pair	Diff	t-value	df	p	adj.p	
c2-c5	0.3804	5.6629	45	0.0000	0.0000	c2 > c5 *
c1-c5	0.4130	5.2318	45	0.0000	0.0000	c1 > c5 *
c2-c4	0.1848	3.9946	45	0.0002	0.0019	c2 > c4 *
c1-c4	0.2174	3.5641	45	0.0009	0.0061	c1 > c4 *
c1-c3	0.2228	3.2215	45	0.0024	0.0142	c1 > c3 *
c4-c5	0.1957	3.1012	45	0.0033	0.0166	c4 > c5 *
c2-c3	0.1902	2.9013	45	0.0057	0.0229	c2 > c3 *
c3-c5	0.1902	2.5358	45	0.0148	0.0443	c3 > c5 *
c1-c2	0.0326	0.9476	45	0.3484	0.6968	c1 = c2
c3-c4	-0.0054	0.0885	45	0.9299	0.9299	c3 = c4

< MULTIPLE COMPARISON for "C at b4" >

== Holm's Sequentially Rejective Bonferroni Procedure ==
== The factor < C at b4 > is analysed as dependent means. ==
== Alpha level is 0.05. ==

Pair	Diff	t-value	df	p	adj.p	
c1-c5	0.2989	3.8932	45	0.0003	0.0032	c1 > c5 *
c2-c5	0.2772	3.4445	45	0.0012	0.0112	c2 > c5 *
c3-c5	0.2228	3.4217	45	0.0013	0.0112	c3 > c5 *
c4-c5	0.1902	2.2328	45	0.0306	0.2141	c4 = c5
c2-c4	0.0870	2.1449	45	0.0374	0.2244	c2 = c4
c1-c4	0.1087	2.0479	45	0.0464	0.2322	c1 = c4
c1-c3	0.0761	1.6125	45	0.1138	0.4554	c1 = c3
c2-c3	0.0543	1.5673	45	0.1241	0.4554	c2 = c3
c1-c2	0.0217	0.7265	45	0.4713	0.9425	c1 = c2
c3-c4	0.0326	0.6429	45	0.5236	0.9425	c3 = c4

output is over --------------------///

[[Simple Effects for a4]]

[sAB-Type Design]

This output was generated by anovakun 4.8.5 under R version 4.0.2.
It was executed on Mon Oct 11 18:22:26 2021.

<< DESCRIPTIVE STATISTICS >>

B	C	n	Mean	S.D.
b1	c1	44	0.0227	0.3316
b1	c2	44	-0.2784	0.6057
b1	c3	44	-0.6080	0.8130
b1	c4	44	-0.7955	0.9265
b1	c5	44	-1.0341	0.9684
b2	c1	44	0.0455	0.3015
b2	c2	44	-0.2045	0.3707
b2	c3	44	-0.3409	0.4944
b2	c4	44	-0.4602	0.6122
b2	c5	44	-0.8068	0.8758
b3	c1	44	0.0114	0.1422
b3	c2	44	-0.0398	0.2219
b3	c3	44	-0.1875	0.3150
b3	c4	44	-0.3182	0.4649
b3	c5	44	-0.5398	0.6170
b4	c1	44	0.0057	0.1373
b4	c2	44	0.0227	0.1301
b4	c3	44	-0.0568	0.1693
b4	c4	44	-0.1193	0.3120
b4	c5	44	-0.2955	0.5037

<< SPHERICITY INDICES >>

== Mendoza's Multisample Sphericity Test and Epsilons ==

Effect	Lambda	approx.Chi	df	p		LB	GG	HF	CM
Global	0.0000	911.5294	189	0.0000	***	0.0526	0.2248	0.2527	0.2511
B	0.0000	80.7478	5	0.0000	***	0.3333	0.4958	0.5094	0.5062
C	0.0000	117.6883	9	0.0000	***	0.2500	0.3911	0.4033	0.4007
B x C	0.0000	303.2337	77	0.0000	***	0.0833	0.4306	0.4964	0.4932

LB = lower.bound, GG = Greenhouse-Geisser
HF = Huynh-Feldt-Lecoutre, CM = Chi-Muller

<< ANOVA TABLE >>

== Adjusted by Greenhouse-Geisser's Epsilon ==

Source	SS	df	MS	F-ratio	p-value		G.eta^2
s	102.6864	43	2.3881				
B	24.5813	1.49	16.5278	20.5316	0.0000	***	0.0907
s x B	51.4813	63.95	0.8050				
C	50.2036	1.56	32.0878	52.7214	0.0000	***	0.1693
s x C	40.9464	67.28	0.6086				
B x C	9.7391	5.17	1.8848	8.1696	0.0000	***	0.0380
s x B x C	51.2609	222.19	0.2307				
Total	330.8989	879	0.3764				

+p < .10, *p < .05, **p < .01, ***p < .001

<< POST ANALYSES >>

< MULTIPLE COMPARISON for "B" >

== Holm's Sequentially Rejective Bonferroni Procedure ==
== The factor < B > is analysed as dependent means. ==
== Alpha level is 0.05. ==

B	n	Mean	S.D.
b1	220	-0.5386	0.8465
b2	220	-0.3534	0.6301
b3	220	-0.2148	0.4362
b4	220	-0.0886	0.3080

Pair	Diff	t-value	df	p	adj.p	
b2-b4	-0.2648	5.7243	43	0.0000	0.0000	b2 < b4 *
b3-b4	-0.1261	5.4663	43	0.0000	0.0000	b3 < b4 *
b1-b4	-0.4500	5.4508	43	0.0000	0.0000	b1 < b4 *
b1-b3	-0.3239	3.9912	43	0.0003	0.0008	b1 < b3 *

Pair	Diff	t-value	df	p	adj.p	
b2-b3	-0.1386	3.3954	43	0.0015	0.0030	b2 < b3 *
b1-b2	-0.1852	2.9181	43	0.0056	0.0056	b1 < b2 *

< MULTIPLE COMPARISON for "C" >

== Holm's Sequentially Rejective Bonferroni Procedure ==
== The factor < C > is analysed as dependent means. ==
== Alpha level is 0.05. ==

C	n	Mean	S.D.
c1	176	0.0213	0.2433
c2	176	-0.1250	0.3937
c3	176	-0.2983	0.5443
c4	176	-0.4233	0.6642
c5	176	-0.6690	0.8077

Pair	Diff	t-value	df	p	adj.p	
c2-c5	0.5440	8.6973	43	0.0000	0.0000	c2 > c5 *
c1-c5	0.6903	8.0231	43	0.0000	0.0000	c1 > c5 *
c3-c5	0.3707	7.1157	43	0.0000	0.0000	c3 > c5 *
c2-c4	0.2983	6.8981	43	0.0000	0.0000	c2 > c4 *
c1-c3	0.3196	6.8176	43	0.0000	0.0000	c1 > c3 *
c1-c4	0.4446	6.7741	43	0.0000	0.0000	c1 > c4 *
c2-c3	0.1733	6.6218	43	0.0000	0.0000	c2 > c3 *
c4-c5	0.2457	6.1441	43	0.0000	0.0000	c4 > c5 *
c1-c2	0.1463	4.2728	43	0.0001	0.0002	c1 > c2 *
c3-c4	0.1250	3.6248	43	0.0008	0.0008	c3 > c4 *

< SIMPLE EFFECTS for "B x C" INTERACTION >

Effect	Lambda	approx.Chi	df	p		LB	GG	HF	CM
B at c1	0.0000	52.3540	5	0.0000	***	0.3333	0.7092	0.7472	0.7424
B at c2	0.0000	53.6609	5	0.0000	***	0.3333	0.5793	0.6015	0.5977
B at c3	0.0000	69.7086	5	0.0000	***	0.3333	0.5110	0.5261	0.5228
B at c4	0.0000	44.0283	5	0.0000	***	0.3333	0.5955	0.6196	0.6156
B at c5	0.0000	39.1771	5	0.0000	***	0.3333	0.6820	0.7164	

```
0.7119
 C at b1  0.0000     50.5430   9 0.0000 *** 0.2500 0.6845 0.7357
0.7310
 C at b2  0.0000     72.3410   9 0.0000 *** 0.2500 0.5182 0.5449
0.5415
 C at b3  0.0000    103.1570   9 0.0000 *** 0.2500 0.4609 0.4806
0.4776
 C at b4  0.0000     70.6986   9 0.0000 *** 0.2500 0.5529 0.5841
0.5804
--------------------------------------------------------------------
------
                               LB = lower.bound, GG = Greenhouse-
Geisser
                              HF = Huynh-Feldt-Lecoutre, CM = Chi-
Muller

--------------------------------------------------------------------
     Source      SS     df       MS  F-ratio  p-value      G.eta^2
--------------------------------------------------------------------
    B at c1  0.0408   2.13  0.0192   0.3245   0.7370 ns    0.0039
s x B at c1  5.4123  91.49  0.0592
--------------------------------------------------------------------
    B at c2  2.5938   1.74  1.4926   9.6721   0.0004 ***   0.0956
s x B at c2 11.5312  74.73  0.1543
--------------------------------------------------------------------
    B at c3  7.4048   1.53  4.8304  14.3904   0.0000 ***   0.1428
s x B at c3 22.1264  65.92  0.3357
--------------------------------------------------------------------
    B at c4 10.7060   1.79  5.9929  18.2453   0.0000 ***   0.1387
s x B at c4 25.2315  76.82  0.3285
--------------------------------------------------------------------
    B at c5 13.5749   2.05  6.6351  15.1850   0.0000 ***   0.1189
s x B at c5 38.4407  87.98  0.4369
--------------------------------------------------------------------
    C at b1 30.7597   2.74 11.2340  37.1375   0.0000 ***   0.1960
s x C at b1 35.6153 117.74  0.3025
--------------------------------------------------------------------
    C at b2 17.5295   2.07  8.4564  25.1295   0.0000 ***   0.2016
s x C at b2 29.9955  89.14  0.3365
--------------------------------------------------------------------
    C at b3  8.7483   1.84  4.7448  24.3848   0.0000 ***   0.2100
s x C at b3 15.4267  79.28  0.1946
--------------------------------------------------------------------
    C at b4  2.9051   2.21  1.3137  11.1836   0.0000 ***   0.1399
s x C at b4 11.1699  95.09  0.1175
--------------------------------------------------------------------
                        +p < .10, *p < .05, **p < .01, ***p < .001


< MULTIPLE COMPARISON for "B at c2" >

== Holm's Sequentially Rejective Bonferroni Procedure ==
== The factor < B at c2 > is analysed as dependent means. ==
== Alpha level is 0.05. ==
```

Pair	Diff	t-value	df	p	adj.p	
b2-b4	-0.2273	4.4878	43	0.0001	0.0003	b2 < b4 *
b2-b3	-0.1648	3.7046	43	0.0006	0.0030	b2 < b3 *
b1-b4	-0.3011	3.6580	43	0.0007	0.0030	b1 < b4 *
b1-b3	-0.2386	2.8795	43	0.0062	0.0186	b1 < b3 *
b3-b4	-0.0625	2.0459	43	0.0469	0.0938	b3 = b4
b1-b2	-0.0739	1.0187	43	0.3141	0.3141	b1 = b2

< MULTIPLE COMPARISON for "B at c3" >

== Holm's Sequentially Rejective Bonferroni Procedure ==
== The factor < B at c3 > is analysed as dependent means. ==
== Alpha level is 0.05. ==

Pair	Diff	t-value	df	p	adj.p	
b1-b4	-0.5511	4.4751	43	0.0001	0.0003	b1 < b4 *
b2-b4	-0.2841	4.0200	43	0.0002	0.0012	b2 < b4 *
b1-b3	-0.4205	3.6507	43	0.0007	0.0028	b1 < b3 *
b3-b4	-0.1307	3.3174	43	0.0019	0.0056	b3 < b4 *
b1-b2	-0.2670	2.9457	43	0.0052	0.0104	b1 < b2 *
b2-b3	-0.1534	2.5633	43	0.0140	0.0140	b2 < b3 *

< MULTIPLE COMPARISON for "B at c4" >

== Holm's Sequentially Rejective Bonferroni Procedure ==
== The factor < B at c4 > is analysed as dependent means. ==
== Alpha level is 0.05. ==

Pair	Diff	t-value	df	p	adj.p	
b2-b4	-0.3409	5.6001	43	0.0000	0.0000	b2 < b4 *
b1-b4	-0.6761	5.4082	43	0.0000	0.0000	b1 < b4 *
b1-b3	-0.4773	4.2069	43	0.0001	0.0005	b1 < b3 *
b3-b4	-0.1989	3.5469	43	0.0010	0.0029	b3 < b4 *
b1-b2	-0.3352	2.9240	43	0.0055	0.0110	b1 < b2 *
b2-b3	-0.1420	2.0398	43	0.0475	0.0475	b2 < b3 *

< MULTIPLE COMPARISON for "B at c5" >

== Holm's Sequentially Rejective Bonferroni Procedure ==
== The factor < B at c5 > is analysed as dependent means. ==
== Alpha level is 0.05. ==

Pair	Diff	t-value	df	p	adj.p	
b1-b4	-0.7386	5.5411	43	0.0000	0.0000	b1 < b4 *
b2-b4	-0.5114	5.0540	43	0.0000	0.0000	b2 < b4 *
b3-b4	-0.2443	4.1099	43	0.0002	0.0007	b3 < b4 *
b1-b3	-0.4943	3.3709	43	0.0016	0.0048	b1 < b3 *
b2-b3	-0.2670	2.7247	43	0.0093	0.0185	b2 < b3 *
b1-b2	-0.2273	1.6656	43	0.1031	0.1031	b1 = b2

< MULTIPLE COMPARISON for "C at b1" >

== Holm's Sequentially Rejective Bonferroni Procedure ==
== The factor < C at b1 > is analysed as dependent means. ==
== Alpha level is 0.05. ==

Pair	Diff	t-value	df	p	adj.p	
c1-c5	1.0568	8.6322	43	0.0000	0.0000	c1 > c5 *
c2-c5	0.7557	7.8438	43	0.0000	0.0000	c2 > c5 *
c1-c4	0.8182	6.7655	43	0.0000	0.0000	c1 > c4 *
c1-c3	0.6307	6.5463	43	0.0000	0.0000	c1 > c3 *
c3-c5	0.4261	6.3308	43	0.0000	0.0000	c3 > c5 *
c2-c4	0.5170	5.7033	43	0.0000	0.0000	c2 > c4 *
c1-c2	0.3011	4.6463	43	0.0000	0.0001	c1 > c2 *
c2-c3	0.3295	4.4297	43	0.0001	0.0002	c2 > c3 *
c4-c5	0.2386	2.0659	43	0.0449	0.0898	c4 = c5
c3-c4	0.1875	1.8642	43	0.0691	0.0898	c3 = c4

< MULTIPLE COMPARISON for "C at b2" >

== Holm's Sequentially Rejective Bonferroni Procedure ==
== The factor < C at b2 > is analysed as dependent means. ==
== Alpha level is 0.05. ==

Pair	Diff	t-value	df	p	adj.p	
c1-c5	0.8523	6.0405	43	0.0000	0.0000	c1 > c5 *
c2-c5	0.6023	5.7283	43	0.0000	0.0000	c2 > c5 *
c1-c4	0.5057	5.5099	43	0.0000	0.0000	c1 > c4 *
c1-c3	0.3864	4.7664	43	0.0000	0.0002	c1 > c3 *
c2-c4	0.2557	4.5647	43	0.0000	0.0002	c2 > c4 *
c3-c5	0.4659	4.4815	43	0.0001	0.0003	c3 > c5 *
c1-c2	0.2500	3.6762	43	0.0007	0.0026	c1 > c2 *
c4-c5	0.3466	3.6139	43	0.0008	0.0026	c4 > c5 *
c2-c3	0.1364	2.9913	43	0.0046	0.0092	c2 > c3 *
c3-c4	0.1193	2.0262	43	0.0490	0.0490	c3 > c4 *

< MULTIPLE COMPARISON for "C at b3" >

== Holm's Sequentially Rejective Bonferroni Procedure ==
== The factor < C at b3 > is analysed as dependent means. ==
== Alpha level is 0.05. ==

Pair	Diff	t-value	df	p	adj.p	
c2-c5	0.5000	5.8920	43	0.0000	0.0000	c2 > c5 *
c1-c5	0.5511	5.6785	43	0.0000	0.0000	c1 > c5 *
c3-c5	0.3523	5.2434	43	0.0000	0.0000	c3 > c5 *
c1-c4	0.3295	4.8889	43	0.0000	0.0001	c1 > c4 *
c2-c4	0.2784	4.6518	43	0.0000	0.0002	c2 > c4 *
c2-c3	0.1477	4.2464	43	0.0001	0.0006	c2 > c3 *
c1-c3	0.1989	3.8889	43	0.0003	0.0014	c1 > c3 *
c4-c5	0.2216	3.8094	43	0.0004	0.0014	c4 > c5 *
c3-c4	0.1307	2.2854	43	0.0273	0.0546	c3 = c4
c1-c2	0.0511	1.5939	43	0.1183	0.1183	c1 = c2

< MULTIPLE COMPARISON for "C at b4" >

== Holm's Sequentially Rejective Bonferroni Procedure ==
== The factor < C at b4 > is analysed as dependent means. ==
== Alpha level is 0.05. ==

Pair	Diff	t-value	df	p	adj.p	
c2-c5	0.3182	4.3941	43	0.0001	0.0007	c2 > c5 *
c1-c5	0.3011	4.1794	43	0.0001	0.0013	c1 > c5 *
c3-c5	0.2386	3.3252	43	0.0018	0.0145	c3 > c5 *
c2-c4	0.1420	3.1188	43	0.0032	0.0227	c2 > c4 *
c4-c5	0.1761	2.6360	43	0.0116	0.0697	c4 = c5
c1-c4	0.1250	2.5811	43	0.0133	0.0697	c1 = c4
c2-c3	0.0795	2.5462	43	0.0146	0.0697	c2 = c3
c1-c3	0.0625	1.9149	43	0.0622	0.1865	c1 = c3
c3-c4	0.0625	1.2793	43	0.2076	0.4153	c3 = c4
c1-c2	-0.0170	0.6841	43	0.4976	0.4976	c1 = c2

output is over --------------------///

[[Simple Effects for a5]]

[sAB-Type Design]

This output was generated by anovakun 4.8.5 under R version 4.0.2.

It was executed on Mon Oct 11 18:22:27 2021.

<< DESCRIPTIVE STATISTICS >>

B	C	n	Mean	S.D.
b1	c1	44	-0.1875	0.3855
b1	c2	44	-0.7216	0.6630
b1	c3	44	-1.0398	0.9384
b1	c4	44	-1.3409	1.1011
b1	c5	44	-1.8750	1.2912
b2	c1	44	-0.1136	0.2825
b2	c2	44	-0.3920	0.5665
b2	c3	44	-0.6932	0.7089
b2	c4	44	-1.0341	0.8134
b2	c5	44	-1.3352	1.0633
b3	c1	44	-0.0568	0.1857
b3	c2	44	-0.1307	0.3906
b3	c3	44	-0.3920	0.5741
b3	c4	44	-0.6364	0.7576
b3	c5	44	-0.8636	0.8499
b4	c1	44	-0.0398	0.2347
b4	c2	44	-0.0795	0.3270
b4	c3	44	-0.2898	0.5730
b4	c4	44	-0.3693	0.5943
b4	c5	44	-0.5795	0.7601

<< SPHERICITY INDICES >>

== Mendoza's Multisample Sphericity Test and Epsilons ==

Effect	Lambda	approx.Chi	df	p		LB	GG	HF	CM
Global	0.0000	918.4691	189	0.0000	***	0.0526	0.1446	0.1554	0.1544
B	0.0000	129.9266	5	0.0000	***	0.3333	0.4073	0.4130	0.4104
C	0.0000	172.8961	9	0.0000	***	0.2500	0.3293	0.3356	0.3335
B x C	0.0000	208.5531	77	0.0000	***	0.0833	0.4998	0.5899	0.5861

LB = lower.bound, GG = Greenhouse-Geisser

HF = Huynh-Feldt-Lecoutre, CM = Chi-

Muller

<< ANOVA TABLE >>

== Adjusted by Greenhouse-Geisser's Epsilon ==

Source	SS	df	MS	F-ratio	p-value		G.eta^2
s	283.6611	43	6.5968				
B	75.1991	1.22	61.5462	64.2845	0.0000	***	0.1454
s x B	50.3009	52.54	0.9574				
C	123.2128	1.32	93.5430	77.7927	0.0000	***	0.2180
s x C	68.1060	56.64	1.2025				
B x C	18.6361	6	3.1070	20.0864	0.0000	***	0.0405
s x B x C	39.8952	257.92	0.1547				
Total	659.0111	879	0.7497				

+p < .10, *p < .05, **p < .01, ***p < .001

<< POST ANALYSES >>

< MULTIPLE COMPARISON for "B" >

== Holm's Sequentially Rejective Bonferroni Procedure ==
== The factor < B > is analysed as dependent means. ==
== Alpha level is 0.05. ==

B	n	Mean	S.D.
b1	220	-1.0330	1.0855
b2	220	-0.7136	0.8489
b3	220	-0.4159	0.6696
b4	220	-0.2716	0.5642

Pair	Diff	t-value	df	p	adj.p	
b1-b4	-0.7614	8.4106	43	0.0000	0.0000	b1 < b4 *
b1-b3	-0.6170	8.2361	43	0.0000	0.0000	b1 < b3 *
b2-b4	-0.4420	7.9161	43	0.0000	0.0000	b2 < b4 *
b2-b3	-0.2977	7.5275	43	0.0000	0.0000	b2 < b3 *
b1-b2	-0.3193	6.7846	43	0.0000	0.0000	b1 < b2 *
b3-b4	-0.1443	6.0798	43	0.0000	0.0000	b3 < b4 *

< MULTIPLE COMPARISON for "C" >

== Holm's Sequentially Rejective Bonferroni Procedure ==
== The factor < C > is analysed as dependent means. ==
== Alpha level is 0.05. ==

C	n	Mean	S.D.
c1	176	-0.0994	0.2855
c2	176	-0.3310	0.5621
c3	176	-0.6037	0.7664
c4	176	-0.8452	0.9094
c5	176	-1.1634	1.1180

Pair	Diff	t-value	df	p	adj.p	
c3-c5	0.5597	10.7618	43	0.0000	0.0000	c3 > c5 *
c2-c5	0.8324	10.0456	43	0.0000	0.0000	c2 > c5 *
c1-c5	1.0639	9.4595	43	0.0000	0.0000	c1 > c5 *
c2-c4	0.5142	8.5067	43	0.0000	0.0000	c2 > c4 *
c1-c4	0.7457	8.4317	43	0.0000	0.0000	c1 > c4 *
c4-c5	0.3182	7.9614	43	0.0000	0.0000	c4 > c5 *
c1-c3	0.5043	7.0234	43	0.0000	0.0000	c1 > c3 *
c3-c4	0.2415	6.7106	43	0.0000	0.0000	c3 > c4 *
c2-c3	0.2727	6.6519	43	0.0000	0.0000	c2 > c3 *
c1-c2	0.2315	5.8406	43	0.0000	0.0000	c1 > c2 *

< SIMPLE EFFECTS for "B x C" INTERACTION >

Effect	Lambda	approx.Chi	df	p		LB	GG	HF	CM
B at c1	0.0000	35.5918	5	0.0000	***	0.3333	0.6323	0.6606	0.6564
B at c2	0.0000	42.7628	5	0.0000	***	0.3333	0.6551	0.6862	0.6819
B at c3	0.0000	52.3698	5	0.0000	***	0.3333	0.5758	0.5977	0.5940
B at c4	0.0000	50.4845	5	0.0000	***	0.3333	0.5563	0.5760	0.5724
B at c5	0.0000	62.2948	5	0.0000	***	0.3333	0.5267	0.5434	0.5400
C at b1	0.0000	81.3738	9	0.0000	***	0.2500	0.4677	0.4882	0.4851
C at b2	0.0000	82.2970	9	0.0000	***	0.2500	0.4524	0.4711	0.4681

```
 C at b3  0.0000    101.6198   9 0.0000 *** 0.2500 0.4273 0.4432
0.4404
 C at b4  0.0000     71.8027   9 0.0000 *** 0.2500 0.5170 0.5436
0.5401
--------------------------------------------------------------------
------
                              LB = lower.bound, GG = Greenhouse-
Geisser
                             HF = Huynh-Feldt-Lecoutre, CM = Chi-
Muller

-------------------------------------------------------------------
     Source      SS    df       MS  F-ratio  p-value      G.eta^2
-------------------------------------------------------------------
    B at c1  0.5866   1.9  0.3093   6.1048   0.0039 **    0.0411
s x B at c1  4.1321 81.56  0.0507
-------------------------------------------------------------------
    B at c2 11.4244  1.97  5.8128  40.4194   0.0000 ***   0.2066
s x B at c2 12.1538 84.51  0.1438
-------------------------------------------------------------------
    B at c3 15.0266  1.73  8.6984  39.3356   0.0000 ***   0.1462
s x B at c3 16.4265 74.28  0.2211
-------------------------------------------------------------------
    B at c4 24.2653  1.67 14.5408  44.5180   0.0000 ***   0.1677
s x B at c4 23.4379 71.76  0.3266
-------------------------------------------------------------------
    B at c5 42.5323  1.58 26.9168  53.7185   0.0000 ***   0.1944
s x B at c5 34.0458 67.95  0.5011
-------------------------------------------------------------------
    C at b1 71.0892  1.87 37.9998  74.4013   0.0000 ***   0.2755
s x C at b1 41.0858 80.44  0.5107
-------------------------------------------------------------------
    C at b2 41.9278  1.81 23.1695  61.1210   0.0000 ***   0.2656
s x C at b2 29.4972 77.81  0.3791
-------------------------------------------------------------------
    C at b3 20.2369  1.71 11.8396  40.7333   0.0000 ***   0.2061
s x C at b3 21.3631  73.5  0.2907
-------------------------------------------------------------------
    C at b4  8.5949  2.07  4.1560  23.0195   0.0000 ***   0.1233
s x C at b4 16.0551 88.93  0.1805
-------------------------------------------------------------------
                       +p < .10, *p < .05, **p < .01, ***p < .001


< MULTIPLE COMPARISON for "B at c1" >

== Holm's Sequentially Rejective Bonferroni Procedure ==
== The factor < B at c1 > is analysed as dependent means. ==
== Alpha level is 0.05. ==

----------------------------------------------------------
  Pair     Diff  t-value  df       p   adj.p
----------------------------------------------------------
 b1-b4  -0.1477   2.8985  43  0.0059  0.0353  b1 < b4 *
```

```
 b1-b3  -0.1307   2.8207  43  0.0072  0.0361  b1 < b3 *
 b2-b4  -0.0739   2.6705  43  0.0106  0.0426  b2 < b4 *
 b2-b3  -0.0568   1.9490  43  0.0578  0.1735  b2 = b3
 b1-b2  -0.0739   1.7626  43  0.0851  0.1735  b1 = b2
 b3-b4  -0.0170   0.6841  43  0.4976  0.4976  b3 = b4
---------------------------------------------------------


< MULTIPLE COMPARISON for "B at c2" >

== Holm's Sequentially Rejective Bonferroni Procedure ==
== The factor < B at c2 > is analysed as dependent means. ==
== Alpha level is 0.05. ==

---------------------------------------------------------
  Pair     Diff  t-value  df       p   adj.p
---------------------------------------------------------
 b1-b4  -0.6420   7.6221  43  0.0000  0.0000  b1 < b4 *
 b1-b3  -0.5909   7.6171  43  0.0000  0.0000  b1 < b3 *
 b2-b3  -0.2614   5.0246  43  0.0000  0.0000  b2 < b3 *
 b2-b4  -0.3125   4.9175  43  0.0000  0.0000  b2 < b4 *
 b1-b2  -0.3295   4.8193  43  0.0000  0.0000  b1 < b2 *
 b3-b4  -0.0511   1.5005  43  0.1408  0.1408  b3 = b4
---------------------------------------------------------


< MULTIPLE COMPARISON for "B at c3" >

== Holm's Sequentially Rejective Bonferroni Procedure ==
== The factor < B at c3 > is analysed as dependent means. ==
== Alpha level is 0.05. ==

---------------------------------------------------------
  Pair     Diff  t-value  df       p   adj.p
---------------------------------------------------------
 b1-b4  -0.7500   7.3641  43  0.0000  0.0000  b1 < b4 *
 b1-b3  -0.6477   6.6106  43  0.0000  0.0000  b1 < b3 *
 b2-b4  -0.4034   6.1457  43  0.0000  0.0000  b2 < b4 *
 b2-b3  -0.3011   5.9647  43  0.0000  0.0000  b2 < b3 *
 b1-b2  -0.3466   4.4647  43  0.0001  0.0001  b1 < b2 *
 b3-b4  -0.1023   2.3645  43  0.0226  0.0226  b3 < b4 *
---------------------------------------------------------


< MULTIPLE COMPARISON for "B at c4" >

== Holm's Sequentially Rejective Bonferroni Procedure ==
== The factor < B at c4 > is analysed as dependent means. ==
== Alpha level is 0.05. ==

---------------------------------------------------------
  Pair     Diff  t-value  df       p   adj.p
---------------------------------------------------------
 b2-b4  -0.6648   7.8357  43  0.0000  0.0000  b2 < b4 *
```

```
 b1-b4  -0.9716   7.5718  43  0.0000  0.0000  b1 < b4 *
 b1-b3  -0.7045   6.4919  43  0.0000  0.0000  b1 < b3 *
 b2-b3  -0.3977   5.8352  43  0.0000  0.0000  b2 < b3 *
 b3-b4  -0.2670   4.8761  43  0.0000  0.0000  b3 < b4 *
 b1-b2  -0.3068   3.8157  43  0.0004  0.0004  b1 < b2 *
----------------------------------------------------------


< MULTIPLE COMPARISON for "B at c5" >

== Holm's Sequentially Rejective Bonferroni Procedure ==
== The factor < B at c5 > is analysed as dependent means. ==
== Alpha level is 0.05. ==

----------------------------------------------------------
  Pair     Diff  t-value  df       p   adj.p
----------------------------------------------------------
 b1-b4  -1.2955   8.3736  43  0.0000  0.0000  b1 < b4 *
 b1-b3  -1.0114   8.1875  43  0.0000  0.0000  b1 < b3 *
 b2-b4  -0.7557   6.4081  43  0.0000  0.0000  b2 < b4 *
 b1-b2  -0.5398   6.2765  43  0.0000  0.0000  b1 < b2 *
 b2-b3  -0.4716   5.3819  43  0.0000  0.0000  b2 < b3 *
 b3-b4  -0.2841   4.6056  43  0.0000  0.0000  b3 < b4 *
----------------------------------------------------------


< MULTIPLE COMPARISON for "C at b1" >

== Holm's Sequentially Rejective Bonferroni Procedure ==
== The factor < C at b1 > is analysed as dependent means. ==
== Alpha level is 0.05. ==

---------------------------------------------------------
  Pair    Diff  t-value  df       p   adj.p
---------------------------------------------------------
 c1-c5  1.6875  10.6484  43  0.0000  0.0000  c1 > c5 *
 c3-c5  0.8352   9.9365  43  0.0000  0.0000  c3 > c5 *
 c2-c5  1.1534   9.0069  43  0.0000  0.0000  c2 > c5 *
 c1-c4  1.1534   8.9175  43  0.0000  0.0000  c1 > c4 *
 c1-c3  0.8523   8.3598  43  0.0000  0.0000  c1 > c3 *
 c1-c2  0.5341   7.1869  43  0.0000  0.0000  c1 > c2 *
 c4-c5  0.5341   6.7921  43  0.0000  0.0000  c4 > c5 *
 c2-c4  0.6193   6.1302  43  0.0000  0.0000  c2 > c4 *
 c2-c3  0.3182   4.4220  43  0.0001  0.0001  c2 > c3 *
 c3-c4  0.3011   3.9588  43  0.0003  0.0003  c3 > c4 *
---------------------------------------------------------


< MULTIPLE COMPARISON for "C at b2" >

== Holm's Sequentially Rejective Bonferroni Procedure ==
== The factor < C at b2 > is analysed as dependent means. ==
== Alpha level is 0.05. ==
```

Pair	Diff	t-value	df	p	adj.p	
c2-c4	0.6420	8.9786	43	0.0000	0.0000	c2 > c4 *
c1-c4	0.9205	8.9310	43	0.0000	0.0000	c1 > c4 *
c2-c5	0.9432	8.8257	43	0.0000	0.0000	c2 > c5 *
c1-c5	1.2216	8.6026	43	0.0000	0.0000	c1 > c5 *
c3-c5	0.6420	7.8833	43	0.0000	0.0000	c3 > c5 *
c1-c3	0.5795	6.5982	43	0.0000	0.0000	c1 > c3 *
c3-c4	0.3409	5.6001	43	0.0000	0.0000	c3 > c4 *
c2-c3	0.3011	5.0614	43	0.0000	0.0000	c2 > c3 *
c1-c2	0.2784	4.3425	43	0.0001	0.0002	c1 > c2 *
c4-c5	0.3011	4.2900	43	0.0001	0.0002	c4 > c5 *

< MULTIPLE COMPARISON for "C at b3" >

== Holm's Sequentially Rejective Bonferroni Procedure ==
== The factor < C at b3 > is analysed as dependent means. ==
== Alpha level is 0.05. ==

Pair	Diff	t-value	df	p	adj.p	
c2-c5	0.7330	8.2873	43	0.0000	0.0000	c2 > c5 *
c1-c5	0.8068	7.1108	43	0.0000	0.0000	c1 > c5 *
c3-c5	0.4716	7.0768	43	0.0000	0.0000	c3 > c5 *
c2-c4	0.5057	6.9780	43	0.0000	0.0000	c2 > c4 *
c1-c4	0.5795	5.8067	43	0.0000	0.0000	c1 > c4 *
c2-c3	0.2614	5.0246	43	0.0000	0.0000	c2 > c3 *
c4-c5	0.2273	4.8081	43	0.0000	0.0001	c4 > c5 *
c1-c3	0.3352	4.7326	43	0.0000	0.0001	c1 > c3 *
c3-c4	0.2443	3.8024	43	0.0004	0.0009	c3 > c4 *
c1-c2	0.0739	1.6433	43	0.1076	0.1076	c1 = c2

< MULTIPLE COMPARISON for "C at b4" >

== Holm's Sequentially Rejective Bonferroni Procedure ==
== The factor < C at b4 > is analysed as dependent means. ==
== Alpha level is 0.05. ==

Pair	Diff	t-value	df	p	adj.p	
c2-c5	0.5000	6.0908	43	0.0000	0.0000	c2 > c5 *
c1-c5	0.5398	5.7595	43	0.0000	0.0000	c1 > c5 *
c1-c4	0.3295	4.6576	43	0.0000	0.0002	c1 > c4 *
c2-c4	0.2898	4.6046	43	0.0000	0.0003	c2 > c4 *
c3-c5	0.2898	4.3901	43	0.0001	0.0004	c3 > c5 *
c4-c5	0.2102	4.3141	43	0.0001	0.0005	c4 > c5 *
c1-c3	0.2500	3.7027	43	0.0006	0.0024	c1 > c3 *

```
 c2-c3  0.2102   3.6237  43  0.0008  0.0024  c2 > c3 *
 c3-c4  0.0795   1.8244  43  0.0750  0.1501  c3 = c4
 c1-c2  0.0398   1.0692  43  0.2909  0.2909  c1 = c2
---------------------------------------------------------

output is over --------------------///
```

```
[[ Simple Effects for b1 ]]

[ AsB-Type Design ]

This output was generated by anovakun 4.8.5 under R version 4.0.2.
It was executed on Mon Oct 11 18:22:27 2021.


<< DESCRIPTIVE STATISTICS >>

-------------------------------
  A   C   n     Mean    S.D.
-------------------------------
 a1  c1  45  -0.0222  0.2601
 a1  c2  45  -0.3000  0.5503
 a1  c3  45  -0.4778  0.5509
 a1  c4  45  -0.5778  0.7108
 a1  c5  45  -0.9000  0.7984
 a2  c1  41   0.0244  0.3298
 a2  c2  41  -0.4207  0.4791
 a2  c3  41  -0.5305  0.6782
 a2  c4  41  -0.7134  0.7171
 a2  c5  41  -1.0610  0.8344
 a3  c1  46  -0.0652  0.2496
 a3  c2  46  -0.4185  0.4946
 a3  c3  46  -0.7337  0.7273
 a3  c4  46  -0.9891  0.9617
 a3  c5  46  -1.5652  1.0832
 a4  c1  44   0.0227  0.3316
 a4  c2  44  -0.2784  0.6057
 a4  c3  44  -0.6080  0.8130
 a4  c4  44  -0.7955  0.9265
 a4  c5  44  -1.0341  0.9684
 a5  c1  44  -0.1875  0.3855
 a5  c2  44  -0.7216  0.6630
 a5  c3  44  -1.0398  0.9384
 a5  c4  44  -1.3409  1.1011
 a5  c5  44  -1.8750  1.2912
-------------------------------


<< SPHERICITY INDICES >>

== Mendoza's Multisample Sphericity Test and Epsilons ==
```

```
--------------------------------------------------------------------
-----
 Effect  Lambda  approx.Chi  df      p         LB     GG     HF
CM
--------------------------------------------------------------------
-----
      C  0.0000    325.9533  49 0.0000 *** 0.2500 0.5988 0.6060
0.6058
--------------------------------------------------------------------
-----
                              LB = lower.bound, GG = Greenhouse-
Geisser
                             HF = Huynh-Feldt-Lecoutre, CM = Chi-
Muller


<< ANOVA TABLE >>

== Adjusted by Greenhouse-Geisser's Epsilon ==
== This data is UNBALANCED!! ==
== Type III SS is applied. ==

-------------------------------------------------------------------
   Source       SS     df      MS  F-ratio  p-value      G.eta^2
-------------------------------------------------------------------
        A  48.1947      4 12.0487   6.0163   0.0001 ***   0.0737
    s x A 430.5769    215  2.0027
-------------------------------------------------------------------
        C 192.1547    2.4 80.2311 236.2301   0.0000 ***   0.2409
    A x C  13.3433   9.58  1.3928   4.1010   0.0000 ***   0.0216
s x A x C 174.8857 514.93  0.3396
-------------------------------------------------------------------
    Total 860.4716   1099  0.7830
                       +p < .10, *p < .05, **p < .01, ***p < .001


<< POST ANALYSES >>

< MULTIPLE COMPARISON for "A" >

== Holm's Sequentially Rejective Bonferroni Procedure ==
== The factor < A > is analysed as independent means. ==
== Alpha level is 0.05. ==

---------------------------
  A   n     Mean    S.D.
---------------------------
 a1 225  -0.4556  0.6650
 a2 205  -0.5402  0.7220
 a3 230  -0.7543  0.9153
 a4 220  -0.5386  0.8465
 a5 220  -1.0330  1.0855
---------------------------
```

Pair	Diff	t-value	df	p	adj.p	
a1-a5	0.5774	4.3032	215	0.0000	0.0003	a1 > a5 *
a4-a5	0.4943	3.6635	215	0.0003	0.0028	a4 > a5 *
a2-a5	0.4927	3.5866	215	0.0004	0.0033	a2 > a5 *
a1-a3	0.2988	2.2517	215	0.0254	0.1775	a1 = a3
a3-a5	0.2786	2.0876	215	0.0380	0.2281	a3 = a5
a3-a4	-0.2157	1.6164	215	0.1075	0.5374	a3 = a4
a2-a3	0.2141	1.5751	215	0.1167	0.5374	a2 = a3
a1-a2	0.0847	0.6198	215	0.5360	1.0000	a1 = a2
a1-a4	0.0831	0.6192	215	0.5365	1.0000	a1 = a4
a2-a4	-0.0016	0.0117	215	0.9907	1.0000	a2 = a4

< MULTIPLE COMPARISON for "C" >

== Holm's Sequentially Rejective Bonferroni Procedure ==
== The factor < C > is analysed as dependent means. ==
== Alpha level is 0.05. ==

C	n	Mean	S.D.
c1	220	-0.0456	0.3211
c2	220	-0.4278	0.5802
c3	220	-0.6779	0.7724
c4	220	-0.8833	0.9288
c5	220	-1.2871	1.0709

Pair	Diff	t-value	df	p	adj.p	
c1-c5	1.2415	20.0876	215	0.0000	0.0000	c1 > c5 *
c2-c5	0.8592	18.1716	215	0.0000	0.0000	c2 > c5 *
c3-c5	0.6091	17.1506	215	0.0000	0.0000	c3 > c5 *
c1-c4	0.8378	15.4408	215	0.0000	0.0000	c1 > c4 *
c1-c3	0.6324	14.7801	215	0.0000	0.0000	c1 > c3 *
c1-c2	0.3823	11.7566	215	0.0000	0.0000	c1 > c2 *
c2-c4	0.4555	11.5398	215	0.0000	0.0000	c2 > c4 *
c4-c5	0.4037	9.8876	215	0.0000	0.0000	c4 > c5 *
c2-c3	0.2501	8.3961	215	0.0000	0.0000	c2 > c3 *
c3-c4	0.2054	5.8015	215	0.0000	0.0000	c3 > c4 *

< SIMPLE EFFECTS for "A x C" INTERACTION >

Effect	Lambda	approx.Chi	df	p	LB	GG	HF

```
CM
--------------------------------------------------------------------
------
 C at a1  0.0000     38.8624   9 0.0000 *** 0.2500 0.6910 0.7419
0.7374
 C at a2  0.0000     68.4244   9 0.0000 *** 0.2500 0.5054 0.5324
0.5285
 C at a3  0.0000     72.0489   9 0.0000 *** 0.2500 0.5400 0.5682
0.5649
 C at a4  0.0000     50.5430   9 0.0000 *** 0.2500 0.6845 0.7357
0.7310
 C at a5  0.0000     81.3738   9 0.0000 *** 0.2500 0.4677 0.4882
0.4851
--------------------------------------------------------------------
------
                               LB = lower.bound, GG = Greenhouse-
Geisser
                              HF = Huynh-Feldt-Lecoutre, CM = Chi-
Muller

--------------------------------------------------------------------
-
     Source       SS     df       MS  F-ratio  p-value      G.eta^2
--------------------------------------------------------------------
-
    A at c1   1.3343      4  0.3336   3.3749   0.0106 *     0.0591
   Er at c1  21.2506    215  0.0988
--------------------------------------------------------------------
-
    A at c2   5.5207      4  1.3802   4.3516   0.0021 **    0.0749
   Er at c2  68.1906    215  0.3172
--------------------------------------------------------------------
-
    A at c3   8.8129      4  2.2032   3.8877   0.0045 **    0.0675
   Er at c3 121.8451    215  0.5667
--------------------------------------------------------------------
-
    A at c4  15.4516      4  3.8629   4.7879   0.0010 **    0.0818
   Er at c4 173.4629    215  0.8068
--------------------------------------------------------------------
-
    A at c5  30.4185      4  7.6046   7.4078   0.0000 ***   0.1211
   Er at c5 220.7133    215  1.0266
--------------------------------------------------------------------
-
    C at a1  19.1222   2.76  6.9186  31.0440   0.0000 ***   0.1930
s x C at a1  27.1028 121.61  0.2229
--------------------------------------------------------------------
-
    C at a2  26.0079   2.02 12.8654  38.3639   0.0000 ***   0.2445
s x C at a2  27.1171  80.86  0.3354
--------------------------------------------------------------------
-
    C at a3  59.8353   2.16 27.6992  61.2444   0.0000 ***   0.3119
```

```
s x C at a3  43.9647  97.21  0.4523
--------------------------------------------------------------------
-
    C at a4  30.7597   2.74 11.2340  37.1375   0.0000 ***   0.1960
s x C at a4  35.6153 117.74  0.3025
--------------------------------------------------------------------
-
    C at a5  71.0892   1.87 37.9998  74.4013   0.0000 ***   0.2755
s x C at a5  41.0858  80.44  0.5107
--------------------------------------------------------------------
-
                         +p < .10, *p < .05, **p < .01, ***p < .001


< MULTIPLE COMPARISON for "A at c1" >

== Holm's Sequentially Rejective Bonferroni Procedure ==
== The factor < A at c1 > is analysed as independent means. ==
== Alpha level is 0.05. ==

----------------------------------------------------------
  Pair     Diff  t-value  df       p   adj.p
----------------------------------------------------------
 a4-a5   0.2102   3.1364 215  0.0019  0.0195  a4 > a5 *
 a2-a5   0.2119   3.1049 215  0.0022  0.0195  a2 > a5 *
 a1-a5   0.1653   2.4796 215  0.0139  0.1114  a1 = a5
 a3-a5   0.1223   1.8445 215  0.0665  0.4654  a3 = a5
 a2-a3   0.0896   1.3271 215  0.1859  1.0000  a2 = a3
 a3-a4  -0.0879   1.3266 215  0.1861  1.0000  a3 = a4
 a1-a2  -0.0466   0.6867 215  0.4930  1.0000  a1 = a2
 a1-a4  -0.0449   0.6744 215  0.5008  1.0000  a1 = a4
 a1-a3   0.0430   0.6523 215  0.5149  1.0000  a1 = a3
 a2-a4   0.0017   0.0244 215  0.9806  1.0000  a2 = a4
----------------------------------------------------------


< MULTIPLE COMPARISON for "A at c2" >

== Holm's Sequentially Rejective Bonferroni Procedure ==
== The factor < A at c2 > is analysed as independent means. ==
== Alpha level is 0.05. ==

----------------------------------------------------------
  Pair     Diff  t-value  df       p   adj.p
----------------------------------------------------------
 a4-a5   0.4432   3.6911 215  0.0003  0.0028  a4 > a5 *
 a1-a5   0.4216   3.5309 215  0.0005  0.0046  a1 > a5 *
 a3-a5   0.3031   2.5524 215  0.0114  0.0911  a3 = a5
 a2-a5   0.3009   2.4611 215  0.0146  0.1025  a2 = a5
 a3-a4  -0.1401   1.1795 215  0.2395  1.0000  a3 = a4
 a2-a4  -0.1423   1.1642 215  0.2456  1.0000  a2 = a4
 a1-a3   0.1185   1.0034 215  0.3168  1.0000  a1 = a3
 a1-a2   0.1207   0.9929 215  0.3219  1.0000  a1 = a2
 a1-a4  -0.0216   0.1808 215  0.8567  1.0000  a1 = a4
```

```
 a2-a3  -0.0023   0.0186 215  0.9852  1.0000  a2 = a3
----------------------------------------------------------


< MULTIPLE COMPARISON for "A at c3" >

== Holm's Sequentially Rejective Bonferroni Procedure ==
== The factor < A at c3 > is analysed as independent means. ==
== Alpha level is 0.05. ==

----------------------------------------------------------
  Pair     Diff  t-value  df       p   adj.p
----------------------------------------------------------
 a1-a5   0.5620   3.5212 215  0.0005  0.0052  a1 > a5 *
 a2-a5   0.5093   3.1166 215  0.0021  0.0187  a2 > a5 *
 a4-a5   0.4318   2.6905 215  0.0077  0.0616  a4 = a5
 a3-a5   0.3061   1.9281 215  0.0552  0.3861  a3 = a5
 a1-a3   0.2559   1.6214 215  0.1064  0.6384  a1 = a3
 a2-a3   0.2032   1.2568 215  0.2102  1.0000  a2 = a3
 a1-a4   0.1302   0.8156 215  0.4156  1.0000  a1 = a4
 a3-a4  -0.1257   0.7921 215  0.4292  1.0000  a3 = a4
 a2-a4   0.0775   0.4741 215  0.6359  1.0000  a2 = a4
 a1-a2   0.0527   0.3243 215  0.7460  1.0000  a1 = a2
----------------------------------------------------------


< MULTIPLE COMPARISON for "A at c4" >

== Holm's Sequentially Rejective Bonferroni Procedure ==
== The factor < A at c4 > is analysed as independent means. ==
== Alpha level is 0.05. ==

----------------------------------------------------------
  Pair     Diff  t-value  df       p   adj.p
----------------------------------------------------------
 a1-a5   0.7631   4.0073 215  0.0001  0.0008  a1 > a5 *
 a2-a5   0.6275   3.2184 215  0.0015  0.0134  a2 > a5 *
 a4-a5   0.5455   2.8483 215  0.0048  0.0386  a4 > a5 *
 a1-a3   0.4114   2.1842 215  0.0300  0.2102  a1 = a3
 a3-a5   0.3518   1.8572 215  0.0646  0.3879  a3 = a5
 a2-a3   0.2757   1.4292 215  0.1544  0.7720  a2 = a3
 a1-a4   0.2177   1.1431 215  0.2543  1.0000  a1 = a4
 a3-a4  -0.1937   1.0225 215  0.3077  1.0000  a3 = a4
 a1-a2   0.1356   0.6994 215  0.4850  1.0000  a1 = a2
 a2-a4   0.0820   0.4208 215  0.6743  1.0000  a2 = a4
----------------------------------------------------------


< MULTIPLE COMPARISON for "A at c5" >

== Holm's Sequentially Rejective Bonferroni Procedure ==
== The factor < A at c5 > is analysed as independent means. ==
== Alpha level is 0.05. ==
```

Pair	Diff	t-value	df	p	adj.p	
a1-a5	0.9750	4.5389	215	0.0000	0.0001	a1 > a5 *
a4-a5	0.8409	3.8928	215	0.0001	0.0012	a4 > a5 *
a2-a5	0.8140	3.7013	215	0.0003	0.0022	a2 > a5 *
a1-a3	0.6652	3.1314	215	0.0020	0.0139	a1 > a3 *
a3-a4	-0.5311	2.4859	215	0.0137	0.0821	a3 = a4
a2-a3	0.5042	2.3172	215	0.0214	0.1072	a2 = a3
a3-a5	0.3098	1.4499	215	0.1485	0.5941	a3 = a5
a1-a2	0.1610	0.7359	215	0.4626	1.0000	a1 = a2
a1-a4	0.1341	0.6242	215	0.5331	1.0000	a1 = a4
a2-a4	-0.0269	0.1222	215	0.9028	1.0000	a2 = a4

< MULTIPLE COMPARISON for "C at a1" >

== Holm's Sequentially Rejective Bonferroni Procedure ==
== The factor < C at a1 > is analysed as dependent means. ==
== Alpha level is 0.05. ==

Pair	Diff	t-value	df	p	adj.p	
c1-c5	0.8778	8.0016	44	0.0000	0.0000	c1 > c5 *
c2-c5	0.6000	7.2529	44	0.0000	0.0000	c2 > c5 *
c1-c3	0.4556	6.5217	44	0.0000	0.0000	c1 > c3 *
c3-c5	0.4222	6.0237	44	0.0000	0.0000	c3 > c5 *
c1-c4	0.5556	5.1844	44	0.0000	0.0000	c1 > c4 *
c4-c5	0.3222	3.9839	44	0.0003	0.0013	c4 > c5 *
c1-c2	0.2778	3.7650	44	0.0005	0.0020	c1 > c2 *
c2-c3	0.1778	3.3877	44	0.0015	0.0045	c2 > c3 *
c2-c4	0.2778	3.2856	44	0.0020	0.0045	c2 > c4 *
c3-c4	0.1000	1.2499	44	0.2180	0.2180	c3 = c4

< MULTIPLE COMPARISON for "C at a2" >

== Holm's Sequentially Rejective Bonferroni Procedure ==
== The factor < C at a2 > is analysed as dependent means. ==
== Alpha level is 0.05. ==

Pair	Diff	t-value	df	p	adj.p	
c1-c5	1.0854	7.7912	40	0.0000	0.0000	c1 > c5 *
c2-c5	0.6402	6.8670	40	0.0000	0.0000	c2 > c5 *
c3-c5	0.5305	6.6618	40	0.0000	0.0000	c3 > c5 *
c1-c4	0.7378	6.2824	40	0.0000	0.0000	c1 > c4 *
c1-c2	0.4451	5.4502	40	0.0000	0.0000	c1 > c2 *
c4-c5	0.3476	5.0205	40	0.0000	0.0001	c4 > c5 *
c1-c3	0.5549	4.9704	40	0.0000	0.0001	c1 > c3 *

```
 c2-c4  0.2927   4.5663  40  0.0000  0.0001  c2 > c4 *
 c3-c4  0.1829   3.3979  40  0.0015  0.0031  c3 > c4 *
 c2-c3  0.1098   1.8526  40  0.0713  0.0713  c2 = c3
---------------------------------------------------------


< MULTIPLE COMPARISON for "C at a3" >

== Holm's Sequentially Rejective Bonferroni Procedure ==
== The factor < C at a3 > is analysed as dependent means. ==
== Alpha level is 0.05. ==

---------------------------------------------------------
  Pair    Diff  t-value  df       p   adj.p
---------------------------------------------------------
 c1-c5  1.5000   9.7619  45  0.0000  0.0000  c1 > c5 *
 c2-c5  1.1467   9.6556  45  0.0000  0.0000  c2 > c5 *
 c3-c5  0.8315   9.0418  45  0.0000  0.0000  c3 > c5 *
 c1-c4  0.9239   7.1713  45  0.0000  0.0000  c1 > c4 *
 c1-c3  0.6685   6.9534  45  0.0000  0.0000  c1 > c3 *
 c2-c4  0.5707   6.1482  45  0.0000  0.0000  c2 > c4 *
 c4-c5  0.5761   5.7218  45  0.0000  0.0000  c4 > c5 *
 c1-c2  0.3533   5.1284  45  0.0000  0.0000  c1 > c2 *
 c2-c3  0.3152   4.4409  45  0.0001  0.0001  c2 > c3 *
 c3-c4  0.2554   3.3996  45  0.0014  0.0014  c3 > c4 *
---------------------------------------------------------


< MULTIPLE COMPARISON for "C at a4" >

== Holm's Sequentially Rejective Bonferroni Procedure ==
== The factor < C at a4 > is analysed as dependent means. ==
== Alpha level is 0.05. ==

---------------------------------------------------------
  Pair    Diff  t-value  df       p   adj.p
---------------------------------------------------------
 c1-c5  1.0568   8.6322  43  0.0000  0.0000  c1 > c5 *
 c2-c5  0.7557   7.8438  43  0.0000  0.0000  c2 > c5 *
 c1-c4  0.8182   6.7655  43  0.0000  0.0000  c1 > c4 *
 c1-c3  0.6307   6.5463  43  0.0000  0.0000  c1 > c3 *
 c3-c5  0.4261   6.3308  43  0.0000  0.0000  c3 > c5 *
 c2-c4  0.5170   5.7033  43  0.0000  0.0000  c2 > c4 *
 c1-c2  0.3011   4.6463  43  0.0000  0.0001  c1 > c2 *
 c2-c3  0.3295   4.4297  43  0.0001  0.0002  c2 > c3 *
 c4-c5  0.2386   2.0659  43  0.0449  0.0898  c4 = c5
 c3-c4  0.1875   1.8642  43  0.0691  0.0898  c3 = c4
---------------------------------------------------------


< MULTIPLE COMPARISON for "C at a5" >

== Holm's Sequentially Rejective Bonferroni Procedure ==
== The factor < C at a5 > is analysed as dependent means. ==
```

== Alpha level is 0.05. ==

Pair	Diff	t-value	df	p	adj.p	
c1-c5	1.6875	10.6484	43	0.0000	0.0000	c1 > c5 *
c3-c5	0.8352	9.9365	43	0.0000	0.0000	c3 > c5 *
c2-c5	1.1534	9.0069	43	0.0000	0.0000	c2 > c5 *
c1-c4	1.1534	8.9175	43	0.0000	0.0000	c1 > c4 *
c1-c3	0.8523	8.3598	43	0.0000	0.0000	c1 > c3 *
c1-c2	0.5341	7.1869	43	0.0000	0.0000	c1 > c2 *
c4-c5	0.5341	6.7921	43	0.0000	0.0000	c4 > c5 *
c2-c4	0.6193	6.1302	43	0.0000	0.0000	c2 > c4 *
c2-c3	0.3182	4.4220	43	0.0001	0.0001	c2 > c3 *
c3-c4	0.3011	3.9588	43	0.0003	0.0003	c3 > c4 *

output is over --------------------///

[[Simple Effects for b2]]

[AsB-Type Design]

This output was generated by anovakun 4.8.5 under R version 4.0.2.
It was executed on Mon Oct 11 18:22:28 2021.

<< DESCRIPTIVE STATISTICS >>

A	C	n	Mean	S.D.
a1	c1	45	-0.0111	0.1406
a1	c2	45	-0.0944	0.2516
a1	c3	45	-0.2722	0.4018
a1	c4	45	-0.4000	0.4689
a1	c5	45	-0.5278	0.5671
a2	c1	41	-0.0305	0.1695
a2	c2	41	-0.1280	0.2569
a2	c3	41	-0.2378	0.3160
a2	c4	41	-0.5305	0.6713
a2	c5	41	-0.6585	0.6040
a3	c1	46	0.0000	0.0527
a3	c2	46	-0.1576	0.3389
a3	c3	46	-0.3152	0.4231
a3	c4	46	-0.6250	0.5980
a3	c5	46	-0.9402	0.6671
a4	c1	44	0.0455	0.3015
a4	c2	44	-0.2045	0.3707
a4	c3	44	-0.3409	0.4944
a4	c4	44	-0.4602	0.6122
a4	c5	44	-0.8068	0.8758
a5	c1	44	-0.1136	0.2825

```
 a5  c2  44  -0.3920  0.5665
 a5  c3  44  -0.6932  0.7089
 a5  c4  44  -1.0341  0.8134
 a5  c5  44  -1.3352  1.0633
-------------------------------


<< SPHERICITY INDICES >>

== Mendoza's Multisample Sphericity Test and Epsilons ==

--------------------------------------------------------------------
-----
 Effect  Lambda  approx.Chi  df      p         LB     GG     HF
CM
--------------------------------------------------------------------
-----
      C  0.0000    357.6419  49 0.0000 *** 0.2500 0.5736 0.5801
0.5800
--------------------------------------------------------------------
-----
                              LB = lower.bound, GG = Greenhouse-
Geisser
                             HF = Huynh-Feldt-Lecoutre, CM = Chi-
Muller


<< ANOVA TABLE >>

== Adjusted by Greenhouse-Geisser's Epsilon ==
== This data is UNBALANCED!! ==
== Type III SS is applied. ==

-------------------------------------------------------------------
   Source       SS     df      MS  F-ratio  p-value      G.eta^2
-------------------------------------------------------------------
        A  27.7454      4  6.9363   7.5709   0.0000 ***   0.0822
    s x A 196.9807    215  0.9162
-------------------------------------------------------------------
        C  95.5524   2.29 41.6482 182.1085   0.0000 ***   0.2357
    A x C   9.3250   9.18  1.0161   4.4430   0.0000 ***   0.0292
s x A x C 112.8105 493.27  0.2287
-------------------------------------------------------------------
    Total 443.0011   1099  0.4031
                       +p < .10, *p < .05, **p < .01, ***p < .001


<< POST ANALYSES >>

< MULTIPLE COMPARISON for "A" >

== Holm's Sequentially Rejective Bonferroni Procedure ==
== The factor < A > is analysed as independent means. ==
== Alpha level is 0.05. ==
```

A	n	Mean	S.D.
a1	225	-0.2611	0.4367
a2	205	-0.3171	0.5056
a3	230	-0.4076	0.5746
a4	220	-0.3534	0.6301
a5	220	-0.7136	0.8489

Pair	Diff	t-value	df	p	adj.p	
a1-a5	0.4525	4.9862	215	0.0000	0.0000	a1 > a5 *
a2-a5	0.3966	4.2679	215	0.0000	0.0003	a2 > a5 *
a4-a5	0.3602	3.9471	215	0.0001	0.0009	a4 > a5 *
a3-a5	0.3060	3.3903	215	0.0008	0.0058	a3 > a5 *
a1-a3	0.1465	1.6323	215	0.1041	0.6245	a1 = a3
a1-a4	0.0923	1.0170	215	0.3103	1.0000	a1 = a4
a2-a3	0.0905	0.9847	215	0.3259	1.0000	a2 = a3
a1-a2	0.0560	0.6055	215	0.5455	1.0000	a1 = a2
a3-a4	-0.0542	0.6004	215	0.5488	1.0000	a3 = a4
a2-a4	0.0363	0.3911	215	0.6961	1.0000	a2 = a4

< MULTIPLE COMPARISON for "C" >

== Holm's Sequentially Rejective Bonferroni Procedure ==
== The factor < C > is analysed as dependent means. ==
== Alpha level is 0.05. ==

C	n	Mean	S.D.
c1	220	-0.0220	0.2146
c2	220	-0.1953	0.3865
c3	220	-0.3719	0.5110
c4	220	-0.6100	0.6741
c5	220	-0.8537	0.8203

Pair	Diff	t-value	df	p	adj.p	
c2-c5	0.6584	16.4073	215	0.0000	0.0000	c2 > c5 *
c1-c5	0.8318	16.2390	215	0.0000	0.0000	c1 > c5 *
c1-c4	0.5880	14.7609	215	0.0000	0.0000	c1 > c4 *
c2-c4	0.4146	13.7962	215	0.0000	0.0000	c2 > c4 *
c3-c5	0.4818	13.5236	215	0.0000	0.0000	c3 > c5 *
c1-c3	0.3499	11.3278	215	0.0000	0.0000	c1 > c3 *
c3-c4	0.2381	8.1021	215	0.0000	0.0000	c3 > c4 *
c2-c3	0.1765	7.6715	215	0.0000	0.0000	c2 > c3 *

c4-c5	0.2438	7.4918	215	0.0000	0.0000	c4 > c5 *
c1-c2	0.1734	7.4353	215	0.0000	0.0000	c1 > c2 *

< SIMPLE EFFECTS for "A x C" INTERACTION >

Effect	Lambda	approx.Chi	df	p		LB	GG	HF	CM
C at a1	0.0000	61.5209	9	0.0000	***	0.2500	0.5984	0.6352	0.6313
C at a2	0.0000	60.8486	9	0.0000	***	0.2500	0.5910	0.6306	0.6260
C at a3	0.0000	49.3586	9	0.0000	***	0.2500	0.6155	0.6538	0.6500
C at a4	0.0000	72.3410	9	0.0000	***	0.2500	0.5182	0.5449	0.5415
C at a5	0.0000	82.2970	9	0.0000	***	0.2500	0.4524	0.4711	0.4681

LB = lower.bound, GG = Greenhouse-Geisser
HF = Huynh-Feldt-Lecoutre, CM = Chi-Muller

Source	SS	df	MS	F-ratio	p-value		G.eta^2
A at c1	0.6002	4	0.1500	3.4013	0.0101	*	0.0595
Er at c1	9.4847	215	0.0441				
A at c2	2.4155	4	0.6039	4.2841	0.0023	**	0.0738
Er at c2	30.3050	215	0.1410				
A at c3	5.9160	4	1.4790	6.2021	0.0001	***	0.1035
Er at c3	51.2703	215	0.2385				
A at c4	11.1546	4	2.7886	6.7854	0.0000	***	0.1121
Er at c4	88.3599	215	0.4110				
A at c5	16.9842	4	4.2460	7.0023	0.0000	***	0.1153
Er at c5	130.3712	215	0.6064				

```
-
    C at a1   8.1361   2.39  3.3991  23.1500   0.0000 ***   0.1904
s x C at a1  15.4639 105.32  0.1468
--------------------------------------------------------------------
-
    C at a2  11.7378   2.36  4.9650  27.2383   0.0000 ***   0.2251
s x C at a2  17.2372  94.56  0.1823
--------------------------------------------------------------------
-
    C at a3  26.1332   2.46 10.6151  57.0403   0.0000 ***   0.3457
s x C at a3  20.6168 110.78  0.1861
--------------------------------------------------------------------
-
    C at a4  17.5295   2.07  8.4564  25.1295   0.0000 ***   0.2016
s x C at a4  29.9955  89.14  0.3365
--------------------------------------------------------------------
-
    C at a5  41.9278   1.81 23.1695  61.1210   0.0000 ***   0.2656
s x C at a5  29.4972  77.81  0.3791
--------------------------------------------------------------------
-
                         +p < .10, *p < .05, **p < .01, ***p < .001


< MULTIPLE COMPARISON for "A at c1" >

== Holm's Sequentially Rejective Bonferroni Procedure ==
== The factor < A at c1 > is analysed as independent means. ==
== Alpha level is 0.05. ==

-----------------------------------------------------------
  Pair     Diff  t-value  df       p   adj.p
-----------------------------------------------------------
 a4-a5   0.1591   3.5527 215  0.0005  0.0047  a4 > a5 *
 a3-a5   0.1136   2.5657 215  0.0110  0.0988  a3 = a5
 a1-a5   0.1025   2.3024 215  0.0223  0.1782  a1 = a5
 a2-a5   0.0831   1.8238 215  0.0696  0.4870  a2 = a5
 a2-a4  -0.0759   1.6657 215  0.0972  0.5834  a2 = a4
 a1-a4  -0.0566   1.2703 215  0.2054  1.0000  a1 = a4
 a3-a4  -0.0455   1.0263 215  0.3059  1.0000  a3 = a4
 a2-a3  -0.0305   0.6758 215  0.4999  1.0000  a2 = a3
 a1-a2   0.0194   0.4273 215  0.6696  1.0000  a1 = a2
 a1-a3  -0.0111   0.2523 215  0.8010  1.0000  a1 = a3
-----------------------------------------------------------


< MULTIPLE COMPARISON for "A at c2" >

== Holm's Sequentially Rejective Bonferroni Procedure ==
== The factor < A at c2 > is analysed as independent means. ==
== Alpha level is 0.05. ==

----------------------------------------------------------
  Pair    Diff  t-value  df       p   adj.p
```

a1-a5	0.2976	3.7388	215	0.0002	0.0024	a1 > a5 *
a2-a5	0.2640	3.2394	215	0.0014	0.0125	a2 > a5 *
a3-a5	0.2344	2.9612	215	0.0034	0.0273	a3 > a5 *
a4-a5	0.1875	2.3425	215	0.0201	0.1405	a4 = a5
a1-a4	0.1101	1.3832	215	0.1680	1.0000	a1 = a4
a2-a4	0.0765	0.9387	215	0.3490	1.0000	a2 = a4
a1-a3	0.0632	0.8024	215	0.4232	1.0000	a1 = a3
a3-a4	0.0469	0.5929	215	0.5539	1.0000	a3 = a4
a1-a2	0.0336	0.4146	215	0.6789	1.0000	a1 = a2
a2-a3	0.0296	0.3666	215	0.7143	1.0000	a2 = a3

< MULTIPLE COMPARISON for "A at c3" >

== Holm's Sequentially Rejective Bonferroni Procedure ==
== The factor < A at c3 > is analysed as independent means. ==
== Alpha level is 0.05. ==

Pair	Diff	t-value	df	p	adj.p	
a2-a5	0.4554	4.2960	215	0.0000	0.0003	a2 > a5 *
a1-a5	0.4210	4.0660	215	0.0001	0.0006	a1 > a5 *
a3-a5	0.3780	3.6705	215	0.0003	0.0024	a3 > a5 *
a4-a5	0.3523	3.3836	215	0.0009	0.0060	a4 > a5 *
a2-a4	0.1031	0.9727	215	0.3318	1.0000	a2 = a4
a2-a3	0.0774	0.7381	215	0.4613	1.0000	a2 = a3
a1-a4	0.0687	0.6634	215	0.5078	1.0000	a1 = a4
a1-a3	0.0430	0.4199	215	0.6750	1.0000	a1 = a3
a1-a2	-0.0344	0.3264	215	0.7444	1.0000	a1 = a2
a3-a4	0.0257	0.2495	215	0.8032	1.0000	a3 = a4

< MULTIPLE COMPARISON for "A at c4" >

== Holm's Sequentially Rejective Bonferroni Procedure ==
== The factor < A at c4 > is analysed as independent means. ==
== Alpha level is 0.05. ==

Pair	Diff	t-value	df	p	adj.p	
a1-a5	0.6341	4.6653	215	0.0000	0.0001	a1 > a5 *
a4-a5	0.5739	4.1987	215	0.0000	0.0004	a4 > a5 *
a2-a5	0.5036	3.6190	215	0.0004	0.0029	a2 > a5 *
a3-a5	0.4091	3.0262	215	0.0028	0.0195	a3 > a5 *
a1-a3	0.2250	1.6739	215	0.0956	0.5736	a1 = a3
a3-a4	-0.1648	1.2189	215	0.2242	1.0000	a3 = a4
a1-a2	0.1305	0.9428	215	0.3469	1.0000	a1 = a2
a2-a3	0.0945	0.6864	215	0.4932	1.0000	a2 = a3
a2-a4	-0.0703	0.5049	215	0.6141	1.0000	a2 = a4

a1-a4	0.0602	0.4431	215	0.6581	1.0000	a1 = a4

< MULTIPLE COMPARISON for "A at c5" >

== Holm's Sequentially Rejective Bonferroni Procedure ==
== The factor < A at c5 > is analysed as independent means. ==
== Alpha level is 0.05. ==

Pair	Diff	t-value	df	p	adj.p	
a1-a5	0.8074	4.8908	215	0.0000	0.0000	a1 > a5 *
a2-a5	0.6767	4.0034	215	0.0001	0.0008	a2 > a5 *
a4-a5	0.5284	3.1828	215	0.0017	0.0134	a4 > a5 *
a1-a3	0.4124	2.5261	215	0.0123	0.0858	a1 = a3
a3-a5	0.3950	2.4056	215	0.0170	0.1020	a3 = a5
a1-a4	0.2790	1.6902	215	0.0924	0.4622	a1 = a4
a2-a3	0.2817	1.6842	215	0.0936	0.4622	a2 = a3
a2-a4	0.1483	0.8773	215	0.3813	1.0000	a2 = a4
a3-a4	-0.1334	0.8124	215	0.4175	1.0000	a3 = a4
a1-a2	0.1308	0.7778	215	0.4376	1.0000	a1 = a2

< MULTIPLE COMPARISON for "C at a1" >

== Holm's Sequentially Rejective Bonferroni Procedure ==
== The factor < C at a1 > is analysed as dependent means. ==
== Alpha level is 0.05. ==

Pair	Diff	t-value	df	p	adj.p	
c1-c5	0.5167	6.0014	44	0.0000	0.0000	c1 > c5 *
c2-c5	0.4333	5.9720	44	0.0000	0.0000	c2 > c5 *
c1-c4	0.3889	5.5939	44	0.0000	0.0000	c1 > c4 *
c2-c4	0.3056	5.3386	44	0.0000	0.0000	c2 > c4 *
c1-c3	0.2611	4.6968	44	0.0000	0.0002	c1 > c3 *
c3-c5	0.2556	3.7019	44	0.0006	0.0030	c3 > c5 *
c2-c3	0.1778	3.4272	44	0.0013	0.0053	c2 > c3 *
c1-c2	0.0833	2.6220	44	0.0120	0.0359	c1 > c2 *
c4-c5	0.1278	2.3809	44	0.0217	0.0433	c4 > c5 *
c3-c4	0.1278	2.0655	44	0.0448	0.0448	c3 > c4 *

< MULTIPLE COMPARISON for "C at a2" >

== Holm's Sequentially Rejective Bonferroni Procedure ==
== The factor < C at a2 > is analysed as dependent means. ==
== Alpha level is 0.05. ==

Pair	Diff	t-value	df	p	adj.p	
c1-c5	0.6280	7.0164	40	0.0000	0.0000	c1 > c5 *
c2-c5	0.5305	6.5449	40	0.0000	0.0000	c2 > c5 *
c3-c5	0.4207	5.8253	40	0.0000	0.0000	c3 > c5 *
c1-c4	0.5000	5.5627	40	0.0000	0.0000	c1 > c4 *
c2-c4	0.4024	4.7831	40	0.0000	0.0001	c2 > c4 *
c1-c3	0.2073	4.2717	40	0.0001	0.0006	c1 > c3 *
c3-c4	0.2927	3.5962	40	0.0009	0.0035	c3 > c4 *
c1-c2	0.0976	2.8044	40	0.0077	0.0232	c1 > c2 *
c2-c3	0.1098	2.5119	40	0.0161	0.0323	c2 > c3 *
c4-c5	0.1280	1.7271	40	0.0919	0.0919	c4 = c5

< MULTIPLE COMPARISON for "C at a3" >

== Holm's Sequentially Rejective Bonferroni Procedure ==
== The factor < C at a3 > is analysed as dependent means. ==
== Alpha level is 0.05. ==

Pair	Diff	t-value	df	p	adj.p	
c2-c5	0.7826	10.1419	45	0.0000	0.0000	c2 > c5 *
c1-c5	0.9402	9.5007	45	0.0000	0.0000	c1 > c5 *
c3-c5	0.6250	9.4951	45	0.0000	0.0000	c3 > c5 *
c2-c4	0.4674	7.1077	45	0.0000	0.0000	c2 > c4 *
c1-c4	0.6250	7.0882	45	0.0000	0.0000	c1 > c4 *
c1-c3	0.3152	5.0534	45	0.0000	0.0000	c1 > c3 *
c4-c5	0.3152	4.9035	45	0.0000	0.0001	c4 > c5 *
c3-c4	0.3098	4.7585	45	0.0000	0.0001	c3 > c4 *
c1-c2	0.1576	3.1537	45	0.0029	0.0057	c1 > c2 *
c2-c3	0.1576	2.9472	45	0.0051	0.0057	c2 > c3 *

< MULTIPLE COMPARISON for "C at a4" >

== Holm's Sequentially Rejective Bonferroni Procedure ==
== The factor < C at a4 > is analysed as dependent means. ==
== Alpha level is 0.05. ==

Pair	Diff	t-value	df	p	adj.p	
c1-c5	0.8523	6.0405	43	0.0000	0.0000	c1 > c5 *
c2-c5	0.6023	5.7283	43	0.0000	0.0000	c2 > c5 *
c1-c4	0.5057	5.5099	43	0.0000	0.0000	c1 > c4 *
c1-c3	0.3864	4.7664	43	0.0000	0.0002	c1 > c3 *
c2-c4	0.2557	4.5647	43	0.0000	0.0002	c2 > c4 *
c3-c5	0.4659	4.4815	43	0.0001	0.0003	c3 > c5 *
c1-c2	0.2500	3.6762	43	0.0007	0.0026	c1 > c2 *

c4-c5	0.3466	3.6139	43	0.0008	0.0026	c4 > c5 *
c2-c3	0.1364	2.9913	43	0.0046	0.0092	c2 > c3 *
c3-c4	0.1193	2.0262	43	0.0490	0.0490	c3 > c4 *

< MULTIPLE COMPARISON for "C at a5" >

== Holm's Sequentially Rejective Bonferroni Procedure ==
== The factor < C at a5 > is analysed as dependent means. ==
== Alpha level is 0.05. ==

Pair	Diff	t-value	df	p	adj.p	
c2-c4	0.6420	8.9786	43	0.0000	0.0000	c2 > c4 *
c1-c4	0.9205	8.9310	43	0.0000	0.0000	c1 > c4 *
c2-c5	0.9432	8.8257	43	0.0000	0.0000	c2 > c5 *
c1-c5	1.2216	8.6026	43	0.0000	0.0000	c1 > c5 *
c3-c5	0.6420	7.8833	43	0.0000	0.0000	c3 > c5 *
c1-c3	0.5795	6.5982	43	0.0000	0.0000	c1 > c3 *
c3-c4	0.3409	5.6001	43	0.0000	0.0000	c3 > c4 *
c2-c3	0.3011	5.0614	43	0.0000	0.0000	c2 > c3 *
c1-c2	0.2784	4.3425	43	0.0001	0.0002	c1 > c2 *
c4-c5	0.3011	4.2900	43	0.0001	0.0002	c4 > c5 *

output is over --------------------///

[[Simple Effects for b3]]

[AsB-Type Design]

This output was generated by anovakun 4.8.5 under R version 4.0.2.
It was executed on Mon Oct 11 18:22:29 2021.

<< DESCRIPTIVE STATISTICS >>

A	C	n	Mean	S.D.
a1	c1	45	-0.0111	0.1187
a1	c2	45	-0.0333	0.1735
a1	c3	45	-0.0722	0.2038
a1	c4	45	-0.1444	0.2743
a1	c5	45	-0.2278	0.3946
a2	c1	41	0.0488	0.2033
a2	c2	41	-0.0061	0.1044
a2	c3	41	-0.0183	0.2704
a2	c4	41	-0.1890	0.3344
a2	c5	41	-0.2988	0.4944
a3	c1	46	0.0109	0.0906

```
 a3  c2  46  -0.0217  0.2347
 a3  c3  46  -0.2120  0.4624
 a3  c4  46  -0.2065  0.4160
 a3  c5  46  -0.4022  0.5413
 a4  c1  44   0.0114  0.1422
 a4  c2  44  -0.0398  0.2219
 a4  c3  44  -0.1875  0.3150
 a4  c4  44  -0.3182  0.4649
 a4  c5  44  -0.5398  0.6170
 a5  c1  44  -0.0568  0.1857
 a5  c2  44  -0.1307  0.3906
 a5  c3  44  -0.3920  0.5741
 a5  c4  44  -0.6364  0.7576
 a5  c5  44  -0.8636  0.8499
-------------------------------


<< SPHERICITY INDICES >>

== Mendoza's Multisample Sphericity Test and Epsilons ==

--------------------------------------------------------------------
-----
 Effect  Lambda  approx.Chi  df      p         LB     GG     HF
CM
--------------------------------------------------------------------
-----
      C  0.0000    433.2277  49 0.0000 *** 0.2500 0.5696 0.5761
0.5759
--------------------------------------------------------------------
-----
                              LB = lower.bound, GG = Greenhouse-
Geisser
                             HF = Huynh-Feldt-Lecoutre, CM = Chi-
Muller


<< ANOVA TABLE >>

== Adjusted by Greenhouse-Geisser's Epsilon ==
== This data is UNBALANCED!! ==
== Type III SS is applied. ==

-------------------------------------------------------------------
   Source       SS     df      MS  F-ratio  p-value      G.eta^2
-------------------------------------------------------------------
        A  15.2733      4  3.8183   7.7139   0.0000 ***   0.0792
    s x A 106.4231    215  0.4950
-------------------------------------------------------------------
        C  31.8865   2.28 13.9943  96.4349   0.0000 ***   0.1523
    A x C   7.0939   9.11  0.7783   5.3636   0.0000 ***   0.0384
s x A x C  71.0904 489.88  0.1451
-------------------------------------------------------------------
    Total 231.7964   1099  0.2109
```

+p < .10, *p < .05, **p < .01, ***p < .001

<< POST ANALYSES >>

< MULTIPLE COMPARISON for "A" >

== Holm's Sequentially Rejective Bonferroni Procedure ==
== The factor < A > is analysed as independent means. ==
== Alpha level is 0.05. ==

A	n	Mean	S.D.
a1	225	-0.0978	0.2618
a2	205	-0.0927	0.3339
a3	230	-0.1663	0.4104
a4	220	-0.2148	0.4362
a5	220	-0.4159	0.6696

Pair	Diff	t-value	df	p	adj.p	
a1-a5	0.3181	4.7690	215	0.0000	0.0000	a1 > a5 *
a2-a5	0.3232	4.7326	215	0.0000	0.0000	a2 > a5 *
a3-a5	0.2496	3.7620	215	0.0002	0.0017	a3 > a5 *
a4-a5	0.2011	2.9984	215	0.0030	0.0212	a4 > a5 *
a2-a4	0.1221	1.7876	215	0.0752	0.4515	a2 = a4
a1-a4	0.1170	1.7538	215	0.0809	0.4515	a1 = a4
a2-a3	0.0736	1.0894	215	0.2772	1.0000	a2 = a3
a1-a3	0.0685	1.0387	215	0.3001	1.0000	a1 = a3
a3-a4	0.0485	0.7305	215	0.4659	1.0000	a3 = a4
a1-a2	-0.0051	0.0750	215	0.9403	1.0000	a1 = a2

< MULTIPLE COMPARISON for "C" >

== Holm's Sequentially Rejective Bonferroni Procedure ==
== The factor < C > is analysed as dependent means. ==
== Alpha level is 0.05. ==

C	n	Mean	S.D.
c1	220	0.0006	0.1548
c2	220	-0.0463	0.2468
c3	220	-0.1764	0.4079
c4	220	-0.2989	0.5081
c5	220	-0.4664	0.6351

Pair	Diff	t-value	df	p	adj.p	
c2-c5	0.4201	12.4831	215	0.0000	0.0000	c2 > c5 *
c1-c5	0.4670	11.9294	215	0.0000	0.0000	c1 > c5 *
c2-c4	0.2526	10.4034	215	0.0000	0.0000	c2 > c4 *
c1-c4	0.2995	9.8879	215	0.0000	0.0000	c1 > c4 *
c3-c5	0.2900	9.6654	215	0.0000	0.0000	c3 > c5 *
c4-c5	0.1675	7.1571	215	0.0000	0.0000	c4 > c5 *
c1-c3	0.1770	7.0394	215	0.0000	0.0000	c1 > c3 *
c2-c3	0.1301	6.2944	215	0.0000	0.0000	c2 > c3 *
c3-c4	0.1225	5.0479	215	0.0000	0.0000	c3 > c4 *
c1-c2	0.0469	2.9282	215	0.0038	0.0038	c1 > c2 *

< SIMPLE EFFECTS for "A x C" INTERACTION >

Effect	Lambda	approx.Chi	df	p		LB	GG	HF	CM
C at a1	0.0000	49.2256	9	0.0000	***	0.2500	0.5887	0.6241	0.6203
C at a2	0.0000	76.1514	9	0.0000	***	0.2500	0.4715	0.4940	0.4904
C at a3	0.0000	46.2855	9	0.0000	***	0.2500	0.7470	0.8061	0.8014
C at a4	0.0000	103.1570	9	0.0000	***	0.2500	0.4609	0.4806	0.4776
C at a5	0.0000	101.6198	9	0.0000	***	0.2500	0.4273	0.4432	0.4404

LB = lower.bound, GG = Greenhouse-Geisser
HF = Huynh-Feldt-Lecoutre, CM = Chi-Muller

Source	SS	df	MS	F-ratio	p-value		G.eta^2
A at c1	0.2563	4	0.0641	2.7585	0.0288	*	0.0488
Er at c1	4.9937	215	0.0232				
A at c2	0.4167	4	0.1042	1.7339	0.1436	ns	0.0313
Er at c2	12.9182	215	0.0601				
A at c3	3.6221	4	0.9055	5.9333	0.0002	***	0.0994
Er at c3	32.8128	215	0.1526				
A at c4	6.9882	4	1.7471	7.5807	0.0000	***	0.1236
Er at c4	49.5490	215	0.2305				

```
--------------------------------------------------------------------
    A at c5 11.0839       4  2.7710   7.7131   0.0000 ***   0.1255
   Er at c5 77.2397     215  0.3593
--------------------------------------------------------------------
    C at a1  1.4128    2.35  0.6000   8.6792   0.0001 ***   0.0920
s x C at a1  7.1622 103.61  0.0691
--------------------------------------------------------------------
    C at a2  3.4768    1.89  1.8436  13.2790   0.0000 ***   0.1529
s x C at a2 10.4732   75.43  0.1388
--------------------------------------------------------------------
    C at a3  5.1348    2.99  1.7184  13.8651   0.0000 ***   0.1331
s x C at a3 16.6652 134.47  0.1239
--------------------------------------------------------------------
    C at a4  8.7483    1.84  4.7448  24.3848   0.0000 ***   0.2100
s x C at a4 15.4267   79.28  0.1946
--------------------------------------------------------------------
    C at a5 20.2369    1.71 11.8396  40.7333   0.0000 ***   0.2061
s x C at a5 21.3631    73.5  0.2907
--------------------------------------------------------------------
                        +p < .10, *p < .05, **p < .01, ***p < .001


< MULTIPLE COMPARISON for "A at c1" >

== Holm's Sequentially Rejective Bonferroni Procedure ==
== The factor < A at c1 > is analysed as independent means. ==
== Alpha level is 0.05. ==

----------------------------------------------------------
  Pair     Diff  t-value  df       p   adj.p
----------------------------------------------------------
 a2-a5   0.1056   3.1921 215  0.0016  0.0162  a2 > a5 *
 a3-a5   0.0677   2.1062 215  0.0363  0.3271  a3 = a5
 a4-a5   0.0682   2.0984 215  0.0370  0.3271  a4 = a5
 a1-a2  -0.0599   1.8202 215  0.0701  0.4908  a1 = a2
 a1-a5   0.0457   1.4146 215  0.1586  0.9518  a1 = a5
 a2-a3   0.0379   1.1582 215  0.2481  1.0000  a2 = a3
 a2-a4   0.0374   1.1311 215  0.2593  1.0000  a2 = a4
 a1-a4  -0.0225   0.6956 215  0.4875  1.0000  a1 = a4
 a1-a3  -0.0220   0.6879 215  0.4923  1.0000  a1 = a3
 a3-a4  -0.0005   0.0154 215  0.9877  1.0000  a3 = a4
----------------------------------------------------------


< MULTIPLE COMPARISON for "A at c3" >

== Holm's Sequentially Rejective Bonferroni Procedure ==
== The factor < A at c3 > is analysed as independent means. ==
== Alpha level is 0.05. ==

----------------------------------------------------------
  Pair     Diff  t-value  df       p   adj.p
----------------------------------------------------------
 a2-a5   0.3738   4.4075 215  0.0000  0.0002  a2 > a5 *
```

Pair	Diff	t-value	df	p	adj.p	
a1-a5	0.3198	3.8614	215	0.0001	0.0013	a1 > a5 *
a4-a5	0.2045	2.4558	215	0.0148	0.1188	a4 = a5
a2-a3	0.1937	2.3081	215	0.0219	0.1536	a2 = a3
a3-a5	0.1801	2.1861	215	0.0299	0.1793	a3 = a5
a2-a4	0.1692	1.9954	215	0.0473	0.2363	a2 = a4
a1-a3	0.1397	1.7059	215	0.0895	0.3578	a1 = a3
a1-a4	0.1153	1.3918	215	0.1654	0.4963	a1 = a4
a1-a2	-0.0539	0.6394	215	0.5232	1.0000	a1 = a2
a3-a4	-0.0245	0.2969	215	0.7668	1.0000	a3 = a4

< MULTIPLE COMPARISON for "A at c4" >

== Holm's Sequentially Rejective Bonferroni Procedure ==
== The factor < A at c4 > is analysed as independent means. ==
== Alpha level is 0.05. ==

Pair	Diff	t-value	df	p	adj.p	
a1-a5	0.4919	4.8332	215	0.0000	0.0000	a1 > a5 *
a2-a5	0.4473	4.2929	215	0.0000	0.0002	a2 > a5 *
a3-a5	0.4298	4.2461	215	0.0000	0.0003	a3 > a5 *
a4-a5	0.3182	3.1088	215	0.0021	0.0149	a4 > a5 *
a1-a4	0.1737	1.7070	215	0.0893	0.5356	a1 = a4
a2-a4	0.1292	1.2395	215	0.2165	1.0000	a2 = a4
a3-a4	0.1117	1.1030	215	0.2713	1.0000	a3 = a4
a1-a3	0.0621	0.6167	215	0.5381	1.0000	a1 = a3
a1-a2	0.0446	0.4301	215	0.6675	1.0000	a1 = a2
a2-a3	0.0175	0.1697	215	0.8654	1.0000	a2 = a3

< MULTIPLE COMPARISON for "A at c5" >

== Holm's Sequentially Rejective Bonferroni Procedure ==
== The factor < A at c5 > is analysed as independent means. ==
== Alpha level is 0.05. ==

Pair	Diff	t-value	df	p	adj.p	
a1-a5	0.6359	5.0038	215	0.0000	0.0000	a1 > a5 *
a2-a5	0.5649	4.3416	215	0.0000	0.0002	a2 > a5 *
a3-a5	0.4615	3.6511	215	0.0003	0.0026	a3 > a5 *
a4-a5	0.3239	2.5344	215	0.0120	0.0838	a4 = a5
a1-a4	0.3120	2.4552	215	0.0149	0.0893	a1 = a4
a2-a4	0.2410	1.8523	215	0.0654	0.3268	a2 = a4
a1-a3	0.1744	1.3877	215	0.1667	0.6666	a1 = a3
a3-a4	0.1376	1.0887	215	0.2775	0.8326	a3 = a4
a2-a3	0.1034	0.8032	215	0.4228	0.8455	a2 = a3
a1-a2	0.0710	0.5487	215	0.5838	0.8455	a1 = a2

< MULTIPLE COMPARISON for "C at a1" >

== Holm's Sequentially Rejective Bonferroni Procedure ==
== The factor < C at a1 > is analysed as dependent means. ==
== Alpha level is 0.05. ==

Pair	Diff	t-value	df	p	adj.p	
c3-c5	0.1556	3.6175	44	0.0008	0.0076	c3 > c5 *
c2-c5	0.1944	3.5364	44	0.0010	0.0087	c2 > c5 *
c1-c5	0.2167	3.4744	44	0.0012	0.0093	c1 > c5 *
c2-c4	0.1111	3.1623	44	0.0028	0.0198	c2 > c4 *
c1-c4	0.1333	3.0336	44	0.0040	0.0243	c1 > c4 *
c3-c4	0.0722	2.2295	44	0.0309	0.1547	c3 = c4
c4-c5	0.0833	1.9149	44	0.0620	0.2481	c4 = c5
c1-c3	0.0611	1.7132	44	0.0937	0.2812	c1 = c3
c2-c3	0.0389	1.2656	44	0.2123	0.4246	c2 = c3
c1-c2	0.0222	0.7031	44	0.4857	0.4857	c1 = c2

< MULTIPLE COMPARISON for "C at a2" >

== Holm's Sequentially Rejective Bonferroni Procedure ==
== The factor < C at a2 > is analysed as dependent means. ==
== Alpha level is 0.05. ==

Pair	Diff	t-value	df	p	adj.p	
c1-c4	0.2378	4.6074	40	0.0000	0.0004	c1 > c4 *
c1-c5	0.3476	4.5404	40	0.0001	0.0005	c1 > c5 *
c2-c5	0.2927	3.7983	40	0.0005	0.0039	c2 > c5 *
c2-c4	0.1829	3.5921	40	0.0009	0.0062	c2 > c4 *
c3-c5	0.2805	3.5437	40	0.0010	0.0062	c3 > c5 *
c3-c4	0.1707	3.4878	40	0.0012	0.0062	c3 > c4 *
c4-c5	0.1098	2.4632	40	0.0182	0.0727	c4 = c5
c1-c2	0.0549	1.5960	40	0.1184	0.3551	c1 = c2
c1-c3	0.0671	1.5674	40	0.1249	0.3551	c1 = c3
c2-c3	0.0122	0.3497	40	0.7284	0.7284	c2 = c3

< MULTIPLE COMPARISON for "C at a3" >

== Holm's Sequentially Rejective Bonferroni Procedure ==
== The factor < C at a3 > is analysed as dependent means. ==
== Alpha level is 0.05. ==

Pair	Diff	t-value	df	p	adj.p

c2-c5	0.3804	5.6629	45	0.0000	0.0000	c2 > c5 *
c1-c5	0.4130	5.2318	45	0.0000	0.0000	c1 > c5 *
c2-c4	0.1848	3.9946	45	0.0002	0.0019	c2 > c4 *
c1-c4	0.2174	3.5641	45	0.0009	0.0061	c1 > c4 *
c1-c3	0.2228	3.2215	45	0.0024	0.0142	c1 > c3 *
c4-c5	0.1957	3.1012	45	0.0033	0.0166	c4 > c5 *
c2-c3	0.1902	2.9013	45	0.0057	0.0229	c2 > c3 *
c3-c5	0.1902	2.5358	45	0.0148	0.0443	c3 > c5 *
c1-c2	0.0326	0.9476	45	0.3484	0.6968	c1 = c2
c3-c4	-0.0054	0.0885	45	0.9299	0.9299	c3 = c4

< MULTIPLE COMPARISON for "C at a4" >

== Holm's Sequentially Rejective Bonferroni Procedure ==
== The factor < C at a4 > is analysed as dependent means. ==
== Alpha level is 0.05. ==

Pair	Diff	t-value	df	p	adj.p	
c2-c5	0.5000	5.8920	43	0.0000	0.0000	c2 > c5 *
c1-c5	0.5511	5.6785	43	0.0000	0.0000	c1 > c5 *
c3-c5	0.3523	5.2434	43	0.0000	0.0000	c3 > c5 *
c1-c4	0.3295	4.8889	43	0.0000	0.0001	c1 > c4 *
c2-c4	0.2784	4.6518	43	0.0000	0.0002	c2 > c4 *
c2-c3	0.1477	4.2464	43	0.0001	0.0006	c2 > c3 *
c1-c3	0.1989	3.8889	43	0.0003	0.0014	c1 > c3 *
c4-c5	0.2216	3.8094	43	0.0004	0.0014	c4 > c5 *
c3-c4	0.1307	2.2854	43	0.0273	0.0546	c3 = c4
c1-c2	0.0511	1.5939	43	0.1183	0.1183	c1 = c2

< MULTIPLE COMPARISON for "C at a5" >

== Holm's Sequentially Rejective Bonferroni Procedure ==
== The factor < C at a5 > is analysed as dependent means. ==
== Alpha level is 0.05. ==

Pair	Diff	t-value	df	p	adj.p	
c2-c5	0.7330	8.2873	43	0.0000	0.0000	c2 > c5 *
c1-c5	0.8068	7.1108	43	0.0000	0.0000	c1 > c5 *
c3-c5	0.4716	7.0768	43	0.0000	0.0000	c3 > c5 *
c2-c4	0.5057	6.9780	43	0.0000	0.0000	c2 > c4 *
c1-c4	0.5795	5.8067	43	0.0000	0.0000	c1 > c4 *
c2-c3	0.2614	5.0246	43	0.0000	0.0000	c2 > c3 *
c4-c5	0.2273	4.8081	43	0.0000	0.0001	c4 > c5 *
c1-c3	0.3352	4.7326	43	0.0000	0.0001	c1 > c3 *
c3-c4	0.2443	3.8024	43	0.0004	0.0009	c3 > c4 *

```
 c1-c2  0.0739   1.6433  43  0.1076  0.1076  c1 = c2
---------------------------------------------------------

output is over --------------------///


[[ Simple Effects for b4 ]]

[ AsB-Type Design ]

This output was generated by anovakun 4.8.5 under R version 4.0.2.
It was executed on Mon Oct 11 18:22:30 2021.


<< DESCRIPTIVE STATISTICS >>

-------------------------------
  A   C   n     Mean    S.D.
-------------------------------
 a1  c1  45   0.0000  0.0000
 a1  c2  45   0.0000  0.0754
 a1  c3  45  -0.0278  0.1619
 a1  c4  45  -0.1167  0.2900
 a1  c5  45  -0.1056  0.2528
 a2  c1  41   0.0122  0.1364
 a2  c2  41  -0.0183  0.1713
 a2  c3  41  -0.0122  0.3352
 a2  c4  41  -0.0244  0.2359
 a2  c5  41  -0.1463  0.3831
 a3  c1  46   0.0109  0.1577
 a3  c2  46  -0.0109  0.1173
 a3  c3  46  -0.0652  0.2710
 a3  c4  46  -0.0978  0.3139
 a3  c5  46  -0.2880  0.5296
 a4  c1  44   0.0057  0.1373
 a4  c2  44   0.0227  0.1301
 a4  c3  44  -0.0568  0.1693
 a4  c4  44  -0.1193  0.3120
 a4  c5  44  -0.2955  0.5037
 a5  c1  44  -0.0398  0.2347
 a5  c2  44  -0.0795  0.3270
 a5  c3  44  -0.2898  0.5730
 a5  c4  44  -0.3693  0.5943
 a5  c5  44  -0.5795  0.7601
-------------------------------


<< SPHERICITY INDICES >>

== Mendoza's Multisample Sphericity Test and Epsilons ==

--------------------------------------------------------------------
-----
 Effect  Lambda  approx.Chi  df      p         LB     GG     HF
```

```
CM
----------------------------------------------------------------------
-----
      C  0.0000    449.7029  49 0.0000 *** 0.2500 0.6651 0.6742
0.6741
----------------------------------------------------------------------
-----
                              LB = lower.bound, GG = Greenhouse-
Geisser
                             HF = Huynh-Feldt-Lecoutre, CM = Chi-
Muller


<< ANOVA TABLE >>

== Adjusted by Greenhouse-Geisser's Epsilon ==
== This data is UNBALANCED!! ==
== Type III SS is applied. ==

-----------------------------------------------------------------
   Source       SS    df     MS  F-ratio  p-value      G.eta^2
-----------------------------------------------------------------
        A   7.8098     4 1.9525   5.9847   0.0001 ***   0.0595
    s x A  70.1415   215 0.3262
-----------------------------------------------------------------
        C  11.3737  2.66 4.2751  45.8255   0.0000 ***   0.0843
    A x C   3.8199 10.64 0.3590   3.8477   0.0000 ***   0.0300
s x A x C  53.3622   572 0.0933
-----------------------------------------------------------------
    Total 146.6264  1099 0.1334
                     +p < .10, *p < .05, **p < .01, ***p < .001


<< POST ANALYSES >>

< MULTIPLE COMPARISON for "A" >

== Holm's Sequentially Rejective Bonferroni Procedure ==
== The factor < A > is analysed as independent means. ==
== Alpha level is 0.05. ==

---------------------------
  A   n     Mean    S.D.
---------------------------
 a1 225  -0.0500  0.1948
 a2 205  -0.0378  0.2725
 a3 230  -0.0902  0.3284
 a4 220  -0.0886  0.3080
 a5 220  -0.2716  0.5642
---------------------------

----------------------------------------------------------
  Pair     Diff  t-value  df       p   adj.p
----------------------------------------------------------
```

```
 a2-a5   0.2338   4.2164 215  0.0000  0.0004  a2 > a5 *
 a1-a5   0.2216   4.0917 215  0.0001  0.0005  a1 > a5 *
 a3-a5   0.1814   3.3672 215  0.0009  0.0072  a3 > a5 *
 a4-a5   0.1830   3.3595 215  0.0009  0.0072  a4 > a5 *
 a2-a3   0.0524   0.9554 215  0.3405  1.0000  a2 = a3
 a2-a4   0.0508   0.9168 215  0.3603  1.0000  a2 = a4
 a1-a3   0.0402   0.7509 215  0.4535  1.0000  a1 = a3
 a1-a4   0.0386   0.7134 215  0.4764  1.0000  a1 = a4
 a1-a2  -0.0122   0.2211 215  0.8252  1.0000  a1 = a2
 a3-a4  -0.0016   0.0294 215  0.9766  1.0000  a3 = a4
----------------------------------------------------------


< MULTIPLE COMPARISON for "C" >

== Holm's Sequentially Rejective Bonferroni Procedure ==
== The factor < C > is analysed as dependent means. ==
== Alpha level is 0.05. ==

---------------------------
  C   n     Mean    S.D.
---------------------------
 c1 220  -0.0022  0.1530
 c2 220  -0.0172  0.1866
 c3 220  -0.0904  0.3482
 c4 220  -0.1455  0.3865
 c5 220  -0.2830  0.5369
---------------------------

---------------------------------------------------------
  Pair    Diff  t-value  df       p   adj.p
---------------------------------------------------------
 c1-c5  0.2808   8.9801 215  0.0000  0.0000  c1 > c5 *
 c2-c5  0.2658   8.8318 215  0.0000  0.0000  c2 > c5 *
 c3-c5  0.1926   7.2054 215  0.0000  0.0000  c3 > c5 *
 c1-c4  0.1433   6.2427 215  0.0000  0.0000  c1 > c4 *
 c2-c4  0.1283   6.1465 215  0.0000  0.0000  c2 > c4 *
 c4-c5  0.1375   4.8567 215  0.0000  0.0000  c4 > c5 *
 c1-c3  0.0882   4.4092 215  0.0000  0.0001  c1 > c3 *
 c2-c3  0.0732   4.0493 215  0.0001  0.0002  c2 > c3 *
 c3-c4  0.0551   2.7174 215  0.0071  0.0142  c3 > c4 *
 c1-c2  0.0150   1.2274 215  0.2210  0.2210  c1 = c2
---------------------------------------------------------


< SIMPLE EFFECTS for "A x C" INTERACTION >

--------------------------------------------------------------------
------
  Effect  Lambda  approx.Chi  df      p         LB     GG     HF
CM
--------------------------------------------------------------------
------
 C at a1  0.0000     93.6185   9 0.0000 *** 0.2500 0.6169 0.6564
```

```
0.6524
 C at a2  0.0000     48.4921   9 0.0000 *** 0.2500 0.6645 0.7162
0.7110
 C at a3  0.0000     94.4459   9 0.0000 *** 0.2500 0.5347 0.5622
0.5589
 C at a4  0.0000     70.6986   9 0.0000 *** 0.2500 0.5529 0.5841
0.5804
 C at a5  0.0000     71.8027   9 0.0000 *** 0.2500 0.5170 0.5436
0.5401
--------------------------------------------------------------------
------
                               LB = lower.bound, GG = Greenhouse-
Geisser
                              HF = Huynh-Feldt-Lecoutre, CM = Chi-
Muller

-------------------------------------------------------------------
     Source      SS      df      MS  F-ratio  p-value      G.eta^2
-------------------------------------------------------------------
    A at c1  0.0814       4 0.0204   0.8679   0.4840 ns    0.0159
   Er at c1  5.0424     215 0.0235
-------------------------------------------------------------------
    A at c2  0.2564       4 0.0641   1.8704   0.1167 ns    0.0336
   Er at c2  7.3672     215 0.0343
-------------------------------------------------------------------
    A at c3  2.2549       4 0.5637   4.9874   0.0007 ***   0.0849
   Er at c3 24.3019     215 0.1130
-------------------------------------------------------------------
    A at c4  2.9774       4 0.7443   5.3825   0.0004 ***   0.0910
   Er at c4 29.7326     215 0.1383
-------------------------------------------------------------------
    A at c5  6.0597       4 1.5149   5.7082   0.0002 ***   0.0960
   Er at c5 57.0597     215 0.2654
-------------------------------------------------------------------
    C at a1  0.5861    2.47 0.2375   4.2882   0.0106 *     0.0690
s x C at a1  6.0139  108.57 0.0554
-------------------------------------------------------------------
    C at a2  0.6354    2.66 0.2390   4.5878   0.0064 **    0.0420
s x C at a2  5.5396  106.32 0.0521
-------------------------------------------------------------------
    C at a3  2.5913    2.14 1.2116   7.9958   0.0005 ***   0.1050
s x C at a3 14.5837   96.25 0.1515
-------------------------------------------------------------------
    C at a4  2.9051    2.21 1.3137  11.1836   0.0000 ***   0.1399
s x C at a4 11.1699   95.09 0.1175
-------------------------------------------------------------------
    C at a5  8.5949    2.07 4.1560  23.0195   0.0000 ***   0.1233
s x C at a5 16.0551   88.93 0.1805
-------------------------------------------------------------------
                       +p < .10, *p < .05, **p < .01, ***p < .001


< MULTIPLE COMPARISON for "A at c3" >
```

== Holm's Sequentially Rejective Bonferroni Procedure ==
== The factor < A at c3 > is analysed as independent means. ==
== Alpha level is 0.05. ==

Pair	Diff	t-value	df	p	adj.p	
a2-a5	0.2776	3.8036	215	0.0002	0.0019	a2 > a5 *
a1-a5	0.2620	3.6756	215	0.0003	0.0027	a1 > a5 *
a4-a5	0.2330	3.2500	215	0.0013	0.0107	a4 > a5 *
a3-a5	0.2246	3.1674	215	0.0018	0.0123	a3 > a5 *
a2-a3	0.0530	0.7343	215	0.4636	1.0000	a2 = a3
a2-a4	0.0446	0.6115	215	0.5415	1.0000	a2 = a4
a1-a3	0.0374	0.5311	215	0.5959	1.0000	a1 = a3
a1-a4	0.0290	0.4074	215	0.6841	1.0000	a1 = a4
a1-a2	-0.0156	0.2147	215	0.8302	1.0000	a1 = a2
a3-a4	-0.0084	0.1185	215	0.9058	1.0000	a3 = a4

< MULTIPLE COMPARISON for "A at c4" >

== Holm's Sequentially Rejective Bonferroni Procedure ==
== The factor < A at c4 > is analysed as independent means. ==
== Alpha level is 0.05. ==

Pair	Diff	t-value	df	p	adj.p	
a2-a5	0.3449	4.2731	215	0.0000	0.0003	a2 > a5 *
a3-a5	0.2715	3.4621	215	0.0006	0.0058	a3 > a5 *
a1-a5	0.2527	3.2045	215	0.0016	0.0125	a1 > a5 *
a4-a5	0.2500	3.1532	215	0.0018	0.0129	a4 > a5 *
a2-a4	0.0949	1.1760	215	0.2409	1.0000	a2 = a4
a1-a2	-0.0923	1.1493	215	0.2517	1.0000	a1 = a2
a2-a3	0.0734	0.9194	215	0.3589	1.0000	a2 = a3
a3-a4	0.0215	0.2741	215	0.7843	1.0000	a3 = a4
a1-a3	-0.0188	0.2416	215	0.8093	1.0000	a1 = a3
a1-a4	0.0027	0.0336	215	0.9732	1.0000	a1 = a4

< MULTIPLE COMPARISON for "A at c5" >

== Holm's Sequentially Rejective Bonferroni Procedure ==
== The factor < A at c5 > is analysed as independent means. ==
== Alpha level is 0.05. ==

Pair	Diff	t-value	df	p	adj.p	
a1-a5	0.4740	4.3397	215	0.0000	0.0002	a1 > a5 *
a2-a5	0.4332	3.8740	215	0.0001	0.0013	a2 > a5 *
a3-a5	0.2915	2.6834	215	0.0079	0.0628	a3 = a5

a4-a5	0.2841	2.5866	215	0.0104	0.0725	a4 = a5
a1-a4	0.1899	1.7387	215	0.0835	0.5012	a1 = a4
a1-a3	0.1825	1.6895	215	0.0926	0.5012	a1 = a3
a2-a4	0.1491	1.3335	215	0.1838	0.7352	a2 = a4
a2-a3	0.1417	1.2807	215	0.2017	0.7352	a2 = a3
a1-a2	0.0408	0.3667	215	0.7142	1.0000	a1 = a2
a3-a4	0.0074	0.0682	215	0.9457	1.0000	a3 = a4

< MULTIPLE COMPARISON for "C at a1" >

== Holm's Sequentially Rejective Bonferroni Procedure ==
== The factor < C at a1 > is analysed as dependent means. ==
== Alpha level is 0.05. ==

Pair	Diff	t-value	df	p	adj.p	
c1-c5	0.1056	2.8014	44	0.0075	0.0753	c1 = c5
c2-c5	0.1056	2.7411	44	0.0088	0.0793	c2 = c5
c1-c4	0.1167	2.6988	44	0.0098	0.0793	c1 = c4
c2-c4	0.1167	2.6120	44	0.0123	0.0859	c2 = c4
c3-c5	0.0778	1.9264	44	0.0605	0.3632	c3 = c5
c3-c4	0.0889	1.8353	44	0.0732	0.3661	c3 = c4
c1-c3	0.0278	1.1512	44	0.2559	1.0000	c1 = c3
c2-c3	0.0278	1.0935	44	0.2801	1.0000	c2 = c3
c4-c5	-0.0111	0.1999	44	0.8425	1.0000	c4 = c5
c1-c2	0.0000	0.0000	44	1.0000	1.0000	c1 = c2

< MULTIPLE COMPARISON for "C at a2" >

== Holm's Sequentially Rejective Bonferroni Procedure ==
== The factor < C at a2 > is analysed as dependent means. ==
== Alpha level is 0.05. ==

Pair	Diff	t-value	df	p	adj.p	
c1-c5	0.1585	2.9553	40	0.0052	0.0522	c1 = c5
c3-c5	0.1341	2.8008	40	0.0078	0.0703	c3 = c5
c2-c5	0.1280	2.6724	40	0.0108	0.0868	c2 = c5
c4-c5	0.1220	2.5451	40	0.0149	0.1042	c4 = c5
c1-c4	0.0366	1.2325	40	0.2250	1.0000	c1 = c4
c1-c2	0.0305	1.1516	40	0.2563	1.0000	c1 = c2
c1-c3	0.0244	0.6278	40	0.5337	1.0000	c1 = c3
c3-c4	0.0122	0.4221	40	0.6752	1.0000	c3 = c4
c2-c4	0.0061	0.1835	40	0.8553	1.0000	c2 = c4
c2-c3	-0.0061	0.1332	40	0.8947	1.0000	c2 = c3

< MULTIPLE COMPARISON for "C at a3" >

== Holm's Sequentially Rejective Bonferroni Procedure ==
== The factor < C at a3 > is analysed as dependent means. ==
== Alpha level is 0.05. ==

Pair	Diff	t-value	df	p	adj.p	
c1-c5	0.2989	3.8932	45	0.0003	0.0032	c1 > c5 *
c2-c5	0.2772	3.4445	45	0.0012	0.0112	c2 > c5 *
c3-c5	0.2228	3.4217	45	0.0013	0.0112	c3 > c5 *
c4-c5	0.1902	2.2328	45	0.0306	0.2141	c4 = c5
c2-c4	0.0870	2.1449	45	0.0374	0.2244	c2 = c4
c1-c4	0.1087	2.0479	45	0.0464	0.2322	c1 = c4
c1-c3	0.0761	1.6125	45	0.1138	0.4554	c1 = c3
c2-c3	0.0543	1.5673	45	0.1241	0.4554	c2 = c3
c1-c2	0.0217	0.7265	45	0.4713	0.9425	c1 = c2
c3-c4	0.0326	0.6429	45	0.5236	0.9425	c3 = c4

< MULTIPLE COMPARISON for "C at a4" >

== Holm's Sequentially Rejective Bonferroni Procedure ==
== The factor < C at a4 > is analysed as dependent means. ==
== Alpha level is 0.05. ==

Pair	Diff	t-value	df	p	adj.p	
c2-c5	0.3182	4.3941	43	0.0001	0.0007	c2 > c5 *
c1-c5	0.3011	4.1794	43	0.0001	0.0013	c1 > c5 *
c3-c5	0.2386	3.3252	43	0.0018	0.0145	c3 > c5 *
c2-c4	0.1420	3.1188	43	0.0032	0.0227	c2 > c4 *
c4-c5	0.1761	2.6360	43	0.0116	0.0697	c4 = c5
c1-c4	0.1250	2.5811	43	0.0133	0.0697	c1 = c4
c2-c3	0.0795	2.5462	43	0.0146	0.0697	c2 = c3
c1-c3	0.0625	1.9149	43	0.0622	0.1865	c1 = c3
c3-c4	0.0625	1.2793	43	0.2076	0.4153	c3 = c4
c1-c2	-0.0170	0.6841	43	0.4976	0.4976	c1 = c2

< MULTIPLE COMPARISON for "C at a5" >

== Holm's Sequentially Rejective Bonferroni Procedure ==
== The factor < C at a5 > is analysed as dependent means. ==
== Alpha level is 0.05. ==

Pair	Diff	t-value	df	p	adj.p	
c2-c5	0.5000	6.0908	43	0.0000	0.0000	c2 > c5 *

```
 c1-c5  0.5398   5.7595  43  0.0000  0.0000  c1 > c5 *
 c1-c4  0.3295   4.6576  43  0.0000  0.0002  c1 > c4 *
 c2-c4  0.2898   4.6046  43  0.0000  0.0003  c2 > c4 *
 c3-c5  0.2898   4.3901  43  0.0001  0.0004  c3 > c5 *
 c4-c5  0.2102   4.3141  43  0.0001  0.0005  c4 > c5 *
 c1-c3  0.2500   3.7027  43  0.0006  0.0024  c1 > c3 *
 c2-c3  0.2102   3.6237  43  0.0008  0.0024  c2 > c3 *
 c3-c4  0.0795   1.8244  43  0.0750  0.1501  c3 = c4
 c1-c2  0.0398   1.0692  43  0.2909  0.2909  c1 = c2
---------------------------------------------------------

output is over --------------------///



[[ Simple Effects for c1 ]]

[ AsB-Type Design ]

This output was generated by anovakun 4.8.5 under R version 4.0.2.
It was executed on Mon Oct 11 18:22:31 2021.


<< DESCRIPTIVE STATISTICS >>

-------------------------------
  A   B   n     Mean    S.D.
-------------------------------
 a1  b1  45  -0.0222  0.2601
 a1  b2  45  -0.0111  0.1406
 a1  b3  45  -0.0111  0.1187
 a1  b4  45   0.0000  0.0000
 a2  b1  41   0.0244  0.3298
 a2  b2  41  -0.0305  0.1695
 a2  b3  41   0.0488  0.2033
 a2  b4  41   0.0122  0.1364
 a3  b1  46  -0.0652  0.2496
 a3  b2  46   0.0000  0.0527
 a3  b3  46   0.0109  0.0906
 a3  b4  46   0.0109  0.1577
 a4  b1  44   0.0227  0.3316
 a4  b2  44   0.0455  0.3015
 a4  b3  44   0.0114  0.1422
 a4  b4  44   0.0057  0.1373
 a5  b1  44  -0.1875  0.3855
 a5  b2  44  -0.1136  0.2825
 a5  b3  44  -0.0568  0.1857
 a5  b4  44  -0.0398  0.2347
-------------------------------


<< SPHERICITY INDICES >>

== Mendoza's Multisample Sphericity Test and Epsilons ==
```

```
---------------------------------------------------------------------
-----
 Effect  Lambda  approx.Chi  df      p         LB     GG     HF
CM
---------------------------------------------------------------------
-----
      B  0.0000    293.2677  29 0.0000 *** 0.3333 0.6697 0.6760
0.6759
---------------------------------------------------------------------
-----
                              LB = lower.bound, GG = Greenhouse-
Geisser
                             HF = Huynh-Feldt-Lecoutre, CM = Chi-
Muller


<< ANOVA TABLE >>

== Adjusted by Greenhouse-Geisser's Epsilon ==
== This data is UNBALANCED!! ==
== Type III SS is applied. ==

------------------------------------------------------------------
   Source      SS     df     MS  F-ratio  p-value      G.eta^2
------------------------------------------------------------------
        A  1.6218      4 0.4054   4.1364   0.0030 **    0.0383
    s x A 21.0740    215 0.0980
------------------------------------------------------------------
        B  0.3008   2.01 0.1497   3.2835   0.0382 *     0.0073
    A x B  0.6504   8.04 0.0809   1.7749   0.0797 +     0.0157
s x A x B 19.6976 431.97 0.0456
------------------------------------------------------------------
    Total 43.3520    879 0.0493
                     +p < .10, *p < .05, **p < .01, ***p < .001


<< POST ANALYSES >>

< MULTIPLE COMPARISON for "A" >

== Holm's Sequentially Rejective Bonferroni Procedure ==
== The factor < A > is analysed as independent means. ==
== Alpha level is 0.05. ==

---------------------------
  A   n     Mean    S.D.
---------------------------
 a1 180  -0.0111  0.1582
 a2 164   0.0137  0.2220
 a3 184  -0.0109  0.1586
 a4 176   0.0213  0.2433
 a5 176  -0.0994  0.2855
---------------------------
```

Pair	Diff	t-value	df	p	adj.p	
a4-a5	0.1207	3.6177	215	0.0004	0.0037	a4 > a5 *
a2-a5	0.1132	3.3300	215	0.0010	0.0092	a2 > a5 *
a3-a5	0.0886	2.6829	215	0.0079	0.0629	a3 = a5
a1-a5	0.0883	2.6612	215	0.0084	0.0629	a1 = a5
a1-a4	-0.0324	0.9768	215	0.3298	1.0000	a1 = a4
a3-a4	-0.0322	0.9748	215	0.3308	1.0000	a3 = a4
a1-a2	-0.0248	0.7347	215	0.4633	1.0000	a1 = a2
a2-a3	0.0246	0.7314	215	0.4654	1.0000	a2 = a3
a2-a4	-0.0076	0.2233	215	0.8235	1.0000	a2 = a4
a1-a3	-0.0002	0.0074	215	0.9941	1.0000	a1 = a3

< MULTIPLE COMPARISON for "B" >

== Holm's Sequentially Rejective Bonferroni Procedure ==
== The factor < B > is analysed as dependent means. ==
== Alpha level is 0.05. ==

B	n	Mean	S.D.
b1	220	-0.0456	0.3211
b2	220	-0.0220	0.2146
b3	220	0.0006	0.1548
b4	220	-0.0022	0.1530

Pair	Diff	t-value	df	p	adj.p	
b1-b3	-0.0462	2.2229	215	0.0273	0.1636	b1 = b3
b1-b4	-0.0434	2.0789	215	0.0388	0.1941	b1 = b4
b2-b3	-0.0226	1.6722	215	0.0959	0.3838	b2 = b3
b2-b4	-0.0198	1.5020	215	0.1346	0.4037	b2 = b4
b1-b2	-0.0236	1.2664	215	0.2067	0.4135	b1 = b2
b3-b4	0.0028	0.2836	215	0.7770	0.7770	b3 = b4

< SIMPLE EFFECTS for "A x B" INTERACTION >

Effect	Lambda	approx.Chi	df	p		LB	GG	HF	CM
B at a1	0.0000	55.2157	5	0.0000	***	0.3333	0.5477	0.5661	0.5627

```
 B at a2  0.0000     55.0422   5 0.0000 *** 0.3333 0.5598 0.5816
0.5773
 B at a3  0.0000     70.4796   5 0.0000 *** 0.3333 0.5365 0.5534
0.5502
 B at a4  0.0000     52.3540   5 0.0000 *** 0.3333 0.7092 0.7472
0.7424
 B at a5  0.0000     35.5918   5 0.0000 *** 0.3333 0.6323 0.6606
0.6564
--------------------------------------------------------------------
------
                               LB = lower.bound, GG = Greenhouse-
Geisser
                              HF = Huynh-Feldt-Lecoutre, CM = Chi-
Muller

------------------------------------------------------------------
     Source      SS     df      MS  F-ratio  p-value      G.eta^2
------------------------------------------------------------------
    A at b1  1.3343      4 0.3336   3.3749   0.0106 *     0.0591
   Er at b1 21.2506   215 0.0988
------------------------------------------------------------------
    A at b2  0.6002      4 0.1500   3.4013   0.0101 *     0.0595
   Er at b2  9.4847   215 0.0441
------------------------------------------------------------------
    A at b3  0.2563      4 0.0641   2.7585   0.0288 *     0.0488
   Er at b3  4.9937   215 0.0232
------------------------------------------------------------------
    A at b4  0.0814      4 0.0204   0.8679   0.4840 ns    0.0159
   Er at b4  5.0424   215 0.0235
------------------------------------------------------------------
    B at a1  0.0111  1.64 0.0068   0.1870   0.7868 ns    0.0025
s x B at a1  2.6139  72.3 0.0362
------------------------------------------------------------------
    B at a2  0.1353  1.68 0.0806   1.2443   0.2900 ns    0.0168
s x B at a2  4.3491 67.18 0.0647
------------------------------------------------------------------
    B at a3  0.1848  1.61 0.1148   2.6065   0.0918 +     0.0401
s x B at a3  3.1902 72.42 0.0440
------------------------------------------------------------------
    B at a4  0.0408  2.13 0.0192   0.3245   0.7370 ns    0.0039
s x B at a4  5.4123 91.49 0.0592
------------------------------------------------------------------
    B at a5  0.5866   1.9 0.3093   6.1048   0.0039 **    0.0411
s x B at a5  4.1321 81.56 0.0507
------------------------------------------------------------------
                      +p < .10, *p < .05, **p < .01, ***p < .001


< MULTIPLE COMPARISON for "A at b1" >

== Holm's Sequentially Rejective Bonferroni Procedure ==
== The factor < A at b1 > is analysed as independent means. ==
== Alpha level is 0.05. ==
```

Pair	Diff	t-value	df	p	adj.p	
a4-a5	0.2102	3.1364	215	0.0019	0.0195	a4 > a5 *
a2-a5	0.2119	3.1049	215	0.0022	0.0195	a2 > a5 *
a1-a5	0.1653	2.4796	215	0.0139	0.1114	a1 = a5
a3-a5	0.1223	1.8445	215	0.0665	0.4654	a3 = a5
a2-a3	0.0896	1.3271	215	0.1859	1.0000	a2 = a3
a3-a4	-0.0879	1.3266	215	0.1861	1.0000	a3 = a4
a1-a2	-0.0466	0.6867	215	0.4930	1.0000	a1 = a2
a1-a4	-0.0449	0.6744	215	0.5008	1.0000	a1 = a4
a1-a3	0.0430	0.6523	215	0.5149	1.0000	a1 = a3
a2-a4	0.0017	0.0244	215	0.9806	1.0000	a2 = a4

< MULTIPLE COMPARISON for "A at b2" >

== Holm's Sequentially Rejective Bonferroni Procedure ==
== The factor < A at b2 > is analysed as independent means. ==
== Alpha level is 0.05. ==

Pair	Diff	t-value	df	p	adj.p	
a4-a5	0.1591	3.5527	215	0.0005	0.0047	a4 > a5 *
a3-a5	0.1136	2.5657	215	0.0110	0.0988	a3 = a5
a1-a5	0.1025	2.3024	215	0.0223	0.1782	a1 = a5
a2-a5	0.0831	1.8238	215	0.0696	0.4870	a2 = a5
a2-a4	-0.0759	1.6657	215	0.0972	0.5834	a2 = a4
a1-a4	-0.0566	1.2703	215	0.2054	1.0000	a1 = a4
a3-a4	-0.0455	1.0263	215	0.3059	1.0000	a3 = a4
a2-a3	-0.0305	0.6758	215	0.4999	1.0000	a2 = a3
a1-a2	0.0194	0.4273	215	0.6696	1.0000	a1 = a2
a1-a3	-0.0111	0.2523	215	0.8010	1.0000	a1 = a3

< MULTIPLE COMPARISON for "A at b3" >

== Holm's Sequentially Rejective Bonferroni Procedure ==
== The factor < A at b3 > is analysed as independent means. ==
== Alpha level is 0.05. ==

Pair	Diff	t-value	df	p	adj.p	
a2-a5	0.1056	3.1921	215	0.0016	0.0162	a2 > a5 *
a3-a5	0.0677	2.1062	215	0.0363	0.3271	a3 = a5
a4-a5	0.0682	2.0984	215	0.0370	0.3271	a4 = a5
a1-a2	-0.0599	1.8202	215	0.0701	0.4908	a1 = a2
a1-a5	0.0457	1.4146	215	0.1586	0.9518	a1 = a5
a2-a3	0.0379	1.1582	215	0.2481	1.0000	a2 = a3
a2-a4	0.0374	1.1311	215	0.2593	1.0000	a2 = a4

```
 a1-a4  -0.0225   0.6956 215  0.4875  1.0000  a1 = a4
 a1-a3  -0.0220   0.6879 215  0.4923  1.0000  a1 = a3
 a3-a4  -0.0005   0.0154 215  0.9877  1.0000  a3 = a4
----------------------------------------------------------


< MULTIPLE COMPARISON for "B at a3" >

== Holm's Sequentially Rejective Bonferroni Procedure ==
== The factor < B at a3 > is analysed as dependent means. ==
== Alpha level is 0.05. ==

----------------------------------------------------------
  Pair     Diff  t-value  df       p   adj.p
----------------------------------------------------------
 b1-b3  -0.0761   1.9251  45  0.0606  0.3633  b1 = b3
 b1-b4  -0.0761   1.7078  45  0.0946  0.4728  b1 = b4
 b1-b2  -0.0652   1.6978  45  0.0964  0.4728  b1 = b2
 b2-b3  -0.0109   0.8135  45  0.4202  1.0000  b2 = b3
 b2-b4  -0.0109   0.5303  45  0.5985  1.0000  b2 = b4
 b3-b4   0.0000   0.0000  45  1.0000  1.0000  b3 = b4
----------------------------------------------------------


< MULTIPLE COMPARISON for "B at a5" >

== Holm's Sequentially Rejective Bonferroni Procedure ==
== The factor < B at a5 > is analysed as dependent means. ==
== Alpha level is 0.05. ==

----------------------------------------------------------
  Pair     Diff  t-value  df       p   adj.p
----------------------------------------------------------
 b1-b4  -0.1477   2.8985  43  0.0059  0.0353  b1 < b4 *
 b1-b3  -0.1307   2.8207  43  0.0072  0.0361  b1 < b3 *
 b2-b4  -0.0739   2.6705  43  0.0106  0.0426  b2 < b4 *
 b2-b3  -0.0568   1.9490  43  0.0578  0.1735  b2 = b3
 b1-b2  -0.0739   1.7626  43  0.0851  0.1735  b1 = b2
 b3-b4  -0.0170   0.6841  43  0.4976  0.4976  b3 = b4
----------------------------------------------------------

output is over --------------------///


[[ Simple Effects for c2 ]]

[ AsB-Type Design ]

This output was generated by anovakun 4.8.5 under R version 4.0.2.
It was executed on Mon Oct 11 18:22:31 2021.


<< DESCRIPTIVE STATISTICS >>
```

A	B	n	Mean	S.D.
a1	b1	45	-0.3000	0.5503
a1	b2	45	-0.0944	0.2516
a1	b3	45	-0.0333	0.1735
a1	b4	45	0.0000	0.0754
a2	b1	41	-0.4207	0.4791
a2	b2	41	-0.1280	0.2569
a2	b3	41	-0.0061	0.1044
a2	b4	41	-0.0183	0.1713
a3	b1	46	-0.4185	0.4946
a3	b2	46	-0.1576	0.3389
a3	b3	46	-0.0217	0.2347
a3	b4	46	-0.0109	0.1173
a4	b1	44	-0.2784	0.6057
a4	b2	44	-0.2045	0.3707
a4	b3	44	-0.0398	0.2219
a4	b4	44	0.0227	0.1301
a5	b1	44	-0.7216	0.6630
a5	b2	44	-0.3920	0.5665
a5	b3	44	-0.1307	0.3906
a5	b4	44	-0.0795	0.3270

<< SPHERICITY INDICES >>

== Mendoza's Multisample Sphericity Test and Epsilons ==

Effect	Lambda	approx.Chi	df	p	LB	GG	HF	CM
B	0.0000	314.2335	29	0.0000 ***	0.3333	0.5796	0.5840	0.5838

LB = lower.bound, GG = Greenhouse-Geisser
HF = Huynh-Feldt-Lecoutre, CM = Chi-Muller

<< ANOVA TABLE >>

== Adjusted by Greenhouse-Geisser's Epsilon ==
== This data is UNBALANCED!! ==
== Type III SS is applied. ==

Source	SS	df	MS	F-ratio	p-value	G.eta^2

A	5.8055	4	1.4514	4.5469	0.0015	**	0.0466
s x A	68.6281	215	0.3192				
B	23.2320	1.74	13.3599	99.5929	0.0000	***	0.1636
A x B	2.8038	6.96	0.4031	3.0049	0.0045	**	0.0231
s x A x B	50.1530	373.87	0.1341				
Total	150.5898	879	0.1713				

+p < .10, *p < .05, **p < .01, ***p < .001

<< POST ANALYSES >>

< MULTIPLE COMPARISON for "A" >

== Holm's Sequentially Rejective Bonferroni Procedure ==
== The factor < A > is analysed as independent means. ==
== Alpha level is 0.05. ==

A	n	Mean	S.D.
a1	180	-0.1069	0.3353
a2	164	-0.1433	0.3324
a3	184	-0.1522	0.3639
a4	176	-0.1250	0.3937
a5	176	-0.3310	0.5621

Pair	Diff	t-value	df	p	adj.p	
a1-a5	0.2240	3.7405	215	0.0002	0.0024	a1 > a5 *
a4-a5	0.2060	3.4198	215	0.0007	0.0067	a4 > a5 *
a2-a5	0.1877	3.0606	215	0.0025	0.0199	a2 > a5 *
a3-a5	0.1788	3.0014	215	0.0030	0.0210	a3 > a5 *
a1-a3	0.0452	0.7636	215	0.4459	1.0000	a1 = a3
a1-a2	0.0363	0.5960	215	0.5518	1.0000	a1 = a2
a3-a4	-0.0272	0.4562	215	0.6487	1.0000	a3 = a4
a1-a4	0.0181	0.3015	215	0.7633	1.0000	a1 = a4
a2-a4	-0.0183	0.2983	215	0.7657	1.0000	a2 = a4
a2-a3	0.0089	0.1464	215	0.8838	1.0000	a2 = a3

< MULTIPLE COMPARISON for "B" >

== Holm's Sequentially Rejective Bonferroni Procedure ==
== The factor < B > is analysed as dependent means. ==
== Alpha level is 0.05. ==

B	n	Mean	S.D.

```
---------------------------
 b1 220  -0.4278  0.5802
 b2 220  -0.1953  0.3865
 b3 220  -0.0463  0.2468
 b4 220  -0.0172  0.1866
---------------------------

----------------------------------------------------------
  Pair     Diff  t-value  df       p   adj.p
----------------------------------------------------------
 b1-b4  -0.4106  11.6207 215  0.0000  0.0000  b1 < b4 *
 b1-b3  -0.3815  11.3140 215  0.0000  0.0000  b1 < b3 *
 b2-b3  -0.1490   8.2968 215  0.0000  0.0000  b2 < b3 *
 b2-b4  -0.1781   8.2108 215  0.0000  0.0000  b2 < b4 *
 b1-b2  -0.2325   7.7794 215  0.0000  0.0000  b1 < b2 *
 b3-b4  -0.0291   2.2004 215  0.0288  0.0288  b3 < b4 *
----------------------------------------------------------


< SIMPLE EFFECTS for "A x B" INTERACTION >

--------------------------------------------------------------------
------
  Effect  Lambda  approx.Chi  df      p         LB     GG     HF
CM
--------------------------------------------------------------------
------
 B at a1  0.0000    111.8155   5 0.0000 *** 0.3333 0.4417 0.4502
0.4474
 B at a2  0.0000     61.2685   5 0.0000 *** 0.3333 0.5161 0.5329
0.5290
 B at a3  0.0000     36.2544   5 0.0000 *** 0.3333 0.6605 0.6908
0.6868
 B at a4  0.0000     53.6609   5 0.0000 *** 0.3333 0.5793 0.6015
0.5977
 B at a5  0.0000     42.7628   5 0.0000 *** 0.3333 0.6551 0.6862
0.6819
--------------------------------------------------------------------
------
                               LB = lower.bound, GG = Greenhouse-
Geisser
                              HF = Huynh-Feldt-Lecoutre, CM = Chi-
Muller

------------------------------------------------------------------
     Source      SS    df     MS  F-ratio  p-value      G.eta^2
------------------------------------------------------------------
    A at b1  5.5207     4 1.3802   4.3516   0.0021 **    0.0749
   Er at b1 68.1906   215 0.3172
------------------------------------------------------------------
    A at b2  2.4155     4 0.6039   4.2841   0.0023 **    0.0738
   Er at b2 30.3050   215 0.1410
------------------------------------------------------------------
    A at b3  0.4167     4 0.1042   1.7339   0.1436 ns    0.0313
```

```
   Er at b3 12.9182   215 0.0601
--------------------------------------------------------------------
    A at b4  0.2564     4 0.0641   1.8704   0.1167 ns    0.0336
   Er at b4  7.3672   215 0.0343
--------------------------------------------------------------------
    B at a1  2.4427  1.33 1.8433  11.1185   0.0005 ***   0.1214
s x B at a1  9.6667 58.31 0.1658
--------------------------------------------------------------------
    B at a2  4.5777  1.55 2.9568  23.9641   0.0000 ***   0.2542
s x B at a2  7.6410 61.93 0.1234
--------------------------------------------------------------------
    B at a3  4.9647  1.98 2.5054  24.3889   0.0000 ***   0.2048
s x B at a3  9.1603 89.17 0.1027
--------------------------------------------------------------------
    B at a4  2.5938  1.74 1.4926   9.6721   0.0004 ***   0.0956
s x B at a4 11.5312 74.73 0.1543
--------------------------------------------------------------------
    B at a5 11.4244  1.97 5.8128  40.4194   0.0000 ***   0.2066
s x B at a5 12.1538 84.51 0.1438
--------------------------------------------------------------------
                      +p < .10, *p < .05, **p < .01, ***p < .001


< MULTIPLE COMPARISON for "A at b1" >

== Holm's Sequentially Rejective Bonferroni Procedure ==
== The factor < A at b1 > is analysed as independent means. ==
== Alpha level is 0.05. ==

------------------------------------------------------------
  Pair     Diff  t-value  df       p   adj.p
------------------------------------------------------------
 a4-a5   0.4432   3.6911 215  0.0003  0.0028  a4 > a5 *
 a1-a5   0.4216   3.5309 215  0.0005  0.0046  a1 > a5 *
 a3-a5   0.3031   2.5524 215  0.0114  0.0911  a3 = a5
 a2-a5   0.3009   2.4611 215  0.0146  0.1025  a2 = a5
 a3-a4  -0.1401   1.1795 215  0.2395  1.0000  a3 = a4
 a2-a4  -0.1423   1.1642 215  0.2456  1.0000  a2 = a4
 a1-a3   0.1185   1.0034 215  0.3168  1.0000  a1 = a3
 a1-a2   0.1207   0.9929 215  0.3219  1.0000  a1 = a2
 a1-a4  -0.0216   0.1808 215  0.8567  1.0000  a1 = a4
 a2-a3  -0.0023   0.0186 215  0.9852  1.0000  a2 = a3
------------------------------------------------------------


< MULTIPLE COMPARISON for "A at b2" >

== Holm's Sequentially Rejective Bonferroni Procedure ==
== The factor < A at b2 > is analysed as independent means. ==
== Alpha level is 0.05. ==

-----------------------------------------------------------
  Pair    Diff  t-value  df       p   adj.p
-----------------------------------------------------------
```

a1-a5	0.2976	3.7388	215	0.0002	0.0024	a1 > a5 *
a2-a5	0.2640	3.2394	215	0.0014	0.0125	a2 > a5 *
a3-a5	0.2344	2.9612	215	0.0034	0.0273	a3 > a5 *
a4-a5	0.1875	2.3425	215	0.0201	0.1405	a4 = a5
a1-a4	0.1101	1.3832	215	0.1680	1.0000	a1 = a4
a2-a4	0.0765	0.9387	215	0.3490	1.0000	a2 = a4
a1-a3	0.0632	0.8024	215	0.4232	1.0000	a1 = a3
a3-a4	0.0469	0.5929	215	0.5539	1.0000	a3 = a4
a1-a2	0.0336	0.4146	215	0.6789	1.0000	a1 = a2
a2-a3	0.0296	0.3666	215	0.7143	1.0000	a2 = a3

< MULTIPLE COMPARISON for "B at a1" >

== Holm's Sequentially Rejective Bonferroni Procedure ==
== The factor < B at a1 > is analysed as dependent means. ==
== Alpha level is 0.05. ==

Pair	Diff	t-value	df	p	adj.p	
b1-b4	-0.3000	3.6399	44	0.0007	0.0043	b1 < b4 *
b1-b3	-0.2667	3.5696	44	0.0009	0.0044	b1 < b3 *
b1-b2	-0.2056	2.9619	44	0.0049	0.0197	b1 < b2 *
b2-b3	-0.0611	2.6932	44	0.0100	0.0299	b2 < b3 *
b2-b4	-0.0944	2.6389	44	0.0115	0.0299	b2 < b4 *
b3-b4	-0.0333	1.4306	44	0.1596	0.1596	b3 = b4

< MULTIPLE COMPARISON for "B at a2" >

== Holm's Sequentially Rejective Bonferroni Procedure ==
== The factor < B at a2 > is analysed as dependent means. ==
== Alpha level is 0.05. ==

Pair	Diff	t-value	df	p	adj.p	
b1-b3	-0.4146	5.6124	40	0.0000	0.0000	b1 < b3 *
b1-b4	-0.4024	5.2573	40	0.0000	0.0000	b1 < b4 *
b1-b2	-0.2927	4.8952	40	0.0000	0.0001	b1 < b2 *
b2-b3	-0.1220	3.3758	40	0.0016	0.0049	b2 < b3 *
b2-b4	-0.1098	2.6186	40	0.0124	0.0248	b2 < b4 *
b3-b4	0.0122	0.4669	40	0.6431	0.6431	b3 = b4

< MULTIPLE COMPARISON for "B at a3" >

== Holm's Sequentially Rejective Bonferroni Procedure ==
== The factor < B at a3 > is analysed as dependent means. ==
== Alpha level is 0.05. ==

Pair	Diff	t-value	df	p	adj.p	
b1-b4	-0.4076	5.9637	45	0.0000	0.0000	b1 < b4 *
b1-b3	-0.3967	5.9120	45	0.0000	0.0000	b1 < b3 *
b1-b2	-0.2609	4.1977	45	0.0001	0.0005	b1 < b2 *
b2-b3	-0.1359	3.4485	45	0.0012	0.0037	b2 < b3 *
b2-b4	-0.1467	3.2114	45	0.0024	0.0049	b2 < b4 *
b3-b4	-0.0109	0.3397	45	0.7357	0.7357	b3 = b4

< MULTIPLE COMPARISON for "B at a4" >

== Holm's Sequentially Rejective Bonferroni Procedure ==
== The factor < B at a4 > is analysed as dependent means. ==
== Alpha level is 0.05. ==

Pair	Diff	t-value	df	p	adj.p	
b2-b4	-0.2273	4.4878	43	0.0001	0.0003	b2 < b4 *
b2-b3	-0.1648	3.7046	43	0.0006	0.0030	b2 < b3 *
b1-b4	-0.3011	3.6580	43	0.0007	0.0030	b1 < b4 *
b1-b3	-0.2386	2.8795	43	0.0062	0.0186	b1 < b3 *
b3-b4	-0.0625	2.0459	43	0.0469	0.0938	b3 = b4
b1-b2	-0.0739	1.0187	43	0.3141	0.3141	b1 = b2

< MULTIPLE COMPARISON for "B at a5" >

== Holm's Sequentially Rejective Bonferroni Procedure ==
== The factor < B at a5 > is analysed as dependent means. ==
== Alpha level is 0.05. ==

Pair	Diff	t-value	df	p	adj.p	
b1-b4	-0.6420	7.6221	43	0.0000	0.0000	b1 < b4 *
b1-b3	-0.5909	7.6171	43	0.0000	0.0000	b1 < b3 *
b2-b3	-0.2614	5.0246	43	0.0000	0.0000	b2 < b3 *
b2-b4	-0.3125	4.9175	43	0.0000	0.0000	b2 < b4 *
b1-b2	-0.3295	4.8193	43	0.0000	0.0000	b1 < b2 *
b3-b4	-0.0511	1.5005	43	0.1408	0.1408	b3 = b4

output is over --------------------///

[[Simple Effects for c3]]

[AsB-Type Design]

This output was generated by anovakun 4.8.5 under R version 4.0.2.
It was executed on Mon Oct 11 18:22:32 2021.

<< DESCRIPTIVE STATISTICS >>

A	B	n	Mean	S.D.
a1	b1	45	-0.4778	0.5509
a1	b2	45	-0.2722	0.4018
a1	b3	45	-0.0722	0.2038
a1	b4	45	-0.0278	0.1619
a2	b1	41	-0.5305	0.6782
a2	b2	41	-0.2378	0.3160
a2	b3	41	-0.0183	0.2704
a2	b4	41	-0.0122	0.3352
a3	b1	46	-0.7337	0.7273
a3	b2	46	-0.3152	0.4231
a3	b3	46	-0.2120	0.4624
a3	b4	46	-0.0652	0.2710
a4	b1	44	-0.6080	0.8130
a4	b2	44	-0.3409	0.4944
a4	b3	44	-0.1875	0.3150
a4	b4	44	-0.0568	0.1693
a5	b1	44	-1.0398	0.9384
a5	b2	44	-0.6932	0.7089
a5	b3	44	-0.3920	0.5741
a5	b4	44	-0.2898	0.5730

<< SPHERICITY INDICES >>

== Mendoza's Multisample Sphericity Test and Epsilons ==

Effect	Lambda	approx.Chi	df	p		LB	GG	HF	CM
B	0.0000	241.8911	29	0.0000	***	0.3333	0.6243	0.6296	0.6294

LB = lower.bound, GG = Greenhouse-Geisser
HF = Huynh-Feldt-Lecoutre, CM = Chi-Muller

<< ANOVA TABLE >>

== Adjusted by Greenhouse-Geisser's Epsilon ==
== This data is UNBALANCED!! ==
== Type III SS is applied. ==

Source	SS	df	MS	F-ratio	p-value		G.eta^2
A	18.6307	4	4.6577	7.0625	0.0000	***	0.0749
s x A	141.7905	215	0.6595				
B	44.7761	1.87	23.9084	108.8523	0.0000	***	0.1628
A x B	1.9752	7.49	0.2637	1.2005	0.2993	ns	0.0085
s x A x B	88.4396	402.66	0.2196				
Total	295.7494	879	0.3365				

+p < .10, *p < .05, **p < .01, ***p < .001

<< POST ANALYSES >>

< MULTIPLE COMPARISON for "A" >

== Holm's Sequentially Rejective Bonferroni Procedure ==
== The factor < A > is analysed as independent means. ==
== Alpha level is 0.05. ==

A	n	Mean	S.D.
a1	180	-0.2125	0.4038
a2	164	-0.1997	0.4774
a3	184	-0.3315	0.5539
a4	176	-0.2983	0.5443
a5	176	-0.6037	0.7664

Pair	Diff	t-value	df	p	adj.p		
a2-a5	0.4040	4.5837	215	0.0000	0.0001	a2 > a5	*
a1-a5	0.3912	4.5442	215	0.0000	0.0001	a1 > a5	*
a4-a5	0.3054	3.5278	215	0.0005	0.0041	a4 > a5	*
a3-a5	0.2722	3.1787	215	0.0017	0.0119	a3 > a5	*
a2-a3	0.1318	1.5116	215	0.1321	0.7926	a2 = a3	
a1-a3	0.1190	1.3980	215	0.1635	0.8177	a1 = a3	
a2-a4	0.0986	1.1187	215	0.2645	1.0000	a2 = a4	
a1-a4	0.0858	0.9966	215	0.3201	1.0000	a1 = a4	
a3-a4	-0.0332	0.3881	215	0.6984	1.0000	a3 = a4	
a1-a2	-0.0128	0.1461	215	0.8840	1.0000	a1 = a2	

< MULTIPLE COMPARISON for "B" >

== Holm's Sequentially Rejective Bonferroni Procedure ==
== The factor < B > is analysed as dependent means. ==
== Alpha level is 0.05. ==

B	n	Mean	S.D.
b1	220	-0.6779	0.7724
b2	220	-0.3719	0.5110
b3	220	-0.1764	0.4079
b4	220	-0.0904	0.3482

Pair	Diff	t-value	df	p	adj.p	
b1-b4	-0.5876	12.5319	215	0.0000	0.0000	b1 < b4 *
b1-b3	-0.5015	11.3546	215	0.0000	0.0000	b1 < b3 *
b2-b4	-0.2815	9.4387	215	0.0000	0.0000	b2 < b4 *
b1-b2	-0.3061	8.5004	215	0.0000	0.0000	b1 < b2 *
b2-b3	-0.1955	7.6115	215	0.0000	0.0000	b2 < b3 *
b3-b4	-0.0860	3.8662	215	0.0001	0.0001	b3 < b4 *

output is over --------------------///

[[Simple Effects for c4]]

[AsB-Type Design]

This output was generated by anovakun 4.8.5 under R version 4.0.2.
It was executed on Mon Oct 11 18:22:32 2021.

<< DESCRIPTIVE STATISTICS >>

A	B	n	Mean	S.D.
a1	b1	45	-0.5778	0.7108
a1	b2	45	-0.4000	0.4689
a1	b3	45	-0.1444	0.2743
a1	b4	45	-0.1167	0.2900
a2	b1	41	-0.7134	0.7171
a2	b2	41	-0.5305	0.6713
a2	b3	41	-0.1890	0.3344
a2	b4	41	-0.0244	0.2359
a3	b1	46	-0.9891	0.9617
a3	b2	46	-0.6250	0.5980
a3	b3	46	-0.2065	0.4160
a3	b4	46	-0.0978	0.3139

```
 a4  b1  44  -0.7955  0.9265
 a4  b2  44  -0.4602  0.6122
 a4  b3  44  -0.3182  0.4649
 a4  b4  44  -0.1193  0.3120
 a5  b1  44  -1.3409  1.1011
 a5  b2  44  -1.0341  0.8134
 a5  b3  44  -0.6364  0.7576
 a5  b4  44  -0.3693  0.5943
-------------------------------


<< SPHERICITY INDICES >>

== Mendoza's Multisample Sphericity Test and Epsilons ==

--------------------------------------------------------------------
-----
 Effect  Lambda  approx.Chi  df      p         LB     GG     HF
CM
--------------------------------------------------------------------
-----
      B  0.0000    226.5019  29 0.0000 *** 0.3333 0.6381 0.6437
0.6436
--------------------------------------------------------------------
-----
                              LB = lower.bound, GG = Greenhouse-
Geisser
                             HF = Huynh-Feldt-Lecoutre, CM = Chi-
Muller


<< ANOVA TABLE >>

== Adjusted by Greenhouse-Geisser's Epsilon ==
== This data is UNBALANCED!! ==
== Type III SS is applied. ==

-------------------------------------------------------------------
   Source       SS     df      MS  F-ratio  p-value      G.eta^2
-------------------------------------------------------------------
        A  31.4247      4  7.8562   7.4818   0.0000 ***   0.0844
    s x A 225.7583    215  1.0500
-------------------------------------------------------------------
        B  71.2120   1.91 37.1984 132.7361   0.0000 ***   0.1727
    A x B   5.1471   7.66  0.6722   2.3985   0.0170 *     0.0149
s x A x B 115.3461 411.59  0.2802
-------------------------------------------------------------------
    Total 449.1830    879  0.5110
                       +p < .10, *p < .05, **p < .01, ***p < .001


<< POST ANALYSES >>

< MULTIPLE COMPARISON for "A" >
```

== Holm's Sequentially Rejective Bonferroni Procedure ==
== The factor < A > is analysed as independent means. ==
== Alpha level is 0.05. ==

A	n	Mean	S.D.
a1	180	-0.3097	0.5037
a2	164	-0.3643	0.5935
a3	184	-0.4796	0.7128
a4	176	-0.4233	0.6642
a5	176	-0.8452	0.9094

Pair	Diff	t-value	df	p	adj.p	
a1-a5	0.5354	4.9293	215	0.0000	0.0000	a1 > a5 *
a2-a5	0.4808	4.3235	215	0.0000	0.0002	a2 > a5 *
a4-a5	0.4219	3.8621	215	0.0001	0.0012	a4 > a5 *
a3-a5	0.3656	3.3834	215	0.0009	0.0060	a3 > a5 *
a1-a3	0.1699	1.5815	215	0.1152	0.6914	a1 = a3
a2-a3	0.1153	1.0477	215	0.2960	1.0000	a2 = a3
a1-a4	0.1136	1.0455	215	0.2969	1.0000	a1 = a4
a2-a4	0.0590	0.5302	215	0.5965	1.0000	a2 = a4
a3-a4	-0.0563	0.5213	215	0.6027	1.0000	a3 = a4
a1-a2	0.0546	0.4937	215	0.6221	1.0000	a1 = a2

< MULTIPLE COMPARISON for "B" >

== Holm's Sequentially Rejective Bonferroni Procedure ==
== The factor < B > is analysed as dependent means. ==
== Alpha level is 0.05. ==

B	n	Mean	S.D.
b1	220	-0.8833	0.9288
b2	220	-0.6100	0.6741
b3	220	-0.2989	0.5081
b4	220	-0.1455	0.3865

Pair	Diff	t-value	df	p	adj.p	
b1-b4	-0.7378	13.7046	215	0.0000	0.0000	b1 < b4 *
b2-b4	-0.4645	13.1920	215	0.0000	0.0000	b2 < b4 *
b1-b3	-0.5844	12.0424	215	0.0000	0.0000	b1 < b3 *
b2-b3	-0.3111	10.4774	215	0.0000	0.0000	b2 < b3 *
b1-b2	-0.2734	6.4480	215	0.0000	0.0000	b1 < b2 *

```
 b3-b4  -0.1534   6.2799 215  0.0000  0.0000  b3 < b4 *
----------------------------------------------------------


< SIMPLE EFFECTS for "A x B" INTERACTION >

--------------------------------------------------------------------
------
  Effect  Lambda  approx.Chi  df      p        LB     GG     HF
CM
--------------------------------------------------------------------
------
 B at a1  0.0000     30.4731   5 0.0000 *** 0.3333 0.6807 0.7142
0.7098
 B at a2  0.0000     46.1119   5 0.0000 *** 0.3333 0.6321 0.6627
0.6578
 B at a3  0.0000     45.5669   5 0.0000 *** 0.3333 0.6062 0.6303
0.6266
 B at a4  0.0000     44.0283   5 0.0000 *** 0.3333 0.5955 0.6196
0.6156
 B at a5  0.0000     50.4845   5 0.0000 *** 0.3333 0.5563 0.5760
0.5724
--------------------------------------------------------------------
------
                              LB = lower.bound, GG = Greenhouse-
Geisser
                             HF = Huynh-Feldt-Lecoutre, CM = Chi-
Muller

--------------------------------------------------------------------
     Source        SS    df       MS  F-ratio  p-value      G.eta^2
--------------------------------------------------------------------
    A at b1  15.4516      4  3.8629   4.7879   0.0010 **    0.0818
   Er at b1 173.4629    215  0.8068
--------------------------------------------------------------------
    A at b2  11.1546      4  2.7886   6.7854   0.0000 ***   0.1121
   Er at b2  88.3599    215  0.4110
--------------------------------------------------------------------
    A at b3   6.9882      4  1.7471   7.5807   0.0000 ***   0.1236
   Er at b3  49.5490    215  0.2305
--------------------------------------------------------------------
    A at b4   2.9774      4  0.7443   5.3825   0.0004 ***   0.0910
   Er at b4  29.7326    215  0.1383
--------------------------------------------------------------------
    B at a1   6.5066  2.04  3.1862  15.1856   0.0000 ***   0.1433
s x B at a1  18.8528 89.85  0.2098
--------------------------------------------------------------------
    B at a2  12.1261   1.9  6.3946  27.4006   0.0000 ***   0.2112
s x B at a2  17.7020 75.85  0.2334
--------------------------------------------------------------------
    B at a3  23.0499  1.82 12.6753  34.4349   0.0000 ***   0.2479
s x B at a3  30.1219 81.83  0.3681
--------------------------------------------------------------------
    B at a4  10.7060  1.79  5.9929  18.2453   0.0000 ***   0.1387
```

```
s x B at a4  25.2315 76.82  0.3285
-------------------------------------------------------------------
    B at a5  24.2653  1.67 14.5408  44.5180   0.0000 ***   0.1677
s x B at a5  23.4379 71.76  0.3266
-------------------------------------------------------------------
                        +p < .10, *p < .05, **p < .01, ***p < .001


< MULTIPLE COMPARISON for "A at b1" >

== Holm's Sequentially Rejective Bonferroni Procedure ==
== The factor < A at b1 > is analysed as independent means. ==
== Alpha level is 0.05. ==

---------------------------------------------------------
  Pair     Diff  t-value  df       p   adj.p
---------------------------------------------------------
 a1-a5   0.7631   4.0073 215  0.0001  0.0008  a1 > a5 *
 a2-a5   0.6275   3.2184 215  0.0015  0.0134  a2 > a5 *
 a4-a5   0.5455   2.8483 215  0.0048  0.0386  a4 > a5 *
 a1-a3   0.4114   2.1842 215  0.0300  0.2102  a1 = a3
 a3-a5   0.3518   1.8572 215  0.0646  0.3879  a3 = a5
 a2-a3   0.2757   1.4292 215  0.1544  0.7720  a2 = a3
 a1-a4   0.2177   1.1431 215  0.2543  1.0000  a1 = a4
 a3-a4  -0.1937   1.0225 215  0.3077  1.0000  a3 = a4
 a1-a2   0.1356   0.6994 215  0.4850  1.0000  a1 = a2
 a2-a4   0.0820   0.4208 215  0.6743  1.0000  a2 = a4
---------------------------------------------------------


< MULTIPLE COMPARISON for "A at b2" >

== Holm's Sequentially Rejective Bonferroni Procedure ==
== The factor < A at b2 > is analysed as independent means. ==
== Alpha level is 0.05. ==

---------------------------------------------------------
  Pair     Diff  t-value  df       p   adj.p
---------------------------------------------------------
 a1-a5   0.6341   4.6653 215  0.0000  0.0001  a1 > a5 *
 a4-a5   0.5739   4.1987 215  0.0000  0.0004  a4 > a5 *
 a2-a5   0.5036   3.6190 215  0.0004  0.0029  a2 > a5 *
 a3-a5   0.4091   3.0262 215  0.0028  0.0195  a3 > a5 *
 a1-a3   0.2250   1.6739 215  0.0956  0.5736  a1 = a3
 a3-a4  -0.1648   1.2189 215  0.2242  1.0000  a3 = a4
 a1-a2   0.1305   0.9428 215  0.3469  1.0000  a1 = a2
 a2-a3   0.0945   0.6864 215  0.4932  1.0000  a2 = a3
 a2-a4  -0.0703   0.5049 215  0.6141  1.0000  a2 = a4
 a1-a4   0.0602   0.4431 215  0.6581  1.0000  a1 = a4
---------------------------------------------------------


< MULTIPLE COMPARISON for "A at b3" >
```

== Holm's Sequentially Rejective Bonferroni Procedure ==
== The factor < A at b3 > is analysed as independent means. ==
== Alpha level is 0.05. ==

Pair	Diff	t-value	df	p	adj.p	
a1-a5	0.4919	4.8332	215	0.0000	0.0000	a1 > a5 *
a2-a5	0.4473	4.2929	215	0.0000	0.0002	a2 > a5 *
a3-a5	0.4298	4.2461	215	0.0000	0.0003	a3 > a5 *
a4-a5	0.3182	3.1088	215	0.0021	0.0149	a4 > a5 *
a1-a4	0.1737	1.7070	215	0.0893	0.5356	a1 = a4
a2-a4	0.1292	1.2395	215	0.2165	1.0000	a2 = a4
a3-a4	0.1117	1.1030	215	0.2713	1.0000	a3 = a4
a1-a3	0.0621	0.6167	215	0.5381	1.0000	a1 = a3
a1-a2	0.0446	0.4301	215	0.6675	1.0000	a1 = a2
a2-a3	0.0175	0.1697	215	0.8654	1.0000	a2 = a3

< MULTIPLE COMPARISON for "A at b4" >

== Holm's Sequentially Rejective Bonferroni Procedure ==
== The factor < A at b4 > is analysed as independent means. ==
== Alpha level is 0.05. ==

Pair	Diff	t-value	df	p	adj.p	
a2-a5	0.3449	4.2731	215	0.0000	0.0003	a2 > a5 *
a3-a5	0.2715	3.4621	215	0.0006	0.0058	a3 > a5 *
a1-a5	0.2527	3.2045	215	0.0016	0.0125	a1 > a5 *
a4-a5	0.2500	3.1532	215	0.0018	0.0129	a4 > a5 *
a2-a4	0.0949	1.1760	215	0.2409	1.0000	a2 = a4
a1-a2	-0.0923	1.1493	215	0.2517	1.0000	a1 = a2
a2-a3	0.0734	0.9194	215	0.3589	1.0000	a2 = a3
a3-a4	0.0215	0.2741	215	0.7843	1.0000	a3 = a4
a1-a3	-0.0188	0.2416	215	0.8093	1.0000	a1 = a3
a1-a4	0.0027	0.0336	215	0.9732	1.0000	a1 = a4

< MULTIPLE COMPARISON for "B at a1" >

== Holm's Sequentially Rejective Bonferroni Procedure ==
== The factor < B at a1 > is analysed as dependent means. ==
== Alpha level is 0.05. ==

Pair	Diff	t-value	df	p	adj.p	
b1-b3	-0.4333	4.6192	44	0.0000	0.0002	b1 < b3 *
b1-b4	-0.4611	4.4001	44	0.0001	0.0003	b1 < b4 *
b2-b3	-0.2556	4.3572	44	0.0001	0.0003	b2 < b3 *

b2-b4	-0.2833	3.7057	44	0.0006	0.0018	b2 < b4 *
b1-b2	-0.1778	2.3249	44	0.0248	0.0495	b1 < b2 *
b3-b4	-0.0278	0.4933	44	0.6242	0.6242	b3 = b4

< MULTIPLE COMPARISON for "B at a2" >

== Holm's Sequentially Rejective Bonferroni Procedure ==
== The factor < B at a2 > is analysed as dependent means. ==
== Alpha level is 0.05. ==

Pair	Diff	t-value	df	p	adj.p	
b1-b4	-0.6890	6.4704	40	0.0000	0.0000	b1 < b4 *
b2-b4	-0.5061	5.6711	40	0.0000	0.0000	b2 < b4 *
b1-b3	-0.5244	5.2121	40	0.0000	0.0000	b1 < b3 *
b2-b3	-0.3415	4.5236	40	0.0001	0.0002	b2 < b3 *
b3-b4	-0.1646	4.0566	40	0.0002	0.0004	b3 < b4 *
b1-b2	-0.1829	2.2858	40	0.0276	0.0276	b1 < b2 *

< MULTIPLE COMPARISON for "B at a3" >

== Holm's Sequentially Rejective Bonferroni Procedure ==
== The factor < B at a3 > is analysed as dependent means. ==
== Alpha level is 0.05. ==

Pair	Diff	t-value	df	p	adj.p	
b2-b3	-0.4185	6.9491	45	0.0000	0.0000	b2 < b3 *
b1-b4	-0.8913	6.7745	45	0.0000	0.0000	b1 < b4 *
b2-b4	-0.5272	6.5821	45	0.0000	0.0000	b2 < b4 *
b1-b3	-0.7826	6.4393	45	0.0000	0.0000	b1 < b3 *
b1-b2	-0.3641	3.2671	45	0.0021	0.0042	b1 < b2 *
b3-b4	-0.1087	1.7961	45	0.0792	0.0792	b3 = b4

< MULTIPLE COMPARISON for "B at a4" >

== Holm's Sequentially Rejective Bonferroni Procedure ==
== The factor < B at a4 > is analysed as dependent means. ==
== Alpha level is 0.05. ==

Pair	Diff	t-value	df	p	adj.p	
b2-b4	-0.3409	5.6001	43	0.0000	0.0000	b2 < b4 *
b1-b4	-0.6761	5.4082	43	0.0000	0.0000	b1 < b4 *
b1-b3	-0.4773	4.2069	43	0.0001	0.0005	b1 < b3 *

```
 b3-b4  -0.1989   3.5469  43  0.0010  0.0029  b3 < b4 *
 b1-b2  -0.3352   2.9240  43  0.0055  0.0110  b1 < b2 *
 b2-b3  -0.1420   2.0398  43  0.0475  0.0475  b2 < b3 *
-----------------------------------------------------------


< MULTIPLE COMPARISON for "B at a5" >

== Holm's Sequentially Rejective Bonferroni Procedure ==
== The factor < B at a5 > is analysed as dependent means. ==
== Alpha level is 0.05. ==

-----------------------------------------------------------
  Pair     Diff  t-value  df       p   adj.p
-----------------------------------------------------------
 b2-b4  -0.6648   7.8357  43  0.0000  0.0000  b2 < b4 *
 b1-b4  -0.9716   7.5718  43  0.0000  0.0000  b1 < b4 *
 b1-b3  -0.7045   6.4919  43  0.0000  0.0000  b1 < b3 *
 b2-b3  -0.3977   5.8352  43  0.0000  0.0000  b2 < b3 *
 b3-b4  -0.2670   4.8761  43  0.0000  0.0000  b3 < b4 *
 b1-b2  -0.3068   3.8157  43  0.0004  0.0004  b1 < b2 *
-----------------------------------------------------------

output is over --------------------///


[[ Simple Effects for c5 ]]

[ AsB-Type Design ]

This output was generated by anovakun 4.8.5 under R version 4.0.2.
It was executed on Mon Oct 11 18:22:33 2021.


<< DESCRIPTIVE STATISTICS >>

-------------------------------
  A   B   n     Mean    S.D.
-------------------------------
 a1  b1  45  -0.9000  0.7984
 a1  b2  45  -0.5278  0.5671
 a1  b3  45  -0.2278  0.3946
 a1  b4  45  -0.1056  0.2528
 a2  b1  41  -1.0610  0.8344
 a2  b2  41  -0.6585  0.6040
 a2  b3  41  -0.2988  0.4944
 a2  b4  41  -0.1463  0.3831
 a3  b1  46  -1.5652  1.0832
 a3  b2  46  -0.9402  0.6671
 a3  b3  46  -0.4022  0.5413
 a3  b4  46  -0.2880  0.5296
 a4  b1  44  -1.0341  0.9684
 a4  b2  44  -0.8068  0.8758
 a4  b3  44  -0.5398  0.6170
```

```
 a4  b4  44  -0.2955  0.5037
 a5  b1  44  -1.8750  1.2912
 a5  b2  44  -1.3352  1.0633
 a5  b3  44  -0.8636  0.8499
 a5  b4  44  -0.5795  0.7601
-------------------------------


<< SPHERICITY INDICES >>

== Mendoza's Multisample Sphericity Test and Epsilons ==

--------------------------------------------------------------------
-----
 Effect  Lambda  approx.Chi  df      p         LB     GG     HF
CM
--------------------------------------------------------------------
-----
     B  0.0000    262.8179  29 0.0000 *** 0.3333 0.6465 0.6523
0.6521
--------------------------------------------------------------------
-----
                              LB = lower.bound, GG = Greenhouse-
Geisser
                             HF = Huynh-Feldt-Lecoutre, CM = Chi-
Muller


<< ANOVA TABLE >>

== Adjusted by Greenhouse-Geisser's Epsilon ==
== This data is UNBALANCED!! ==
== Type III SS is applied. ==

-----------------------------------------------------------------
   Source       SS   df      MS  F-ratio  p-value      G.eta^2
-----------------------------------------------------------------
        A  55.5099    4 13.8775   9.1495   0.0000 ***   0.1026
    s x A 326.1023  215  1.5168
-----------------------------------------------------------------
        B 130.6356 1.94 67.3548 176.3333   0.0000 ***   0.2121
    A x B   9.0363 7.76  1.1648   3.0493   0.0027 **    0.0183
s x A x B 159.2816  417  0.3820
-----------------------------------------------------------------
    Total 681.5497  879  0.7754
                     +p < .10, *p < .05, **p < .01, ***p < .001


<< POST ANALYSES >>

< MULTIPLE COMPARISON for "A" >

== Holm's Sequentially Rejective Bonferroni Procedure ==
== The factor < A > is analysed as independent means. ==
```

== Alpha level is 0.05. ==

A	n	Mean	S.D.
a1	180	-0.4403	0.6199
a2	164	-0.5412	0.6941
a3	184	-0.7989	0.8926
a4	176	-0.6690	0.8077
a5	176	-1.1634	1.1180

Pair	Diff	t-value	df	p	adj.p	
a1-a5	0.7231	5.5385	215	0.0000	0.0000	a1 > a5 *
a2-a5	0.6222	4.6549	215	0.0000	0.0001	a2 > a5 *
a4-a5	0.4943	3.7652	215	0.0002	0.0017	a4 > a5 *
a3-a5	0.3644	2.8066	215	0.0055	0.0383	a3 > a5 *
a1-a3	0.3586	2.7777	215	0.0060	0.0383	a1 > a3 *
a2-a3	0.2578	1.9489	215	0.0526	0.2630	a2 = a3
a1-a4	0.2288	1.7522	215	0.0812	0.3247	a1 = a4
a3-a4	-0.1299	1.0002	215	0.3183	0.9550	a3 = a4
a2-a4	0.1279	0.9567	215	0.3398	0.9550	a2 = a4
a1-a2	0.1009	0.7588	215	0.4488	0.9550	a1 = a2

< MULTIPLE COMPARISON for "B" >

== Holm's Sequentially Rejective Bonferroni Procedure ==
== The factor < B > is analysed as dependent means. ==
== Alpha level is 0.05. ==

B	n	Mean	S.D.
b1	220	-1.2871	1.0709
b2	220	-0.8537	0.8203
b3	220	-0.4664	0.6351
b4	220	-0.2830	0.5369

Pair	Diff	t-value	df	p	adj.p	
b1-b4	-1.0041	16.0627	215	0.0000	0.0000	b1 < b4 *
b1-b3	-0.8206	14.4569	215	0.0000	0.0000	b1 < b3 *
b2-b4	-0.5707	12.7956	215	0.0000	0.0000	b2 < b4 *
b2-b3	-0.3873	10.6975	215	0.0000	0.0000	b2 < b3 *
b1-b2	-0.4333	9.1149	215	0.0000	0.0000	b1 < b2 *
b3-b4	-0.1834	6.4854	215	0.0000	0.0000	b3 < b4 *

< SIMPLE EFFECTS for "A x B" INTERACTION >

Effect	Lambda	approx.Chi	df	p		LB	GG	HF	CM
B at a1	0.0000	45.1779	5	0.0000	***	0.3333	0.5842	0.6065	0.6029
B at a2	0.0000	50.1290	5	0.0000	***	0.3333	0.5426	0.5624	0.5583
B at a3	0.0000	35.6414	5	0.0000	***	0.3333	0.6672	0.6983	0.6943
B at a4	0.0000	39.1771	5	0.0000	***	0.3333	0.6820	0.7164	0.7119
B at a5	0.0000	62.2948	5	0.0000	***	0.3333	0.5267	0.5434	0.5400

LB = lower.bound, GG = Greenhouse-Geisser
HF = Huynh-Feldt-Lecoutre, CM = Chi-Muller

Source	SS	df	MS	F-ratio	p-value		G.eta^2
A at b1	30.4185	4	7.6046	7.4078	0.0000	***	0.1211
Er at b1	220.7133	215	1.0266				
A at b2	16.9842	4	4.2460	7.0023	0.0000	***	0.1153
Er at b2	130.3712	215	0.6064				
A at b3	11.0839	4	2.7710	7.7131	0.0000	***	0.1255
Er at b3	77.2397	215	0.3593				
A at b4	6.0597	4	1.5149	5.7082	0.0002	***	0.0960
Er at b4	57.0597	215	0.2654				
B at a1	16.9288	1.75	9.6585	41.7602	0.0000	***	0.2461
s x B at a1	17.8368	77.12	0.2313				
B at a2	20.4432	1.63	12.5585	33.0681	0.0000	***	0.2603
s x B at a2	24.7287	65.11	0.3798				
B at a3	47.1766	2	23.5682	47.9983	0.0000	***	0.3235
s x B at a3	44.2296	90.08	0.4910				
B at a4	13.5749	2.05	6.6351	15.1850	0.0000	***	0.1189
s x B at a4	38.4407	87.98	0.4369				
B at a5	42.5323	1.58	26.9168	53.7185	0.0000	***	0.1944

s x B at a5 34.0458 67.95 0.5011

+p < .10, *p < .05, **p < .01, ***p < .001

< MULTIPLE COMPARISON for "A at b1" >

== Holm's Sequentially Rejective Bonferroni Procedure ==
== The factor < A at b1 > is analysed as independent means. ==
== Alpha level is 0.05. ==

Pair	Diff	t-value	df	p	adj.p	
a1-a5	0.9750	4.5389	215	0.0000	0.0001	a1 > a5 *
a4-a5	0.8409	3.8928	215	0.0001	0.0012	a4 > a5 *
a2-a5	0.8140	3.7013	215	0.0003	0.0022	a2 > a5 *
a1-a3	0.6652	3.1314	215	0.0020	0.0139	a1 > a3 *
a3-a4	-0.5311	2.4859	215	0.0137	0.0821	a3 = a4
a2-a3	0.5042	2.3172	215	0.0214	0.1072	a2 = a3
a3-a5	0.3098	1.4499	215	0.1485	0.5941	a3 = a5
a1-a2	0.1610	0.7359	215	0.4626	1.0000	a1 = a2
a1-a4	0.1341	0.6242	215	0.5331	1.0000	a1 = a4
a2-a4	-0.0269	0.1222	215	0.9028	1.0000	a2 = a4

< MULTIPLE COMPARISON for "A at b2" >

== Holm's Sequentially Rejective Bonferroni Procedure ==
== The factor < A at b2 > is analysed as independent means. ==
== Alpha level is 0.05. ==

Pair	Diff	t-value	df	p	adj.p	
a1-a5	0.8074	4.8908	215	0.0000	0.0000	a1 > a5 *
a2-a5	0.6767	4.0034	215	0.0001	0.0008	a2 > a5 *
a4-a5	0.5284	3.1828	215	0.0017	0.0134	a4 > a5 *
a1-a3	0.4124	2.5261	215	0.0123	0.0858	a1 = a3
a3-a5	0.3950	2.4056	215	0.0170	0.1020	a3 = a5
a1-a4	0.2790	1.6902	215	0.0924	0.4622	a1 = a4
a2-a3	0.2817	1.6842	215	0.0936	0.4622	a2 = a3
a2-a4	0.1483	0.8773	215	0.3813	1.0000	a2 = a4
a3-a4	-0.1334	0.8124	215	0.4175	1.0000	a3 = a4
a1-a2	0.1308	0.7778	215	0.4376	1.0000	a1 = a2

< MULTIPLE COMPARISON for "A at b3" >

== Holm's Sequentially Rejective Bonferroni Procedure ==
== The factor < A at b3 > is analysed as independent means. ==
== Alpha level is 0.05. ==

Pair	Diff	t-value	df	p	adj.p	
a1-a5	0.6359	5.0038	215	0.0000	0.0000	a1 > a5 *
a2-a5	0.5649	4.3416	215	0.0000	0.0002	a2 > a5 *
a3-a5	0.4615	3.6511	215	0.0003	0.0026	a3 > a5 *
a4-a5	0.3239	2.5344	215	0.0120	0.0838	a4 = a5
a1-a4	0.3120	2.4552	215	0.0149	0.0893	a1 = a4
a2-a4	0.2410	1.8523	215	0.0654	0.3268	a2 = a4
a1-a3	0.1744	1.3877	215	0.1667	0.6666	a1 = a3
a3-a4	0.1376	1.0887	215	0.2775	0.8326	a3 = a4
a2-a3	0.1034	0.8032	215	0.4228	0.8455	a2 = a3
a1-a2	0.0710	0.5487	215	0.5838	0.8455	a1 = a2

< MULTIPLE COMPARISON for "A at b4" >

== Holm's Sequentially Rejective Bonferroni Procedure ==
== The factor < A at b4 > is analysed as independent means. ==
== Alpha level is 0.05. ==

Pair	Diff	t-value	df	p	adj.p	
a1-a5	0.4740	4.3397	215	0.0000	0.0002	a1 > a5 *
a2-a5	0.4332	3.8740	215	0.0001	0.0013	a2 > a5 *
a3-a5	0.2915	2.6834	215	0.0079	0.0628	a3 = a5
a4-a5	0.2841	2.5866	215	0.0104	0.0725	a4 = a5
a1-a4	0.1899	1.7387	215	0.0835	0.5012	a1 = a4
a1-a3	0.1825	1.6895	215	0.0926	0.5012	a1 = a3
a2-a4	0.1491	1.3335	215	0.1838	0.7352	a2 = a4
a2-a3	0.1417	1.2807	215	0.2017	0.7352	a2 = a3
a1-a2	0.0408	0.3667	215	0.7142	1.0000	a1 = a2
a3-a4	0.0074	0.0682	215	0.9457	1.0000	a3 = a4

< MULTIPLE COMPARISON for "B at a1" >

== Holm's Sequentially Rejective Bonferroni Procedure ==
== The factor < B at a1 > is analysed as dependent means. ==
== Alpha level is 0.05. ==

Pair	Diff	t-value	df	p	adj.p	
b1-b4	-0.7944	7.4466	44	0.0000	0.0000	b1 < b4 *
b1-b3	-0.6722	7.1806	44	0.0000	0.0000	b1 < b3 *
b2-b4	-0.4222	5.7698	44	0.0000	0.0000	b2 < b4 *
b1-b2	-0.3722	5.4598	44	0.0000	0.0000	b1 < b2 *
b2-b3	-0.3000	5.0636	44	0.0000	0.0000	b2 < b3 *
b3-b4	-0.1222	2.5079	44	0.0159	0.0159	b3 < b4 *

< MULTIPLE COMPARISON for "B at a2" >

== Holm's Sequentially Rejective Bonferroni Procedure ==
== The factor < B at a2 > is analysed as dependent means. ==
== Alpha level is 0.05. ==

Pair	Diff	t-value	df	p	adj.p	
b1-b4	-0.9146	6.6444	40	0.0000	0.0000	b1 < b4 *
b1-b3	-0.7622	6.0396	40	0.0000	0.0000	b1 < b3 *
b2-b4	-0.5122	5.4720	40	0.0000	0.0000	b2 < b4 *
b1-b2	-0.4024	4.7831	40	0.0000	0.0001	b1 < b2 *
b2-b3	-0.3598	4.5218	40	0.0001	0.0001	b2 < b3 *
b3-b4	-0.1524	2.6665	40	0.0110	0.0110	b3 < b4 *

< MULTIPLE COMPARISON for "B at a3" >

== Holm's Sequentially Rejective Bonferroni Procedure ==
== The factor < B at a3 > is analysed as dependent means. ==
== Alpha level is 0.05. ==

Pair	Diff	t-value	df	p	adj.p	
b1-b3	-1.1630	8.4567	45	0.0000	0.0000	b1 < b3 *
b1-b4	-1.2772	8.0559	45	0.0000	0.0000	b1 < b4 *
b2-b3	-0.5380	7.1049	45	0.0000	0.0000	b2 < b3 *
b2-b4	-0.6522	6.1458	45	0.0000	0.0000	b2 < b4 *
b1-b2	-0.6250	4.6927	45	0.0000	0.0001	b1 < b2 *
b3-b4	-0.1141	1.3959	45	0.1696	0.1696	b3 = b4

< MULTIPLE COMPARISON for "B at a4" >

== Holm's Sequentially Rejective Bonferroni Procedure ==
== The factor < B at a4 > is analysed as dependent means. ==
== Alpha level is 0.05. ==

Pair	Diff	t-value	df	p	adj.p	
b1-b4	-0.7386	5.5411	43	0.0000	0.0000	b1 < b4 *
b2-b4	-0.5114	5.0540	43	0.0000	0.0000	b2 < b4 *
b3-b4	-0.2443	4.1099	43	0.0002	0.0007	b3 < b4 *
b1-b3	-0.4943	3.3709	43	0.0016	0.0048	b1 < b3 *
b2-b3	-0.2670	2.7247	43	0.0093	0.0185	b2 < b3 *
b1-b2	-0.2273	1.6656	43	0.1031	0.1031	b1 = b2

< MULTIPLE COMPARISON for "B at a5" >

== Holm's Sequentially Rejective Bonferroni Procedure ==
== The factor < B at a5 > is analysed as dependent means. ==
== Alpha level is 0.05. ==

Pair	Diff	t-value	df	p	adj.p	
b1-b4	-1.2955	8.3736	43	0.0000	0.0000	b1 < b4 *
b1-b3	-1.0114	8.1875	43	0.0000	0.0000	b1 < b3 *
b2-b4	-0.7557	6.4081	43	0.0000	0.0000	b2 < b4 *
b1-b2	-0.5398	6.2765	43	0.0000	0.0000	b1 < b2 *
b2-b3	-0.4716	5.3819	43	0.0000	0.0000	b2 < b3 *
b3-b4	-0.2841	4.6056	43	0.0000	0.0000	b3 < b4 *

output is over --------------------///
